# A network-based framework for shape analysis enables accurate characterization of leaf epidermal cells

**DOI:** 10.1038/s41467-020-20730-y

**Published:** 2021-01-19

**Authors:** Jacqueline Nowak, Ryan Christopher Eng, Timon Matz, Matti Waack, Staffan Persson, Arun Sampathkumar, Zoran Nikoloski

**Affiliations:** 1 School of Biosciences, University of Melbourne, Parkville, VIC 3010 Australia; 2 Bioinformatics, Institute of Biochemistry and Biology, University of Potsdam, 14476 Potsdam, Germany; 3 Systems Biology and Mathematical Modelling, Max Planck Institute of Molecular Plant Physiology, 14476 Potsdam, Germany; 4 Plant Cell Biology and Microscopy, Max Planck Institute of Molecular Plant Physiology, 14476 Potsdam, Germany; 5 Joint International Research Laboratory of Metabolic & Developmental Sciences, State Key Laboratory of Hybrid Rice, School of Life Sciences and Biotechnology, Shanghai Jiao Tong University, Shanghai, China; 6 Department for Plant and Environmental Sciences, University of Copenhagen, 1871 Frederiksberg C, Denmark; 7 Copenhagen Plant Science Center, University of Copenhagen, 1871 Frederiksberg C, Denmark

**Keywords:** Cytological techniques, Cell growth, Image processing, Morphogenesis, Plant cell biology

## Abstract

Cell shape is crucial for the function and development of organisms. Yet, versatile frameworks for cell shape quantification, comparison, and classification remain underdeveloped. Here, we introduce a visibility graph representation of shapes that facilitates network-driven characterization and analyses across shapes encountered in different domains. Using the example of complex shape of leaf pavement cells, we show that our framework accurately quantifies cell protrusions and invaginations and provides additional functionality in comparison to the contending approaches. We further show that structural properties of the visibility graphs can be used to quantify pavement cell shape complexity and allow for classification of plants into their respective phylogenetic clades. Therefore, the visibility graphs provide a robust and unique framework to accurately quantify and classify the shape of different objects.

## Abstract (web summary)

While cell shape is crucial for function and development of organisms, versatile frameworks for cell shape quantification, comparison, and classification remain underdeveloped. Here, the authors use a network-based framework for Arabidopsis leaf epidermal cell shape characterization and classification.

## Introduction

There is a myriad of shapes in nature but understanding their origin and evolution remains a challenge^1^. Differences in shapes are not only observed between categories of multicellular eukaryotes but are also found on the level of their individual cells. The shape of cells varies from simple to highly complex and can alter during the course of development^2^. Cell shape is thought to be an integrated result of subcellular processes and the forces acting on the cell^3^ and often dictates cellular functions^4^. The development of quantitative shape descriptors is the first step to enable comparison of cell shapes as well as to probe their relationship with cellular processes and effects on cellular functions. Therefore, a quantitative descriptor of shape must be rich enough to: (i) obtain global insights in differences among shapes, (ii) specify key characteristics of individual shapes, and (iii) precisely reconstruct the cell shape in silico.

Shapes can be compared based on two classes of global descriptors: region-based, quantifying key shape properties (e.g., area, convex hull, eccentricity, medial axis, and combinations thereof), and boundary-based (e.g., Fourier descriptors). Descriptors of shape can be obtained by following one of two principal approaches: structural (discrete) approaches, which divide the shapes into subparts, and global (continuous) approaches, which analyze shapes in their entirety^5^. The resulting descriptors facilitate the comparison of cell shapes but may introduce bias based on the properties of the descriptors employed. For instance, comparison based on distances between region-based descriptors is size-dependent and is thus unsuitable for developmental studies^6^. While ratios of region-based descriptors overcome this issue, they provide limited information for shape comparison^7^. The usage of landmarks positioned along the shape boundary facilitates easier comparison of shapes, although landmarks of different shapes have to be superimposed to account for scaling, rotation, and translation^8^. Further, due to the lack of common landmarks in cell shapes, these points have to be placed manually or by arbitrary sampling, thus rendering automated analyses challenging.

A more sophisticated type of boundary-based descriptors cast the closed two-dimensional shape boundary as a sum of ellipses by applying the Fourier Transform^9^. The resulting descriptor is similarity invariant, resulting in a shape descriptor independent of shape rotation, orientation, and scaling^10^. Another type of size- and orientation-invariant boundary-based shape descriptor characterizes a two-dimensional shape as a linear combination of sequential boundary samples following the autoregressive model approach^11^. However, the versatility of these descriptors to simultaneously address the problems of shape comparison, specification of key properties, and characterization of cell shape complexity remain inadequate.

The puzzle-shaped pavement cells that appear on the cotyledon and leaf epidermis of many plants represent complex cell shapes with specific local features^12^. These local features are given by the convex and concave parts of the cell boundary, namely cell protrusions, referred to as lobes, and invaginating regions termed necks. Lobes and necks have been used to compare differences in pavement cell shape^13^. Despite efforts to devise descriptors that simultaneously identify differences between pavement cells across different taxa and accurately characterize their local shape features^6,14,15^, comparative analyses that assess the performance based on a gold standard are rare^6^. Such efforts can be regarded as a necessary step toward understanding the contribution of cellular processes in the emergence of complex cell shapes^16–19^. Here, we propose a unique network-based shape representation, called visibility graph, whose properties can serve both as global and local shape descriptors. We refer to the corresponding framework as GraVis.

## Results

### A visibility graph is a graph-based representation of shape

**Fig. 1:**
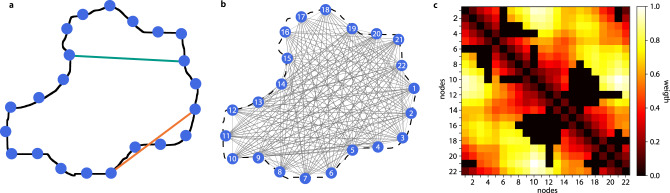
Visibility graphs as a descriptor of shape. **a** Contour of an illustrative shape. Nodes (blue) are placed equidistantly along the contour and used to create a visibility graph. Two nodes are connected in the visibility graph if they can “see each other”, i.e., the segment connecting them does not cross or align with the contour (teal edge); otherwise, the nodes cannot see each other (orange edge). The edges between nodes can be weighted according to their edge length (Euclidean distance *d*_*E*_). **b** Visualization of the visibility graph for the cell in (**a**). **c** Heatmap of the weighted and scaled adjacency matrix of the visibility graph (**a**), where edges with a weight of zero indicate the absence of said edge, thus indicating “non-visibility” between the nodes. The weighted visibility graph is used for local feature extraction, while the unweighted graph is used for global shape description and shape comparison. Source data are provided as a Source Data file.

We represent the shape, defined by the shape contour, with a visibility graph. The contour is given by a one-pixel border around the defined shape. The visibility graph is a mathematical structure that is fully specified by the set of nodes and a set of edges, connecting pairs of nodes. The nodes of the visibility graph correspond to pixels of the contour and are equidistantly placed along the contour (Fig. 1a). A node can be viewed as a person standing next to one side of a wall that represents the shape boundary. Two nodes are then connected by an (undirected) edge if they can see each other, i.e., the segment that connects them does not cross or align with the shape boundary (Fig. 1a). Testing this condition for every pair of nodes results in the set of edges, altogether specifying the visibility graph for the analyzed shape (Fig. 1b).

The visibility graph can be represented by its adjacency matrix, *A*, whose rows and columns correspond to the nodes. A nonzero value of a matrix entry indicates that there is an edge between the two nodes, corresponding to a matrix row and column. If all entries in the adjacency matrix take the value of either 0 or 1, the graph is referred to as unweighted. If other values can be assigned to the edges, the graph is said to be weighted. In a weighted visibility graph, the edges are assigned the Euclidean distance, *d*_*E*_, between the positions of the nodes (Fig. 1c). The edge weights can further be scaled to the maximum value over all edges in the network, resulting in a scaled weighted visibility graph. It can be easily deduced that the unweighted visibility graph and the scaled weighted visibility graph are scale-invariant, i.e., are not dependent on the size of the entity whose shape is analyzed. In addition, both the unweighted- and the scaled weighted visibility graphs are orientation- and rotation-invariant. The weighted visibility graph is in one-to-one correspondence with the actual shape if a single node for every pixel on the boundary is used. To provide a high-quality approximation, we estimate the pixel distance between node placement based on the image resolution and contour length (see “Methods”). As a result, the same shape acquired under different resolutions may have different numbers of nodes.

The visibility graph is a fundamental combinatorial structure in computational geometry to represent a set of objects along with a visibility relation between them. Depending on how the objects are defined, three primary types of visibility graphs are used to characterize, recognize, and reconstruct the objects^20,21^, and they have found various applications (Supplementary Fig. 1). For instance, when the visibility graphs are formed on the vertices of multiple polygons, they can be used for computation of shortest paths among polygonal obstacles, with applications in robotics^22^; when nodes correspond to the amplitude of particular time points, visibility graphs can characterize properties of time series^23^; when the nodes are placed along with a piecewise linear approximation of a shape, network clusters in the resulting visibility graph have been used for shape decomposition, but not for shape comparison^24,25^. Our visibility graph concept also differs from the shape context^26^, used for shape matching. The shape context provides a descriptor based on the length and rotation of neighboring points on the contour that can be used to measure the similarity between two objects. However, visibility graphs have not yet been used for shape comparison and characterization of local and global properties of shape, which is the main contribution of our study.

### Comparison of visibility graphs

**Fig. 2:**
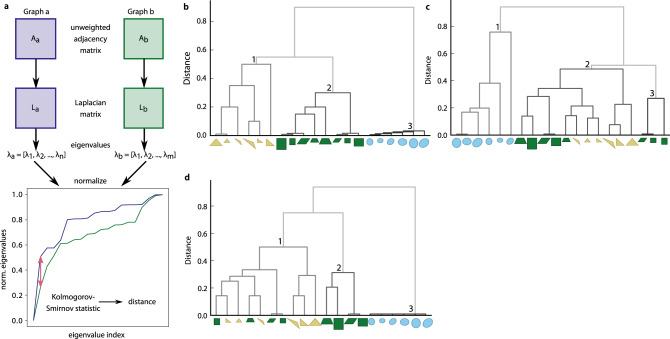
Comparison of unweighted visibility graphs. **a** Two visibility graphs are compared based on the spectrum (i.e., distribution of eigenvalues) *λ* of the Laplacian *L* obtained from the unweighted adjacency matrices *A*. The Kolmogorov–Smirnov statistic is used to quantify the difference between the distributions of two spectra. **b**–**d** Clustering dendrogram obtained by the procedure for a set of synthetic, simple triangular, circular, and rectangular shapes with **b** the same number of nodes, **c** variable number of nodes between the network representations of the shapes, and **d** variable number of nodes which were reduced using modularity clustering to have the same number of nodes. Source data are provided as a Source Data file.

While visibility graphs with an equal number of nodes can be compared based on Euclidean distance of their adjacency matrices, after appropriate rotation (see Supplementary Note 1, Supplementary Fig. 2 for illustration of rotational distance), the comparison of visibility graphs with a different number of nodes requires a distance measure applicable to matrices of a different dimension. A suitable and easy-to-implement distance measure for visibility graphs with a different numbers of nodes relies on the comparison of the distribution of entries, or on the distribution of eigenvalues, of the matrices associated with the graphs. These distributions provide a summary of values that characterize the graphs. We propose to compare the unweighted visibility graphs based on the differences in the distribution of eigenvalues of the Laplacian matrix (i.e., another matrix representation of a given graph), *L* = *A* − *D*, with *D* denoting the diagonal matrix of node degrees (Fig. 2a). The distribution of the eigenvalues of a Laplacian, which are guaranteed to be nonnegative, have already been used to compare images^27^. To quantify the distance between the distributions of eigenvalues for two visibility graphs we use the Kolmogorov–Smirnov statistic (Fig. 2a)^28^. The resulting distances yield a distance matrix that can be employed in clustering.

We test the proposed approach for comparison of visibility graphs obtained from simple, synthetic shapes. To this end, we create a set of 20 shapes divided into three groups: triangular (i.e., right-angled, equilateral, and obtuse), rectangular (i.e., square, rectangle, trapeze, and rhombus), and circular (i.e., circle, ellipse, and rotated ellipse), each in two sizes. The coordinates for each shape are manually defined with uniform spacing of nodes, such that either all shapes have the same number of nodes or the number of nodes can vary (see Table 1).

**Table 1:** Properties of the visibility graphs of the synthetic set with equal and unequal number of nodes.

Shape	Equal				Unequal			
	Small		Large		Small		Large	
	*n*	*m*	*n*	*m*	*n*	*m*	*n*	*m*
*Triangular*								
Rectangular	20	132	20	112	14	64	28	256
Equilateral	20	128	20	128	16	84	24	192
Obtuse	20	125	20	138	14	63	29	276
*Rectangular*								
Square	20	150	20	150	12	54	32	384
Rectangle	20	148	20	148	16	91	40	592
Trapeze	20	146	20	144	14	69	34	424
Rhombus	20	150	20	148	12	54	32	384
*Circular*								
Circle	20	190	20	190	24	276	32	496
Ellipse	20	190	20	190	22	231	32	496
Rotated ellipse	20	190	20	190	20	190	33	528

*Note*: Each shape in the synthetic set was used to generate a smaller and a larger visibility graph. For each graph, the number of input coordinates (nodes), $\left| V \right| = n$, and the corresponding number of edges, $\left| E \right| = m$, are shown.

We then use the defined coordinates of the shapes as node positions, create the corresponding visibility graphs, and quantify their pairwise distances using the described method based on eigenvalue distributions (Fig. 2b, c, see Supplementary Fig. 2b for rotational distance). By using hierarchical complete-linkage clustering with the resulting distance matrices, we find that the shapes are accurately grouped into rectangular, triangular, and circular when all shapes are described with the same number of nodes (Fig. 2b).

In addition, we show that our distance measure provides good clustering of the shapes even when different numbers of nodes are used in building the visibility graphs corresponding to the synthetic shapes (Fig. 2c). We further test the Laplacian distance measure based on the weighted adjacency matrices of the visibility graphs for the set of synthetic shapes; however, this approach does not result in improved clustering (Supplementary Fig. 3). These results show that the structure of the visibility graphs, specified by the considered edges, is rich enough to capture global differences between shapes. In addition, they demonstrate that the eigenvalue-based distance measure is suitable to quantify differences between matrices of different sizes.

To compare visibility graphs with different numbers of nodes, we also design a procedure for the reduction of visibility graphs based on the modularity cluster quality measure^29^. This procedure allows the comparison of two graphs with different numbers of nodes by using the rotational distance (Supplementary Note 2, Supplementary Fig. 4). The rotational distance finds the superposition of the nodes of two visibility graphs such that the corresponding Euclidean distance of their adjacency matrices is minimized. To assess how our graph comparison compares to other methods, we also use the Fourier transform to compute the distance between the synthetic shapes in the simpler scenario in which they all have the same number of nodes (Supplementary Note 3, Supplementary Fig. 5a). Although Fourier transform is the most widely used approach for shape characterization, the resulting complete-linkage clustering shows that the shapes are not separated well into the three respective classes (Supplementary Fig. 5b–e).

We provide quantitative support for these results by calculating the Biological Homogeneity Index (BHI) to measure the quality of clusters (Supplementary Note 4)^30^. We find that GraVis has a perfect score for graphs with an equal number of nodes. In addition, using a different number of nodes and with and without reduction, based on modularity, GraVis still shows high cluster homogeneity and outperforms the approach based on Fourier transform (Supplementary Table 1). The rotational distance fares similarly well, whereas the Fourier transform results in the least homogeneous clusters (Supplementary Table 1).

To investigate the sensitivity of the algorithm to the spatial resolution of the node placement, we further used the set of synthetic shapes with the same number of nodes to illustrate the clustering quality based on different node densities. Therefore, we selected the large shapes (3 triangles, 4 squares, 3 circles) with each the equal number of nodes (*n* = 20) and used the node reduction method based on modularity clustering (Supplementary Fig. 4) to reduce the number of nodes of each graph stepwise, until all graphs contained 12 nodes. For all these graph sets we calculated the distance matrices and used them for hierarchical complete-linkage clustering (Supplementary Fig. 6). We then use the resulting clusters to compute the BHI and observe that it decreases for visibility graphs with a reduced number of nodes (Supplementary Fig. 7). The visibility graphs with 20 and 19 nodes per graph have a perfect score of 1.0 for all clusters, thus showing that the corresponding node density of 10–14 pixel/nodes is optimal for the detection of distinguishing global shape features. In addition, we observe that the BHI score is slightly below 0.9 for 17 and 18 nodes, demonstrating the robustness of our approach for small differences in node numbers. Changes in BHI for the number of nodes that differ by one is not larger than 36%.

Next, by comparing a set of selected pavement cells, which includes shapes along a gradient of complexity, we find that the Laplacian distance measure results in the most homogenous clusters, closely followed by the Fourier transform using the correlation distance (Supplementary Fig. 8, Supplementary Table 2). Therefore, we conclude that the proposed approaches for comparison of visibility graphs with the same or different number of nodes provide excellent ways for comparison of shapes and outperform classical solutions in the tested scenarios.

### Visibility graph as a global descriptor of shape

**Fig. 3:**
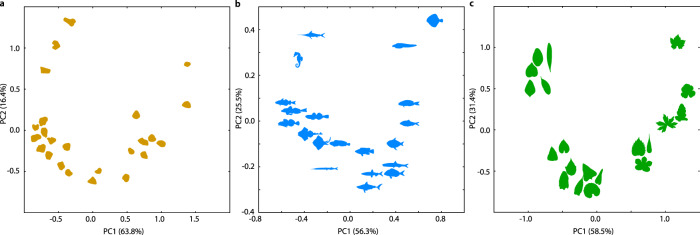
Comparison of shapes from different domains based on their visibility graphs. The distances between visibility graphs based on the spectra of their Laplacians can be used in multivariate analyses and visualizations to distinguish different groups of similar shapes. We show the principal component analyses of shapes from three different domains: **a** sand grains, **b** fish shapes, and **c** leaf shapes^13,31,32^. Source data are provided as a Source Data file.

We next test the ability of the visibility graphs to serve as global descriptors of shapes from different domains. We use images of 24 sand grains of the Australian coast, 20 fish shapes from the WoRMS database^31^, and 20 leaf shapes provided by Vöfely et al.^32^. We then create the corresponding visibility graphs and distance matrices for the shapes from the three different domains (see Supplementary Table 3). The principal component analysis (PCA) demonstrates that our approach can distinguish the shapes according to their complexity (Fig. 3). For example, the PCA of the fish shapes (Fig. 3b) can be split into three parts: in the upper left corner are the most complex shapes (i.e., seahorse and swordfish), whereas in the upper right corner are the simpler, smoother fish shapes (i.e., turbot and eelpout). The rest of the shapes are intermediates of the two extremes and are centered at the bottom, where again fish with smoother shapes, with fewer fins, are on the right side and more complex shaped fishes, with more fins, on the left of the PCA plot. The same trend can be seen for the sand grains (Fig. 3a) and laterally reversed for the leaves (Fig. 3c). We further compare the resulting shapes using the Fourier transform approach. The resulting PCAs show no obvious clusters or gradient of shape complexity. Thus, in contrast to the visibility graph approach, the Fourier transform analysis performs worse in separating the shapes based on their complexity (Supplementary Fig. 9). Together, these results show that visibility graphs can serve as powerful global descriptors of shape, and is applicable with data from different domains.

### Visibility graphs quantify the complexity of epidermal pavement cells

**Fig. 4:**
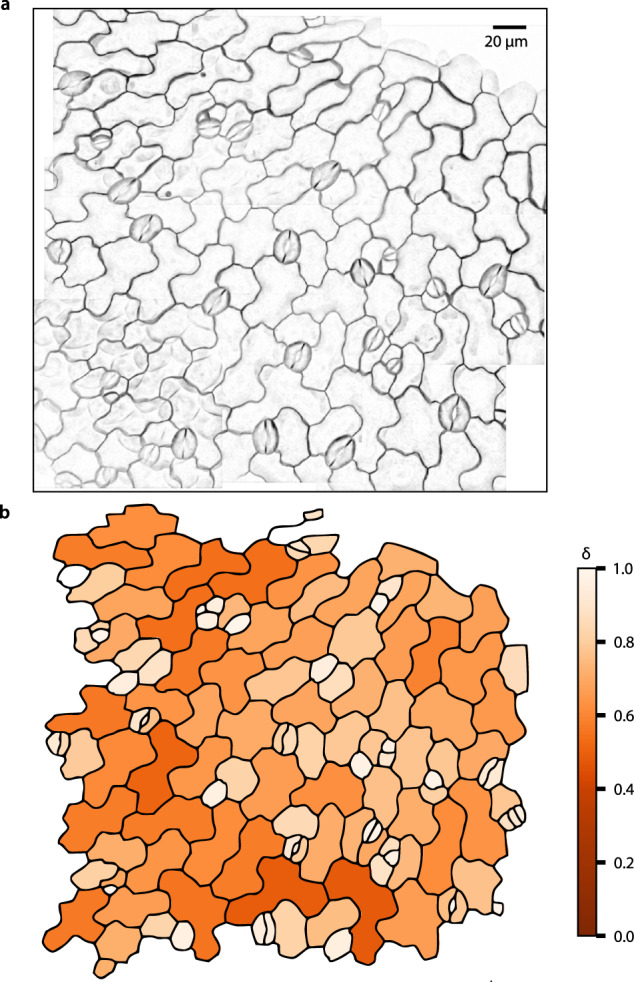
Heatmap of Arabidopsis pavement cell completeness. **a** Example microscopy image of Arabidopsis pavement cells at 96 h after germination (single image). **b** The original microscopy image can be recreated by plotting the extracted visibility graphs of all detected pavement cells and weighting them according to their relative completeness (*δ*), which is calculated by the relative density of the corresponding visibility graphs. Simple, round shapes like stomata have smaller relative completeness (white) in comparison to the more complex pavement cell shapes (dark orange). Source data are provided as a Source Data file.

In addition to shape clustering, the visibility graphs can be used to quantify the complexity of shapes. To illustrate this point, we use epidermal pavement cells, which are frequently employed to investigate morphogenesis^16,33,34^. During cell morphogenesis, pavement cells change from simple, polygonal shapes to more complex shapes with a defined neck (invaginations) and lobe (protrusions) domains. To depict the distribution of different cell complexities or the change of cell complexity over time, we can use the relative graph completeness of the corresponding visibility graphs. We quantify the (relative) graph density *δ* for undirected graphs by the ratio between the graph edges and the maximum number of edges in the graph (see “Methods”, Eq. 1). In a dense graph, the number of edges scales with the square of the nodes, i.e., it is close to the number of all possible edges on those nodes. As a result, round cells, where each node is visible by all others, will be represented by dense graphs, while more complex cells will yield sparser graphs. Therefore, global network properties, like the graph density, can be used to provide insights in the relative completeness of cell shape (i.e., how many edges are missing for the graph to become complete). For instance, stomatal pores (Fig. 4b), formed by two guard cells have a typical round shape and can be easily distinguished from complex pavement cells by using the graph density as a measure of relative completeness (dark orange, Fig. 4b).

Besides the here proposed relative completeness measure based on graph density, there are other measures to describe the complexity of pavements cells. These include the circularity, quantifying how close a shape is to a circle, and the number of lobes of pavement cells^6,14^ scored by GraVis (see Section on lobe quantification, below). Through a comparative analysis, we find that there is a significant positive Pearson correlation between the relative completeness and the circularity of pavement cells (*r* = 0.84, *p* value < 10^−35^, Supplementary Fig. 10a). Moreover, there is a significant negative Pearson correlation between the relative completeness and the number of lobes per cell without (*r* = −0.35, *p*-value < 10^−5^) and with tri-cellular junctions (*r* = −0.57, *p* value < 10^−12^, Supplementary Fig. 10c, d). Inspection of the correlation plots indicates that the visibility graph approach provides a more refined description of cell complexity since there are cells of small circularity and high relative completeness values (Supplementary Fig. 10a). In fact, the majority of cells with high relative completeness and small circularity belong to stomata cells, as shown in Supplementary Fig. 10b. In addition, the lack of perfect negative correlation to the number of lobes suggests that the relative completeness, as a continuous measure, offers a different aspect of quantifying cell complexity.

### Centrality measures of visibility graph characterize local shape features

**Fig. 5:**
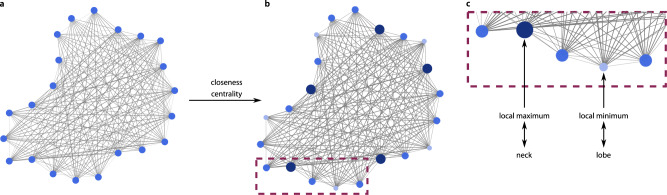
Detection of lobes and necks in pavement cells based on visibility graphs. **a**, **b** The closeness centrality is calculated for the nodes of a visibility graph, visualized by different node sizes. **c** Lobes (light blue nodes) and necks (dark blue nodes) correspond to the local minima and maxima along the contour, respectively.

While global properties of visibility graphs, such as the graph density, discussed above, allow for comparison of shapes, properties of nodes in the graph can be employed to quantify local shape features. To test this idea, we use centralities of nodes in visibility graphs of pavement cells to determine necks and lobes. Different centrality features can be used to characterize the position and role of nodes in the graph^35^. Nodes on positions of necks are expected to be on average closer, or more visible, to other nodes in comparison to lobes. Therefore, we use the closeness centrality of a node to quantify the likelihood that a node corresponds to a neck or lobe (Fig. 5a, b, see “Methods”, Eq. 2). We hypothesize that lobes and necks correspond to local minima and maxima of the closeness centralities along the contour, respectively (Fig. 5c). We find that the closeness centrality predicts lobes and necks with the highest accuracy, and outperforms predictions based on other centralities (Supplementary Note 5, Supplementary Table 4, Supplementary Fig. 11). We can furthermore discriminate the detected lobes as either true lobes (the indentation between two cells) or as tri-cellular junctions (sites where three cells meet)^36^ by superimposing the lobe positions with positions of detected tri-cellular junctions (see “Methods” and Supplementary Fig. 12). In addition, GraVis can be used to also measure distinct shape parameters, including the cell area, the circularity, the lobe length, and the neck width, to address particular questions relevant to shape analysis^14,37^.

### GraVis accurately quantifies lobe number

Our network-based framework, GraVis, relies on different network properties to analyze shapes. Next, we compare the performance of GraVis with respect to lobe detection against three contending lobe detection tools, namely: LobeFinder, PaCeQuant, and LOCO-EFA^6,14,15^. All three tools use a boundary-based approach to detect the lobes of pavement cells: LobeFinder uses a convex hull-based algorithm to identify lobe positions. PaCeQuant detects lobes by looking for local changes in the contour curvature orientation, while LOCO-EFA uses an advanced elliptical Fourier analysis where the contour is decomposed into a set of unique coefficients. The number of lobes in the latter can be inferred from the resulting ellipse profile which shows the contribution of specific modes.

**Fig. 6:**
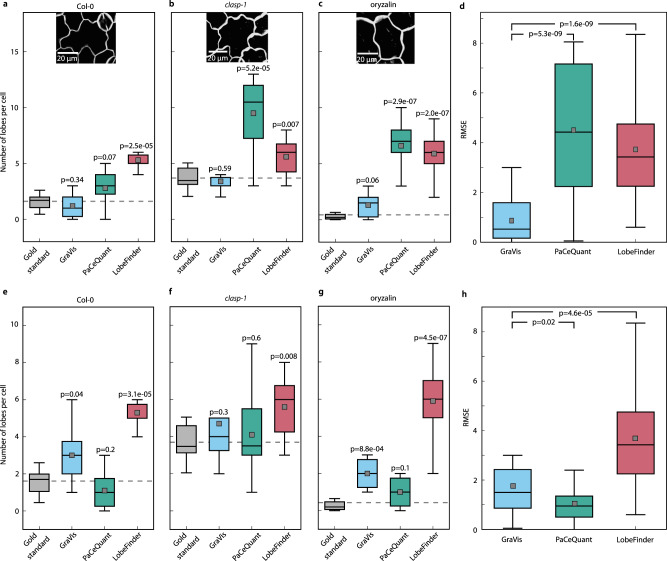
Comparison of visibility graphs with other contending approaches to counting the number of lobes without tri-cellular junctions in leaf pavement cells. The comparison involves three computational approaches: PaCeQuant^14^, based on the contour curvature, LobeFinder^6^, based on the convex hull, and LOCO-EFA^15^, based on elliptical Fourier analysis. Since LOCO-EFA does not distinguish between true lobes and tri-cellular junctions, it was removed from the comparison, but is included in the comparison of tools based on the total number of lobes (Supplementary Fig. 15). The quantitative comparison is based on a gold standard that includes *n* = 30 Arabidopsis pavement cells obtained from each of the three scenarios: **a**, **e** wild type, **b**, **f** *clasp-1* mutant, and **c**, **g** oryzalin treatment, all with manually annotated lobes by 20 experts. The *clasp-1* data used for the gold standard (96 h after germination) differ from the *clasp-1* data used in the comparison of different genotypes (120 h post dissection), as the images were captured at different time points. Shown is the competitive comparison using **a**–**d** default parameters and **e**–**h** tuned parameters to detect the number of lobes without tri-cellular junctions. The dotted line denotes the mean of the gold standard, i.e., manual detection by 20 experts, for the respective scenario. While GraVis performs best based on the residual mean square error (RMSE) using **d** default parameters, it performs as good as PaCeQuant using **h** tuned parameters. *p* values were determined with a two-sided two-sample *t* test and were Benjamini–Hochberg adjusted. Boxplots are shown with median (horizontal line), mean (gray square), 25th and 75th percentiles (box edges), and 1.5-fold of the interquartile range (whiskers). Source data are provided as a Source Data file.

To compare the lobe detection performance of these contending approaches, similar to PaCeQuant, we create a gold standard of pavement cell shapes by manual curation of lobes. To this end, we select 30 pavement cells from 3 different scenarios involving *Arabidopsis thaliana* wild type, oryzalin-treated wild type, and the *clasp-1* mutant. Oryzalin, an inhibitor of microtubule polymerization^38^, is known to inhibit de novo lobe formation and growth of established lobes^39^, while *clasp-1* is a mutant allele of the *CLASP* gene that encodes a regulator of microtubules that impacts pavement cell morphology^40^. We first look at the number of lobes that were detected manually by 20 experts, yielding the gold standard (Supplementary Fig. 13). A detailed evaluation of the consensus of manually detected lobes reveals that, as expected, more pronounced protrusions are detected by the majority of experts, while smaller protrusions are detected by single or only a few individuals (Supplementary Fig. 14). We then compare the number of manually detected lobes to the number of lobes detected by the approaches in each scenario, in which we exclude tri-cellular junctions (Fig. 6a–c). We excluded LOCO-EFA from the analysis since it does not provide information about whether or not detected lobes are tri-cellular junctions. For all three scenarios, with the default parameters for all approaches, the number of detected lobes by GraVis is closer to the mean of the gold standard compared to the other tools (dotted line, Fig. 6a–c) and shows no significant difference to the mean of the gold standard.

To show the accuracy of the approaches with respect to the gold standard, we calculate the root mean square error (RMSE) based on the mean of manually detected and the number of predicted lobes based on default parameter values (Fig. 6d). Our findings demonstrate that GraVis exhibits the lowest RMSE and performs better than the other two compared approaches for quantifying the number of lobes when default parameters are used. We test the significance of the RMSE between GraVis and the other tools using a two-sample *t* test. The RMSE mean of PaCeQuant is 5.2 times higher than that of GraVis (*p* value: 5.28 × 10^−7^), the mean RMSE for LobeFinder is 4.3 times higher (*p* value: 1.61 × 10^−9^). The comparison based on a number of lobes including tri-cellular junctions shows similar findings, with the addition that LOCO-EFA has an RMSE that is 3.9-fold higher in comparison to that of GraVis (*p* value: 1.24 × 10^−7^) (Supplementary Fig. 15a–d). Calculating the difference between the RMSE and the median of manually detected lobes confirms the results that GraVis outperforms the other tools when default parameters are used (Supplementary Fig. 16).

We note that the default value for the number of nodes used, as the only parameter in GraVis, is determined by using the gold standard to calculate the optimal distance between node pixels that depends on the image resolution (Supplementary Note 6, Supplementary Fig. 17). We emphasize that the optimal distance found through this analysis is 0.65 node/µm, which is in turn used in all remaining analyses (see “Methods”). Nevertheless, it can be expected that tuning of the parameter values for the other tools may lead to better performance in comparison to the default settings. To test how the performance depends on the parameter values used, we tune the three parameters (i.e., Gaussian *σ* in curvature analysis, minimal length of a protrusion section, and the minimal length of an indentation section) for PaCeQuant and one parameter (i.e., the threshold between consecutive modes) for LOCO-EFA on a training set of 20 cells randomly sampled from the gold standard (Supplementary Fig. 18). The performance is then evaluated on the remaining 10 cells, as a test set, with respect to the RMSE with the best-performing parameter values on the training set (Supplementary Fig. 18). The parameter values resulting in the smallest overall RMSE are in turn used for the final comparison of performance, demonstrating that both in the cases with and without tri-cellular junctions GraVis performs better or as good as the other tools with respect to the mean (Fig. 6h, Supplementary Fig. 15h). Interestingly, GraVis shows a smaller variance in the RMSE in comparison to all other tools (Supplementary Table 5, Fig. 6, Supplementary Fig. 15). This finding demonstrates that tuning of the contending tools, unlike GraVis, should be done on a case-by-case basis to reduce the variance of predictions, which may severely bias comparisons in different biological scenarios (e.g., unseen genotypes and cell types).

We also calculate the percentage of recovered tri-cellular junctions for each tool and find that PaCeQuant detects all tri-cellular junctions, followed by GraVis that recovers almost all tri-cellular junctions using default parameters (Supplementary Fig. 15i), whereas the percentage of recovered tri-cellular junctions decreases for PaCeQuant and GraVis using tuned parameters (Supplementary Fig. 15j). A detailed analysis of recovered junctions for the manually annotated lobes of the gold standard shows that there is variance in the detection of tri-cellular junctions among the experts (Supplementary Fig. 19)—which is expected in different applications^6,14^.

### GraVis distinguishes genetically modified lines with effects on pavement cell shape

**Fig. 7:**
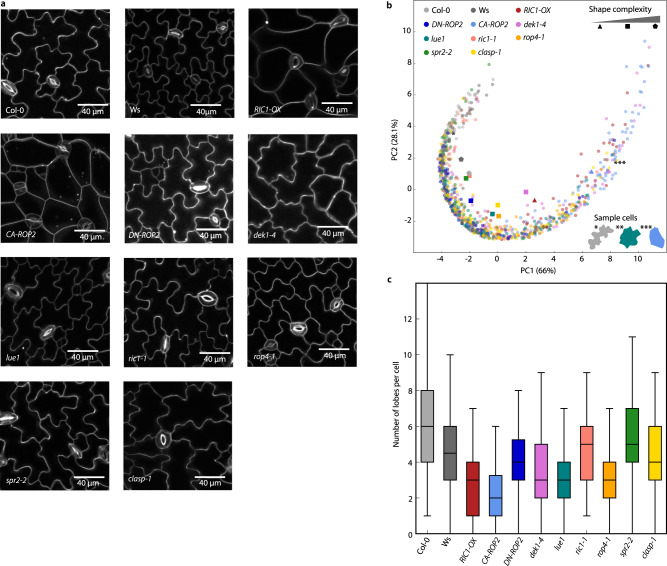
Comparison of pavement cells based on visibility graphs distinguishes Arabidopsis wild-type and genetically modified lines. **a** Images of LTi6b-GFP expressing Arabidopsis wild-type (Col-0, WS) and genetic lines (*RIC1-OX*, *CA-ROP2*, *DN-ROP2*, *dek1-4*, *lue1*, *ric1-1*, *rop4-1*, *spr2-2*, *clasp-1*). For all genetically modified lines we captured six images, for *DN-ROP2* we captured four images. **b** PCA of distances (*n* = 80 pavement cells per sample from different images) between pavement cells of wild-type Col-0 and WS (light gray and dark gray, respectively) and expression lines: *RIC1-OX* (red), *CA-ROP2* (light blue), *DN-ROP2* (dark blue), *dek1-4* (pink), *lue1* (teal), *ric1-1* (salmon), *rop4-1* (orange), *spr2-2* (green), and *clasp-1* (yellow). The circles correspond to selected cells, and the other shapes denote the center of mass for the corresponding expression lines. We used triangles for genetic lines with simple cell shapes (*RIC1-OX*, *DN-ROP2*), pentagons for very complex shapes (Col-0, Ws), and squares for shapes in between. Sample cells for Col-0 (light gray), *CA-ROP2* (light blue), and *lue1* (teal) and are shown in the lower right corner. **c** Boxplot of a number of detected lobes for wild-type and mutants (80 pavement cells per sample). Boxplots are shown with median (horizontal line), 25th, and 75th percentiles (box edges), and 1.5-fold of the interquartile range (whiskers). Source data are provided as a Source Data file.

To show that GraVis is powerful in detecting differences of local features in pavement cells, we use different genetically modified lines in which the number and size of lobes and necks are affected (see Fig. 7c). For each line, we manually select 80 pavement cells to generate the visibility graphs and the corresponding distance matrices. The *clasp-1* images used for this comparison were captured at a different time point than the *clasp-1* data used for the manual lobe detection in the gold standard. By applying GraVis we could separate three distinct regions in the PCA plot. In the lower-left corner, we identify two lines with defects in lobe formation that display reduced shape complexity (*RIC1-OX*, *CA-ROP2*, Fig. 7a)^41^, whereas on the right, we identify a cluster consisting of the two wild-type ecotypes (Col-0, Ws, Fig. 7a) that have highly complex pavement cells. The remaining shapes are between these two extremes and comprise the other lines (*DN-ROP2*, *dek1-4*, *lue1*, *ric1-1*, *rop4-1*, *spr2-2*, *clasp-1*), with an increase of cell complexity from left to right. The number of nodes per unit of contour length in the visibility graph from pavement cells of the analyzed is similar (with some variability across cells within a line, Supplementary Fig. 20). The boxplots of the detected number of lobes, excluding tri-cellular junctions, show a similar result as the PCA (Fig. 7b). Hence, the wild-type ecotypes have the highest number of detected lobes, whereas *RIC1-OX* and *CA-ROP2* have the lowest numbers of detected lobes. We test whether there is a difference in means between the genotypes using the Kruskal–Wallis test, and find that there is both a significant difference for the number of detected lobes without tri-cellular junctions (*p* value < 10^−31^) and with tri-cellular junctions (*p* value < 10^−28^). Further, we test which genotypes show differences in means and create a graph, where the nodes show the genotypes that are connected by edges if there is no difference in mean (*p* value > 0.05, Supplementary Table 6, Supplementary Fig. 21). The clustered genotypes in the graph correspond to the clusters in the PCA (Fig. 7a). While there are a plethora of studies focussing on pavement cell shapes, these studies have used various manual methods to quantify and assess these phenotypes^13,36,39^. By contrast, GraVis can be used to distinguish genotypes based on their pavement cell shapes in an automated fashion, thus enabling efficient genetic screens in future studies.

### GraVis classifies plants into their phylogenetic clade

Plants can be classified into respective taxa and clades based on their leaf shape^42^. However, it remains unclear if the shape of individual cells also allows for accurate classification. To test this hypothesis, we use GraVis in combination with machine learning approaches to classify pavement cells from different vascular plant clades. To this end, we use manually extracted pavement cells from the data set published by Vöfely et al.^13,32^. To predict the phylogenetic clade for a pavement cell, we use the adaxial pavement cells from five different phylogenetic clades: eudicots, monocots, ferns, angiosperms, and gymnosperms. Due to the small sample size and close phylogenetic relationship, the pavement cells from angiosperms and gymnosperms are treated as a single class (Table 2). We use a multiclass support vector machine (SVM) with a Gaussian kernel and trained a model with 80% of the data set. To build the multiclass SVM, we use the well-established one-against-all strategy with accuracy as a measure of performance (see “Methods”). As features, we use the number of lobes along with the minimum, maximum, and selected moments (mean, median, skewness, and kurtosis) of different centrality measures of the visibility graphs representing the pavement cells (see details in Supplementary Information file).

**Table 2:** Prediction of plant clades using nested SVMs.

Clade	Samples	Training set	Accuracy			Test set	Accuracy	Precision	Recall
			SVM1	SVM2	SVM3				
Ferns	1176	919	0.84	–	–	257	0.92	0.87	0.72
Eudicots	3534	2841	0.78	0.75	–	693	0.78	0.76	0.89
Angio-/gymnosperms	209 + 570	167 + 468	0.72	0.71	0.74	42 + 102	0.86	0.55	0.30
Monocots	870	692	0.74	0.73	0.73	178	0.83	0.11	0.12
Total	6359	5087				1272	0.85	0.57	0.51

*Note*: Pavement cells of five different plant clades were used for prediction. The two minority classes angiosperms and gymnosperms were merged. The multi-class SVM was trained on 80% of the total dataset, the remaining 20% were used as a test set to evaluate the resulting SVMs.

We show that ferns can be distinguished from the rest of the clades with high accuracy of 84%. As a result, following the one-against-all strategy, we remove the fern samples from the training set and repeat the procedure with the samples from the remaining clades. The last SVM was built based on the training data for monocots and the merged samples of angio- and gymnosperms. Finally, we use the test set (remaining 20%) to evaluate the overall performance of the SVM models. The average accuracy on the test set is 85%, while the average precision and recall are 57% and 51%, respectively (Table 2).

Furthermore, we use the extracted visibility graphs of all five clades to calculate a distance matrix which we use in a PCA. Indeed, in line with our SVM results, the ferns are easily distinguishable from the other clades (blue), while the other four clades are harder to differentiate (Supplementary Note 7, Supplementary Fig. 22).

In addition, we compare different shape features that were previously used by Vöfely et al.^13^, e.g., the solidity and aspect ratio, with features we extracted with GraVis, e.g., the relative completeness and number of lobes per cell. The Kruskal–Wallis test indicates that there are significant differences in means between the five clades based on the four compared properties (Supplementary Fig. 23). However, Dunn’s post hoc test indicates that the aspect ratio and the number of lobes could not separate the angiosperms and eudicots of the ten compared pairs of clades (Supplementary Table 7). Furthermore, the relative completeness could not separate the angiosperms and monocots and the solidity could not distinguish differences between angiosperms and gymnosperms. In conclusion, the solidity, the aspect ratio, the number of lobes, and the relative completeness are able to distinguish most of the pairs. We note that this analysis does not allow to classify a particular pavement cell into the respective clade, since it is based solely on the comparison of means between clades. To provide a more sophisticated strategy, it might be promising to do a joint analysis of multiple properties to distinguish the shapes of the different plant clades.

## Discussion

Although decades of research efforts have generated diverse descriptors of shapes, their ability to quantify cell shapes and characterize their subtle differences generally remains poor (5, 8, 10). For instance, quantification of shape variations based on Elliptic Fourier analysis has been widely used in biological studies, although it fails in comparison of simple shapes (see Supplementary Fig. 5)^43^. In addition, an extension of the classical descriptors to quantify the number of lobes and necks in cells with complex shapes, like pavement cells in the leaf epidermis, does not result in accurate measurements (see Fig. 6)^6,14,15^.

Since the shape of cells is important for their functionality and development, its accurate quantification can be used to understand genetic basis and programs that control diverse features of cell shape. Here, we propose the concept of a visibility graph as a versatile descriptor of shapes, which is at the core of the GraVis framework. Our network representation extends the visibility graph analysis of time-series data and visibility graphs as used in computational geometry^23^. In both representations, a node denotes a point location, on a timeline or in space, and an edge represents a visible connection between nodes. Rather than representing polygonal obstacles, our visibility graph is used to characterize the shape of a given object by placing nodes along its contour. The main advantage of this shape representation is that it allows the calculation of a variety of local and global network properties to facilitate comparison and extraction of relevant shape characteristics. The network properties can readily be used as features in machine learning approaches.

Following these ideas, we demonstrate that the GraVis framework facilitates comparison of shapes from different domains, accurate quantification of lobes and necks in pavement cells in a fully automated fashion, adding functionality in comparison to the state-of-the-art contenders (of which PaCeQuant is the only automated tool) with and without parameter tuning. In addition, we show that GraVis facilitates accurate classification of plants into respective clades based on the shapes of their adaxial pavement cells. However, the identification of network properties that can be directly related to key shape characteristics may be an involved process. For instance, while we show that the minima of closeness centrality along the contour of a cell coincides with the number of lobes, we also exclude that other centrality measures provide a better means for quantification of this shape feature. In addition, while we show that the features of the visibility graph can be used to infer new biological insights that impact shape, the comparison and mining of graphs will require capitalizing on the recent development in graph alignment and graph kernels^44,45^.

Prospective applications for GraVis include associating the network properties of the visibility graphs with key cellular processes and structures as well as tracking their temporal changes. We expect that accurate quantification of key shape features of different genotypes, such as that of GraVis, may be used to define shape changes across development and facilitate genetic screens to determine the genetic basis of morphogenesis. Finally, our framework can be used to gain a deeper understanding of the interrelation between cells and organ shapes.

## Methods

### Data sets of simple synthetic shapes and pavement cells

We created a synthetic set of simple shapes including three types of triangular (right-angled, equilateral, and obtuse), three types of circular (circle, ellipse, and rotated ellipse), and four types of rectangular shapes (square, rectangle, trapeze, and rhombus). For each object, we created a smaller and a larger shape by using graphs with equal as well as different numbers of nodes (Table 1). We also created a set of 12 pavement cells, which were selected from images of three genotypes (Col-0, *lue1*, *CA-ROP2*). For each cell, we created the visibility graph obtained using the optimal distance based on the image resolution.

### Shape data sets from different domains

We used shape data sets from three different domains: sand grains, fish, and leaves for which we collected sand from different beach locations on the Australian coast. Images were captured using a brightfield microscope and a dark background (Leica M205 FA stereomicroscope, 8.2×). Here, we selected 24 unique shapes from a single image for further processing. Furthermore, we chose 20 open-access images of fish drawings from different subfamilies from the marine species database WoRMS^31,46^. We also selected 20 images of leaf shapes collected from 278 different vascular plant taxa which were openly accessible^13,32^. All selected images were segmented by converting them to grayscale and binarizing them using either a mean or Otsu thresholding. Small particles and holes were then removed. Furthermore, the petioles of leaves were removed to focus only on the shape of the leaves themselves. The visibility graphs for all shapes were created and compared within their corresponding data sets.

### Microscopy images of pavement cells of wild-type and mutants

For imaging of the 96 h post-dissection cotyledons, seeds in the wild-type and *clasp-1* background expressing *35S*_*promotor*_*:LTi6b-GFP*^47^ were sterilized in 70% ethanol and rinsed three times with sterilized dH_2_O before being stored in dH_2_O in the dark at 4 °C for 3 days. Embryos were dissected from the seed coat and the cotyledons were removed from the rest of the embryo. Samples were then mounted under 0.5% micro-agar supplemented with or without 1 µm oryzalin (Sigma-Aldrich) using custom-made chambers with coverslip glass bottoms. Samples were moved to a 21 °C growth cabinet with a 16-h light regiment. Z-stacks (0.3 µm step sizes) of adaxial cotyledon surfaces proximal to the petiole were acquired 96 h post-dissection on a spinning-disk confocal microscope (Roper Scientific) with a 60× objective lens. Overlapping regions of interest were stitched together using the stitching plug-in for ImageJ^48,49^.

For imaging of the wild-type (Col-0, Ws eco-types), *RIC1-OX*, *CA-ROP2*, *DN-ROP2*, *dek1-4*, *ric1-1*, *spr2-2*, *rop4-1*, *clasp-1*, and *lue1* lines^41,50–52^, seeds were sterilized in 70% ethanol and rinsed three times with sterilized dH_2_O and plated onto half-strength Murashige and Skoog media with 1% micro-agar. For all genetically modified lines, we captured six images, except for *DN-ROP2*, for which we captured four images. Plates were stored in the dark at 4 °C for 3 days before being grown vertically in a 21 °C growth cabinet with a 16-h light regiment for five days. Seedlings were incubated in 0.2 mg/mL of propidium iodide (Sigma-Aldrich) for 2 min followed by two rinses with water. Z-slices (0.5 µm step sizes) of adaxial cotyledon surfaces were imaged with an SP8 confocal light microscope (Leica) using a 20× objective lens.

### Preprocessing of pavement cell images

Imported images of pavement cells were converted to grayscale and screened for necessary image cleaning steps, such as removal of artificial edges caused by image stitching, image denoising, or image rescaling. The need for the cleaning steps was evaluated in an automated manner; however, it can also be specifically selected in the GraVis GUI. Artificial edges were detected by using a Sobel filter as well as probabilistic Hough lines and were consequently removed. Noisy images were detected by calculating the percentage of white image pixels after binarization and were subjected to total variation denoising, white top hat transform, and histogram equalization.

After cleaning, the image intensity was rescaled, and a Gaussian filter and a tube filter were used for image smoothing and enhancement of tube-like structures, respectively. This was followed by image binarization, where the threshold for the binarization was selected by calculating the mean of the global threshold using Otsu’s method and the maximum of the pixel intensity histogram. The image was separated into background and pavement cell contours, which were skeletonized after removing small objects and holes. The skeleton was examined for small gaps that were closed if the gap was small enough and the skeleton endings were tilted at a similar angle. To ensure that only whole cells were used for the creation of visibility graphs, protruding branches of the skeleton were detected and removed. The final image was labeled to guarantee that each detected pavement cell had its own label, which was used to extract the corresponding cell contour. Therefore, the pixels belonging to a labeled cell were selected and the cell contour was tracked along the border of the object defined by these pixels. The tracked contour pixels were then sorted in a clockwise manner using the marching squares algorithm, starting from the right-most pixel.

The quality of our pre-processing pipeline was determined by calculating the pixel accuracy (see Supplementary Note 8, Supplementary Fig. 24, Supplementary Table 8). We found that on average 95% of the pixels were correctly classified after image segmentation.

### Creation of visibility graphs

The visibility graph was created from the shape contour by placing equidistant nodes along with it. The optimal pixel distance was calculated from the optimal number of nodes per µm and the image resolution (see below). After positioning the nodes, edges were drawn between each pair of nodes if the corresponding segments did not cross the contour. If a segment between two nodes lay on the contour, we allowed an edge to be drawn if there was no additional node on the segment. Edges were weighted according to the Euclidean distance between nodes. We quantify the (relative) graph completeness *δ* for undirected graphs by the ratio between the number of edges in the graph and the maximum possible number of edges in a graph on the same number of nodes

1 $$\delta = \frac{{2m}}{{n(n - 1)}},$$

where *m* and *n* are the numbers of all edges and nodes in the graph, respectively.

### Comparison of visibility graphs

We implemented different approaches for the comparison of graphs with equal or different numbers of nodes. The comparison approach implemented in GraVis was based on the Laplacian matrices of the graphs. A graph was transformed into its corresponding unweighted adjacency matrix. The Laplacian matrix was computed by subtracting the adjacency matrix from the diagonal matrix with node degrees on the diagonal. The eigenvalues of the Laplacian matrix were calculated and normalized to the largest eigenvalue. The Kolmogorov–Smirnov statistic was then used to calculate the maximum distance between the distribution of Laplacian eigenvalues. The resulting distance between each pair of graphs was stored in a distance matrix which can be used for clustering or for dimension–reduction techniques, like the principal component analysis. Additional approaches for the comparison of visibility graphs, such as the rotational distance, as well as the Fourier transform analysis are outlined in the Supplementary Information file. These approaches can be applied with the reduced visibility graph which was obtained by applying modified modularity clustering. The reduced visibility graph using modularity allowed the comparison of graphs that differ in their numbers of nodes (see Supplementary Information file for details).

### Detection of lobes as local features of pavement cells

The closeness centrality was calculated for each node in the graph. Lobes and necks were identified by the local minima and maxima of the closeness centrality along the contour.

The closeness centrality of a node *v* was calculated as

2 $$C\left( v \right) = \frac{{n - 1}}{{\mathop {\sum}\nolimits_{u \in V} {d(u,v)} }},$$

where *d* (*u*, *v*) is the distance, i.e., the length of the shortest path, between *u* and *v* in the visibility graph on $n = |V|$ nodes. To distinguish true lobes from tri-cellular junctions, we correlated the lobe positions with the positions of detected tri-cellular junctions, allowing for a difference of three pixels in *x*- and *y*-direction. Tri-cellular junctions were identified in the skeletonized image of the pavement cells by searching for the crossing of the skeleton. The position of lobes, necks, and tri-cellular junctions were shown along the cell contour in a visual output per pavement cell (see Supplementary Fig. 12). The cell area *A* was calculated by multiplying the image resolution with the number of pixels that are surrounded by the cell contour. The circularity *C* was calculated from the cell area and perimeter *P*^37^

3 $$C = 4\pi A/P^2.$$

The neck width was calculated as the Euclidean distance between two consecutive necks^14^. The lobe length was calculated as the length of the line intersecting the lobe position, which is orthogonal to the line connecting the two adjacent necks^14^.

### Comparison of lobe detection tools

The contours of 30 pavement cells were selected from microscopy images of Arabidopsis epidermal tissues 96 h after germination for 3 different scenarios (wild type (Col-0 background), oryzalin-treated, *clasp-1* mutant). Lobes of these 30 cells were detected manually by 20 experts in a blind study and used as a gold standard. Lobes were also detected using the tools PaCeQuant, LobeFinder, LOCO-EFA, and GraVis. For PaCeQuant and LobeFinder, the outlines were saved as Region of Interest files (.roi). For LOCO-EFA, the outlines of the selected pavement cells were converted into their corresponding x- and y-pixel positions. The number of detected lobes was provided in table format for GraVis, PaCeQuant, and LobeFinder. In contrast, we selected the number of detected lobes from LOCO-EFAs’ output as the highest modus number with a lambda that dropped by more than 0.5 compared to the lambda of the next mode. For the gold standard, GraVis, PaCeQuant, and LobeFinder, we removed detected lobes in positions of tri-cellular junctions from the analysis, thus only keeping true lobes. Furthermore, we tuned the parameters for GraVis, PaCeQuant, and LOCO-EFA as described in the Supplementary Information file and compared the performances of the tools (Supplementary Figs. 15e–h and 18).

The RMSE was calculated to quantify the difference between the number of lobes detected by the tools and the mean of the number of manually detected lobes. Furthermore, to calculate the optimal pixel distance between visibility graph nodes for GraVis, we created visibility graphs using node distances ranging from 2 to 39 pixels for the 30 pavement cells. We computed the RMSE between the detected lobes and the mean of the gold standard. To this end, we chose the pixel distance with the lowest RMSE and the image resolution to calculate 0.65 nodes/µm as the optimal number of nodes (Supplementary Fig. 17).

### Prediction of plant clades

We used the dataset from Vöfely et al.^13,32^ to predict plant clades using different properties of visibility graphs. The manually traced outlines of pavement cells were provided as coordinate files from which we selected all adaxial cells to create the corresponding visibility graphs. In total, we selected 6359 cell outlines from 5 different plant clades (see Table 2).

For each visibility graph, we calculated the number of lobes and a range of different graph centralities (Supplementary Table 4) for which we further computed the mean, median, minimum, maximum, kurtosis, and skewness from the distribution of node centralities. These were used as features of an SVM. Non-negativity of the features was ensured by subtracting the minimum value from each property. We used 80% of the data for training of a nested SVM, while the remaining 20% were used as a test set. We optimized the SVM by testing different parameter combinations using a grid search.

The multiclass SVM was built using the one-against-all strategy^53^. To account for the imbalance of class sizes, we balanced class sizes by downsampling the majority class to the size of the minority class. This process was repeated ten times for each class. For each sampling, the training data was again split into test and training subsets using fivefold cross-validation. The resulting accuracy, precision, and recall were calculated and averaged for each sampling and used to determine the best-predicted class. The final multiclass SVM model was evaluated using the test set that we put aside. After the performance evaluation of the model, we used the entire data set to retrain the SVMs.

We used the cell outlines of the selected cells to calculate the solidity and aspect ratio as shown in Vöfely et al.^13^ using the measurement tool in ImageJ and calculated the number of lobes and relative completeness of the visibility graphs using GraVis. The Kruskal–Wallis test was used to test whether there is a difference in means between the clades, while Dunn’s post hoc test was used to do a pairwise comparison.

### Requirements for GraVis

GraVis can be downloaded as an executable file for all major operating systems from Github: https://github.com/jnowak90/GraVisGUI. We implemented the described features of GraVis in a simple-to-use graphical user interface (GUI) using Python 3. The GUI can be launched by the downloaded executable files (for Windows, Linux, or MacOS). The GUI is split into two major parts: the shape description, where visibility graphs are extracted from segmented shapes, and the shape comparison, where a distance matrix of selected visibility graphs is computed. For the shape description part, the user can upload images of pavement cells which are firstly preprocessed to segment the individual cells. For each of the detected cells, visibility graphs are created and stored in a results folder. We further implemented a pipeline for the description of shapes from other domains, which require the input of binary images. For the shape comparison part, the user can select visibility graphs from a single image or multiple images to compute a distance matrix which can be used for further analysis, such as PCA or clustering. A more detailed description of the GUI functions, as well as sample images and example workflows, can be found on the Github page. The source code and data of GraVis can be accessed from Github (10.5281/zenodo.4320828).

### Reporting summary

Further information on research design is available in the Nature Research Reporting Summary linked to this article.

## Supplementary information

Supplementary Information

Peer Review File

Reporting Summary

## Data Availability

Data supporting the findings of this work are available within the paper and in the Supplementary Information files. A reporting summary for this Article is available as a Supplementary Information file. The data sets and plant materials generated and analyzed during the current study are available from the corresponding author upon request or from our Github repository [https://github.com/jnowak90/GraVisGUI]. We used publicly available datasets from the WoRMS database [http://www.marinespecies.org]^31^ and from studies of Vöfely et al.^13,32^ [10.5061/dryad.g4q6pv3]. The source data underlying Figs. 1–4, 6, and 7, as well as Supplementary Figs. 2, 3, 5–13, 15–17, 19, 20, and 22–24 are provided as a Source Data file, which is also available at the associated Github repository [10.5281/zenodo.4320828]^54^. Source data are provided with this paper.

## Source data

Source Data

## Supplementary information

**Supplementary information** The online version contains supplementary material available at 10.1038/s41467-020-20730-y.

## Code availability

The source code for the GraVis GUI is available on Github [https://github.com/jnowak90/GraVisGUI] or [10.5281/zenodo.4320828]^54^.
