# Supplementary material for: A network-based framework for shape analysis enables accurate characterization of leaf epidermal cells
Source: Nat Commun. 2021 Jan 19;12:458. doi: 10.1038/s41467-020-20730-y (PMC7815848; doi:10.1038/s41467-020-20730-y)
Supplement: Supplementary file 1 — Supplementary Information [file 41467_2020_20730_MOESM1_ESM.pdf]

Supplementary Information

A network-based framework for shape analysis enables accurate characterization of leaf epidermal cells

Nowak et al.

Supplementary Note 1: Comparison of visibility graphs with same number of nodes with rotational distance.

To compare two visibility graphs with the same number of nodes, we computed the smallest Euclidean distance between the corresponding weighted adjacency matrices upon rotation. To this end, one of the adjacency matrices is fixed and the other is circularly permuted. This is achieved by setting the last row and column of the adjacency matrix to be the first row and column in the circularly rotated matrix and decreasing the remaining respective row- and column-indices by one (Supplementary Fig. 2a). After this circular permutation, the Euclidean distance between the two matrices is calculated. The smallest distance over all circular permutations yields the distance between the two visibility graphs, and corresponds to the best overlay of the graphs.

We use the proposed rotational distance to compare the visibility graphs for a set of synthetic shapes with same number of nodes (see Table 1). The resulting distance matrix is used for hierarchical complete-linkage clustering and shows that the shapes are grouped accurately into rectangular, triangular, and circular shapes (Supplementary Fig. 2b). In addition, we apply the rotational distance to the set of synthetic shapes with different number of nodes using the node reduction method based on modularity clustering (see below). Here, the clustering of the shapes is still fairly accurate, although there seems to be a size-dependent clustering of the triangles (Supplementary Fig. 2c).

Supplementary Note 2: Comparison of visibility graphs based on a node-reducing method using modularity clustering.

While the comparison of visibility graphs using the Laplacian eigenvalues is independent on whether the number of nodes are equal or different between two graphs, the rotational distance and the Fourier transform can only be applied for comparison of graphs with the same number of nodes. Therefore, to compare two graphs of different order (i.e. number of nodes), we implemented an algorithm that reduces the number of nodes to that of the compared graphs with the smaller order. The algorithm is based on modularity clustering and includes the following steps (Supplementary Fig. 4): Given two graphs, we identify which graph has the larger number of nodes and reduce this graph to the order of the smaller graph (Supplementary Fig. 3a). We reduce the number of nodes by partitioning the larger graph into as many network clusters (intuitively, subgraphs which are more densely connected within than to the rest of the network) as there are nodes in the smaller graph. To do the partitioning we rely on the modularity, as a network cluster quality index[1]. Since optimizing modularity is an NP-hard problem, here we use greedy approximation algorithm widely used in the network research community[2], which also allows us to control the number of clusters. Initially, each node is assigned to a cluster (Supplementary Fig. 4b). In the next step, new clusters are created by assigning two consecutive nodes to the same cluster and calculating the modularity. The consecutiveness property needs to be respected, since the reduced graph should maintain the properties of the original visibility graph. Here, all possible combinations of two consecutive merged nodes are created and the combination with the maximum modularity is selected (Supplementary Fig. 4c-d). The nodes which belong to the same cluster are merged and represented by a single new node, whose position is determined by the geometric median of the merged nodes, which minimizes the sum of distances to all node positions (Supplementary Fig. 4e). The merging of nodes is repeated until the larger graph has the same number of nodes as the smaller graph (Supplementary Fig. 4f).

Supplementary Note 3: Shape comparison using the Fourier transform.

We use the Fourier transform on the set of synthetic shapes with both equal and different number of nodes, and compare the performance of the visibility graphs approach. To this end, we employ the

classical contour representation of the distances of the points on the contour from the centroid of the shape. The resulting profile is used as input to the discrete Fourier transforms. Finally, we compare the descriptors of shape resulting from the Fourier transform by using the Euclidean distance and the correlation distance. To apply the shape comparison using the Fourier transform on shapes with different number of nodes, we use the node reducing approach based on modularity clustering to create shapes with equal number of nodes. The resulting distance matrix between all pairwise shape comparisons is used for hierarchical complete-linkage clustering, as shown in Supplementary Fig. 5. Surprisingly, this representation provides a clustering of poor quality, in which rectangular shapes are clustered with triangular shapes and circular shapes are clustered with rectangular shapes (Supplementary Fig. 5b-e).

Supplementary Note 4: Homogeneity of the derived clusterings of shapes.

To compare the quality of clusters derived from the different comparison methods, we calculate the Biological Homogeneity Index (BHI), which provides a measure of homogeneity of the determined clusters[3]. Let $\mathcal{B}(i)$ and $\mathcal{B}(j)$ be functional classes containing shape i and j, respectively. We assign the indicator function $\mathrm{I}(\mathcal{B}(i)=\mathcal{B}(j))$ the value 1 if $\mathcal{B}(i)$ and $\mathcal{B}(j)$ match. Thus, for a given statistical clustering partition C_k, the BHI is defined as:

$$BHI = \frac{1}{K}\sum_{k=1}^{K}\frac{1}{n_k(n_k-1)}\sum_{i\neq j\epsilon C_k}\mathrm{I}(B(i)=B(j)), \quad (1)$$

where K is the number of clusters and n_k is the number of annotated shapes in a cluster. A value of 1 for the BHI denotes perfect clustering, in which all shapes assigned to a cluster belong to the same group (provided as a priori knowledge). Smaller values for the BHI denote larger departure from homogeneity and point at issues of the resulting clusterings. To do so, we use the dendrogram derived from the shape comparisons and cut them into three clusters by using the dendrogram distance, since we compare three different shape types. To prevent clusters with a single shape, we merged single shape clusters with the closest cluster according to the dendrogram distance. The rotational distance and the Fourier transform cannot be used to compare shapes with different number of nodes, so we used the node-reducing approach based on modularity clustering to create shapes with equal number of nodes. As shown in Supplementary Table 1, the values for the BHI for the different algorithms for comparison of visibility graphs outperform the Fourier transform approaches.

Supplementary Note 5: Visibility graph centralities to infer local features of pavement cells.

To show that the closeness centrality is the best graph property to identify lobes as local features of pavement cells, we select and compare the performance of other centrality measures. Let $u, v, t \in V$ be nodes in an undirected graph $G = (V, E)$ with $n = |V|$ vertices and $m = |E|$ edges. We define $\deg(v)$ as the degree of node v and $d(u, v)$ as the length of shortest path connecting nodes v and u. Let $\sigma(v, u)$ be the number of shortest path and $\sigma_v(u, t)$ the number of shortest paths connecting nodes u and t through node v. The adjacency matrix is denoted by $A = (a_{v,u})$ where $a_{v,u} = 1$ if an edge connects nodes v and u, and $a_{v,u} = 0$ otherwise. The effective resistance between the nodes v and u corresponds to the term $p_{uv}(v) - p_{uv}(u)$ and $\tau_{ut}(v)$ is defined as the information throughput of node v [4]. Lastly, we denote λ as the eigenvalue and $\theta_{u,t}(v)$ as the overall commodity sent from node u to t and forwarded by node v (*11*). Following this notation, the used centralities are defined as shown in Supplementary Table 4.

We use the extracted visibility graphs of the pavement cell gold standard and calculate all of the above-mentioned centralities. To detect lobes, we identify the local minima of the centralities and computed the RMSE to measure the difference to the mean of manually detected lobes. The closeness centrality

shows the lowest RMSE, closely followed by the local reaching centrality which is very similar to the closeness centrality (Supplementary Fig. 11).

Supplementary Note 6: Optimal distance between visibility graph nodes.
To find the optimal distance between visibility graph nodes, we extract the visibility graphs of the pavement cell gold standard using various pixel distances. The selected cells of the gold standard are derived from three different images, from which one image (*clasp-1*, $res_{clasp} = 0.1528 \frac{\mu m}{px}$) has a different image resolution than the other two (Col-0/oryzalin-treated, $res_{Col-0/oryzalin} = 0.221 \frac{\mu m}{px}$). Lobes are detected by identifying the local minima of the closeness centrality for each visibility graph and are afterwards compared to the mean of manually detected lobes by calculating the root mean square error (RMSE). For each pixel distance, we compute the mean RMSE and identified the minimum of the means for the cells of the two different image resolutions. The lowest mean RMSE is detected for a distance of seven pixel (Col-0/oryzalin-treated) and 10 pixel (*clasp-1*) between visibility graph nodes (see Supplementary Fig. 17). Given the image resolution, we can calculate the optimal distance, or node coverage, as:

$$node\ coverage = \frac{1}{resolution * pixel\ distance}. \quad (2)$$

Using the detected pixel distance minima and the two different image resolutions, we obtain a node coverage of $0.65 \frac{node}{\mu m}$ for both images, thus defining the optimal pixel distance that is used for the visibility graph creation of our approach.

Supplementary Note 7: Prediction of plant clades from PCs.
We create the visibility graphs of 6359 selected, adaxial pavement cells from 213 different plant species using the data set of Vöfely et al.[5]. From the created visibility graphs, we select ten graphs per species and label them according to their plant clade affiliation (eudicots, monocots, ferns, angiosperms, gymnosperms). The distance matrix of the selected graphs is computed and displayed by using PCA (Supplementary Fig. 22). Here, the ferns (blue) are easily distinguishable from the other four clades, while the centroids of eudicots (green), monocots (pink) and angiosperms (light blue) are clustered closely together.

Supplementary Note 8: Accuracy of the pre-processing and segmentation pipeline.
The quality of our segmentation pipeline is evaluated by calculating the pixel accuracy, a common validation metric for binary classification, which is calculated as:

$$P_A = \frac{TP+TN}{TP+TN+FP+FN}, \quad (3)$$

where TP = True Positive (*i.e.* pixels correctly detected as pavement cells), TN = True Negative (*i.e.* pixels correctly detected as background), FP = False Positive (*i.e.* pixels wrongly detected as pavement cells) and FN = False Negative (*i.e.* pixels wrongly detected as background).

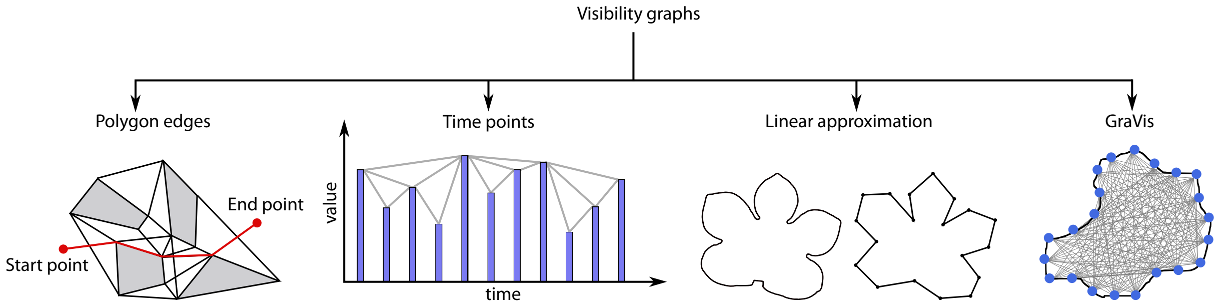

Supplementary Figure 1: Different types of visibility graphs. The visibility graph represents a set of objects along with a visibility relation between them. One type of visibility graph is formed on the vertices of multiple polygons; it is applied in robotics, where it can be used to find the shortest path among polygonal obstacles. In another type of visibility graphs the nodes correspond to the amplitude (*i.e.* value) at time points and edges are placed between nodes if they can see each other, and this type of visibility graphs has been used to deduce properties of time series. In addition, visibility graphs can be used for shape decomposition by placing nodes along a piecewise linear approximation of a shape. The here proposed approach GraVis places nodes along the shape contour and connects nodes which are visible to each other.

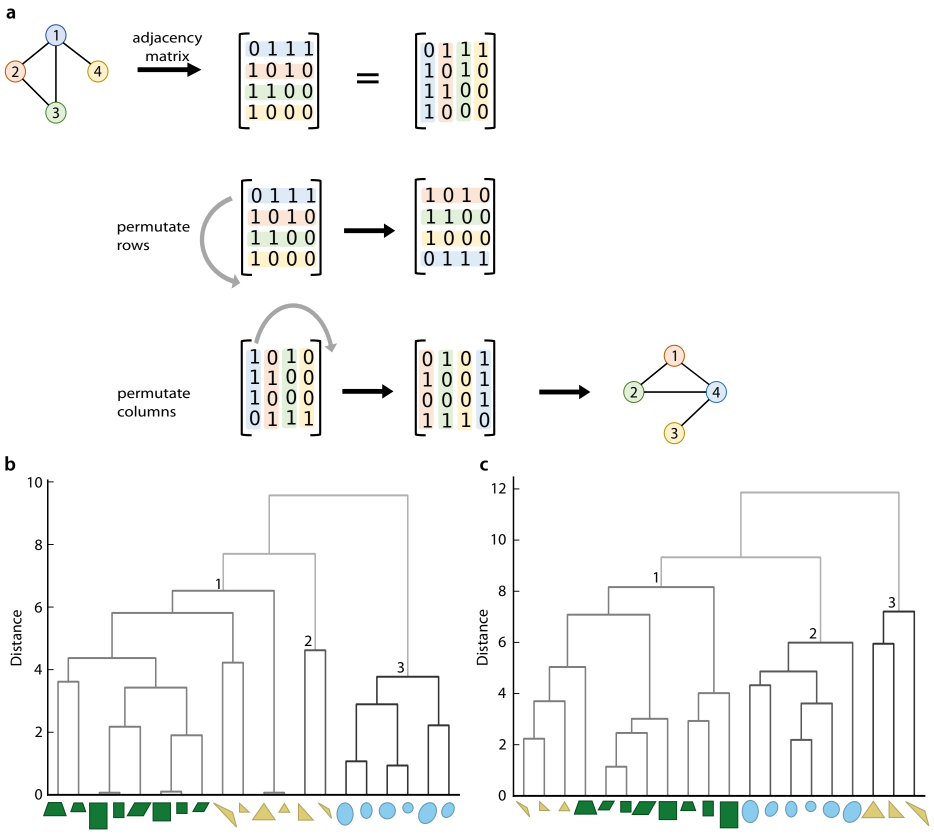

Supplementary Figure 2: Rotational permutation of a graph. (**a**) To circularly permute a graph, the corresponding adjacency matrix is used. The first row and column of the matrix are rotated to the end of the matrix, decreasing the row- and column- indices by one. (**b**) Shapes are compared by calculating the rotational distance between their corresponding visibility graphs. The resulting distance matrix is used for hiererchical complete-linkage clusterting of a set of synthetic shapes with same number of nodes, on which the rotational distance can be readily applied. (**c**) Clustering of shapes with different number of nodes that were reduced to the same size using modularity clustering. The shapes were separated into three distinct clusters, indicated by different shades of gray. The clusters are determined by using the dendrogram distance, whereby to prevent single shape clusters, we merge clusters with single shapes to the next close cluster.

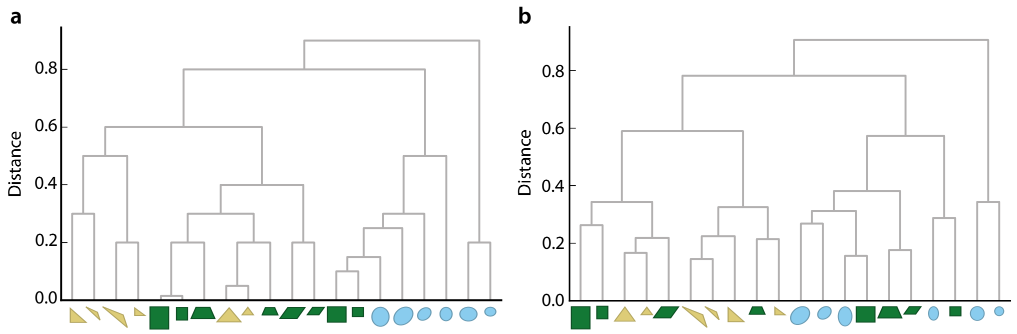

Supplementary Figure 3: Comparison of weighted visibility graphs. Two visibility graphs are compared based on their Laplacian eigenvalues from the weighted adjacency matrices. (**a**) Clustering dendrogram obtained for a set of synthetic shapes with the same number of nodes and (**b**) variable number of nodes. The poor clustering shows that the usage of the weighted adjacency matrices is not suitable for graph comparison.

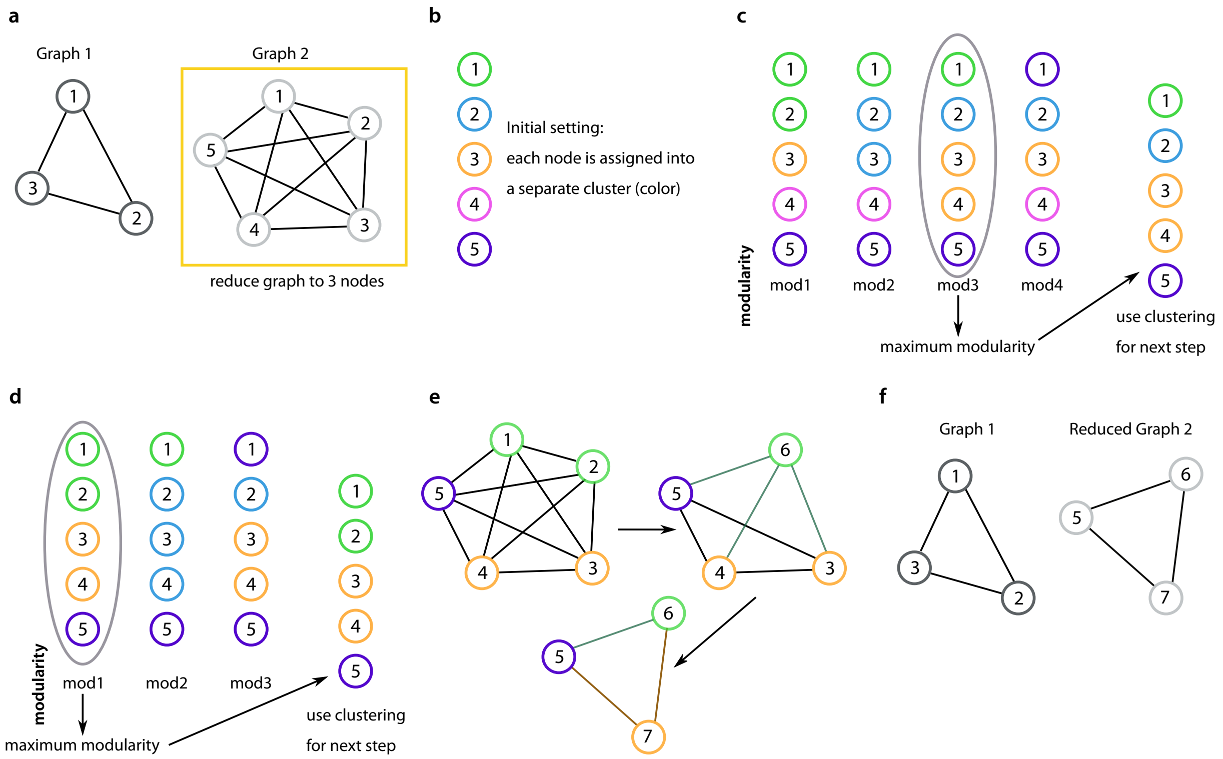

Supplementary Figure 4: Comparison of visibility graphs based on a node-reducing method using modularity clustering. (**a**) Given two graphs, the graph with the larger number of nodes is identified to be reduced to the order of the smaller graph. The number of nodes in the larger graph are reduced by partitioning the graph into as many network clusters as there are nodes in the smaller graphs, whereby we rely on modularity as a network cluster quality index. (**b**) Initially, the nodes of the larger graph are each assigned to a cluster. (**c**) New clusters are created by assigning two consecutive nodes to the same cluster. All possible combinations of two consecutive merged nodes are created. The combination with the maximum modularity is selected. (**d**)This reduction method is applied until the final number of clusters equals the order of the smaller graph. (**e**) Nodes which belong to the same cluster are represented by a single new node, which position is determined by the geometric median of the merged nodes. (**f**) The smaller graph and the reduced larger graph can be used for comparison approaches that require the same number of nodes for two graphs.

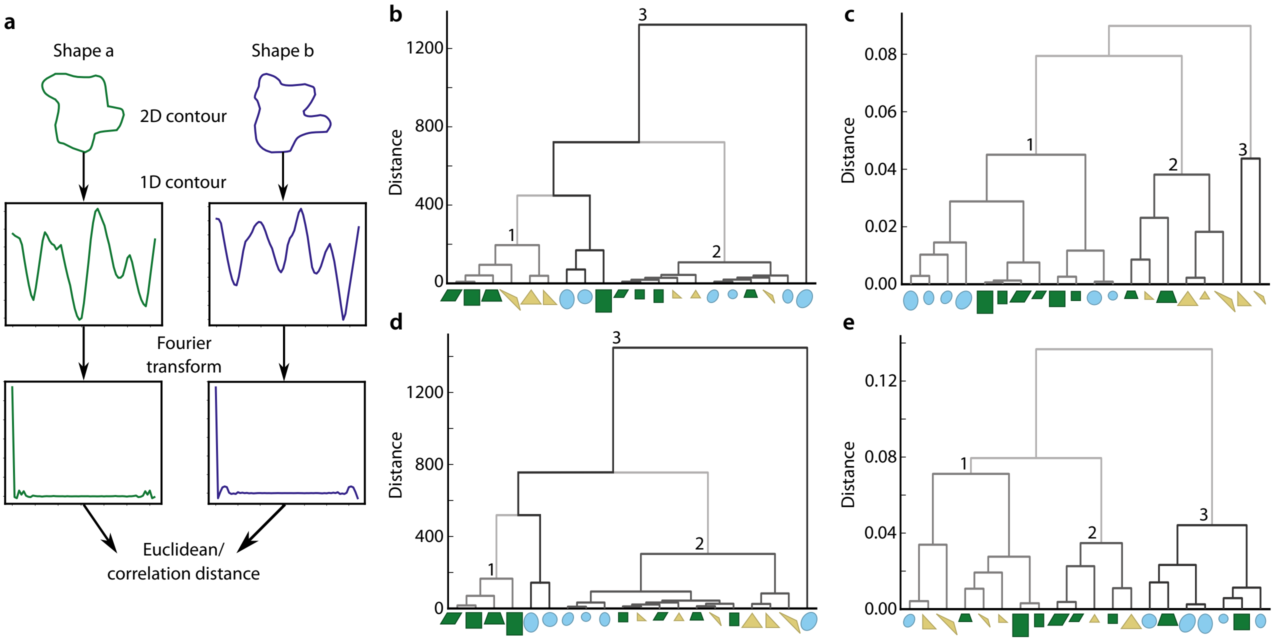

Supplementary Figure 5: Comparison of synthetic shapes using the Fourier transform. (a) Two shapes are compared based on their Fourier transforms obtained from their one-dimensional contour which is calculated by the difference of the shape centroid and its coordinates. The difference between two Fourier transforms is determined by the Euclidean distance or the correlation distance. **(b, c)** Resulting clustering dendrogram for a set of synthetic, simple triangular, rectangular and circular shapes with equal number of coordinates using **(b)** the Euclidean distance and **(c)** the correlation distance. **(d, e)** Resulting clustering dendrogram for shapes with different number of nodes, reduced with modularity clustering using **(d)** the Euclidean distance and **(e)** the correlation distance. The shapes were separated into three distinct clusters, indicated by different shades of gray. The clusters are determined by using the dendrogram distance, whereby to prevent single shape clusters, we merge clusters with single shapes to the next close cluster.

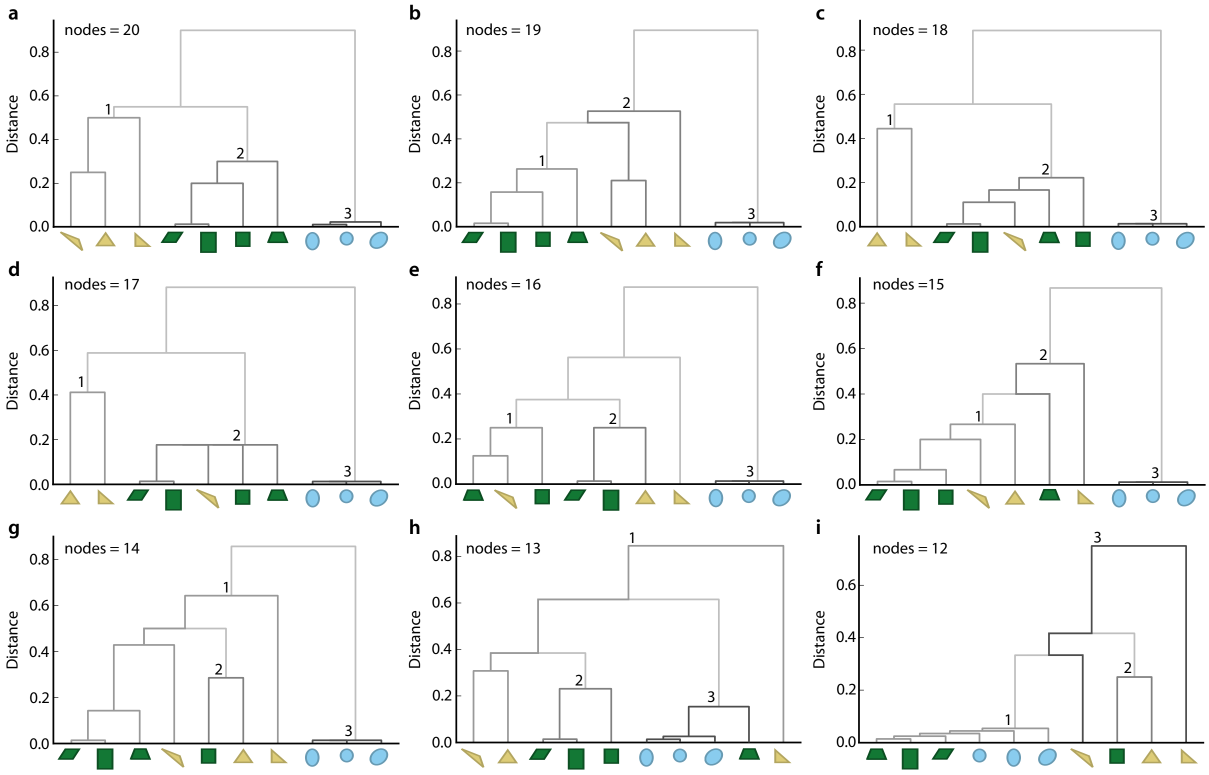

Supplementary Figure 6: Comparison of visibility graphs with different node densities. (**a-i**) The visibility graphs of a set of synthetic shapes with the same number of nodes (20) were reduced stepwise by one node and used to calculate the distance matrix to use for hierarchical clustering. To measure the quality of the clustering, the BHI was calculated for the resulting clusters (see Supplementary Fig. 7).

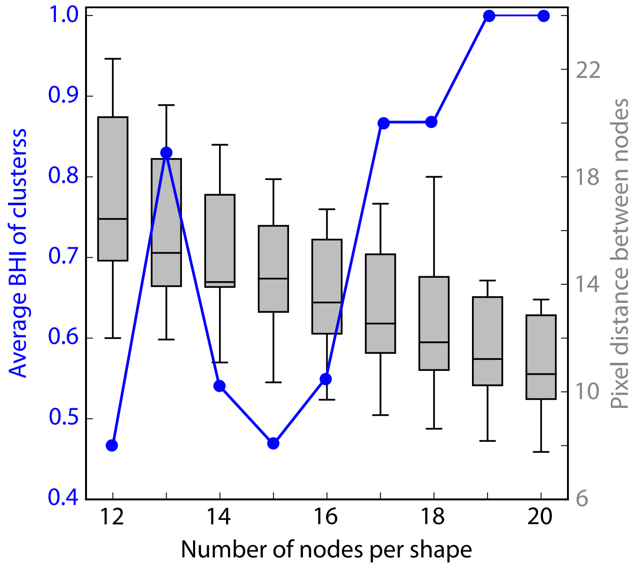

Supplementary Figure 7: Sensitivity of the number of nodes on the performance of shape comparison. Visibility graphs with equal number of nodes were compared against each other for the synthetic shapes. The number of nodes were decreased in a stepwise fashion using the node-reducing method. The distance matrix was calculated for the resulting graphs and used for hierarchical clustering whose quality we quantified with the BHI (blue). The highest average BHI was calculated with graphs that have a distance between of 10-14 pixels between nodes (gray). Boxplots are shown with median (horizontal line), 25th and 75th percentiles (box edges) and 1.5-fold of the interquartile range (whiskers).

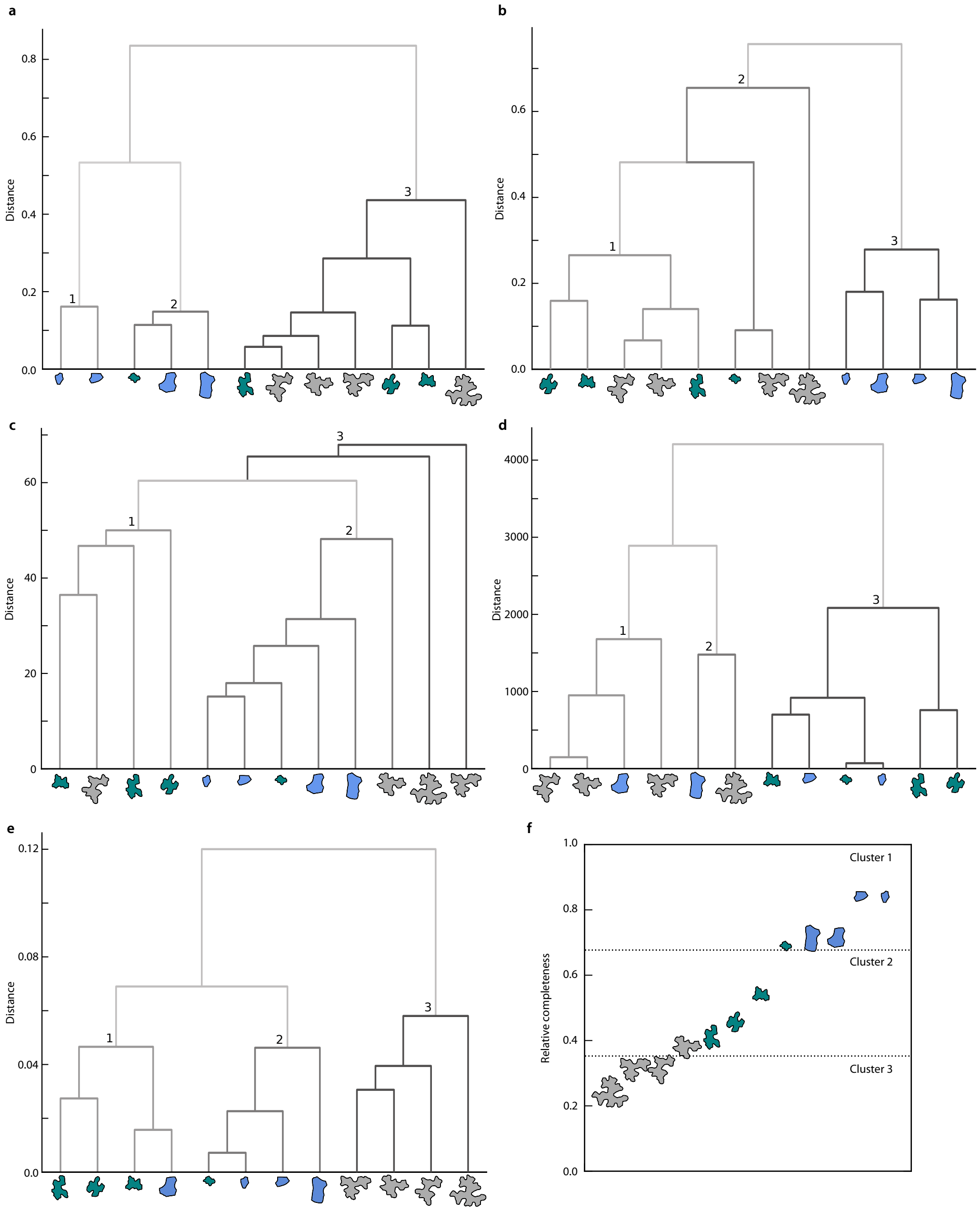

Supplementary Figure 8: Comparison of visibility graphs of pavement cells using different approaches. The visibility graphs of a set of 12 pavement cells from three different genotypes are created and used for shape comparison using (**a**) the Laplacian eigenvalues (GraVis). Additionally, we use (**b**) the Laplacian eigenvalues on the reduced set, for which we also use (**c**) the rotational distance and (**d, e**) the Fourier transform by calculating (**d**) the Euclidean distance and (**e**) the correlation distance. Pavement cells are selected from Col-0 (gray), *CA-ROP2* (blue) and *lue1* (teal). (**f**) The number of clusters is determined based on the relative completeness values of the corresponding visibility graphs to account for the shape complexity. The pavement cells are assigned to clusters based on whether the completeness values lay in the lower, middle or top third (different shades of gray).

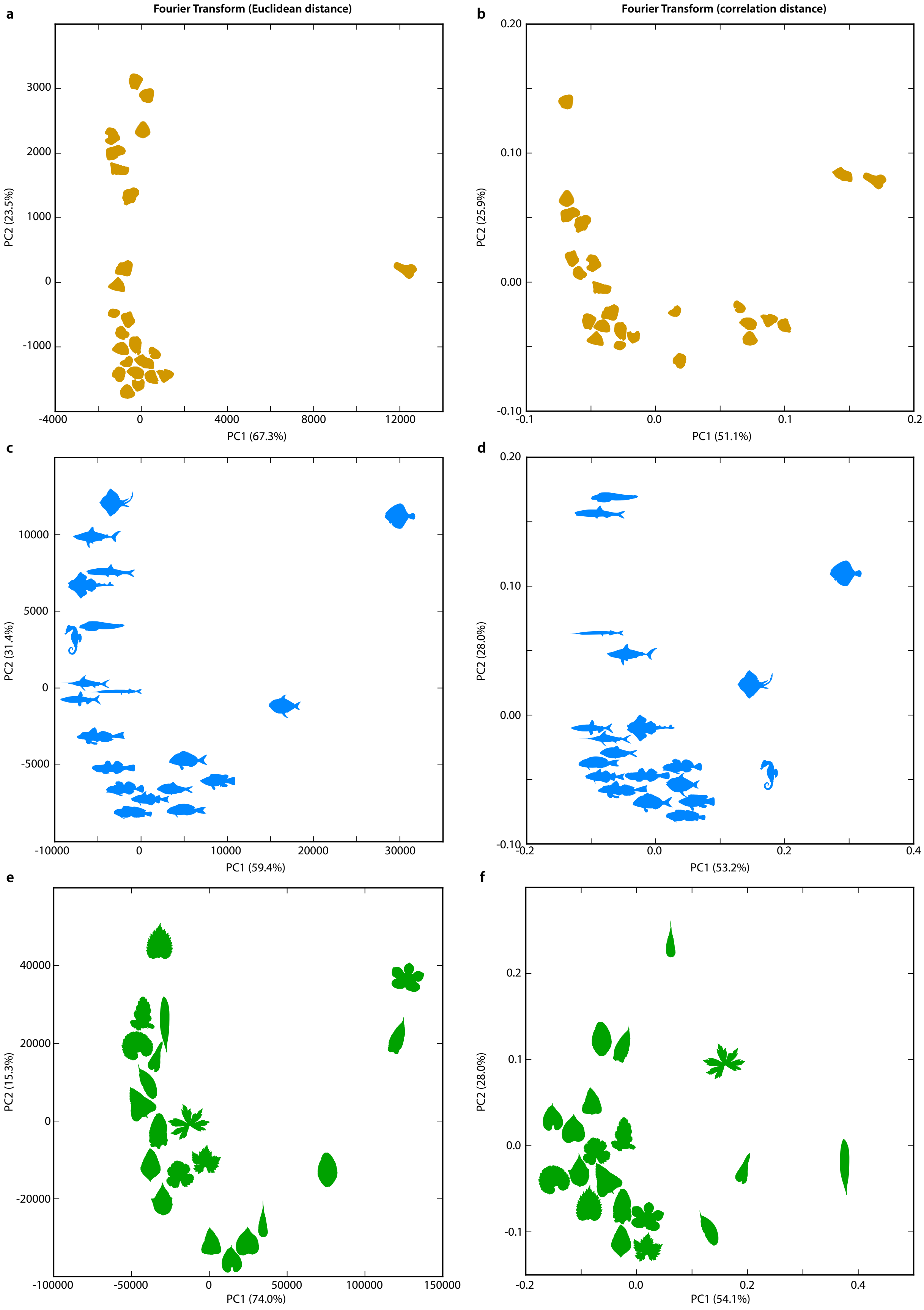
a
Fourier Transform (Euclidean distance)
PC2 (23.5%)
PC1 (67.3%)
3000
2000
1000
0
-1000
-4000
0
4000
8000
12000
b
Fourier Transform (correlation distance)
PC2 (25.9%)
PC1 (51.1%)
0.20
0.10
0.00
-0.10
-0.1
0.0
0.1
0.2
c
PC2 (31.4%)
PC1 (59.4%)
10000
5000
0
-5000
-10000
0
10000
20000
30000
d
PC2 (28.0%)
PC1 (53.2%)
0.20
0.10
0.00
-0.10
-0.2
0.0
0.2
0.4
e
PC2 (15.3%)
PC1 (74.0%)
40000
20000
0
-20000
-100000
-50000
0
50000
100000
150000
f
PC2 (28.0%)
PC1 (54.1%)
0.2
0.1
0.0
-0.1
-0.2
0.0
0.2
0.4

Supplementary Figure 9: Fourier transform approach for analysis of shapes from different domains. The distances between visibility graphs is calculated based on the Fourier transform using (**a, c, e**) the Euclidean distance or (**b, d, f**) the correlation distance. The principal component analysis of shapes from three different domains are shown: (**a, b**) sand grains, (**c, d**) fish shapes and (**e, f**) leaf shapes.

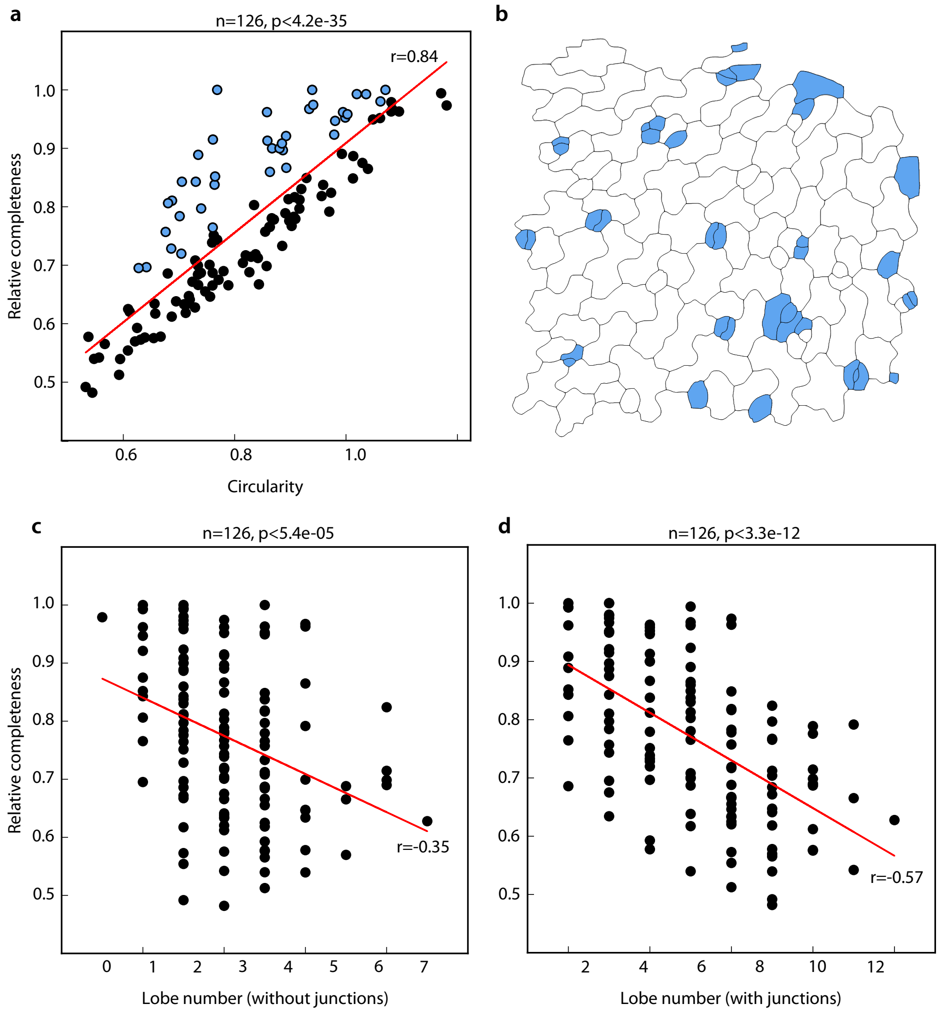

Supplementary Figure 10: Correlations between the relative completeness and other shape metrics. The visibility graphs for the cells in Fig. 4 are used to determine the Pearson correlation coefficient between their relative completeness and (**a**) the circularity, whereby (**b**) depicts the cells with a low circularity and a high relative completeness (blue circles in (**a**)). Further Pearson correlation coefficients are determined between the relative completeness and (**c**) the number of lobes without junctions, and (**d**) the number of lobes with junctions. The number of cells, correlation value, and the associated significance (independent two-sample t-test, two-sided p-value) are included in the respective figure panels.

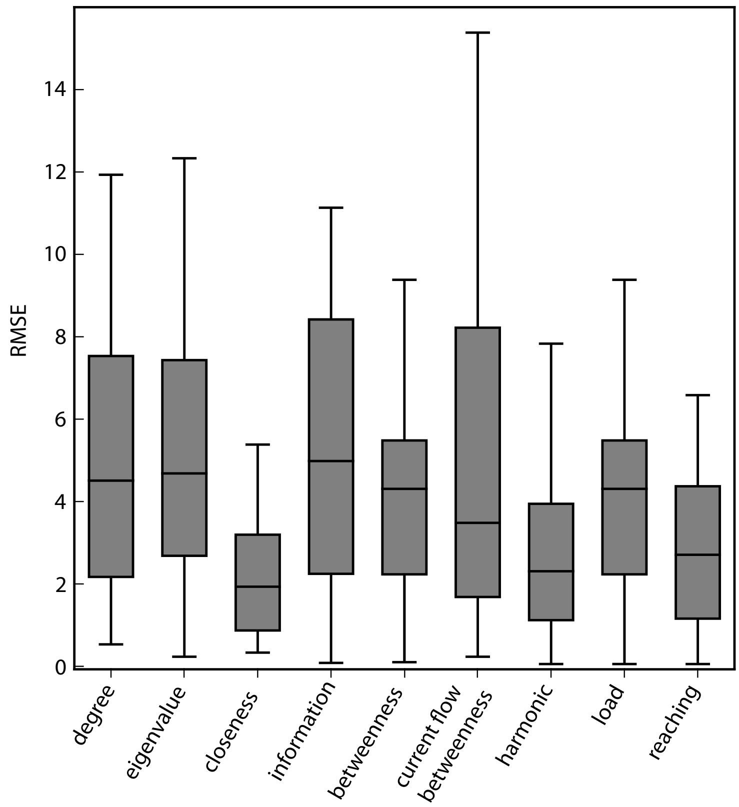

Supplementary Figure 11: RMSE of detected pavement cell lobes for different centralities. The RMSE is calculated from the number of lobes detected using different centralities of the visibility graphs (n=30 independent cells of the gold standard) and the mean of manually annotated nodes. Boxplots are shown with median (horizontal line), 25th and 75th percentiles (box edges) and 1.5-fold of the interquartile range (whiskers).

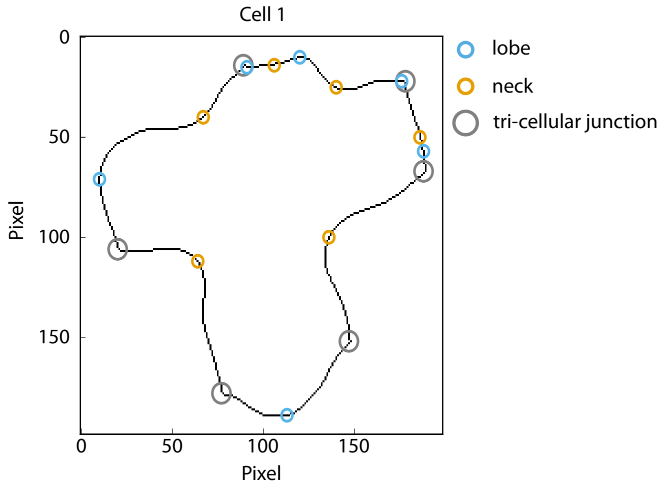

Supplementary Figure 12: Visual output for GraVis depicting positions of lobes, necks and tri-cellular junctions. For each cell, the positions of local shape features are shown by circles. Tri-cellular junctions are shown in gray, necks in orange and lobes in blue. Overlapping gray and blue circles indicate positions where a junction was detected as a lobe.

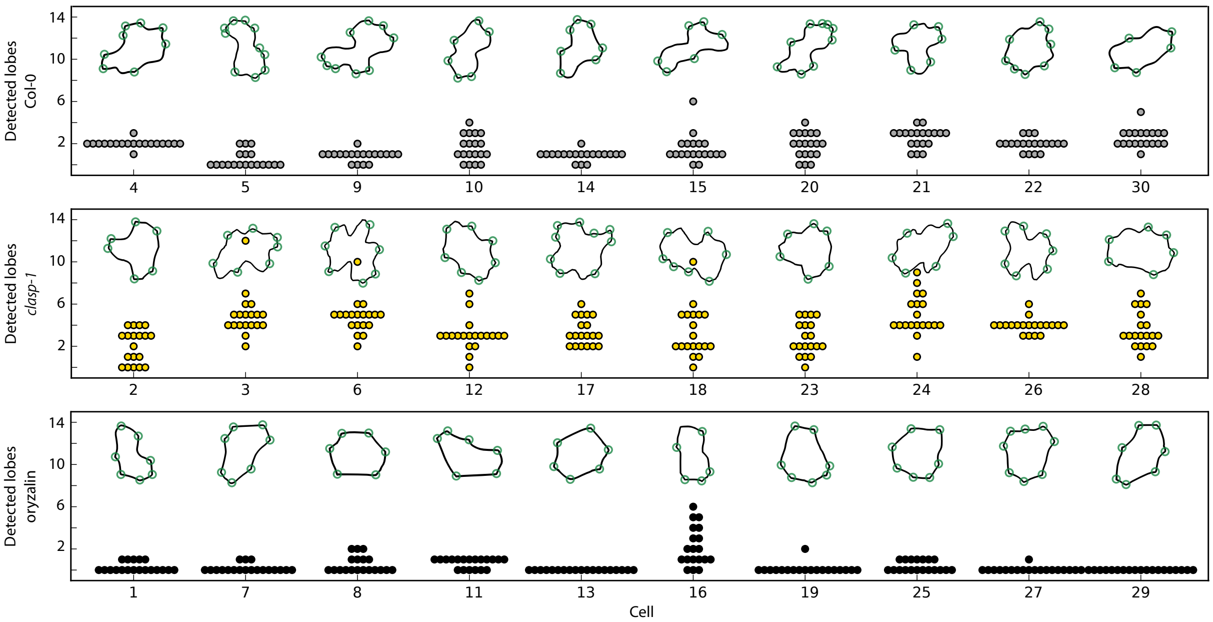

Supplementary Figure 13: Bee swarms of manually detected lobes by 20 experts. The number of detected lobes (without tri-cellular junctions) for the 30 cells of the gold standard that were selected from three different conditions (Col-0: gray, *clasp-1*: yellow, oryzalin: black) are shown as bee swarms. The position of tri-cellular junctions is indicated by green circles.

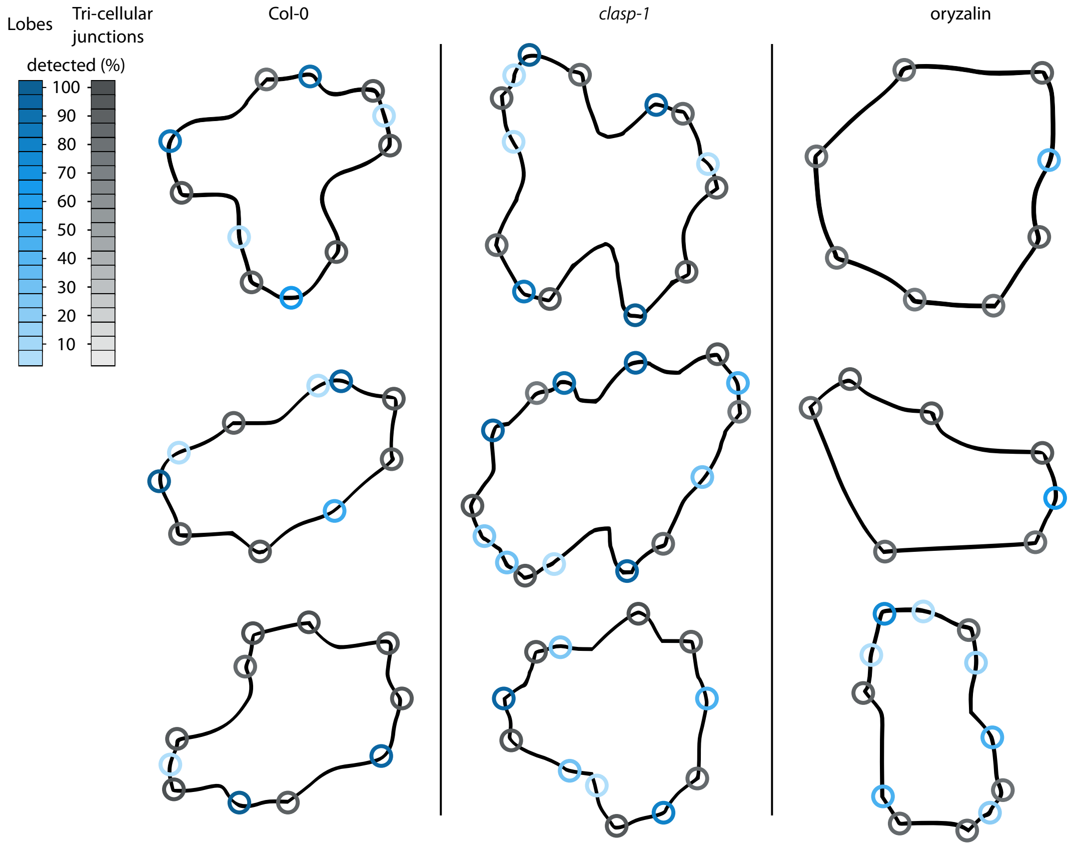

Supplementary Figure 14: Consensus of manually detected lobes by 20 experts. Three cells for each condition are selected from the gold standard to visualize the consensus among the experts regarding the positions of lobes (blue) and tri-cellular junctions (gray). The consensus of experts is indicated by the hue of the colored circles. Light circles indicate a low percentage of consensus (true lobes: light blue, tri-cellular junctions: light gray), while

dark circles indicate a high percentage of consensus among the experts (true lobe: dark blue, tri-cellular junctions: dark gray).

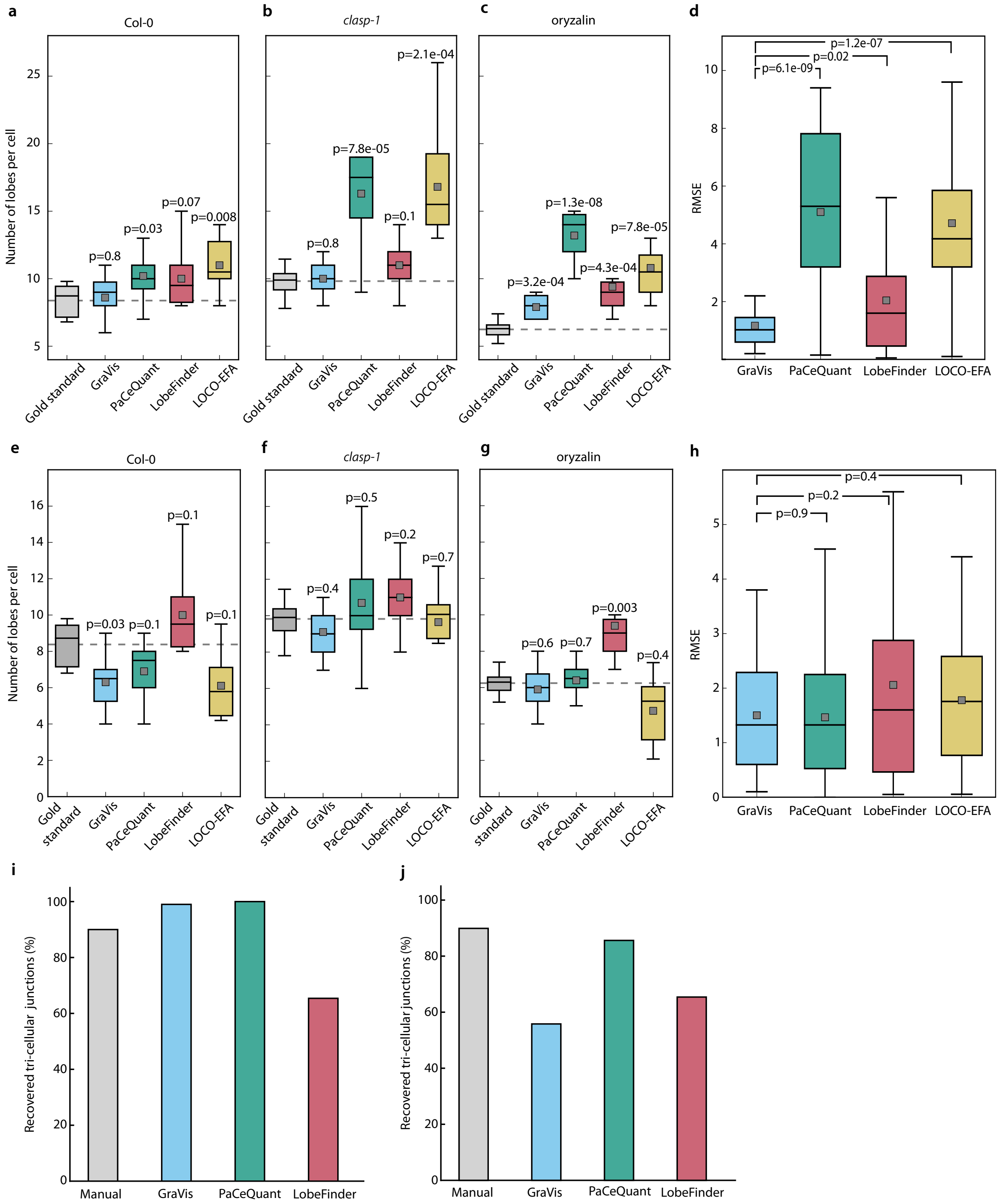

Supplementary Figure 15: Comparison of visibility graph with other contending approaches to count the number of lobes including tri-cellular junctions in leaf pavement cells. The quantitative comparison is based on a gold standard that includes n=30 independent Arabidopsis pavement cells obtained from each of the three scenarios: (**a, e**) wild type (n=10 cells), (**b, f**) *clasp-1* mutant (n=10 cells), and (**c, g**) oryzalin treatment (n=10 cells), all with lobes manually annotated by 20 experts. The dotted line denotes the mean of the gold standard, *i.e.* manual detection by 20 experts, for the respective scenario. Shown is the comparison using (**a-d**) default parameters and (**e-h**) tuned parameters to detect the number of lobes including tri-cellular junctions. The dotted line denotes the mean of the gold standard, i.e., manual detection by 20 experts, for the respective scenario. (d) GraVis performs as good as LobeFinder based on the residual mean square error (RMSE) using default parameters, while GraVis performs as good as the other contenders using (h) tuned parameters. The percentage of recovered tri-cellular junctions for the different tools is shown for (i) default and (j) tuned parameters. P-values were determined with a two-sided two sample t-test and were Benjamini-Hochberg adjusted. Boxplots are shown with

median (horizontal line), mean (gray square), 25th and 75th percentiles (box edges) and 1.5-fold of the interquartile range (whiskers). (**e**) Percentage of recovered tri-cellular junctions for tools which provide positional information of detected lobes (all tools except LOCO-EFA).

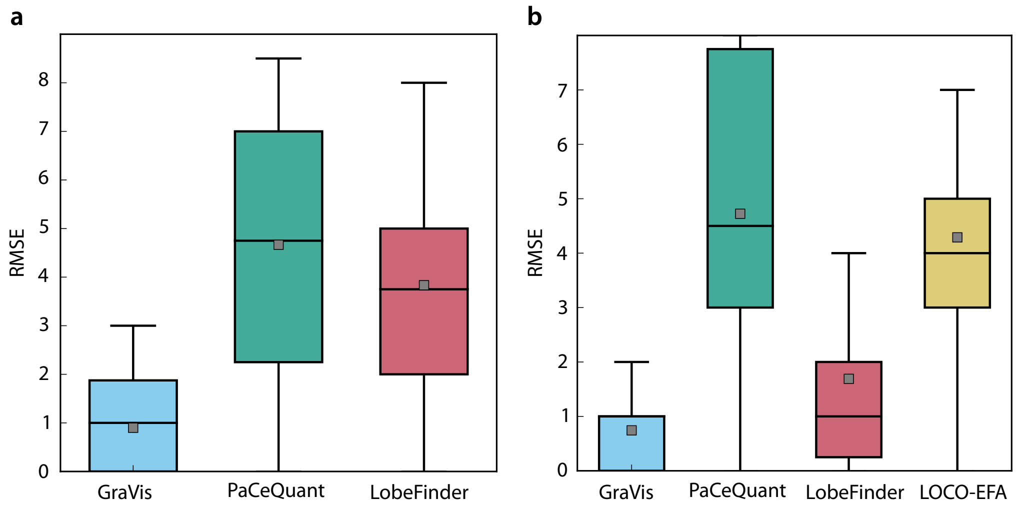

Supplementary Figure 16: Comparison of the performance of lobe detection of different tools. The performance of different lobe detection tools is compared to the median of a gold standard that includes 30 Arabidopsis pavement cells obtained from three scenarios (wild type, *clasp-1* mutant, oryzalin treatment). GraVis outperforms all three contenders based on the residual mean square error (RMSE) for both (**a**) lobes excluding tri-cellular junctions and (**b**) lobes including tri-cellular junctions. Boxplots are shown with median (horizontal line), mean (gray square), 25th and 75th percentiles (box edges) and 1.5-fold of the interquartile range (whiskers).

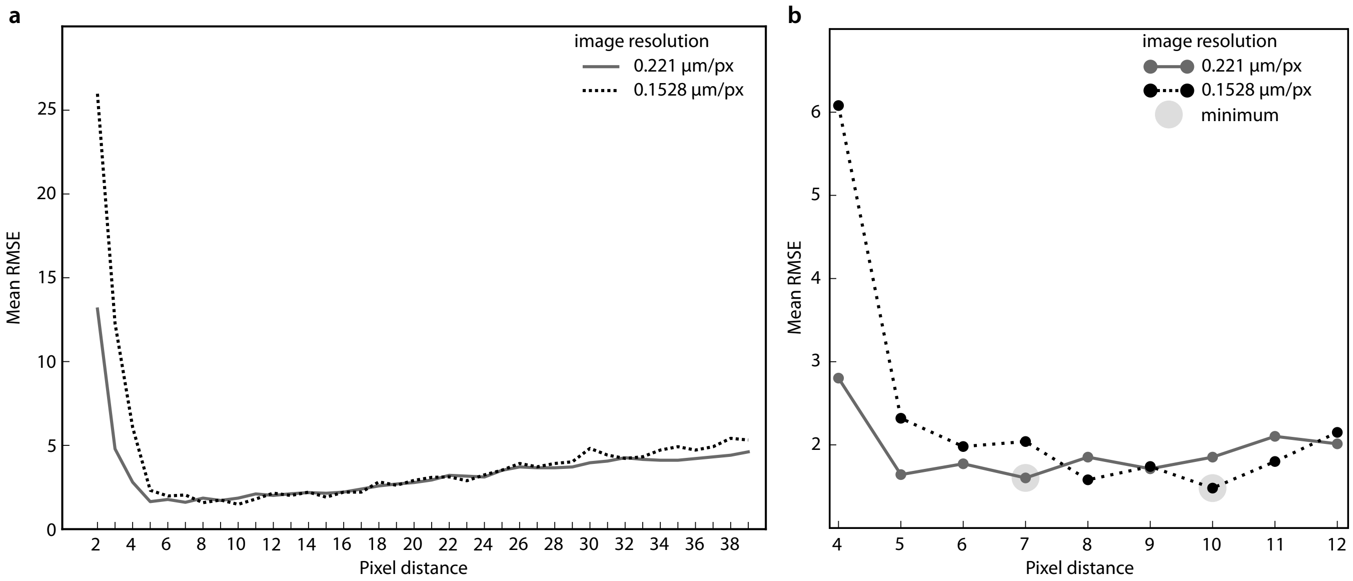

Supplementary Figure 17: Determining the optimal pixel distance for visibility graph node placement. (**a**)The visibility graphs of the 30 cells from the gold standard are created using different pixel distances between nodes. The mean RMSE is computed for each pixel distance ranging from 2 to 39 pixels. (**b**) The minimum of all mean RMSE is then detected for cells from two different image resolutions.

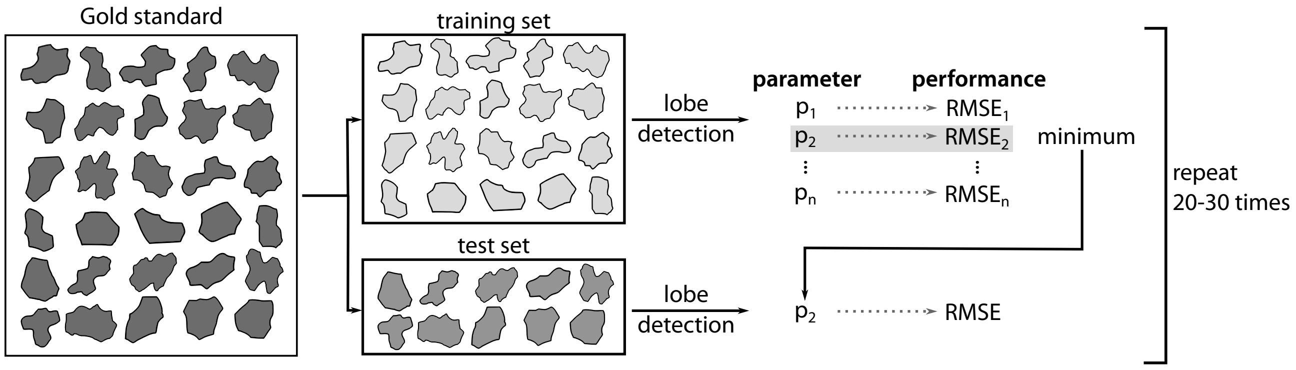

Supplementary Figure 18: Parameter tuning of lobe detection tools based on a gold standard. To find the optimal parameters for the lobe detection tools GraVis, PaCeQuant and LOCO-EFA, the cells of the gold standard are randomly split into a training and test set. Lobes are detected for the training set by using different parameters or parameter combinations. The performance of the thus resulting detected lobes is compared by computing the root mean square error (RMSE) based on the manually detected lobes by 20 experts. The parameter or parameter combination resulting in the lowest RMSE is then used for the lobe detection of the test set, where the performance is evaluated by calculating the RMSE. This procedure is repeated 20 to 30 times and the parameter setting with the overall lowest RMSE is chosen for the comparison of lobe detection tools after tuning (see Supplementary Fig. 19). For GraVis, the node distance was tuned, while for LOCO-EFA the threshold between consecutive modes are tuned to determine the number of detected lobes. In PaCeQuant, all combinations of the three different parameters (Gaussian σ in curvature analysis, minimal length of a protrusion section and the minimal length of an indentation section) were tuned.

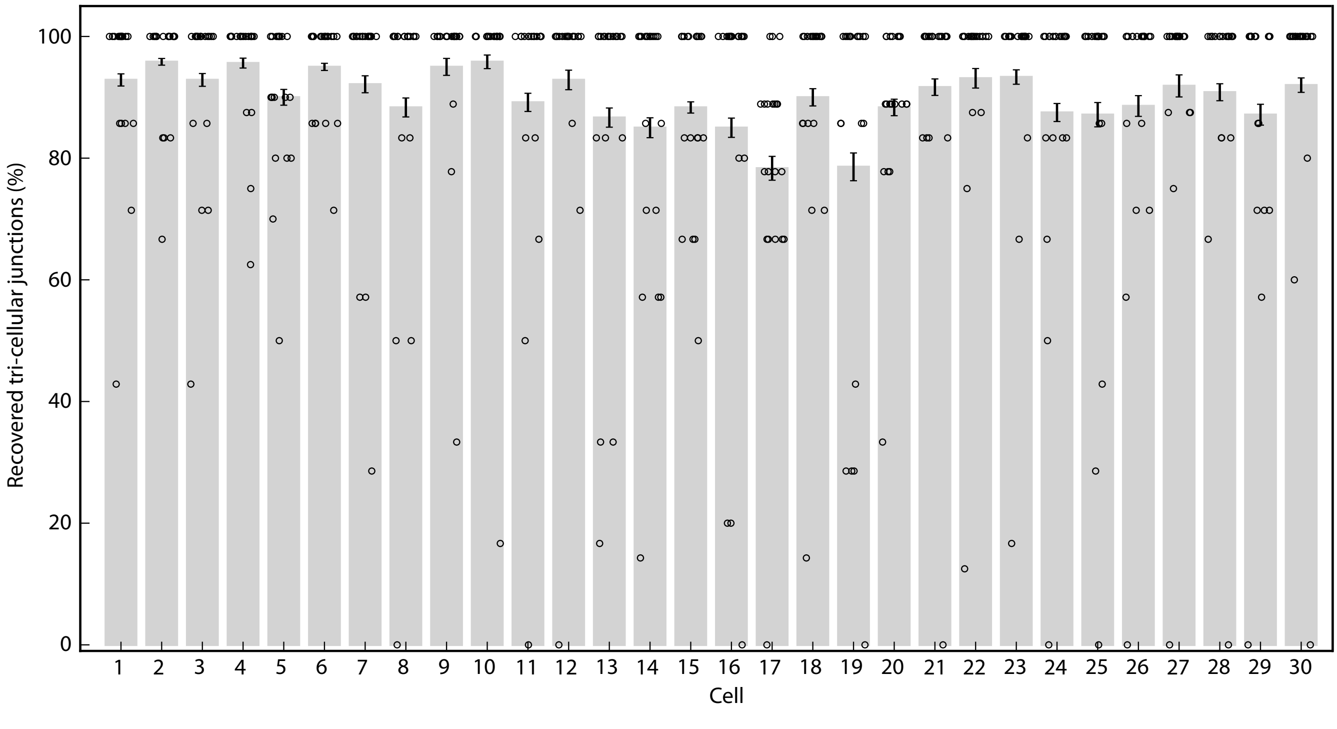

Supplementary Figure 19: Recovery of tri-cellular junctions for each cell of the gold standard. The percentage and variance of manually detected tri-cellular junctions for each cell of the gold standard is shown. The n=20 independent experts were provided only with the pavement cell contours for the manual detection and did not have any information about cell wall segments with neighboring cells, thus leading to variances in the detection of all tri-cellular junctions by the experts. Data are presented as mean values +/- standard deviation.

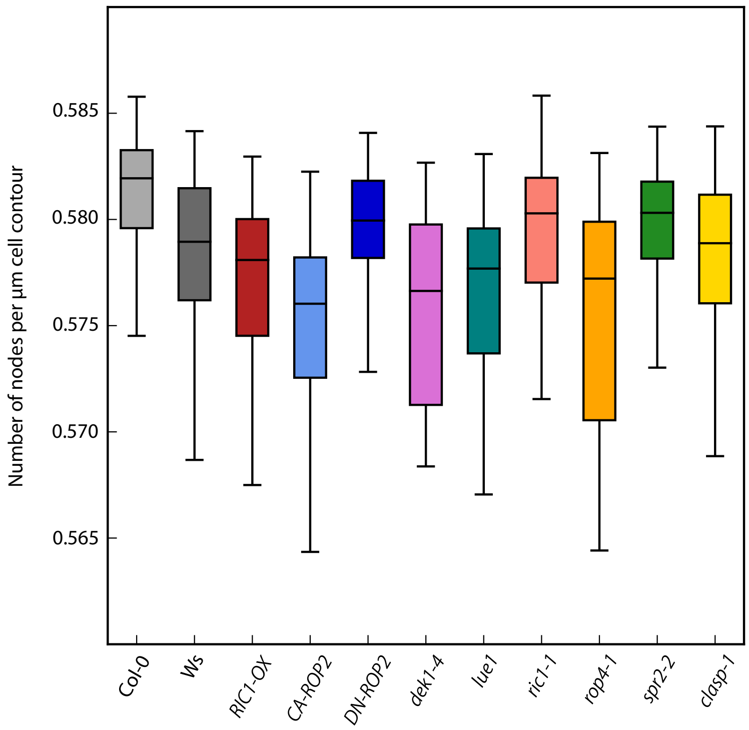

Supplementary Figure 20: Number of nodes among pavement cells of different genetic lines. The set of 80 pavement cells from different genetic lines are used to display the number of nodes of the corresponding visibility graphs. To account for different sizes of pavement cells, the number of nodes is shown in terms of the length of the extracted cell contour (in µm). Boxplots are shown with median (horizontal line), 25th and 75th percentiles (box edges) and 1.5-fold of the interquartile range (whiskers).

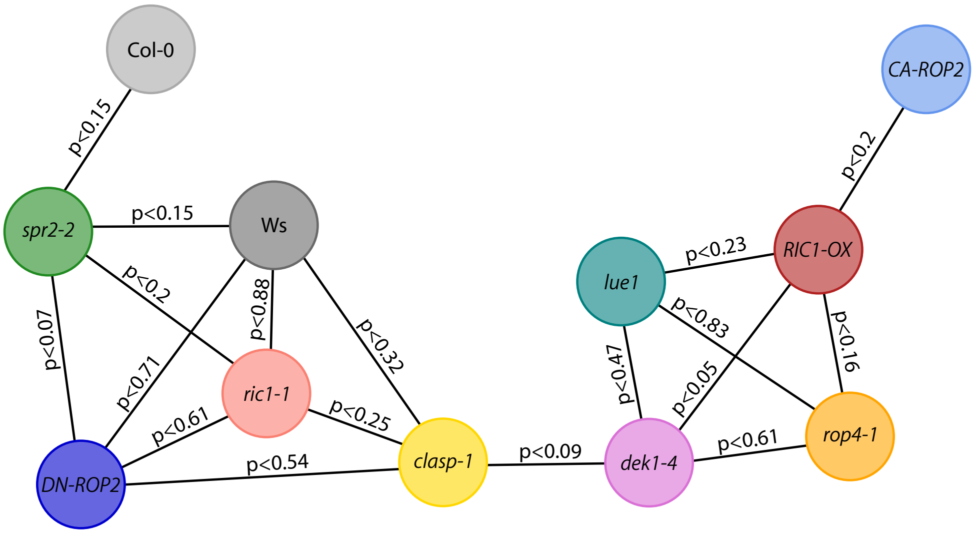

Supplementary Figure 21: Clustering graph of different genetically modified lines. The number of detected lobes for each genotype is used to do test the difference between pairwise means (Supplementary Table 6, two-sided Dunn's post-hoc test, Benjamini-Hochberg corrected). Genotypes that show no significant difference in means are connected by an edge ($p > 0.05$), ultimately displaying clusters of genotypes with similar phenotypes.

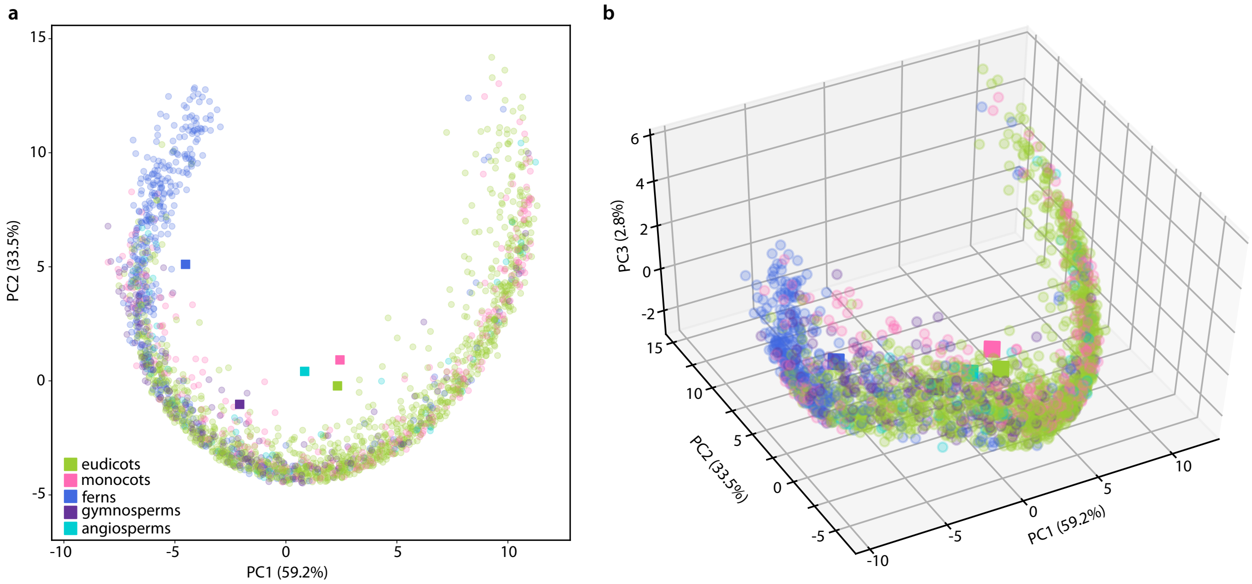

Supplementary Figure 22: PCA of visibility graphs from different plant clades. The distance matrix of visibility graphs from manually segmented pavement cells of 213 different plant species is calculated[5]. The five major clades of the plant species are shown in a (**a**) 2D and (**b**) 3D PCA plot with their corresponding centroid: eudicots (green), monocots (pink), ferns (blue), gymnosperms (purple) and angiosperms (light blue).

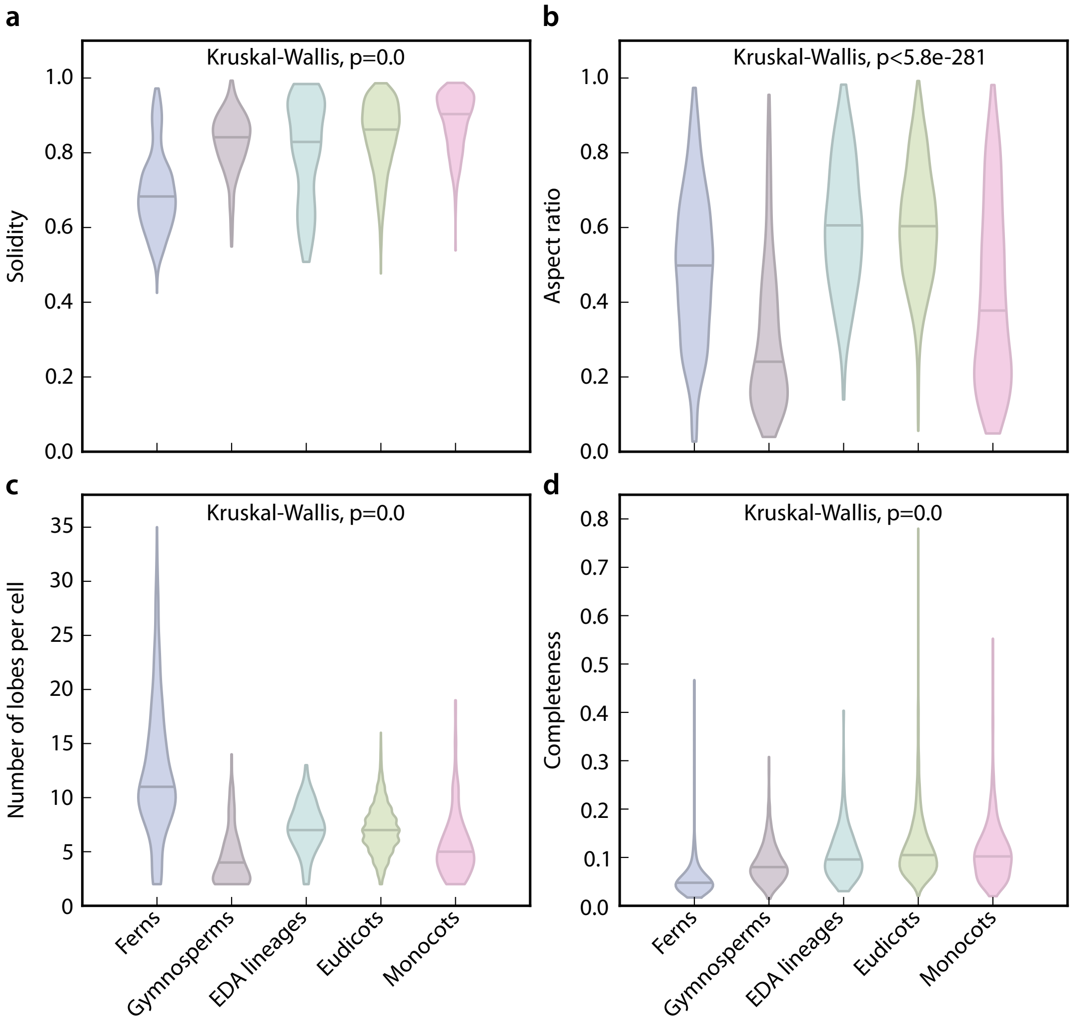

Supplementary Figure 23: Violin plots of selected shape features for pavement cells of different plant clades. The selected cells of 213 different plant species are used to display the distribution of the (**a**) solidity, (**b**) the aspect ratio, (**c**) the number of lobes per cell and (**d**) the relative completeness. The five major clades of the plant species are shown with their corresponding centroid: eudicots (green), monocots (pink), ferns (blue), gymnosperms (purple) and angiosperms (light blue). The lines in the violin plots indicate the median of the data. The one-sided Kruskal-Wallis test was used to test whether there is a difference in means between the clades for the shown shape features.

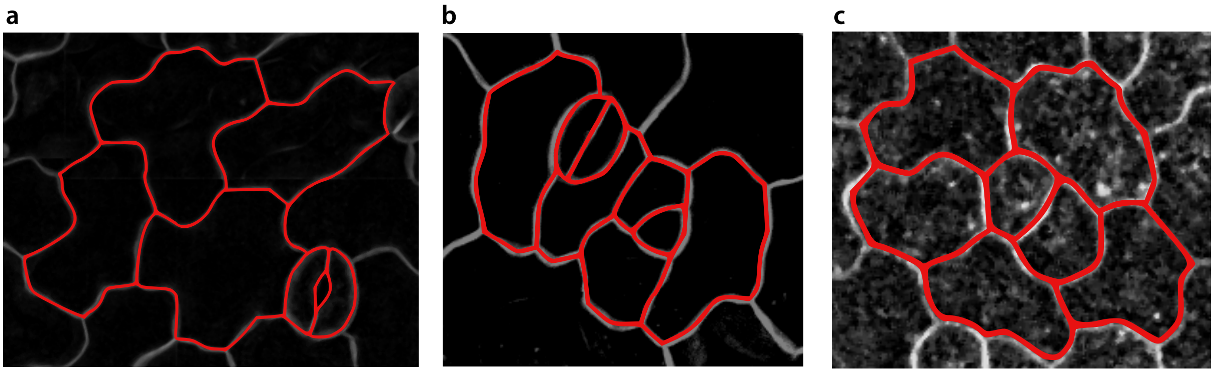

Supplementary Figure 24: Selected images for the calculation of the pixel accuracy after image segmentation. (**a-c**) Three image sections of epidermal pavement cells are selected for manual segmentation and pre-processing using GraVis. Whole cells are manually segmented from the image sections (red), as GraVis removes cut cells during the pre-processing.

Supplementary Table 1: Quality of clusters derived from the comparison of a synthetic set of shapes using different shape comparison methods.

Method	Number of Nodes	BHI			
		Cluster 1	Cluster 2	Cluster 3	Mean
Laplacian	Equal	1.0	1.0	1.0	1.0
	Different	1.0	0.45	1.0	0.82
	Different (reduced)	0.47	1.0	1.0	0.82
Rotational	Equal	0.52	1.0	1.0	0.84
	Different (reduced)	0.56	1.0	1.0	0.85
FT (Euclidean distance)	Equal	0.33	0.29	0.27	0.3
	Different (reduced)	1.0	0.6	0.31	0.64
FT (correlation distance)	Equal	0.45	0.47	1.0	0.64
	Different (reduced)	0.32	0.4	0.52	0.42

The Biological Homogeneity Index (BHI) is calculated using the clusters shown in Supplementary Fig. 2 and 5. The BHI show that the Laplacian eigenvalues, as implemented in GraVis, cluster the synthetic shapes perfectly if all shapes have the same number of nodes. Nevertheless, the Laplacian eigenvalues also perform very well for the synthetic shapes with different number of nodes and the reduced set of nodes, similar to the rotational distance. In contrast, the Fourier transform using either the Euclidean or correlation distance performs worst.

Supplementary Table 2: Quality of clusters derived from the comparison of 12 pavement cells using different shape comparison methods.

Method	BHI			
	Cluster 1	Cluster 2	Cluster 3	Mean
Laplacian	1.0	1.0	0.43	0.81
Laplacian (reduced graph)	0.6	0.33	1.0	0.64
Rotational	0.5	0.67	1.0	0.50
FT (Euclidean distance)	0.17	0.0	0.4	0.19
FT (correlation distance)	0.5	0.5	1.0	0.67

The Biological Homogeneity Index (BHI) is calculated using the clusters shown in Supplementary Fig. 8f. The BHI values show that the Laplacian approach used by GraVis is best to cluster the shapes of pavement cells into distinct groups, followed by the Fourier transform approach using the correlation distance.

Supplementary Table 3: Details of visibility graphs for different domains.

Sand grains		Fish		Leaves	
n	m	n	m	n	m
15	100	211	7871	304	20168
30	405	186	8307	231	18470
41	649	253	7238	169	10087
25	287	172	6078	83	2983
20	186	178	7668	297	10970
42	766	184	10902	124	5581
39	683	118	2732	95	3536
28	376	100	3342	105	3118
30	434	160	6960	106	3692
14	88	197	6686	172	6594
34	512	120	2836	52	363
43	706	103	2720	199	9615
29	377	146	4810	144	8887
74	2032	171	4412	113	4247
41	780	184	7592	147	8381
39	735	160	5582	81	2308
36	561	184	8354	97	4006
33	499	169	5510	125	5860
27	305	150	3992	207	12323
30	388	159	7062	175	13400
25	275				
18	146				
19	150				
21	206				

We select shapes from three different domains (sand grains, fish, leaves) and create the corresponding visibility graphs. For each graph, the number of edges, $|E| = m$ and nodes, $|V| = n$ are provided.

Supplementary Table 4: Definitions of graph centralities used for analysis of local shape features of pavement cells.

Centrality measure	Definition	Reference
Degree	$C_D(v) = \frac{\deg(v)}{n-1}$	Freeman, 1979[6]
Eigenvector	$C_E(v) = \frac{1}{\lambda}\sum_{u \epsilon V} a_{v,u} C_E(u)$	Bonacich, 1987[7]
Closeness	$C_C(v) = \frac{n-1}{\sum_{u \in V} d(u,v)}$	Sabidussi, 1966[8]
Information	$C_I(v) = \frac{n-1}{\sum_{u \in V} p_{uv}(v) - p_{uv}(u)}$	Stephenson & Zelen, 1989[9]

Betweenness	$C_B(v) = \frac{2}{(n-1)(n-2)} \sum_{s,t \in V} \frac{\sigma_v(s,t)}{\sigma(s,t)}$	Freeman, 1977[10]
Current flow betweenness	$C_{CFB}(v) = \frac{2}{(n-1)(n-2)} \sum_{s,t \in V} \tau_{st}(v)$	Newman, 2005[11]
Load	$C_L(v) = \frac{2}{(n-1)(n-2)} \sum_{s,t \in V} \theta_{s,t}(v)$	Goh, 2001[12]
Harmonic	$C_H(v) = \frac{\sum_{u \in V} \frac{1}{d(u,v)}}{n-1}$	Marchiori & Latora, 2000[13]
Local reaching	$C_R(v) = \frac{1}{n-1} \sum_{u \in V} \frac{1}{d(u,v)}$	Mones, 2012[14]

Each listed centrality measure was used to identify lobes of the 30 manually annotated graphs of the gold standard.

Supplementary Table 5: Comparison of the performance of lobe detection of different tools.

Tool	Without tri-cellular junctions					
	RMSE using GS mean (default)		RMSE using GS median		RMSE using GS mean (tuned)	
	mean	var	mean	var	mean	var
GraVis	0.87	0.70	0.90	0.85	1.76	1.89
PaCeQuant	4.50	7.74	4.67	8.26	1.05	0.71
LobeFinder	3.73	4.09	3.83	4.56	3.73	4.09
p-value	10^{-9}	10^{-8}	10^{-8}	10^{-7}	10^{-8}	10^{-5}
	With tri-cellular junctions					
	RMSE using GS mean (default)		RMSE using GS median		RMSE using GS mean (tuned)	
	mean	var	mean	var	mean	var
GraVis	1.19	0.79	1.17	0.78	1.50	1.09
PaCeQuant	5.11	9.16	5.09	9.15	1.47	1.20
LobeFinder	2.06	3.30	2.05	3.27	2.06	3.30
LOCO-EFA	4.72	9.57	4.71	9.57	1.76	1.30
p-value	10^{-8}	10^{-9}	10^{-9}	10^{-10}	0.69	0.005

The performance of different lobe detection tools is compared by computing the root mean square error (RMSE) based on the manually detected lobes by 20 experts. The comparison is done with and without including tri-cellular junctions. Furthermore, the RMSE is calculated based on either the mean of manually detected lobes using default or tuned parameters, or the median of manually detected lobes using default parameters. The difference in means between the tools is tested using the one-sided Kruskal-Wallis test, while the difference in variance between the tools is tested using the one-sided Bartlett's test.

Supplementary Table 6: Adjusted p-values of the pairwise differences for each pair of genetically modified lines.

	Col-0	*DN-ROP2*	*RIC1-OX*	Ws	*clasp-1*	*dek1-4*	*lue1*	*ric1-1*	*rop4-1*	*spr2-2*
CA-ROP2	10^{-20}	10^{-9}	0.2	10^{-10}	10^{-7}	10^{-3}	0.01	10^{-10}	10^{-3}	10^{-14}
Col-0		10^{-4}	10^{-15}	10^{-3}	10^{-5}	10^{-9}	10^{-11}	10^{-3}	10^{-10}	0.15
DN-ROP2			10^{-5}	0.71	0.54	0.02	10^{-3}	0.61	10^{-3}	0.07
RIC1-OX				10^{-6}	10^{-4}	0.05	0.23	10^{-7}	0.16	10^{-10}
Ws					0.32	10^{-3}	10^{-4}	0.88	10^{-4}	0.15
clasp-1						0.09	0.01	0.25	0.02	0.01
dek1-4							0.47	10^{-3}	0.61	10^{-5}
lue1								10^{-4}	0.83	10^{-7}
ric1-1									10^{-4}	0.2
rop4-1										10^{-6}

The number of lobes for the different pavement cell genotypes in Fig. 7c are used to do a pairwise comparison using the two-sided Dunn's post-hoc test with Benjamini-Hochberg correction. Non-significant adjusted p-values (p-value > 0.05) are highlighted in gray and are used to create a graph displaying clusters of genotypes with similar phenotypes (Supplementary Fig. 21).

Supplementary Table 7: Adjusted p-values of the pairwise differences for each pair of clades.

Solidity				
	Ferns	Gymnosperms	EDA lineages	Eudicots
Gymnosperms	10^{-82}			
EDA lineages	10^{-37}	0.84		
Eudicots	10^{-274}	10^{-6}	10^{-3}	
Monocots	10^{-276}	10^{-29}	10^{-16}	10^{-25}
Aspect ratio				
	Ferns	Gymnosperms	EDA lineages	Eudicots
Gymnosperms	10^{-56}			
EDA lineages	10^{-12}	10^{-61}		
Eudicots	10^{-59}	10^{-199}	0.77	
Monocots	10^{-11}	10^{-21}	10^{-27}	10^{-111}
Number of lobes per cell				
	Ferns	Gymnosperms	EDA lineages	Eudicots
Gymnosperms	10^{-296}			
EDA lineages	10^{-29}	10^{-37}		
Eudicots	10^{-173}	10^{-94}	0.17	
Monocots	10^{-255}	10^{-11}	10^{-18}	10^{-53}
Completeness				
	Ferns	Gymnosperms	EDA lineages	Eudicots
Gymnosperms	10^{-56}			
EDA lineages	10^{-53}	10^{-5}		
Eudicots	0.0	10^{-32}	10^{-3}	
Monocots	10^{-160}	10^{-13}	0.52	10^{-4}

The shape metrics listed in Supplementary Fig. 23 are used to do a pairwise comparison using the two-sided Dunn's post-hoc test between the different clades for the solidity, aspect ratio, number of lobes per cell and relative completeness. Non-significant adjusted p-values (p-value < 0.01, Bemjamini-Hochberg correction) are highlighted in gray.

Supplementary Table 8: Pixel accuracy of three manual segmented pavement cell images.

Image	TP	TN	FP	FN	Pixel accuracy (%)
a	1493	133222	392	4588	96.4
b	1016	54432	114	2670	95.2
c	874	31680	64	2230	93.4
					95.0

Three different wild-type images (Supplementary Fig. 24) are selected for manual segmentation of pavement cells and for pre-processing with GraVis. The manual segmented cells are used as ground truth for the calculation of the pixel accuracy of the segmented cells by GraVis. The average pixel accuracy is 95%.

Supplementary References

1. Brandes, U., Delling, D., Gaertler, M., Gorke, R., Hoefer, M., Nikoloski, Z., Wagner, D. On modularity clustering. *IEEE Transactions on Knowledge and Data Engineering* **20**(2): 172-188 (2008).
2. Newman, M. Communities, modules and large-scale structure in networks. *Nature Physics* **8**: 25-31 (2012).
3. Datta, S. & Datta, S. Methods for evaluating clustering algorithms for gene expression data using a reference set of functional classes. *BMC Bioinformatics* **7**: 397 (2006).
4. Brandes, U. & Fleischer, D. Centrality measures based on current flow. *Proc 22nd Symp. Theoretical Aspects of Computer Science (STACS'05),* **LNCS 3404**: 533-544 (2005).
5. Vőfély, R. V., Gallagher, J., Pisano, G. D., Bartlett, M., Braybrook, S. A. Data from: Of puzzles and pavements: a quantitative exploration of leaf epidermal cell shape. Dryad Digital Repository. https://doi.org/10.5061/dryad.g4q6pv3 (2018).
6. Freeman, L. C. Centrality in social networks: conceptual clarification. *Social Networks* **1**(3), 215-239 (1979).
7. Bonacich, P. Power and centrality: a family of measures. *American Journal of Sociology* **92**(5), 1170-1182 (1987).
8. Sabidussi, G. The centrality index of a graph. *Psychometrika* **31**, 581-603 (1966).
9. Stephenson, K. & Zelen, M. Rethinking centrality: methods and examples. *Social Networks* **11**(1), 1-37 (1989).
10. Freeman, L. C. A set of measures of centrality based on betweenness. *Sociometry* **40**(1), 35-41 (1977).
11. Newman, M. E. J. A measure of betweenness centrality based on random walks. *Social Networks* **27**(1), 39-54 (2005).
12. Goh, K. I., Kahng, B., Kim, D. Universal behaviour of load distribution in scale-free networks. *Physical Review Letters* **87**, 278701 (2001).
13. Marchiori, M. & Latora, V. Harmony in the small-world. *Physica A: Statistical Mechanics and its Applications* **285**(3-4), 539-546 (2000).
14. Mones, E., Vicsek, L., Vicsek, T. Hierarchy measure for complex networks. *PLoS ONE* **7**(3), e33799 (2012).
