# Supplementary material for: A network-based framework for shape analysis enables accurate characterization of leaf epidermal cells
Source: Nat Commun. 2021 Jan 19;12:458. doi: 10.1038/s41467-020-20730-y (PMC7815848; doi:10.1038/s41467-020-20730-y)
Supplement: Supplementary file 3 — Reporting Summary [file 41467_2020_20730_MOESM3_ESM.pdf]

nature research

Corresponding author(s): Zoran Nikoloski

Last updated by author(s): Dec 13, 2020

Reporting Summary

Nature Research wishes to improve the reproducibility of the work that we publish. This form provides structure for consistency and transparency in reporting. For further information on Nature Research policies, see Authors & Referees and the Editorial Policy Checklist .

Statistics

For all statistical analyses, confirm that the following items are present in the figure legend, table legend, main text, or Methods section.

n/a	Confirmed	
☐	☒	The exact sample size (*n*) for each experimental group/condition, given as a discrete number and unit of measurement
☐	☒	A statement on whether measurements were taken from distinct samples or whether the same sample was measured repeatedly
☐	☒	The statistical test(s) used AND whether they are one- or two-sided<br>*Only common tests should be described solely by name; describe more complex techniques in the Methods section.*
☒	☐	A description of all covariates tested
☒	☐	A description of any assumptions or corrections, such as tests of normality and adjustment for multiple comparisons
☐	☒	A full description of the statistical parameters including central tendency (e.g. means) or other basic estimates (e.g. regression coefficient) AND variation (e.g. standard deviation) or associated estimates of uncertainty (e.g. confidence intervals)
☐	☒	For null hypothesis testing, the test statistic (e.g. *F*, *t*, *r*) with confidence intervals, effect sizes, degrees of freedom and *P* value noted<br>*Give P values as exact values whenever suitable.*
☒	☐	For Bayesian analysis, information on the choice of priors and Markov chain Monte Carlo settings
☒	☐	For hierarchical and complex designs, identification of the appropriate level for tests and full reporting of outcomes
☐	☒	Estimates of effect sizes (e.g. Cohen's *d*, Pearson's *r*), indicating how they were calculated

Our web collection on statistics for biologists contains articles on many of the points above.

Software and code

Policy information about availability of computer code

Data collection	All images of pavement cells were captured with a spinning-disk confocal microscope (Roper Scientific) with a 60x objective lens. Additional imaging data was collected from a set of synthetic shapes and a brightfield microscope of sand grains.
Data analysis	The images were processed and analyzed using Python (Version 3.5.2) and a Python-based framework (https://github.com/jnowak90/GraVisGUI, (https://doi.org/10.5281/zenodo.4320828). Additional image analysis was done using ImageJ (Version 2.0.0-rc-69/1.52p) and PaCeQuant from the Mitobo plugin (Version 1.8.6). Furthermore, we used the tool LobeFinder (Version 1.0) in Matlab (Version R2019b) and the tool LOCO-EFA (Version 145) in the terminal (Version 2.8.3, MacOS).

For manuscripts utilizing custom algorithms or software that are central to the research but not yet described in published literature, software must be made available to editors/reviewers. We strongly encourage code deposition in a community repository (e.g. GitHub). See the Nature Research guidelines for submitting code & software for further information.

Data

Policy information about availability of data

All manuscripts must include a data availability statement. This statement should provide the following information, where applicable:
- Accession codes, unique identifiers, or web links for publicly available datasets
- A list of figures that have associated raw data
- A description of any restrictions on data availability

The data supporting the manuscript is accessible on Github (https://doi.org/10.5281/zenodo.4320828). We used publicly available datasets from a publication by Vöfely et al., New Phytologist 2018 (https://doi.org/10.5061/dryad.g4q6pv3) and from the public database WoRMS (http://www.marinespecies.org).

Field-specific reporting

Please select the one below that is the best fit for your research. If you are not sure, read the appropriate sections before making your selection.

- [x] Life sciences
- [ ] Behavioural & social sciences
- [ ] Ecological, evolutionary & environmental sciences

For a reference copy of the document with all sections, see nature.com/documents/nr-reporting-summary-flat.pdf

Life sciences study design

All studies must disclose on these points even when the disclosure is negative.

Sample size	Sample sizes of pavement cells were chosen based on the number of suitable segmented cells. For the genetically modified lines of pavement cells, we determined the number of analyzed cells by finding the minimal number of suitable cells for each genotype. For the pavement cell gold standard for the manual detection of lobes, we selected 10 cells for each condition (wildtype, clasp-1, oryzalin treatment) to have a high enough number to analyze the variation between manually detected lobes by the different experts.
Data exclusions	No data was excluded.
Replication	At least 5 biological replicates were imaged for the selected genetically modified lines of pavement cells. All replications were successful.
Randomization	Randomization was not applicable for imaging data.
Blinding	Blinding was not applicable for imaging data.

Reporting for specific materials, systems and methods

We require information from authors about some types of materials, experimental systems and methods used in many studies. Here, indicate whether each material, system or method listed is relevant to your study. If you are not sure if a list item applies to your research, read the appropriate section before selecting a response.

Materials & experimental systems

n/a	Involved in the study
x	☐ Antibodies
x	☐ Eukaryotic cell lines
x	☐ Palaeontology
x	☐ Animals and other organisms
x	☐ Human research participants
x	☐ Clinical data

Methods

n/a	Involved in the study
x	☐ ChIP-seq
x	☐ Flow cytometry
x	☐ MRI-based neuroimaging
