# Supplementary figures and images for: A network-based framework for shape analysis enables accurate characterization of leaf epidermal cells (part 2 of 2)
Source: Nat Commun. 2021 Jan 19;12:458. doi: 10.1038/s41467-020-20730-y (PMC7815848; doi:10.1038/s41467-020-20730-y)

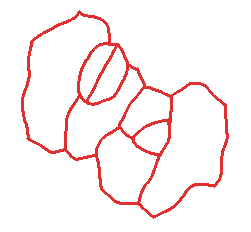

Supplement: Supplementary file 4 — Source Data [file 41467_2020_20730_MOESM4_ESM.zip › SourceData/FigureS24_SegmentationQuality/FigS24_ManualSegmentation_72h.png]

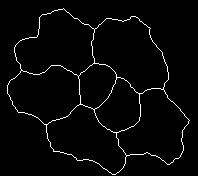

Supplement: Supplementary file 4 — Source Data [file 41467_2020_20730_MOESM4_ESM.zip › SourceData/FigureS24_SegmentationQuality/FigS24_GraVis_skeletonImage_0h.png]

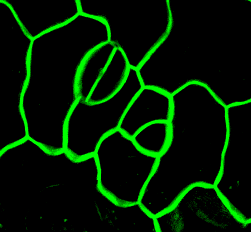

Supplement: Supplementary file 4 — Source Data [file 41467_2020_20730_MOESM4_ESM.zip › SourceData/FigureS24_SegmentationQuality/FigS24_WT_72h-GFP.tif]

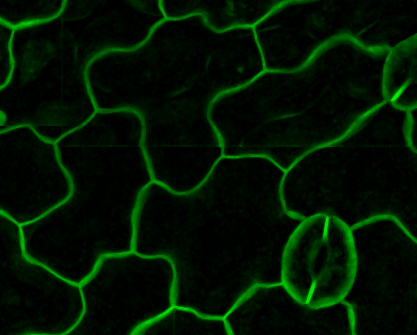

Supplement: Supplementary file 4 — Source Data [file 41467_2020_20730_MOESM4_ESM.zip › SourceData/FigureS24_SegmentationQuality/FigS24_WT_96h-GFP.tif]

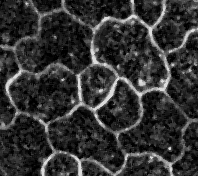

Supplement: Supplementary file 4 — Source Data [file 41467_2020_20730_MOESM4_ESM.zip › SourceData/FigureS24_SegmentationQuality/FigS24_WT_0h-GFP.tif]

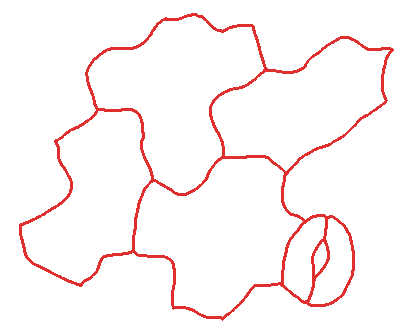

Supplement: Supplementary file 4 — Source Data [file 41467_2020_20730_MOESM4_ESM.zip › SourceData/FigureS24_SegmentationQuality/FigS24_ManualSegmentation_96h.png]

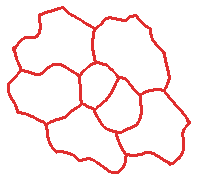

Supplement: Supplementary file 4 — Source Data [file 41467_2020_20730_MOESM4_ESM.zip › SourceData/FigureS24_SegmentationQuality/FigS24_ManualSegmentation_0h.png]

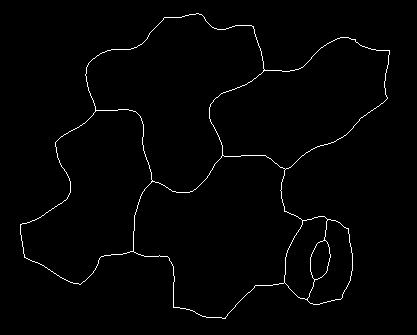

Supplement: Supplementary file 4 — Source Data [file 41467_2020_20730_MOESM4_ESM.zip › SourceData/FigureS24_SegmentationQuality/FigS24_GraVis_skeletonImage_96h.png]

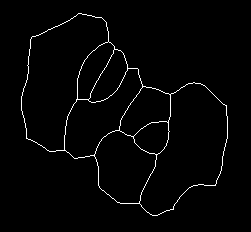

Supplement: Supplementary file 4 — Source Data [file 41467_2020_20730_MOESM4_ESM.zip › SourceData/FigureS24_SegmentationQuality/FigS24_GraVis_skeletonImage_72h.png]

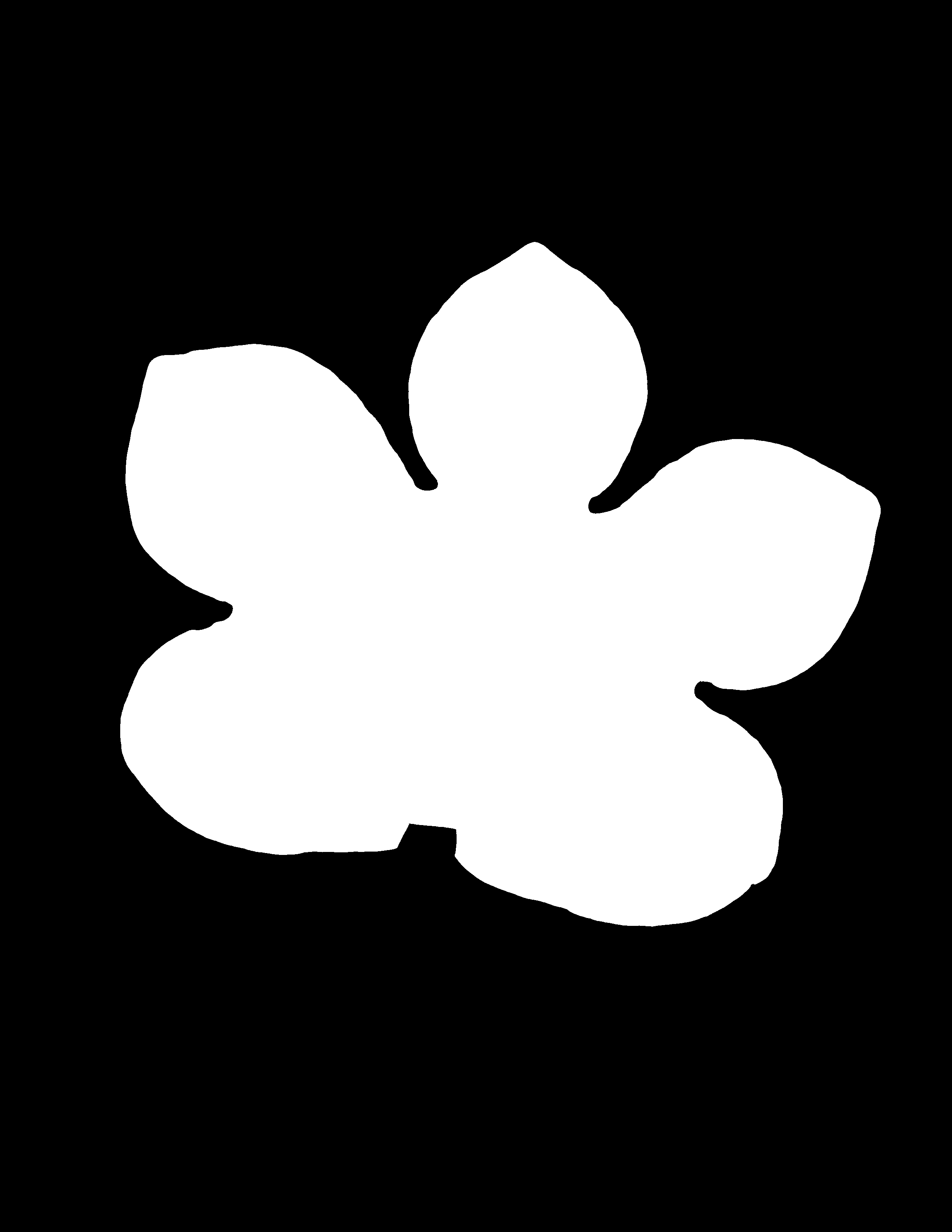

Supplement: Supplementary file 4 — Source Data [file 41467_2020_20730_MOESM4_ESM.zip › SourceData/Figure3_GlobalShapeComparison/Fig3_LeavesLabeled/Leaf1.png]

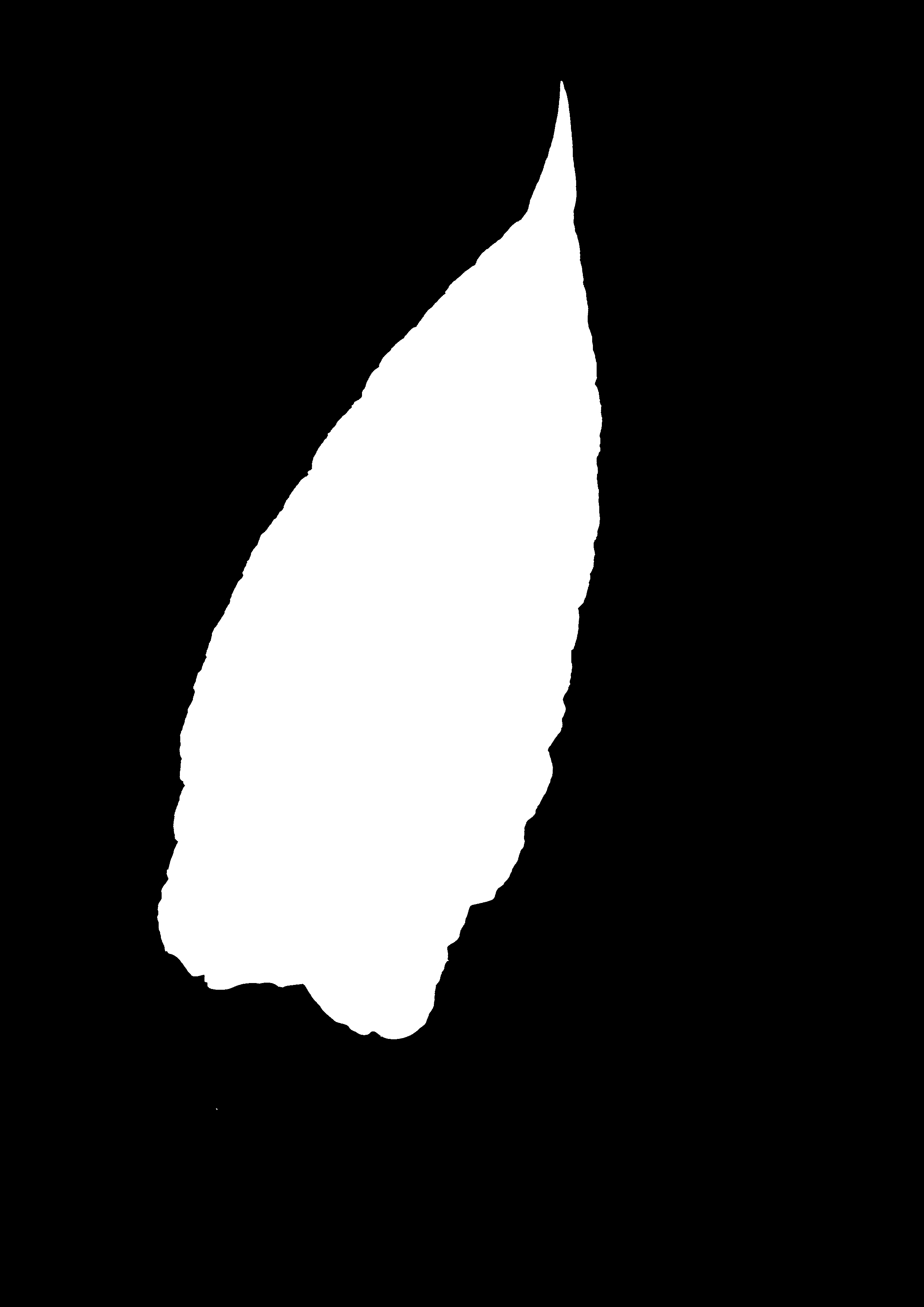

Supplement: Supplementary file 4 — Source Data [file 41467_2020_20730_MOESM4_ESM.zip › SourceData/Figure3_GlobalShapeComparison/Fig3_LeavesLabeled/Leaf2.png]

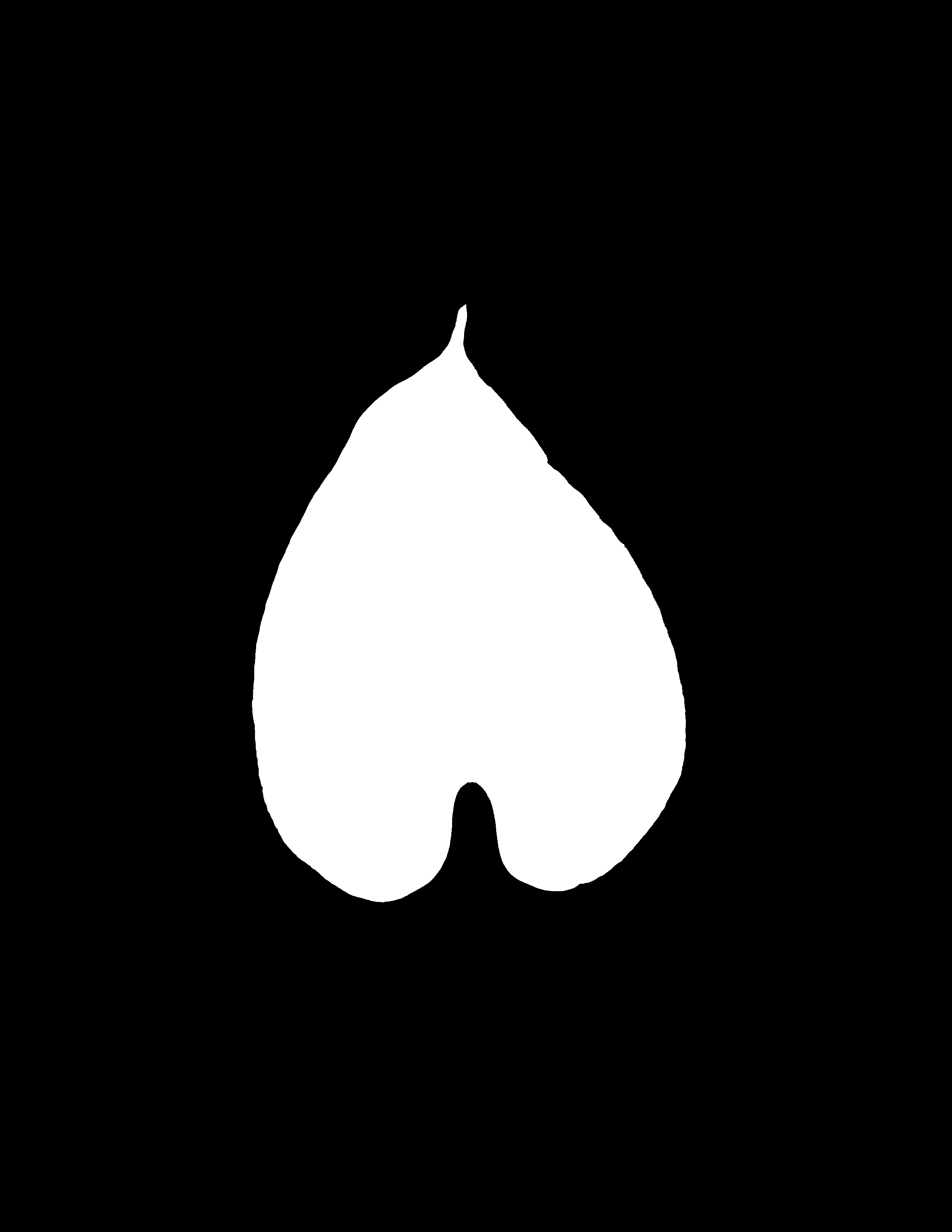

Supplement: Supplementary file 4 — Source Data [file 41467_2020_20730_MOESM4_ESM.zip › SourceData/Figure3_GlobalShapeComparison/Fig3_LeavesLabeled/Leaf3.png]

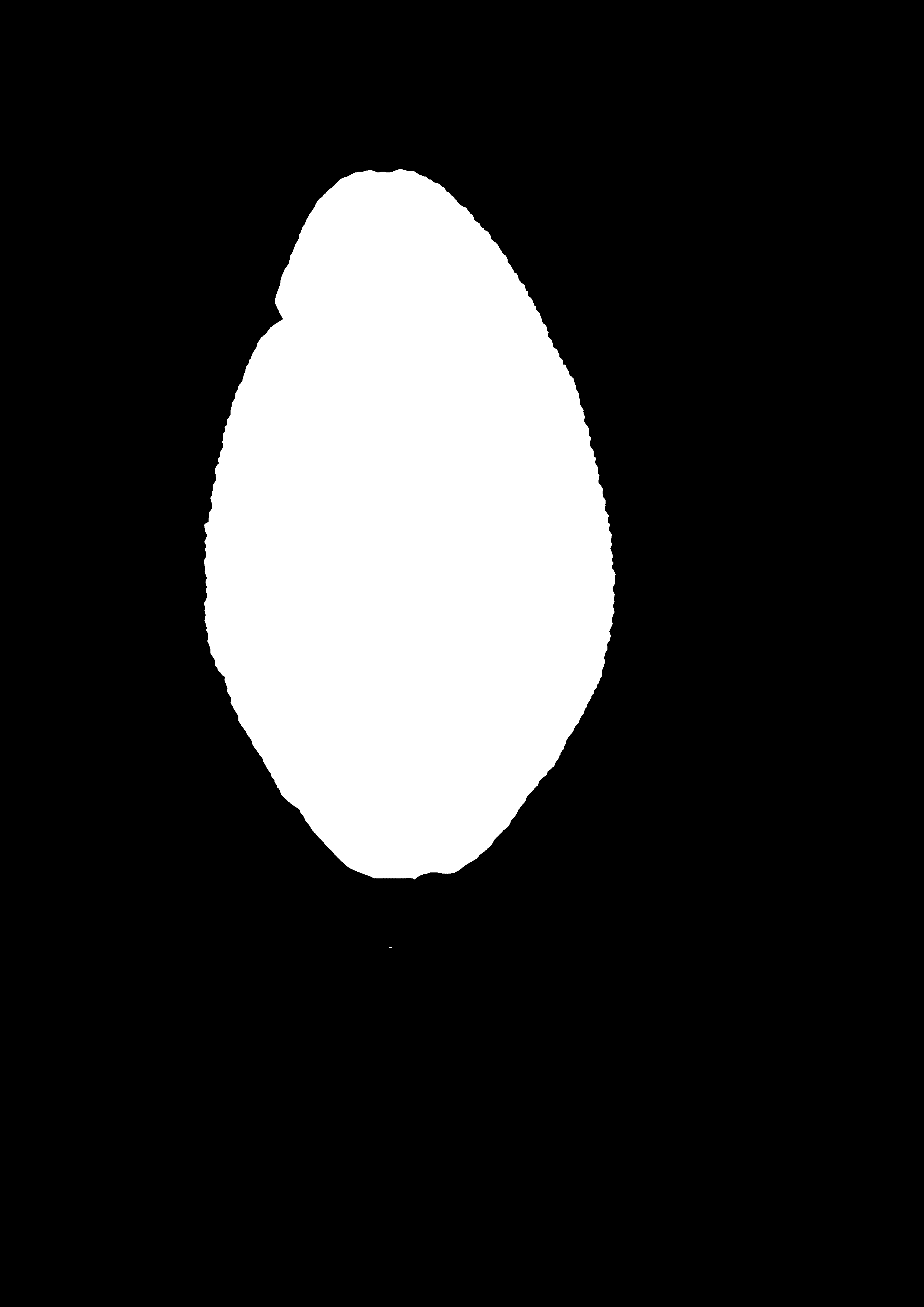

Supplement: Supplementary file 4 — Source Data [file 41467_2020_20730_MOESM4_ESM.zip › SourceData/Figure3_GlobalShapeComparison/Fig3_LeavesLabeled/Leaf20.png]

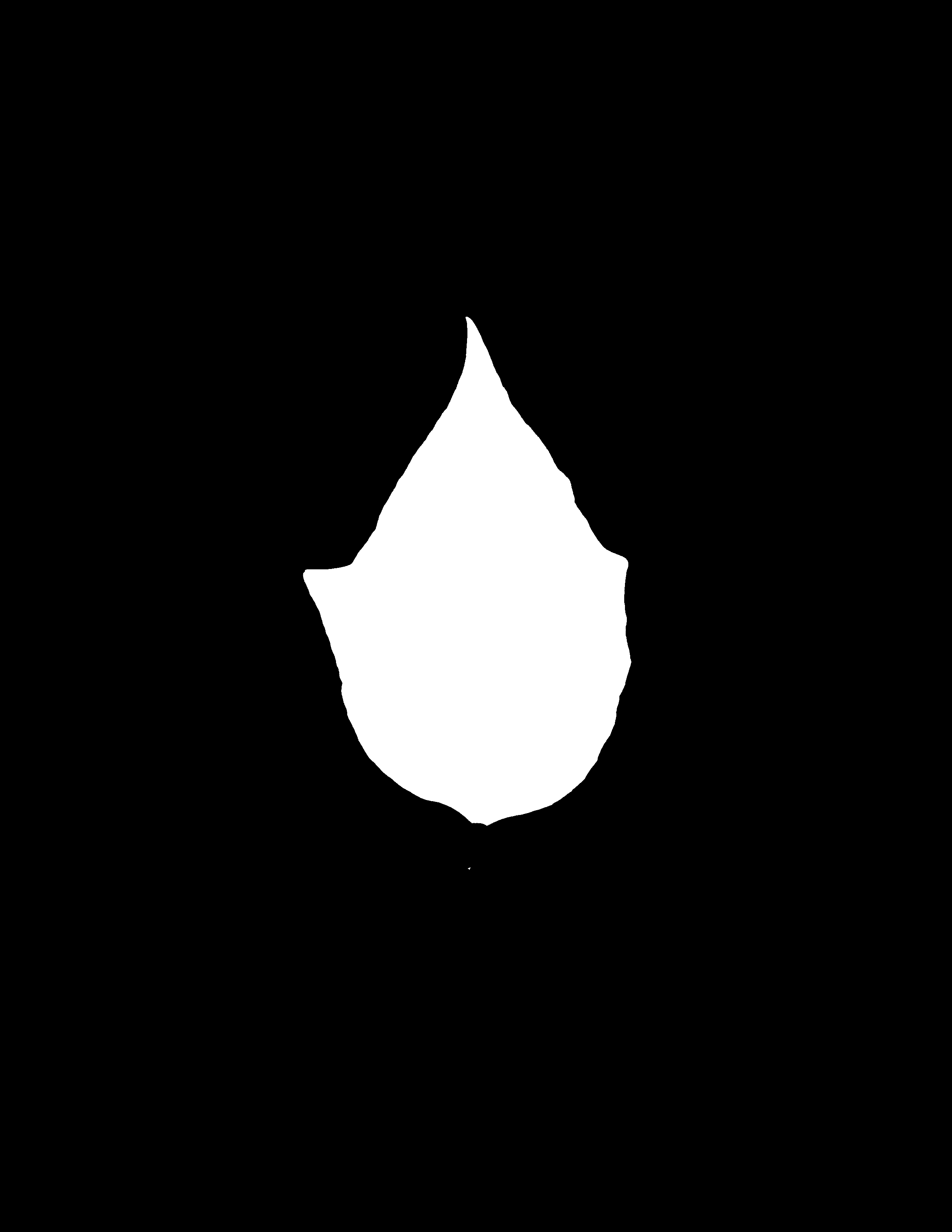

Supplement: Supplementary file 4 — Source Data [file 41467_2020_20730_MOESM4_ESM.zip › SourceData/Figure3_GlobalShapeComparison/Fig3_LeavesLabeled/Leaf18.png]

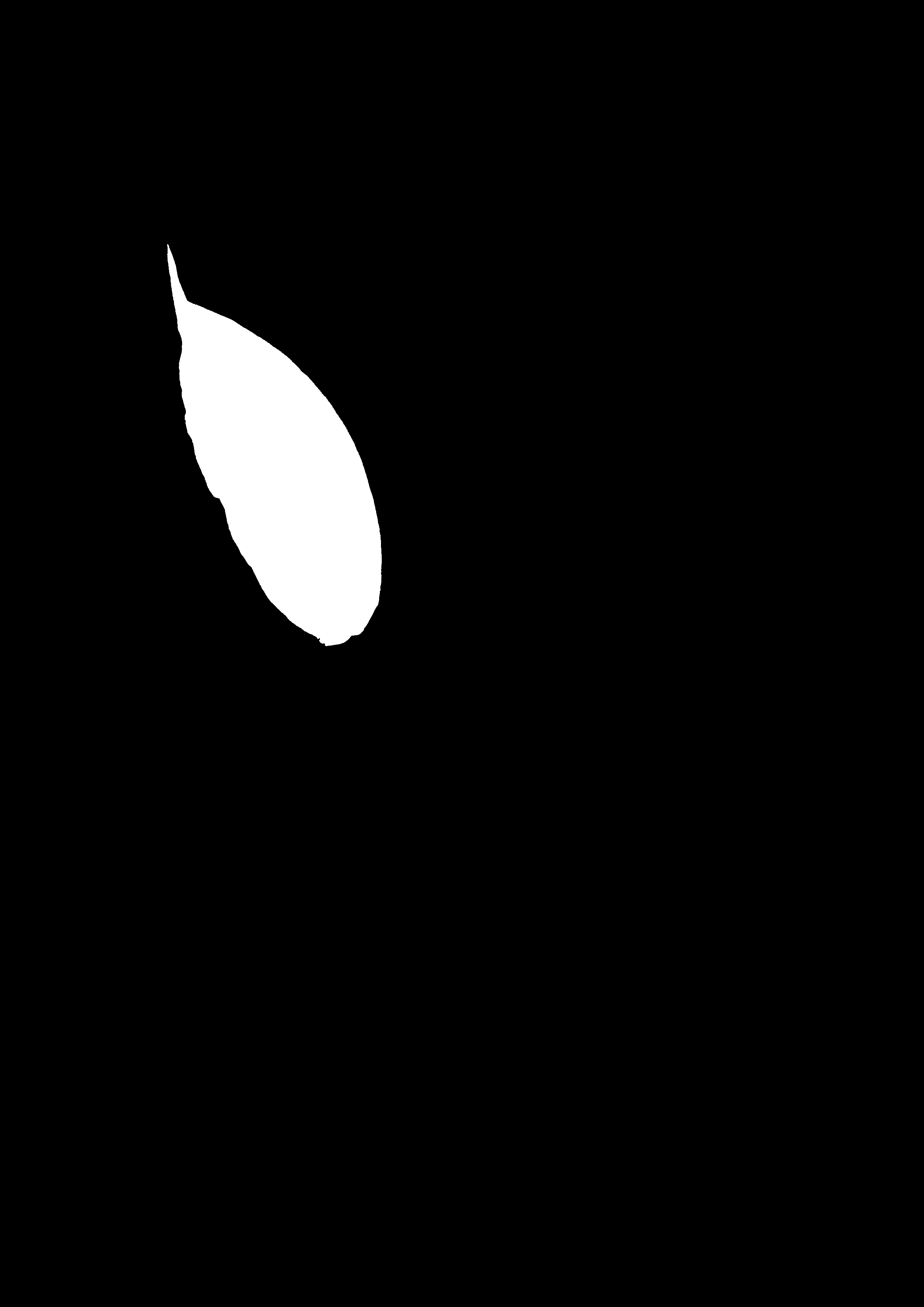

Supplement: Supplementary file 4 — Source Data [file 41467_2020_20730_MOESM4_ESM.zip › SourceData/Figure3_GlobalShapeComparison/Fig3_LeavesLabeled/Leaf7.png]

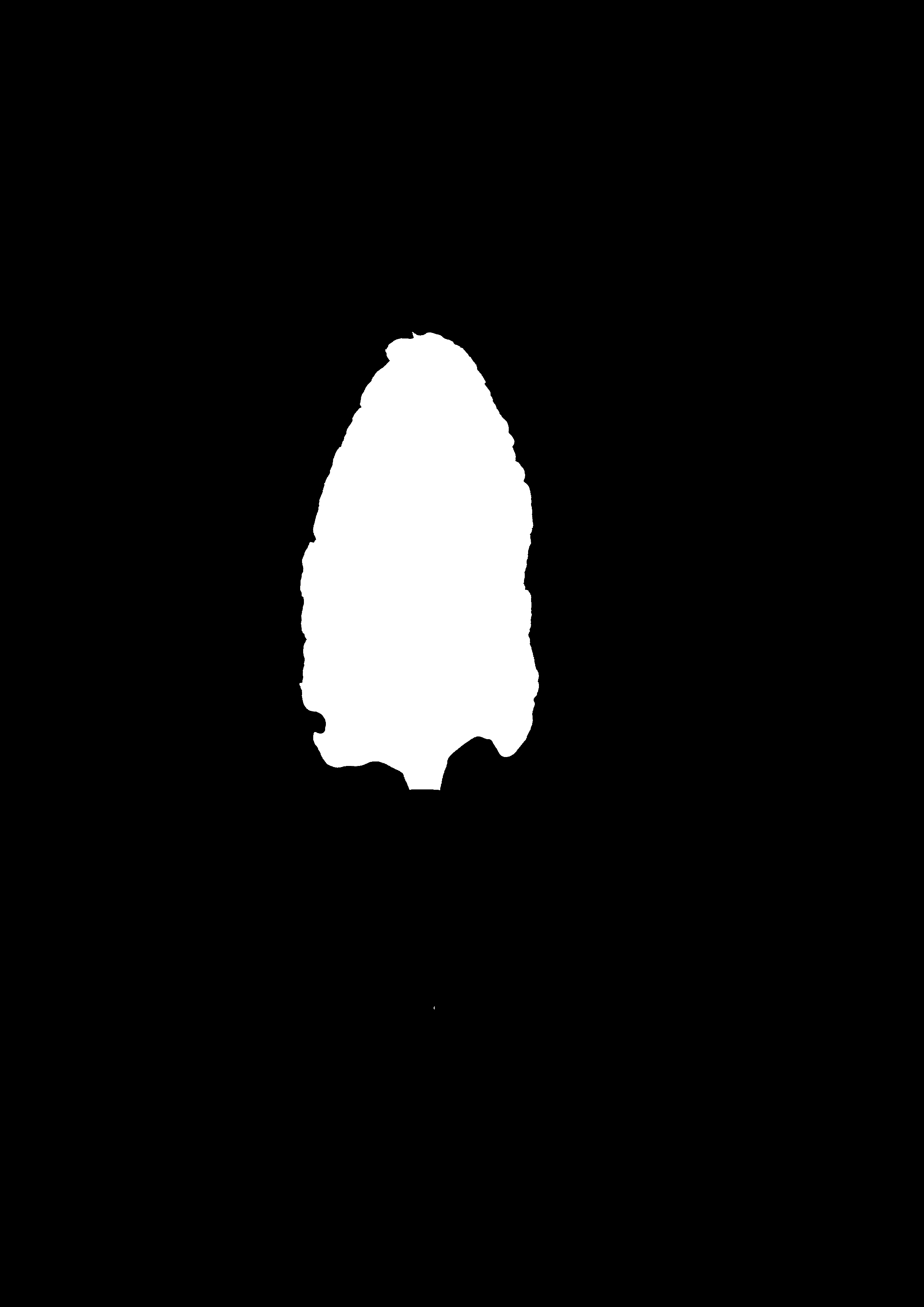

Supplement: Supplementary file 4 — Source Data [file 41467_2020_20730_MOESM4_ESM.zip › SourceData/Figure3_GlobalShapeComparison/Fig3_LeavesLabeled/Leaf6.png]

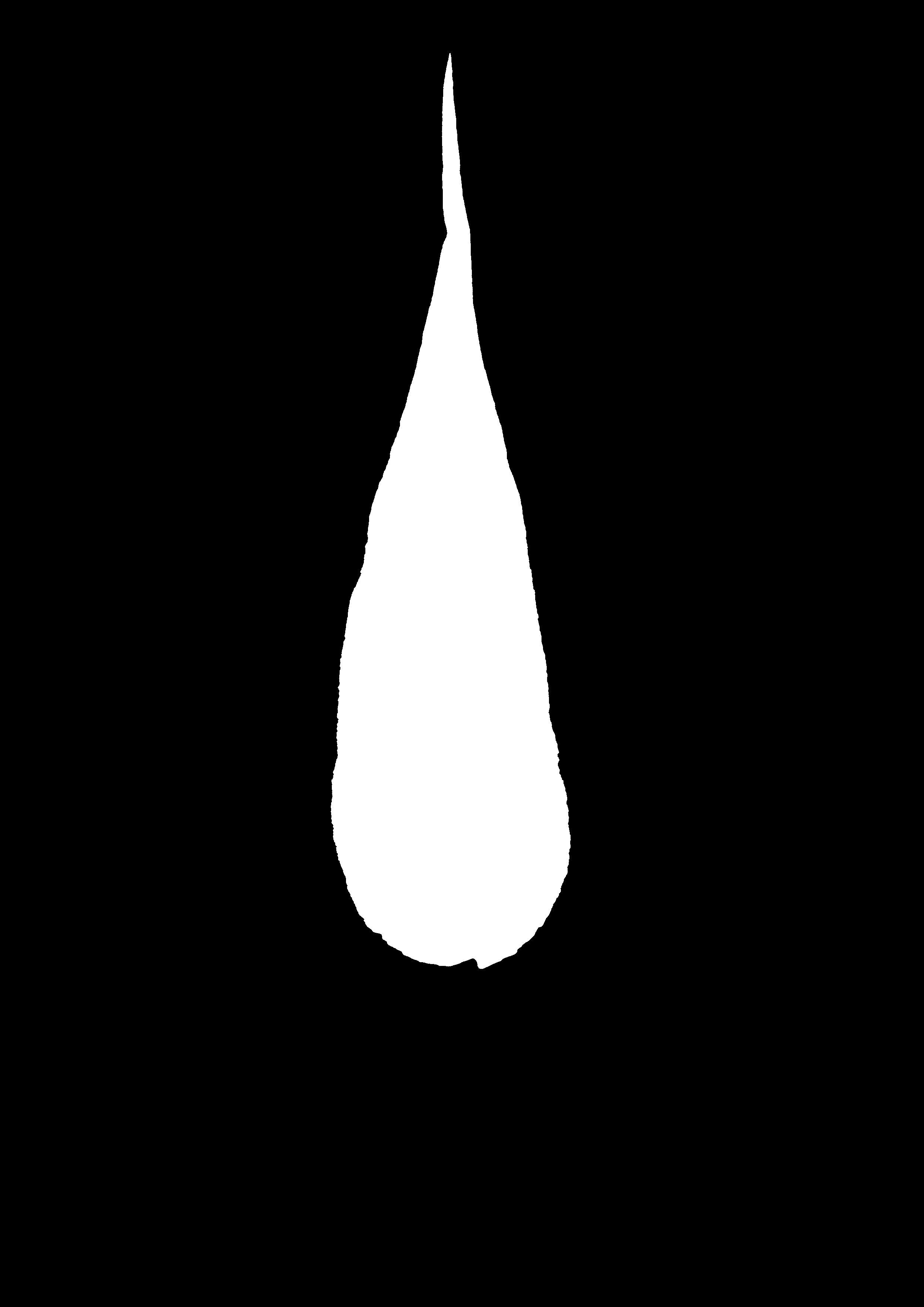

Supplement: Supplementary file 4 — Source Data [file 41467_2020_20730_MOESM4_ESM.zip › SourceData/Figure3_GlobalShapeComparison/Fig3_LeavesLabeled/Leaf19.png]

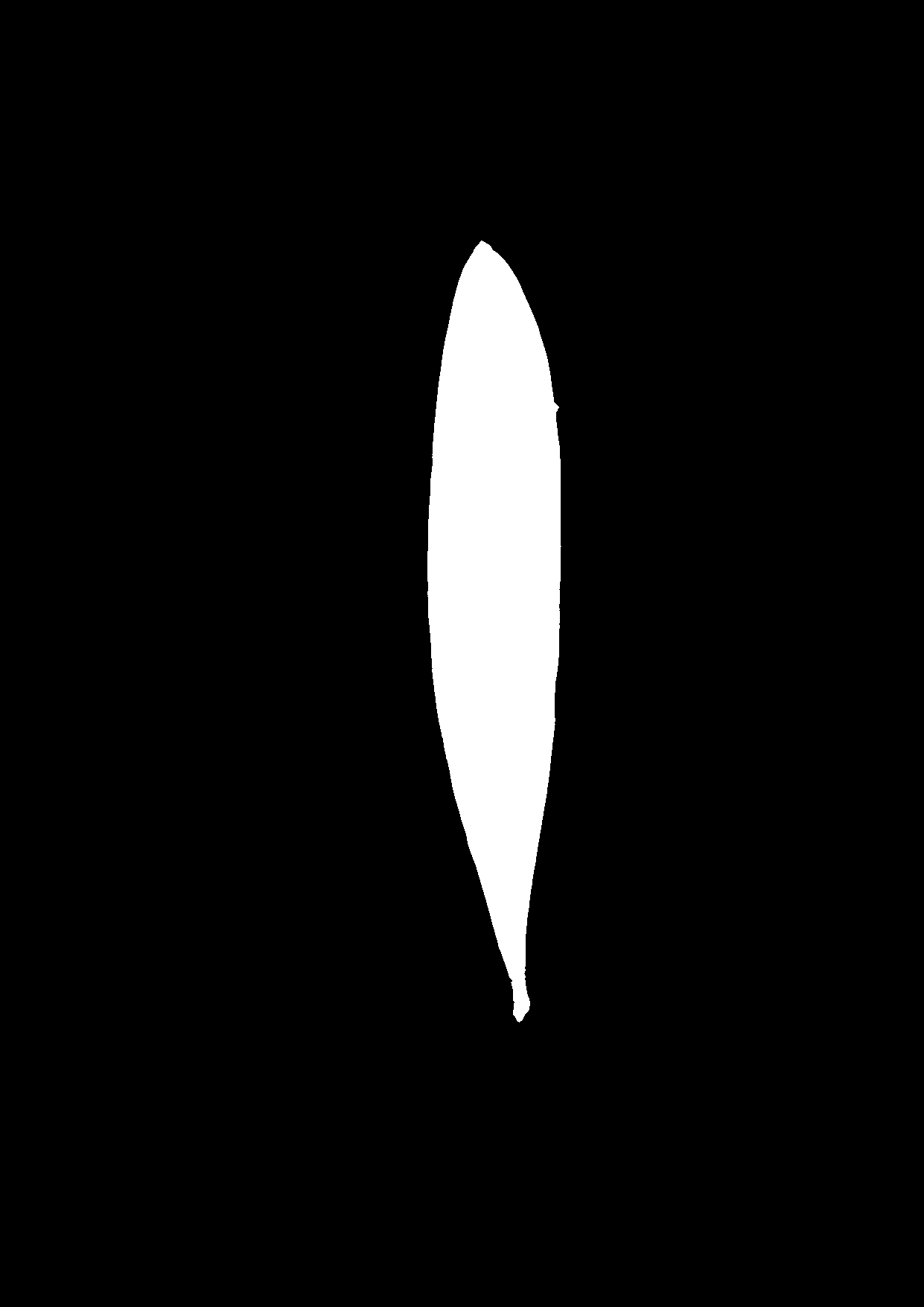

Supplement: Supplementary file 4 — Source Data [file 41467_2020_20730_MOESM4_ESM.zip › SourceData/Figure3_GlobalShapeComparison/Fig3_LeavesLabeled/Leaf4.png]

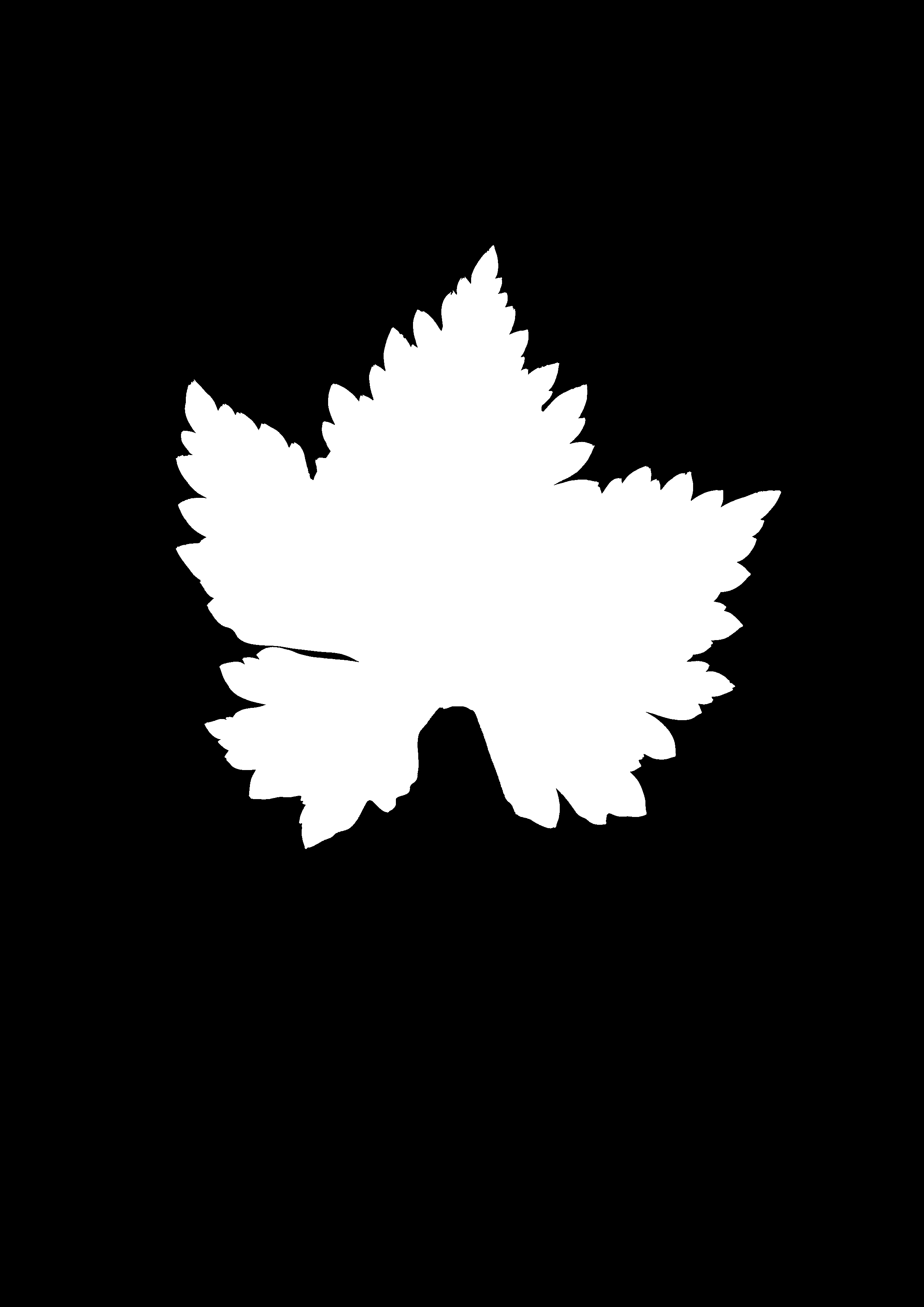

Supplement: Supplementary file 4 — Source Data [file 41467_2020_20730_MOESM4_ESM.zip › SourceData/Figure3_GlobalShapeComparison/Fig3_LeavesLabeled/Leaf5.png]

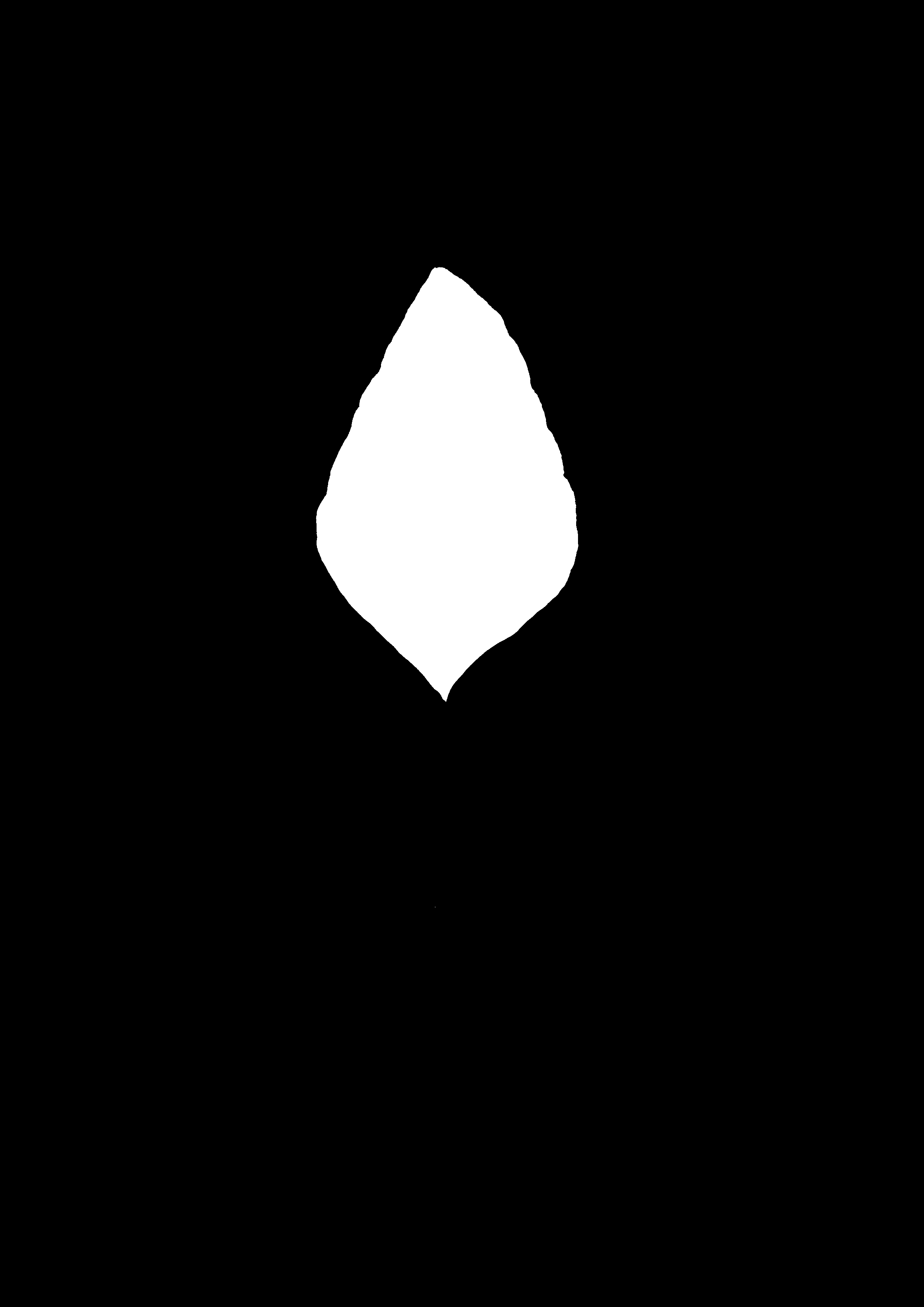

Supplement: Supplementary file 4 — Source Data [file 41467_2020_20730_MOESM4_ESM.zip › SourceData/Figure3_GlobalShapeComparison/Fig3_LeavesLabeled/Leaf17.png]

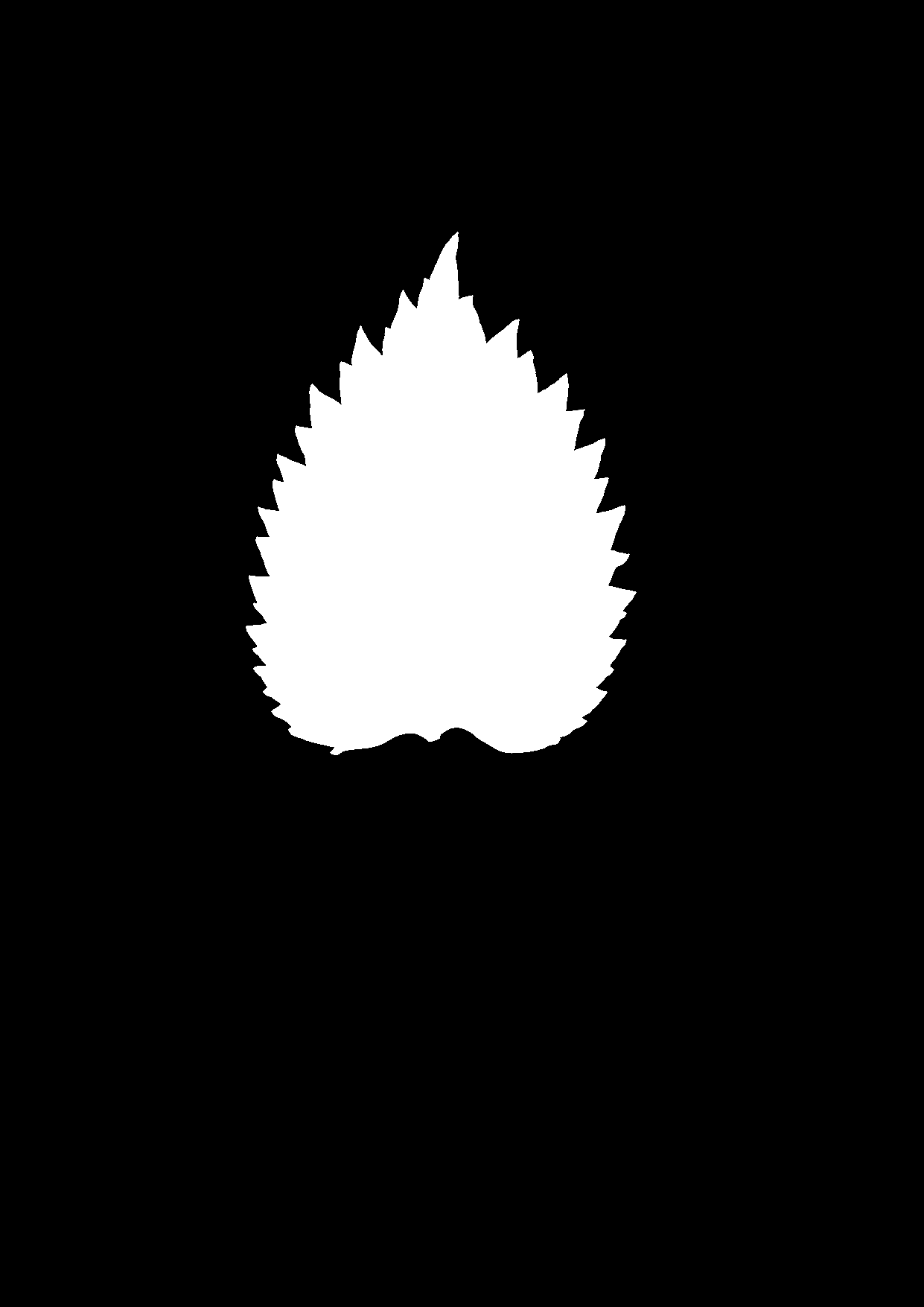

Supplement: Supplementary file 4 — Source Data [file 41467_2020_20730_MOESM4_ESM.zip › SourceData/Figure3_GlobalShapeComparison/Fig3_LeavesLabeled/Leaf8.png]

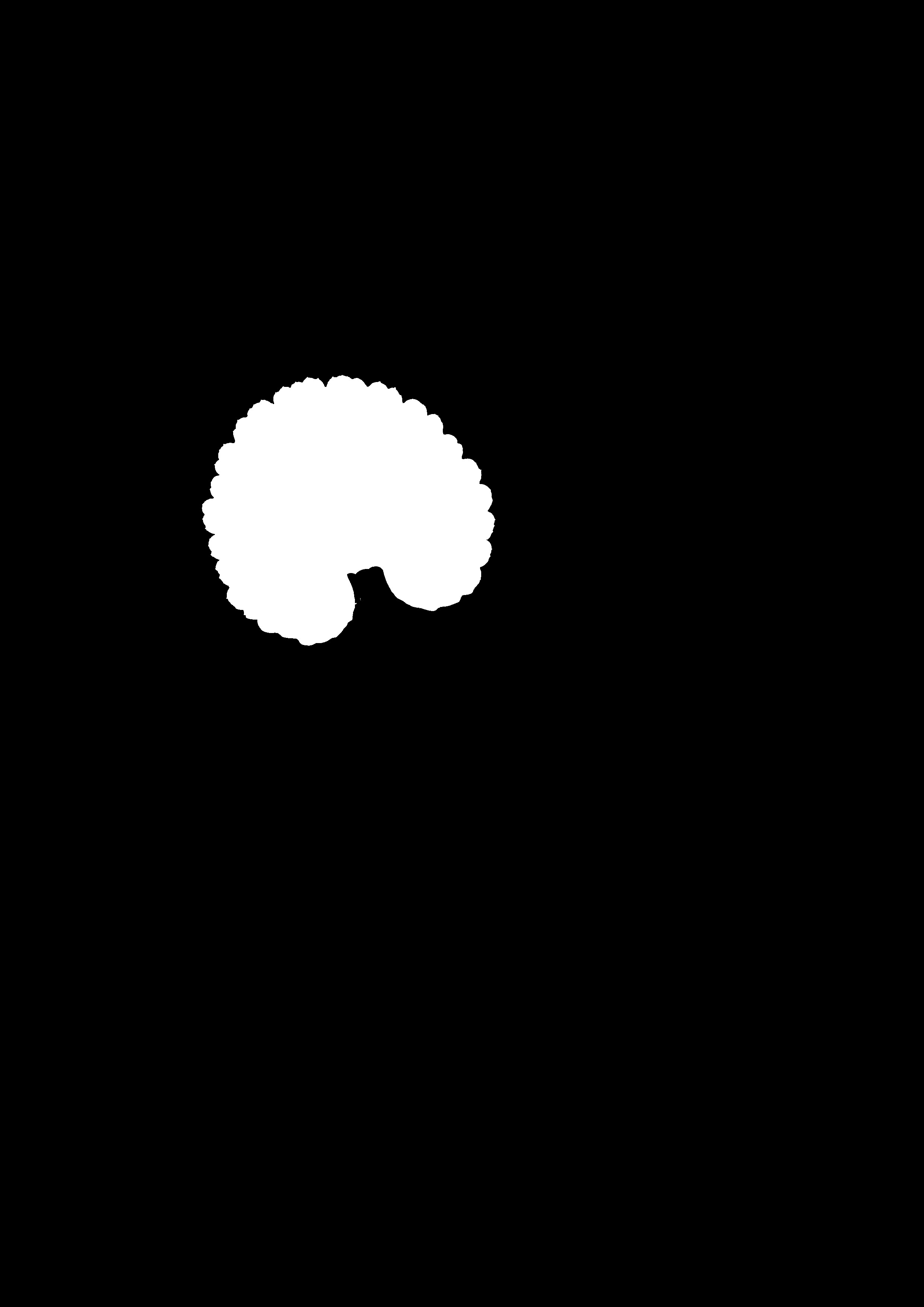

Supplement: Supplementary file 4 — Source Data [file 41467_2020_20730_MOESM4_ESM.zip › SourceData/Figure3_GlobalShapeComparison/Fig3_LeavesLabeled/Leaf9.png]

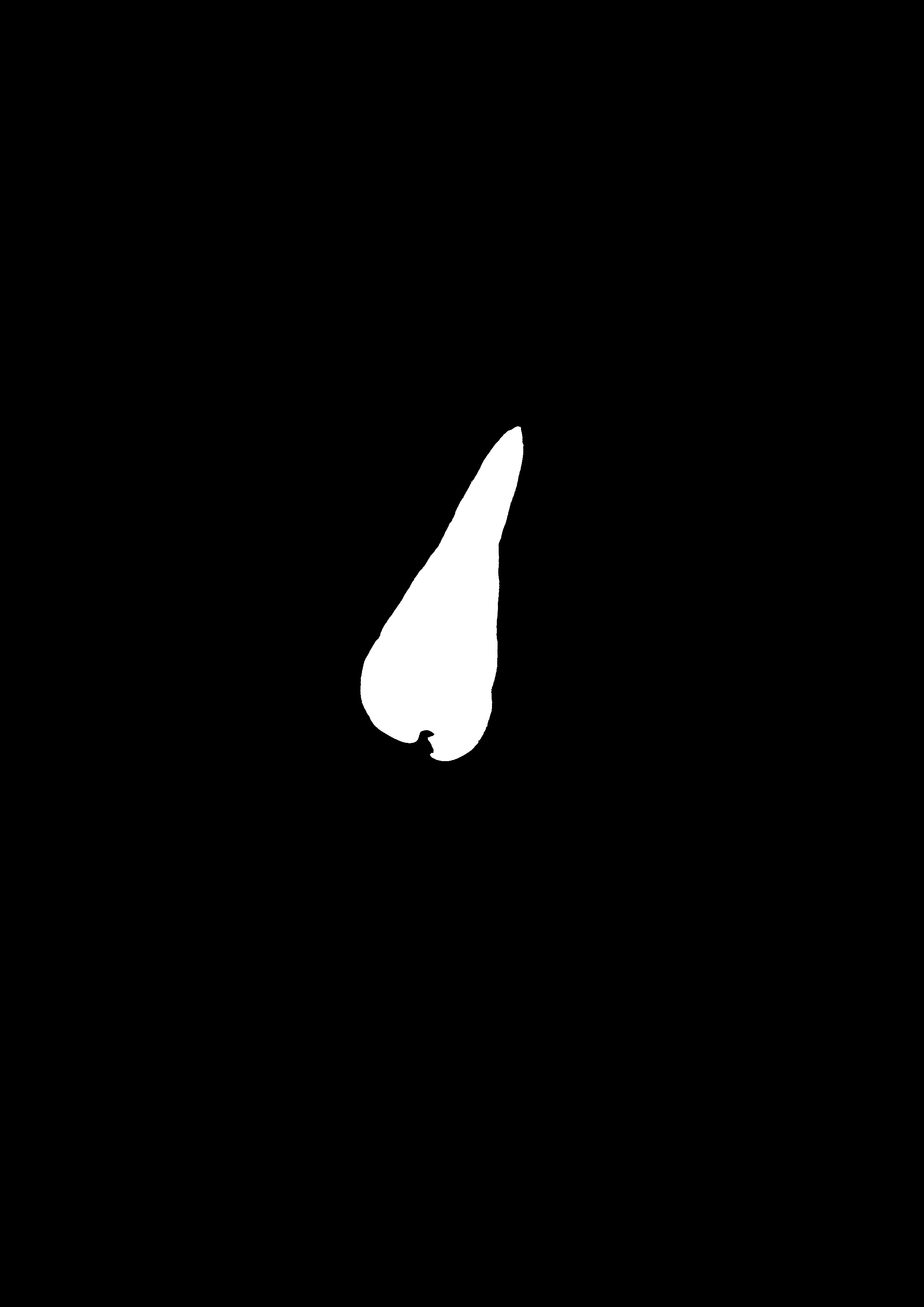

Supplement: Supplementary file 4 — Source Data [file 41467_2020_20730_MOESM4_ESM.zip › SourceData/Figure3_GlobalShapeComparison/Fig3_LeavesLabeled/Leaf16.png]

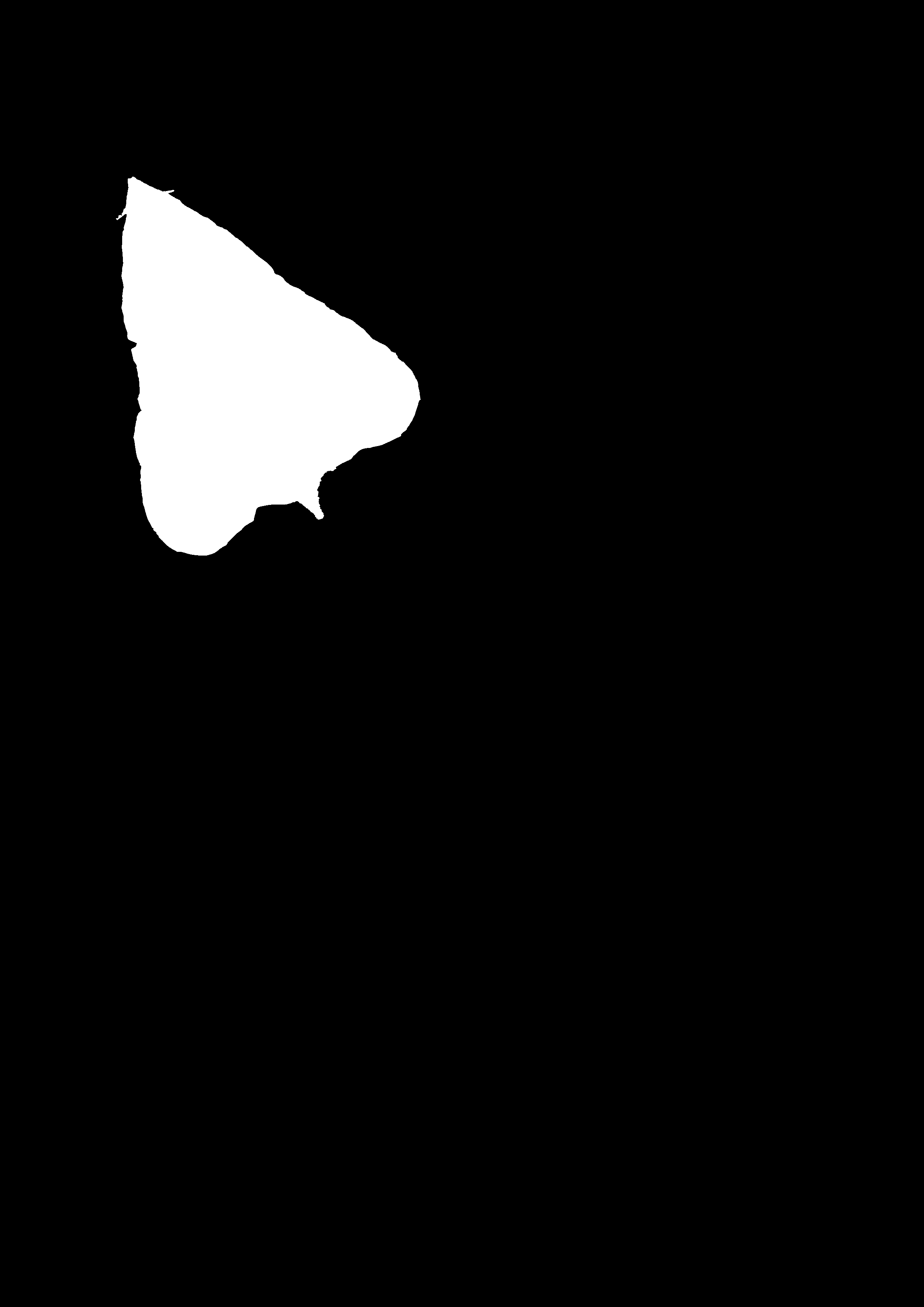

Supplement: Supplementary file 4 — Source Data [file 41467_2020_20730_MOESM4_ESM.zip › SourceData/Figure3_GlobalShapeComparison/Fig3_LeavesLabeled/Leaf14.png]

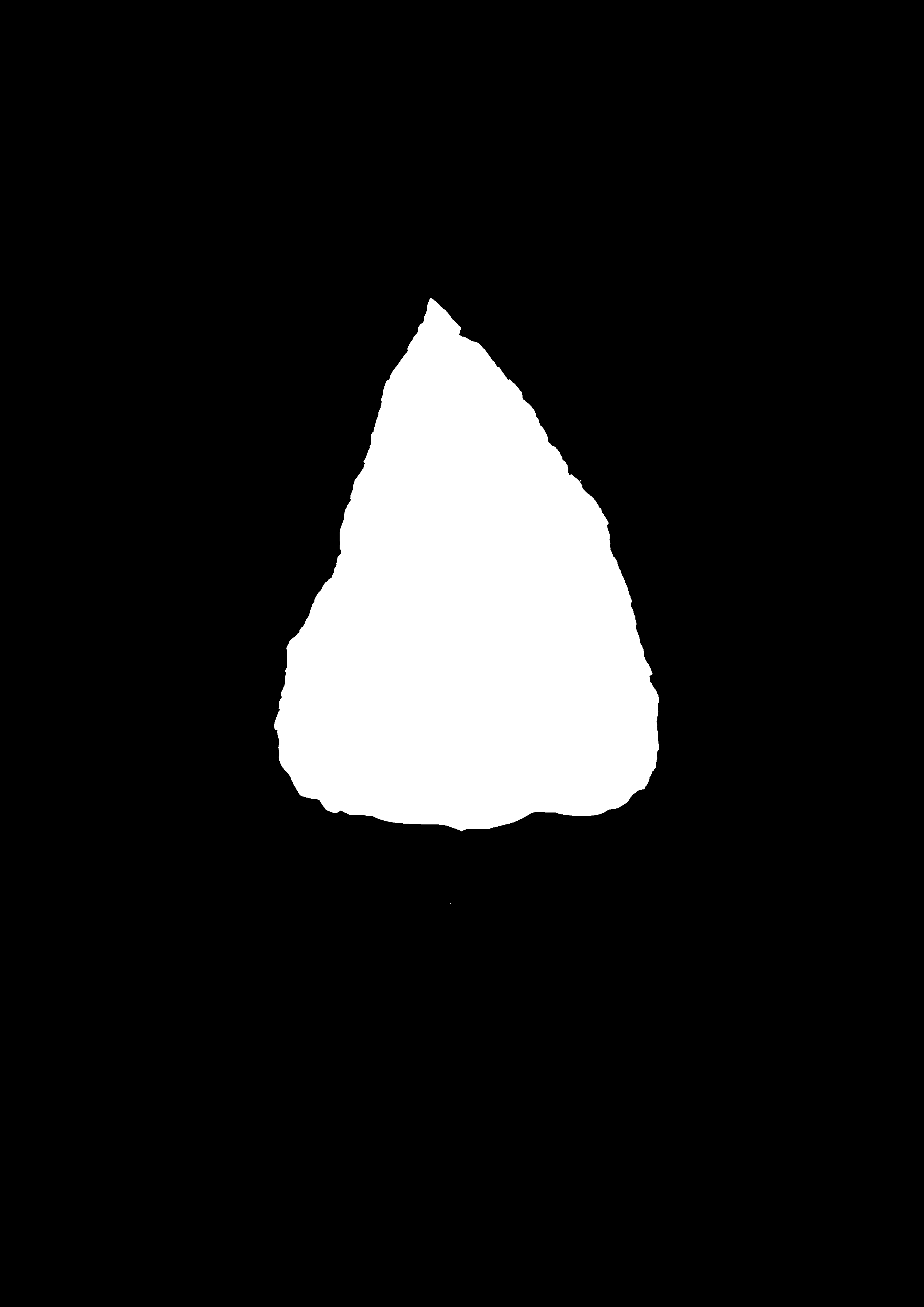

Supplement: Supplementary file 4 — Source Data [file 41467_2020_20730_MOESM4_ESM.zip › SourceData/Figure3_GlobalShapeComparison/Fig3_LeavesLabeled/Leaf15.png]

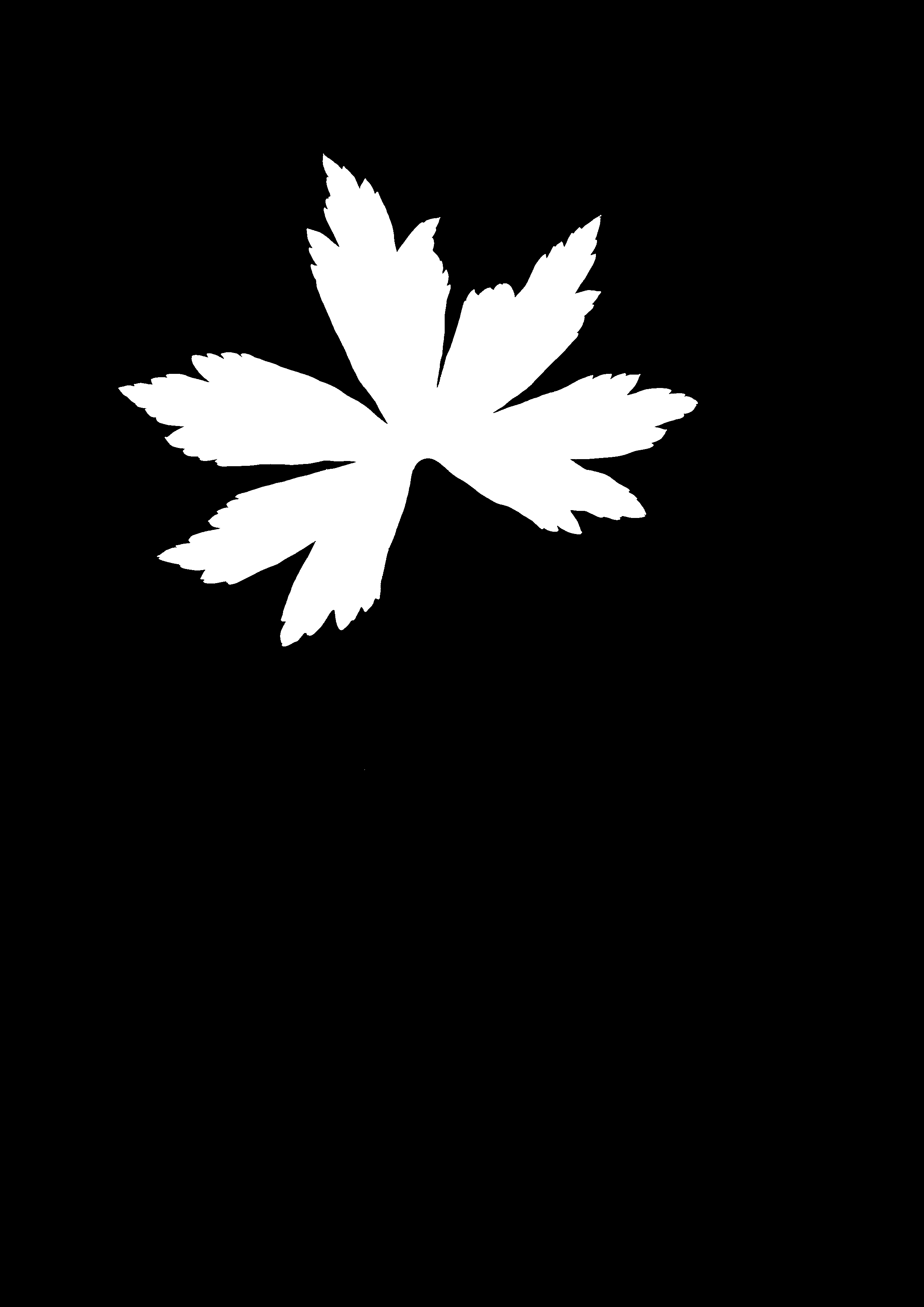

Supplement: Supplementary file 4 — Source Data [file 41467_2020_20730_MOESM4_ESM.zip › SourceData/Figure3_GlobalShapeComparison/Fig3_LeavesLabeled/Leaf11.png]

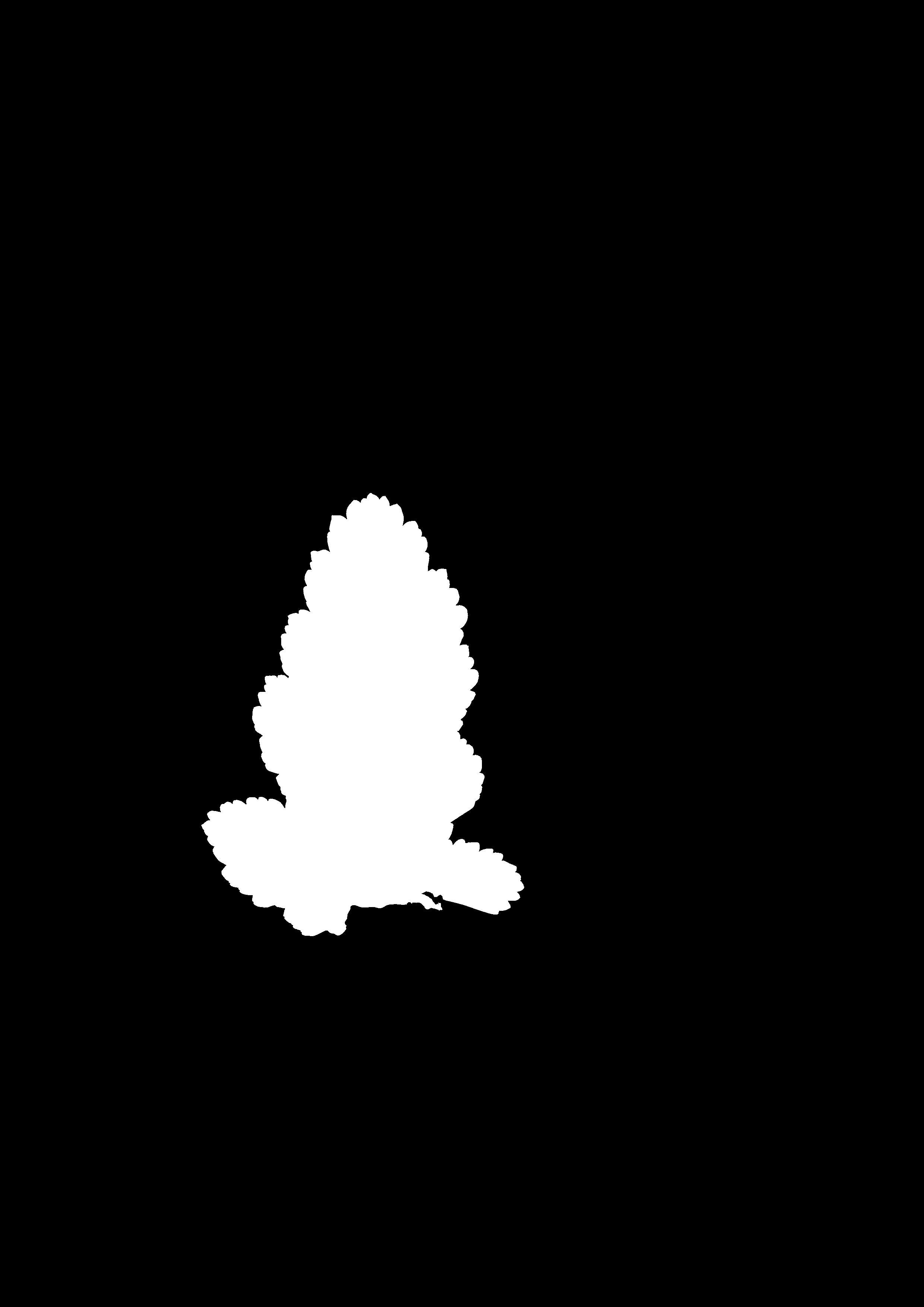

Supplement: Supplementary file 4 — Source Data [file 41467_2020_20730_MOESM4_ESM.zip › SourceData/Figure3_GlobalShapeComparison/Fig3_LeavesLabeled/Leaf10.png]

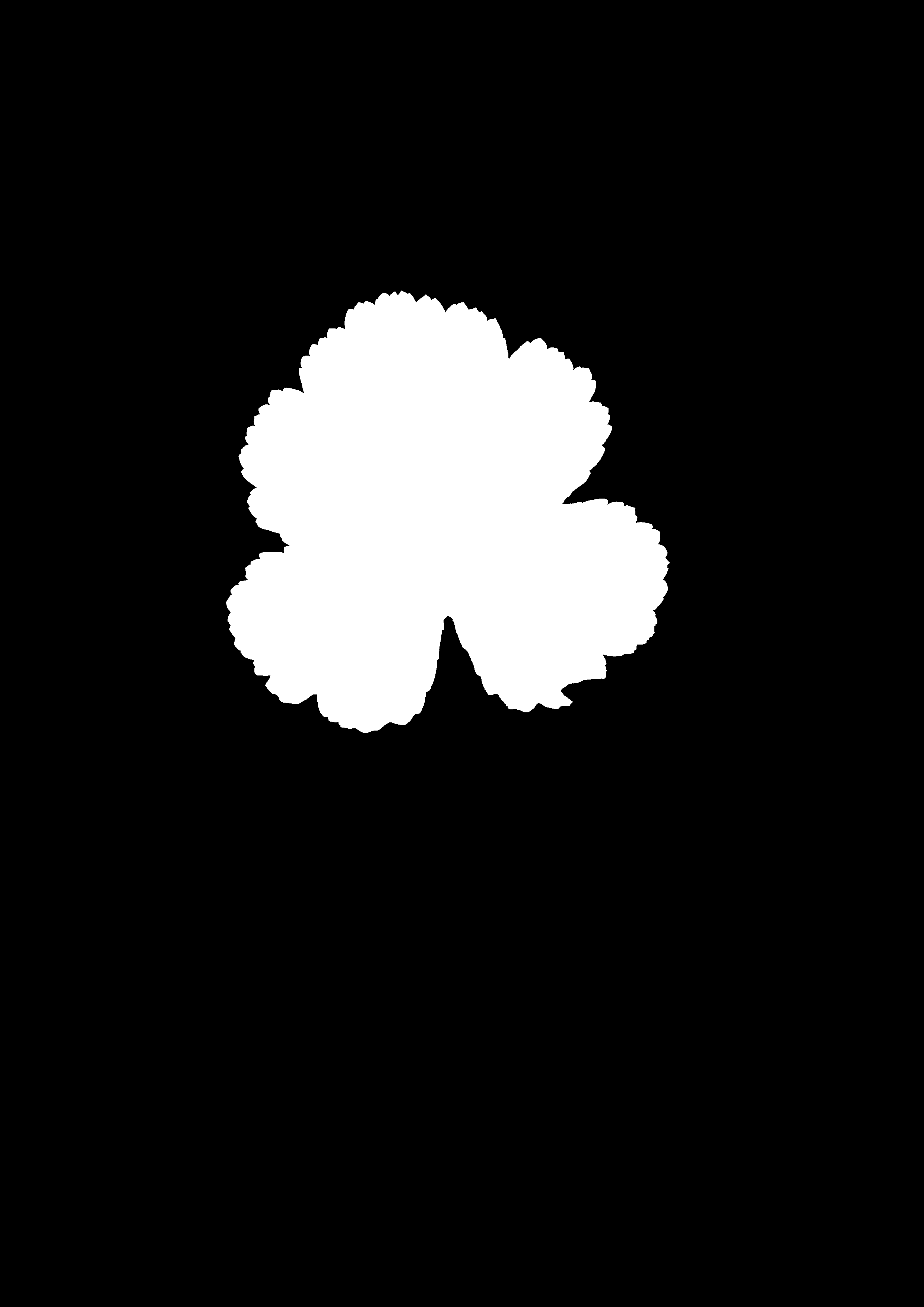

Supplement: Supplementary file 4 — Source Data [file 41467_2020_20730_MOESM4_ESM.zip › SourceData/Figure3_GlobalShapeComparison/Fig3_LeavesLabeled/Leaf12.png]

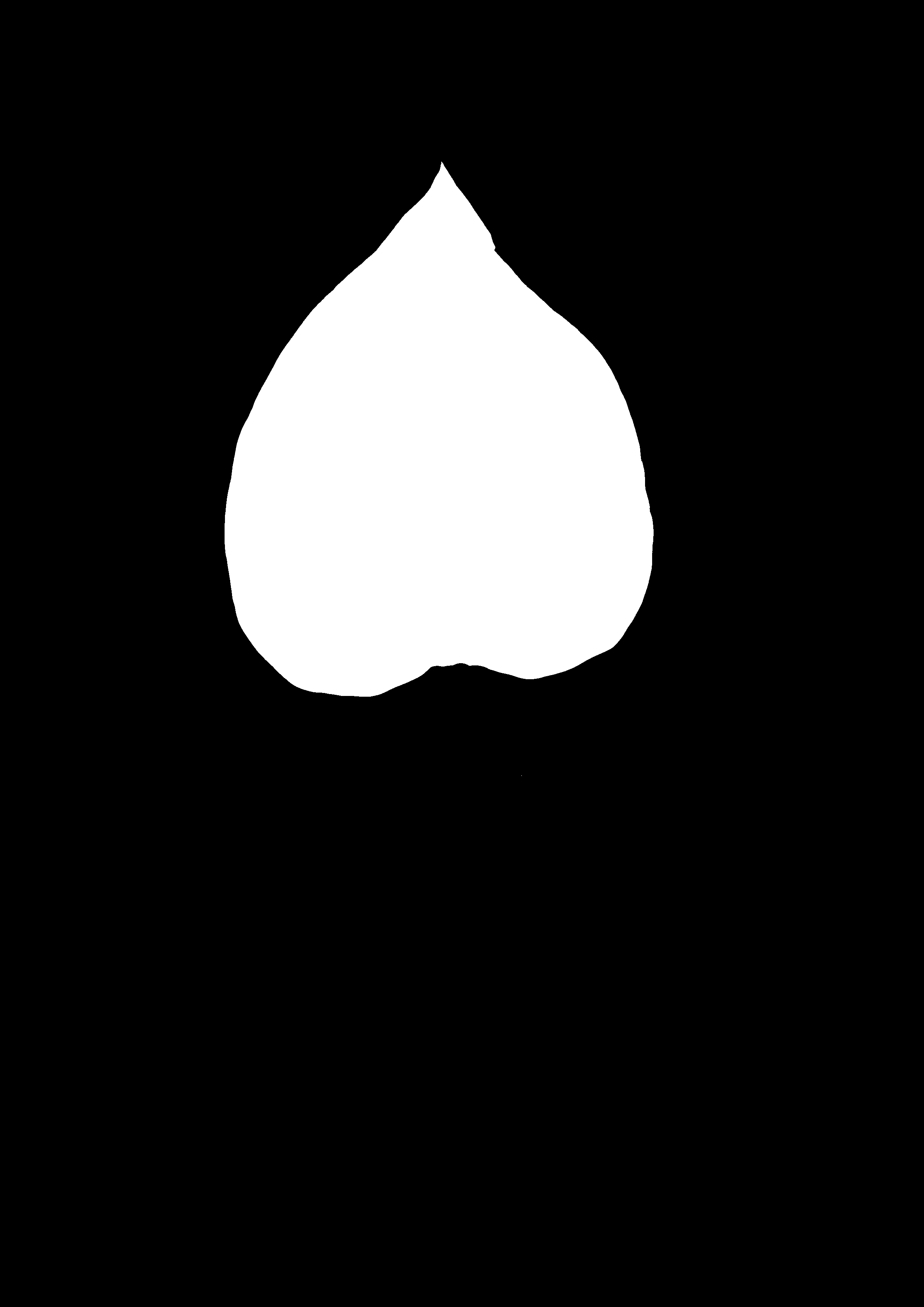

Supplement: Supplementary file 4 — Source Data [file 41467_2020_20730_MOESM4_ESM.zip › SourceData/Figure3_GlobalShapeComparison/Fig3_LeavesLabeled/Leaf13.png]

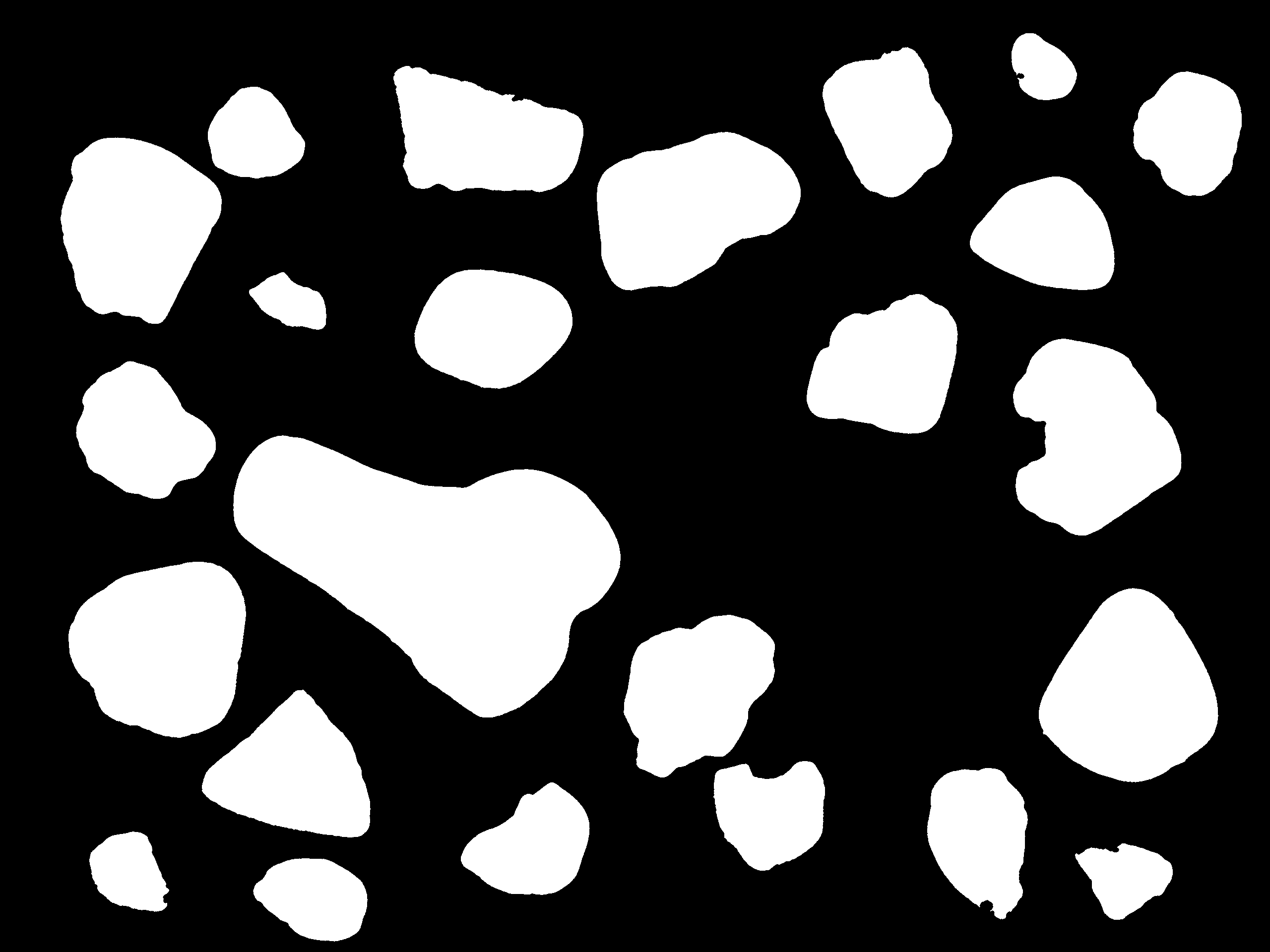

Supplement: Supplementary file 4 — Source Data [file 41467_2020_20730_MOESM4_ESM.zip › SourceData/Figure3_GlobalShapeComparison/Fig3_sandGrainsLabeled.png]

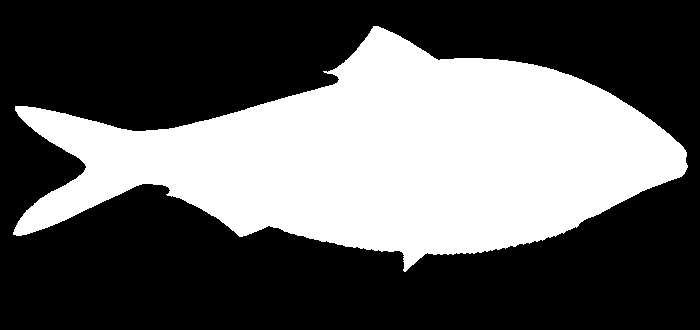

Supplement: Supplementary file 4 — Source Data [file 41467_2020_20730_MOESM4_ESM.zip › SourceData/Figure3_GlobalShapeComparison/Fig3_FishLabeled/Fish20.png]

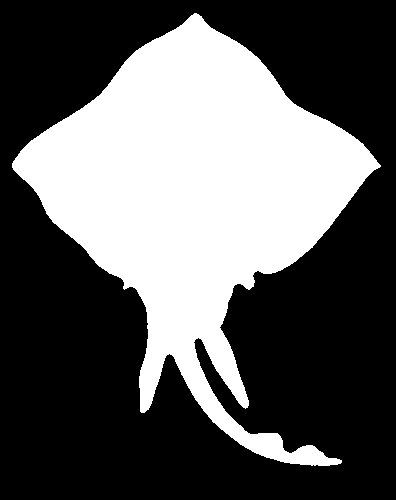

Supplement: Supplementary file 4 — Source Data [file 41467_2020_20730_MOESM4_ESM.zip › SourceData/Figure3_GlobalShapeComparison/Fig3_FishLabeled/Fish19.png]

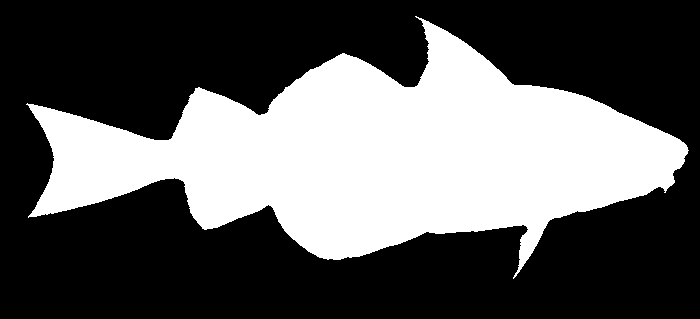

Supplement: Supplementary file 4 — Source Data [file 41467_2020_20730_MOESM4_ESM.zip › SourceData/Figure3_GlobalShapeComparison/Fig3_FishLabeled/Fish18.png]

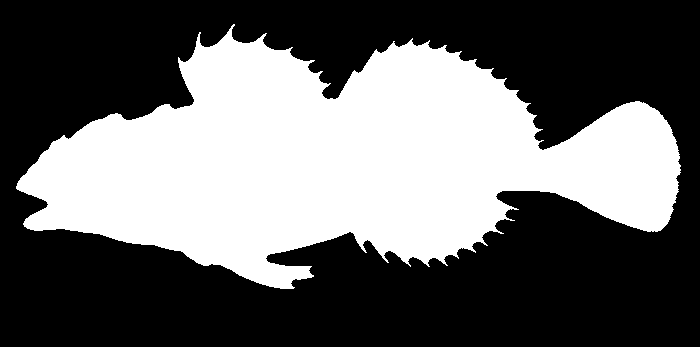

Supplement: Supplementary file 4 — Source Data [file 41467_2020_20730_MOESM4_ESM.zip › SourceData/Figure3_GlobalShapeComparison/Fig3_FishLabeled/Fish1.png]

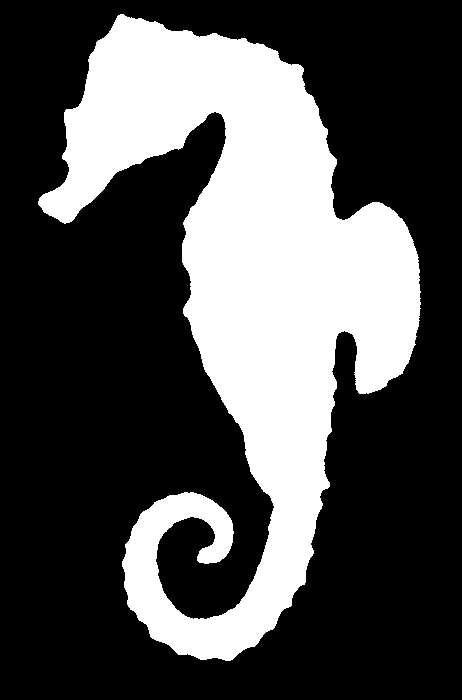

Supplement: Supplementary file 4 — Source Data [file 41467_2020_20730_MOESM4_ESM.zip › SourceData/Figure3_GlobalShapeComparison/Fig3_FishLabeled/Fish3.png]

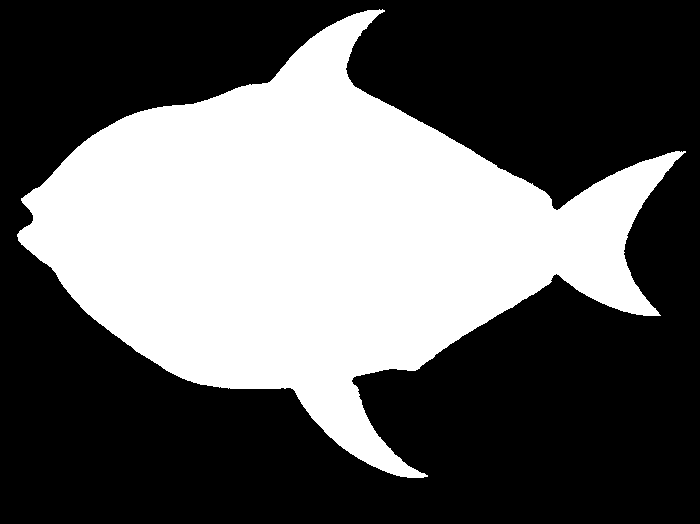

Supplement: Supplementary file 4 — Source Data [file 41467_2020_20730_MOESM4_ESM.zip › SourceData/Figure3_GlobalShapeComparison/Fig3_FishLabeled/Fish2.png]

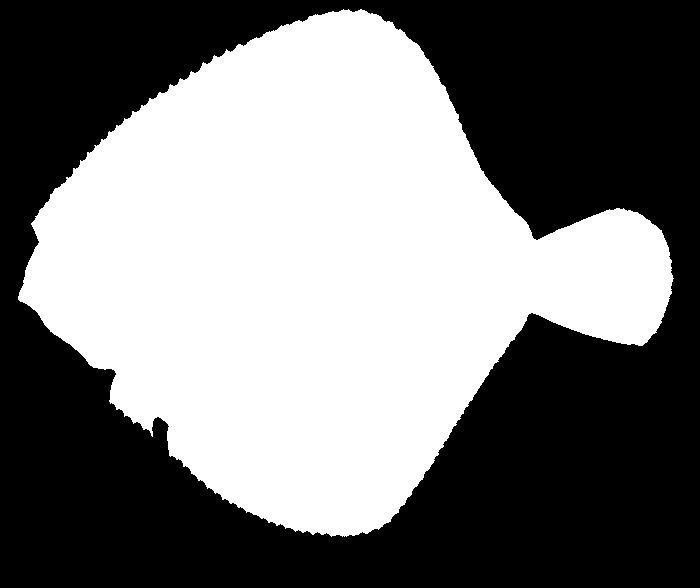

Supplement: Supplementary file 4 — Source Data [file 41467_2020_20730_MOESM4_ESM.zip › SourceData/Figure3_GlobalShapeComparison/Fig3_FishLabeled/Fish6.png]

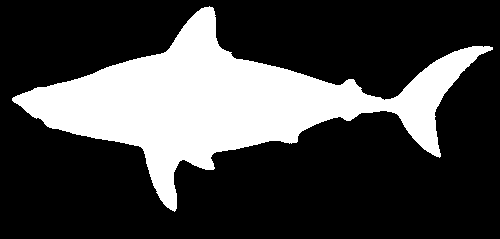

Supplement: Supplementary file 4 — Source Data [file 41467_2020_20730_MOESM4_ESM.zip › SourceData/Figure3_GlobalShapeComparison/Fig3_FishLabeled/Fish7.png]

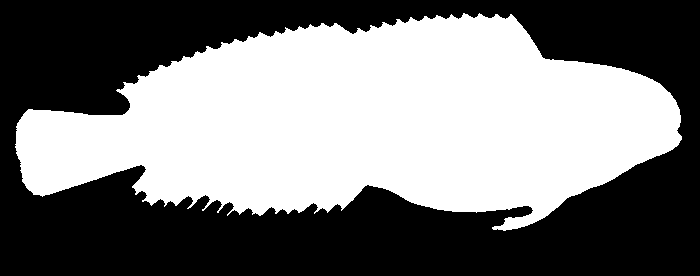

Supplement: Supplementary file 4 — Source Data [file 41467_2020_20730_MOESM4_ESM.zip › SourceData/Figure3_GlobalShapeComparison/Fig3_FishLabeled/Fish5.png]

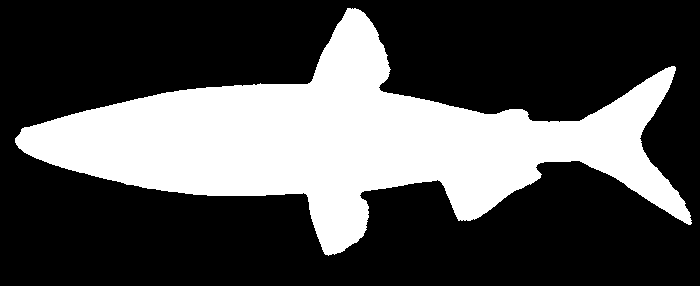

Supplement: Supplementary file 4 — Source Data [file 41467_2020_20730_MOESM4_ESM.zip › SourceData/Figure3_GlobalShapeComparison/Fig3_FishLabeled/Fish4.png]

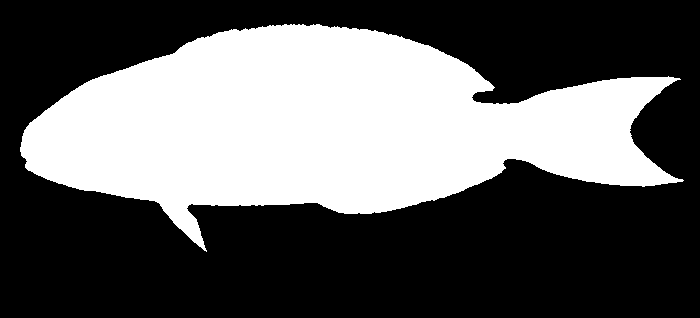

Supplement: Supplementary file 4 — Source Data [file 41467_2020_20730_MOESM4_ESM.zip › SourceData/Figure3_GlobalShapeComparison/Fig3_FishLabeled/Fish9.png]

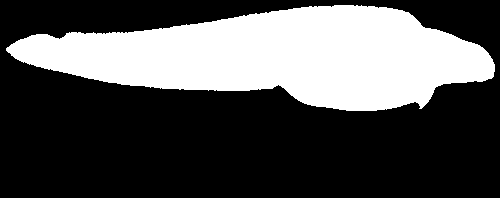

Supplement: Supplementary file 4 — Source Data [file 41467_2020_20730_MOESM4_ESM.zip › SourceData/Figure3_GlobalShapeComparison/Fig3_FishLabeled/Fish8.png]

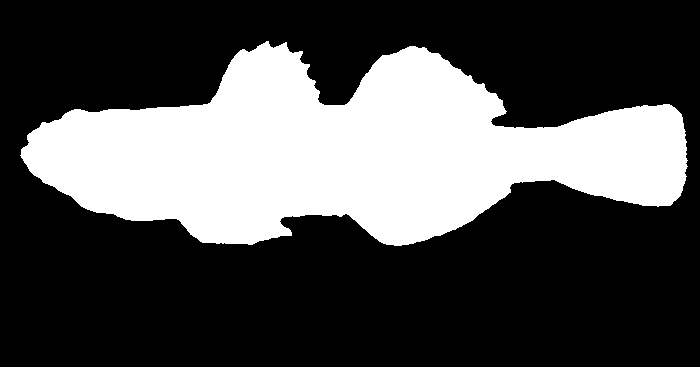

Supplement: Supplementary file 4 — Source Data [file 41467_2020_20730_MOESM4_ESM.zip › SourceData/Figure3_GlobalShapeComparison/Fig3_FishLabeled/Fish16.png]

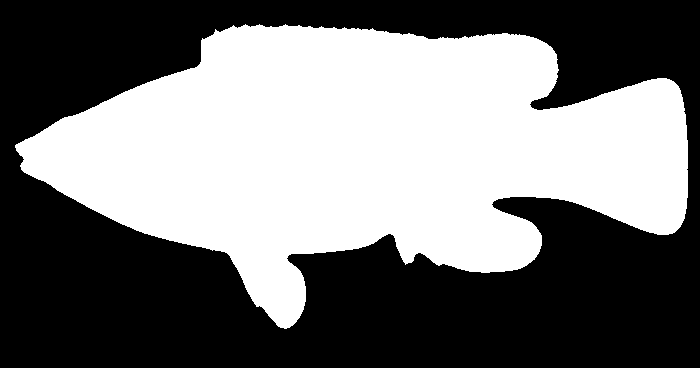

Supplement: Supplementary file 4 — Source Data [file 41467_2020_20730_MOESM4_ESM.zip › SourceData/Figure3_GlobalShapeComparison/Fig3_FishLabeled/Fish17.png]

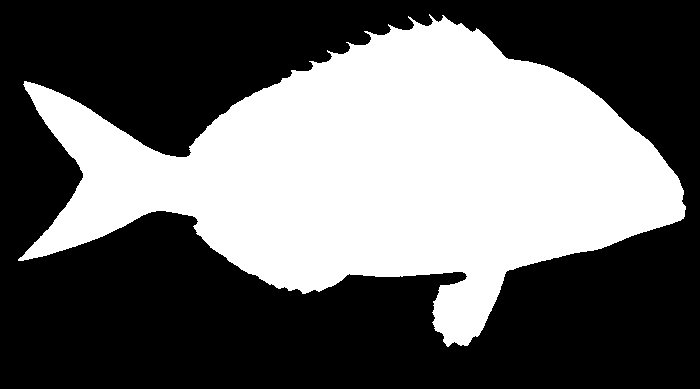

Supplement: Supplementary file 4 — Source Data [file 41467_2020_20730_MOESM4_ESM.zip › SourceData/Figure3_GlobalShapeComparison/Fig3_FishLabeled/Fish15.png]

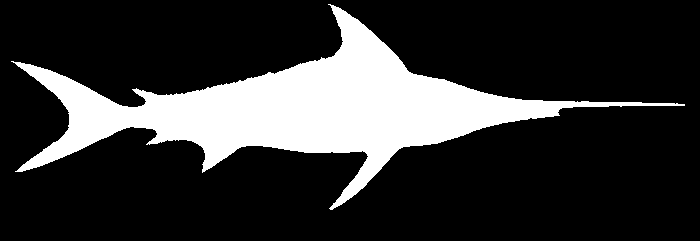

Supplement: Supplementary file 4 — Source Data [file 41467_2020_20730_MOESM4_ESM.zip › SourceData/Figure3_GlobalShapeComparison/Fig3_FishLabeled/Fish14.png]

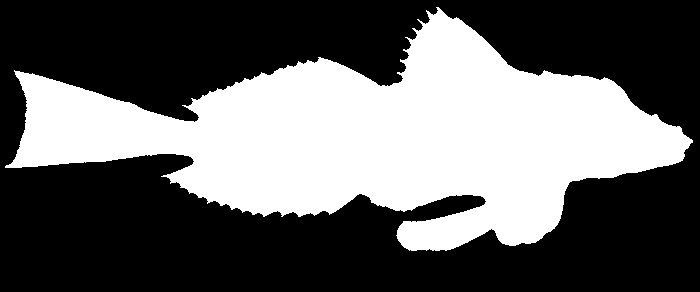

Supplement: Supplementary file 4 — Source Data [file 41467_2020_20730_MOESM4_ESM.zip › SourceData/Figure3_GlobalShapeComparison/Fig3_FishLabeled/Fish10.png]

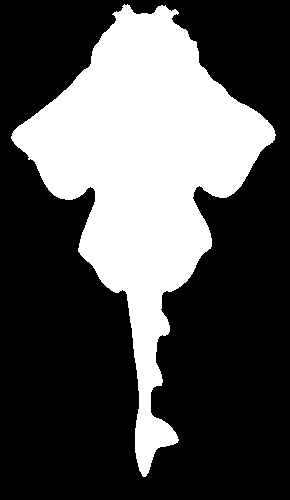

Supplement: Supplementary file 4 — Source Data [file 41467_2020_20730_MOESM4_ESM.zip › SourceData/Figure3_GlobalShapeComparison/Fig3_FishLabeled/Fish11.png]

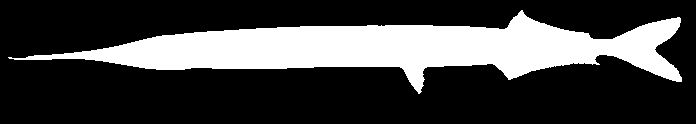

Supplement: Supplementary file 4 — Source Data [file 41467_2020_20730_MOESM4_ESM.zip › SourceData/Figure3_GlobalShapeComparison/Fig3_FishLabeled/Fish13.png]

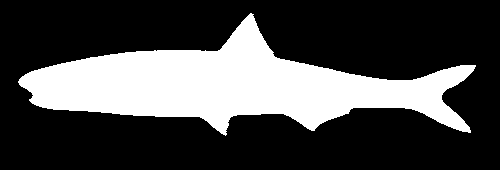

Supplement: Supplementary file 4 — Source Data [file 41467_2020_20730_MOESM4_ESM.zip › SourceData/Figure3_GlobalShapeComparison/Fig3_FishLabeled/Fish12.png]
