# Supplementary figures and images for: A network-based framework for shape analysis enables accurate characterization of leaf epidermal cells (part 1 of 2)
Source: Nat Commun. 2021 Jan 19;12:458. doi: 10.1038/s41467-020-20730-y (PMC7815848; doi:10.1038/s41467-020-20730-y)

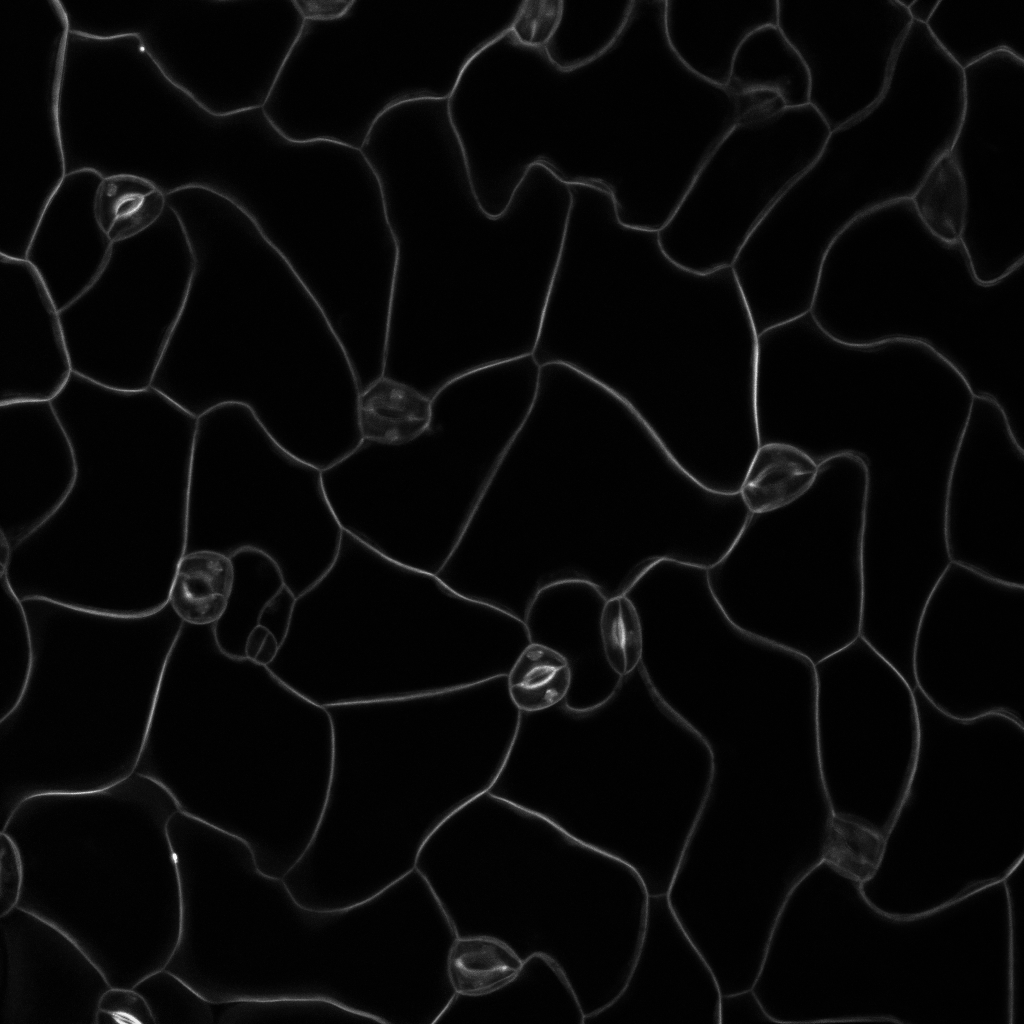

Supplement: Supplementary file 4 — Source Data [file 41467_2020_20730_MOESM4_ESM.zip › SourceData/Figure7_ShapeMutants/Images/RIC1-OX/RIC1-OX_S4.tif]

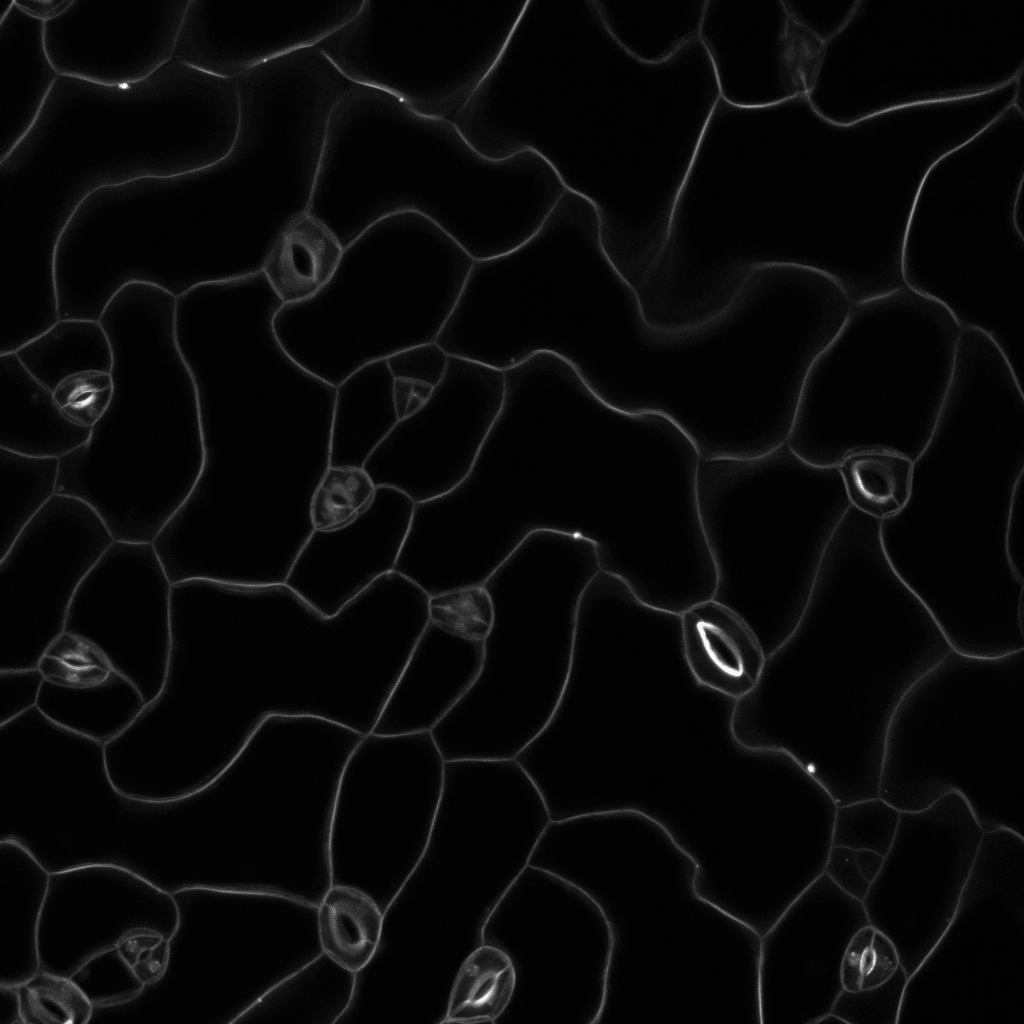

Supplement: Supplementary file 4 — Source Data [file 41467_2020_20730_MOESM4_ESM.zip › SourceData/Figure7_ShapeMutants/Images/RIC1-OX/RIC1-OX_S5.tif]

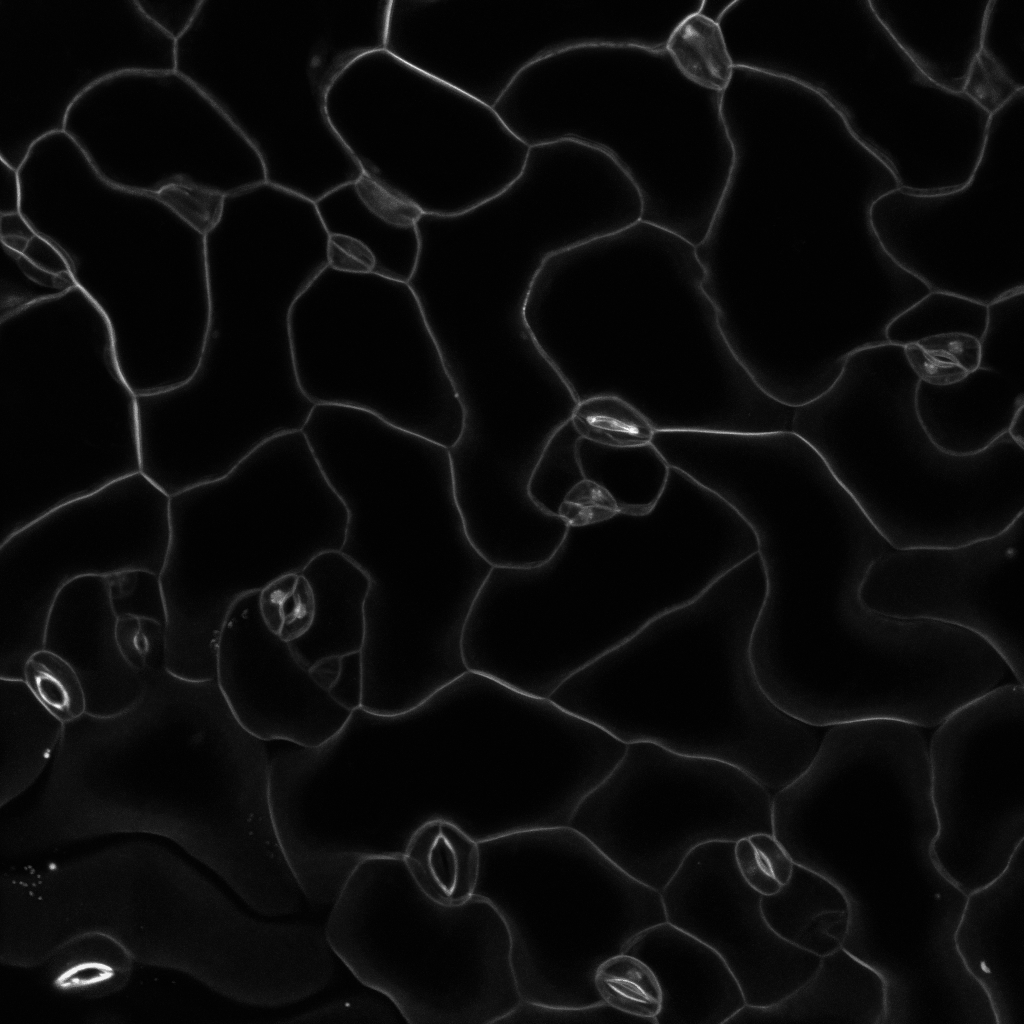

Supplement: Supplementary file 4 — Source Data [file 41467_2020_20730_MOESM4_ESM.zip › SourceData/Figure7_ShapeMutants/Images/RIC1-OX/RIC1-OX_S6.tif]

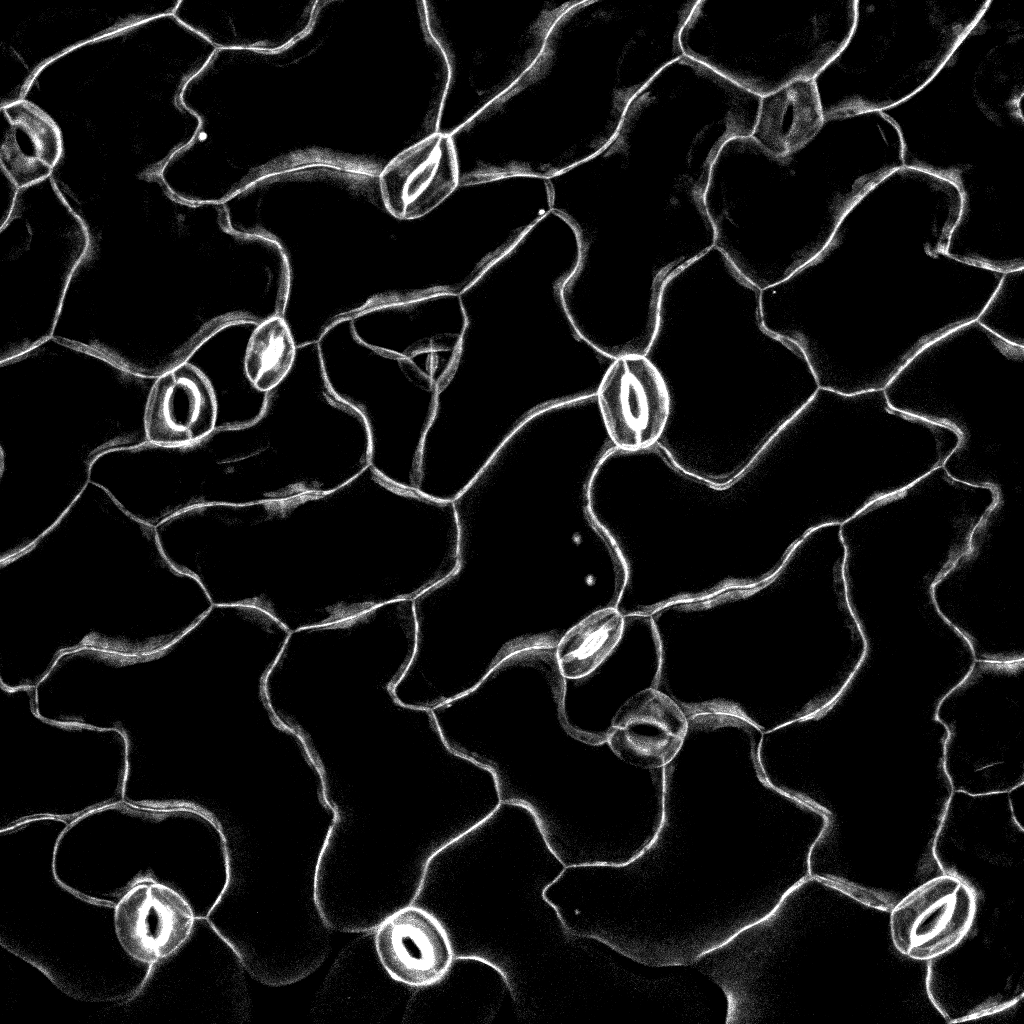

Supplement: Supplementary file 4 — Source Data [file 41467_2020_20730_MOESM4_ESM.zip › SourceData/Figure7_ShapeMutants/Images/RIC1-OX/RIC1-OX_S2.tif]

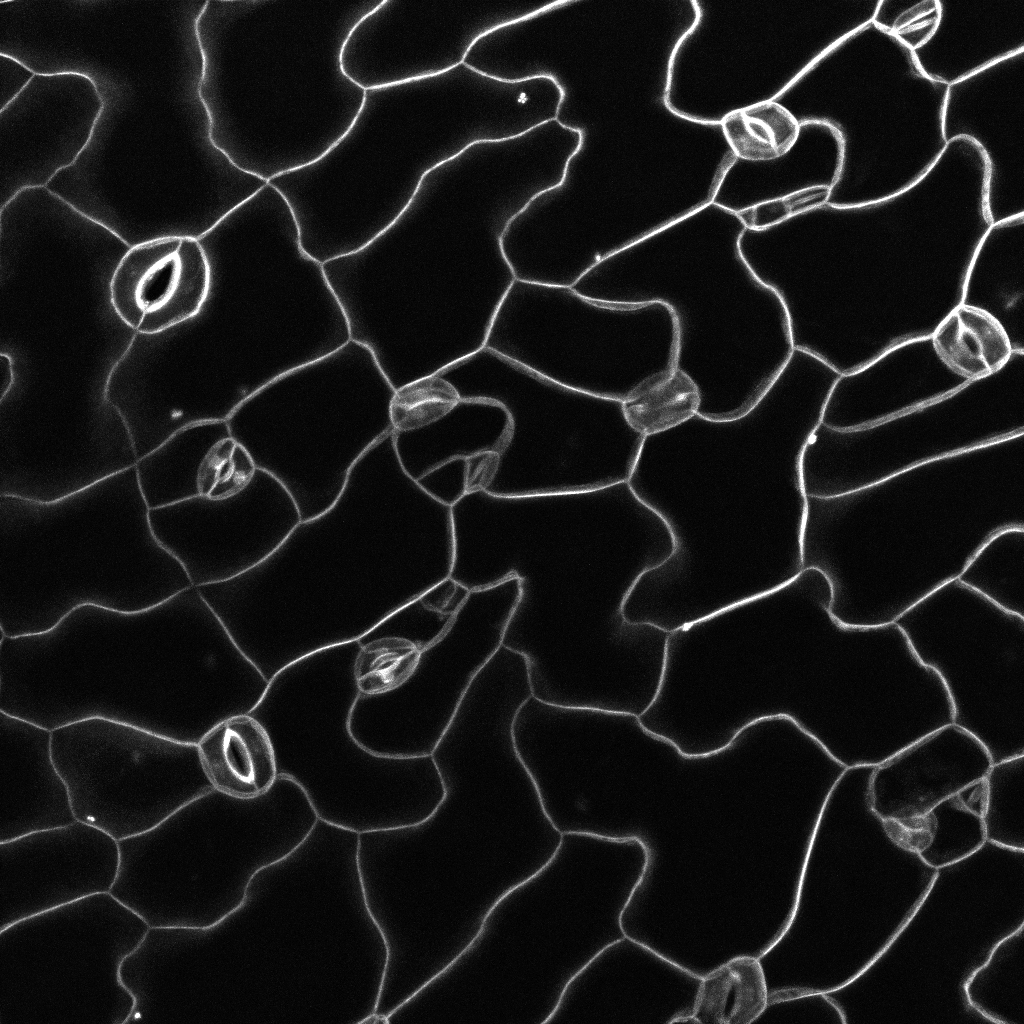

Supplement: Supplementary file 4 — Source Data [file 41467_2020_20730_MOESM4_ESM.zip › SourceData/Figure7_ShapeMutants/Images/RIC1-OX/RIC1-OX_S3.tif]

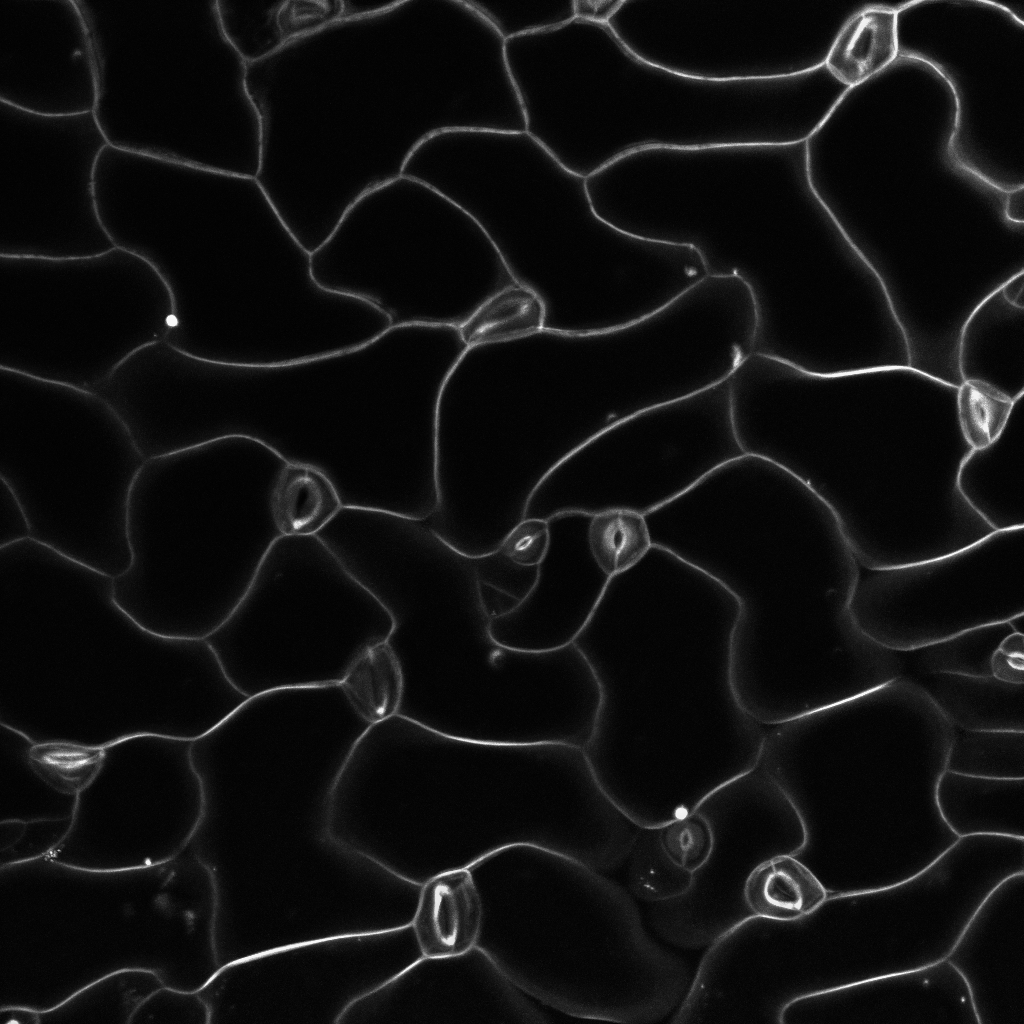

Supplement: Supplementary file 4 — Source Data [file 41467_2020_20730_MOESM4_ESM.zip › SourceData/Figure7_ShapeMutants/Images/RIC1-OX/RIC1-OX_S1.tif]

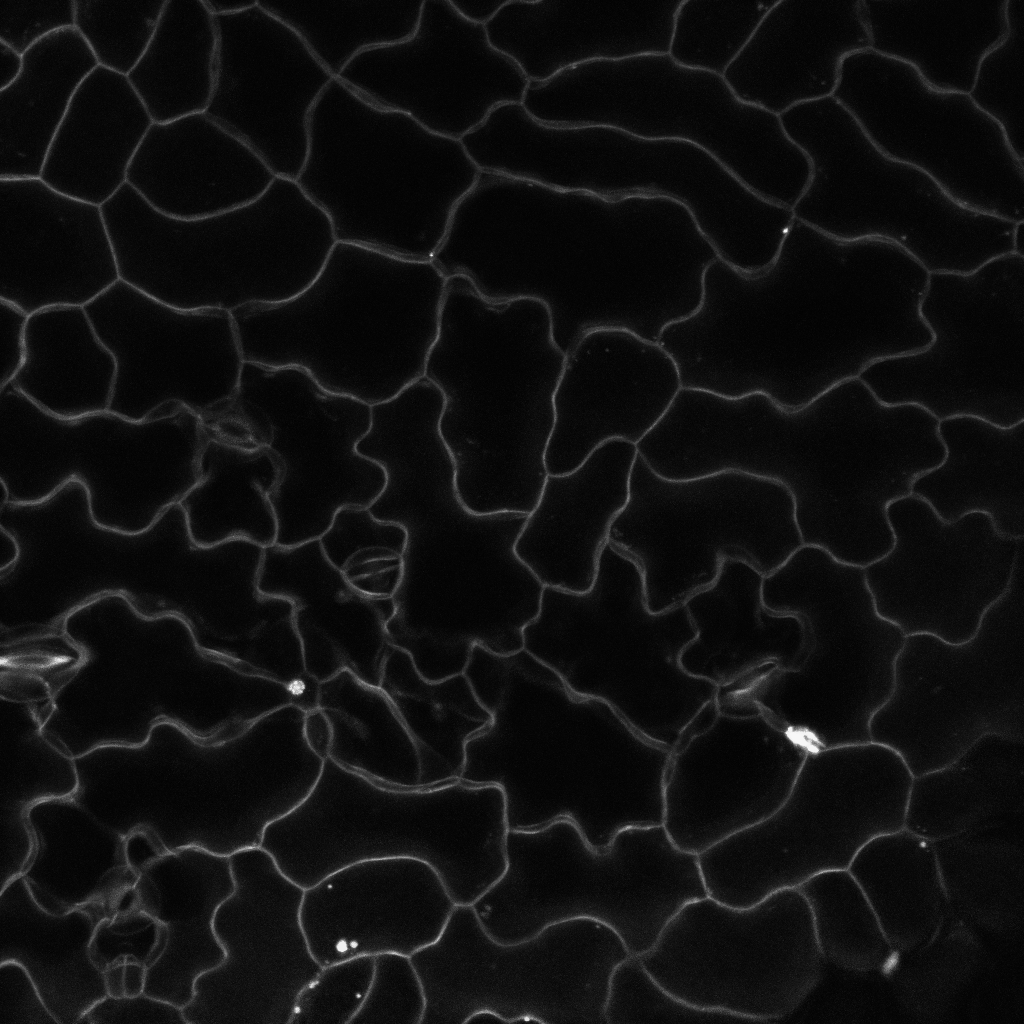

Supplement: Supplementary file 4 — Source Data [file 41467_2020_20730_MOESM4_ESM.zip › SourceData/Figure7_ShapeMutants/Images/dek1-4/dek1-4_S5.tif]

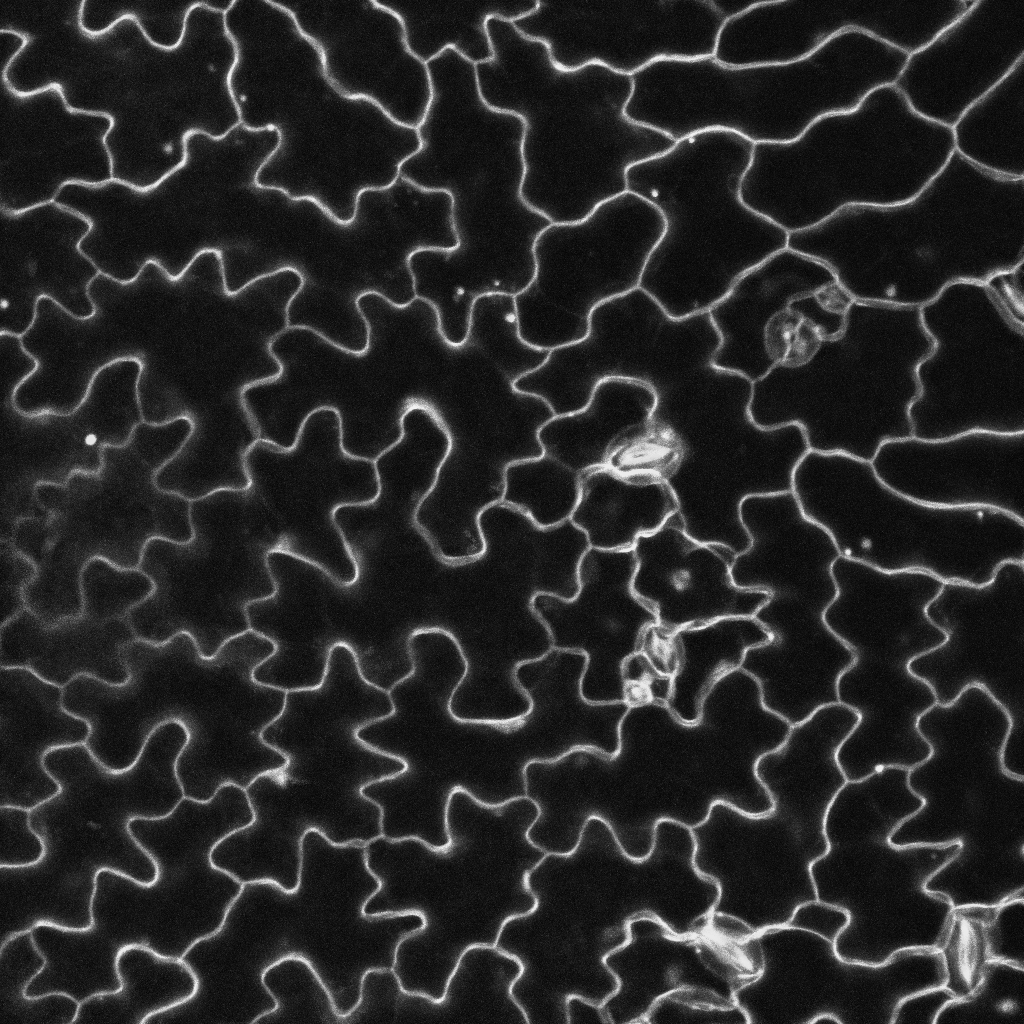

Supplement: Supplementary file 4 — Source Data [file 41467_2020_20730_MOESM4_ESM.zip › SourceData/Figure7_ShapeMutants/Images/dek1-4/dek1-4_S4.tif]

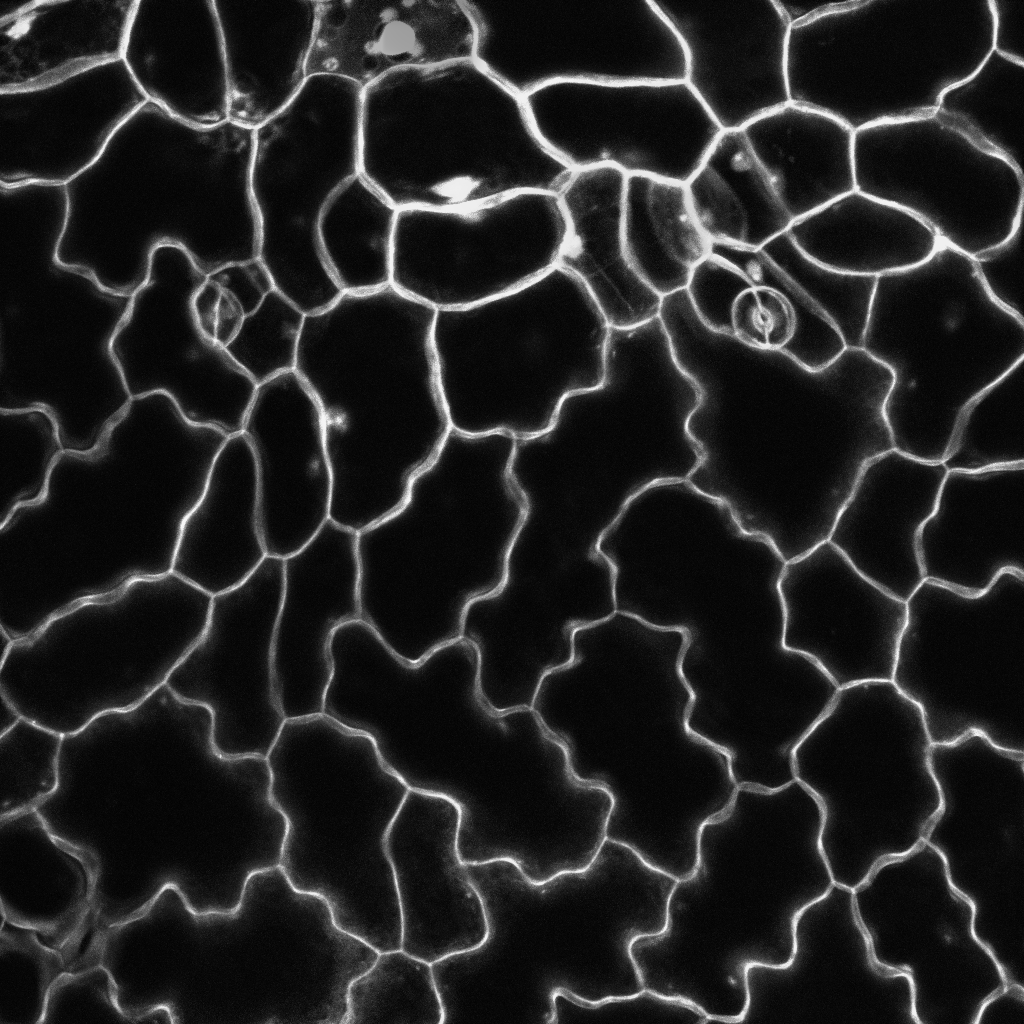

Supplement: Supplementary file 4 — Source Data [file 41467_2020_20730_MOESM4_ESM.zip › SourceData/Figure7_ShapeMutants/Images/dek1-4/dek1-4_S6.tif]

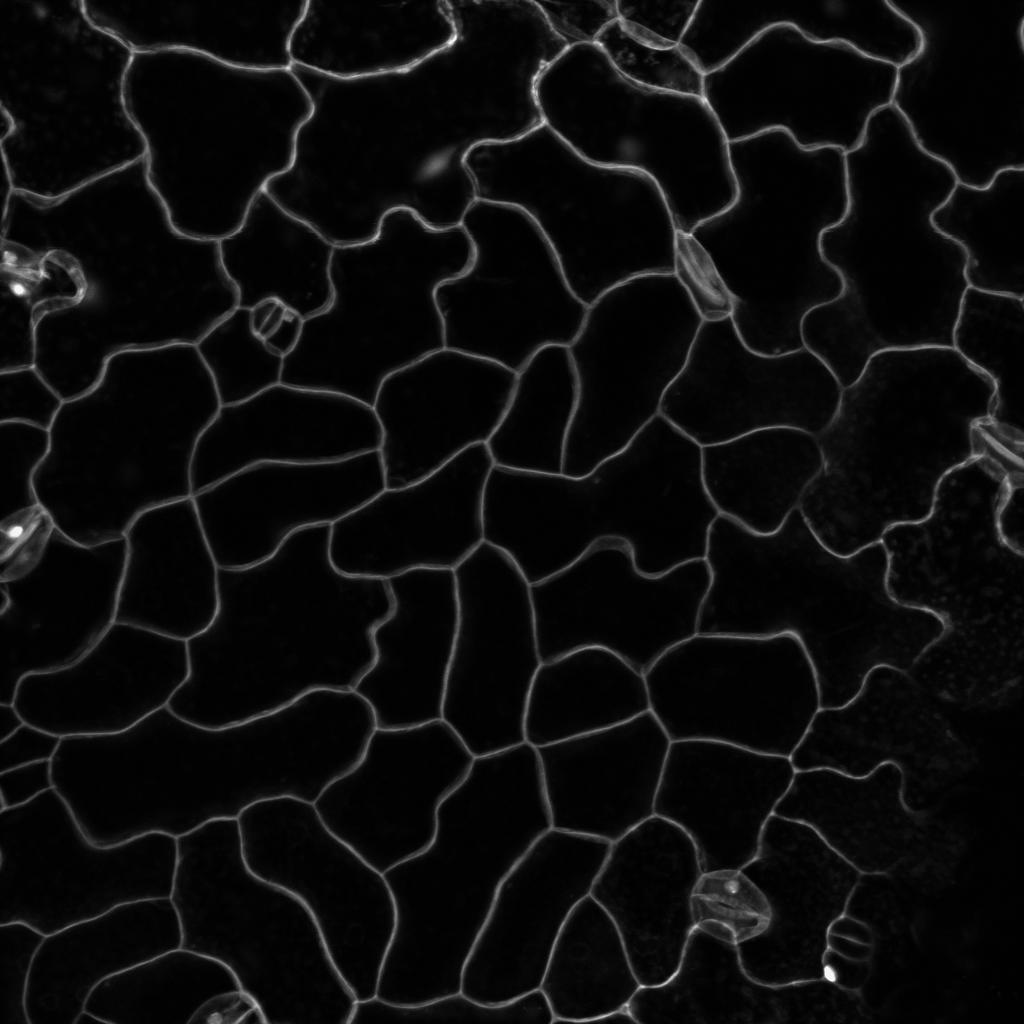

Supplement: Supplementary file 4 — Source Data [file 41467_2020_20730_MOESM4_ESM.zip › SourceData/Figure7_ShapeMutants/Images/dek1-4/dek1-4_S7.tif]

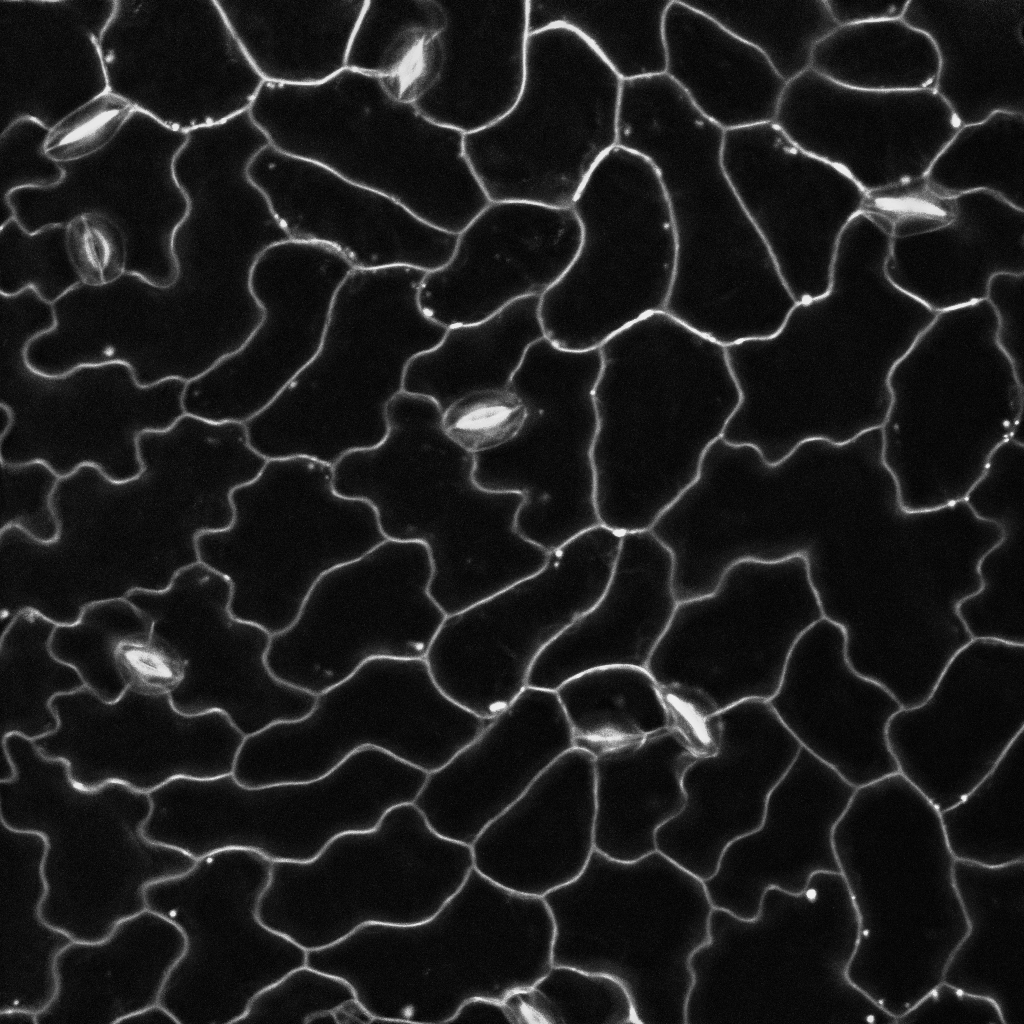

Supplement: Supplementary file 4 — Source Data [file 41467_2020_20730_MOESM4_ESM.zip › SourceData/Figure7_ShapeMutants/Images/dek1-4/dek1-4_S3.tif]

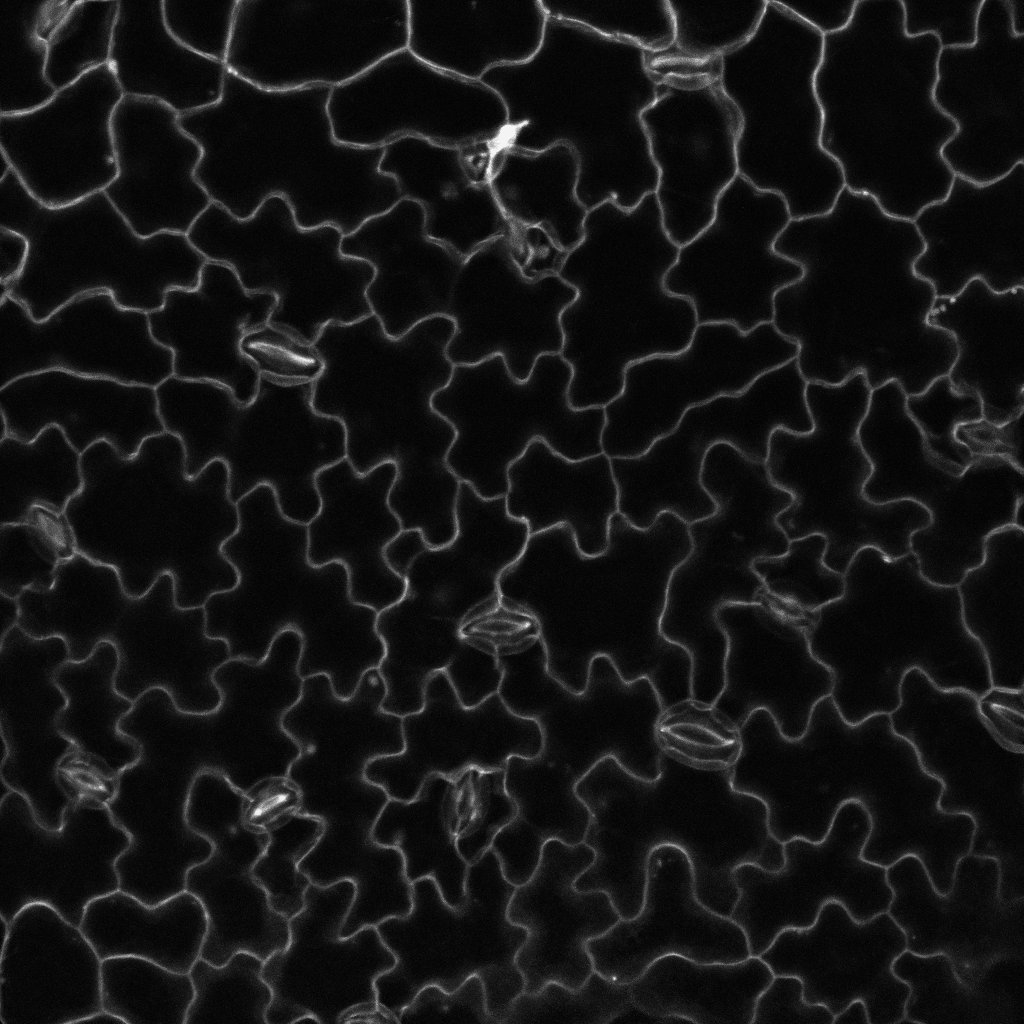

Supplement: Supplementary file 4 — Source Data [file 41467_2020_20730_MOESM4_ESM.zip › SourceData/Figure7_ShapeMutants/Images/dek1-4/dek1-4_S2.tif]

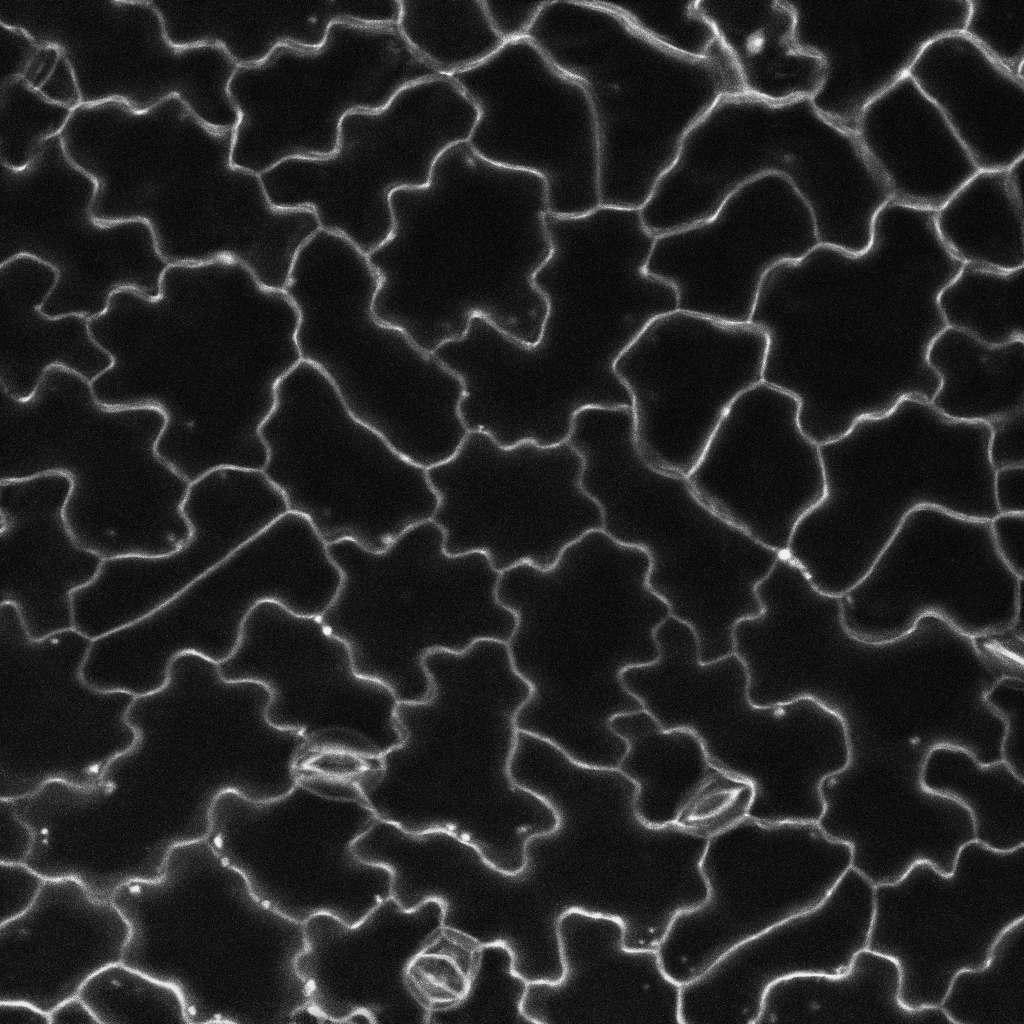

Supplement: Supplementary file 4 — Source Data [file 41467_2020_20730_MOESM4_ESM.zip › SourceData/Figure7_ShapeMutants/Images/dek1-4/dek1-4_S1.tif]

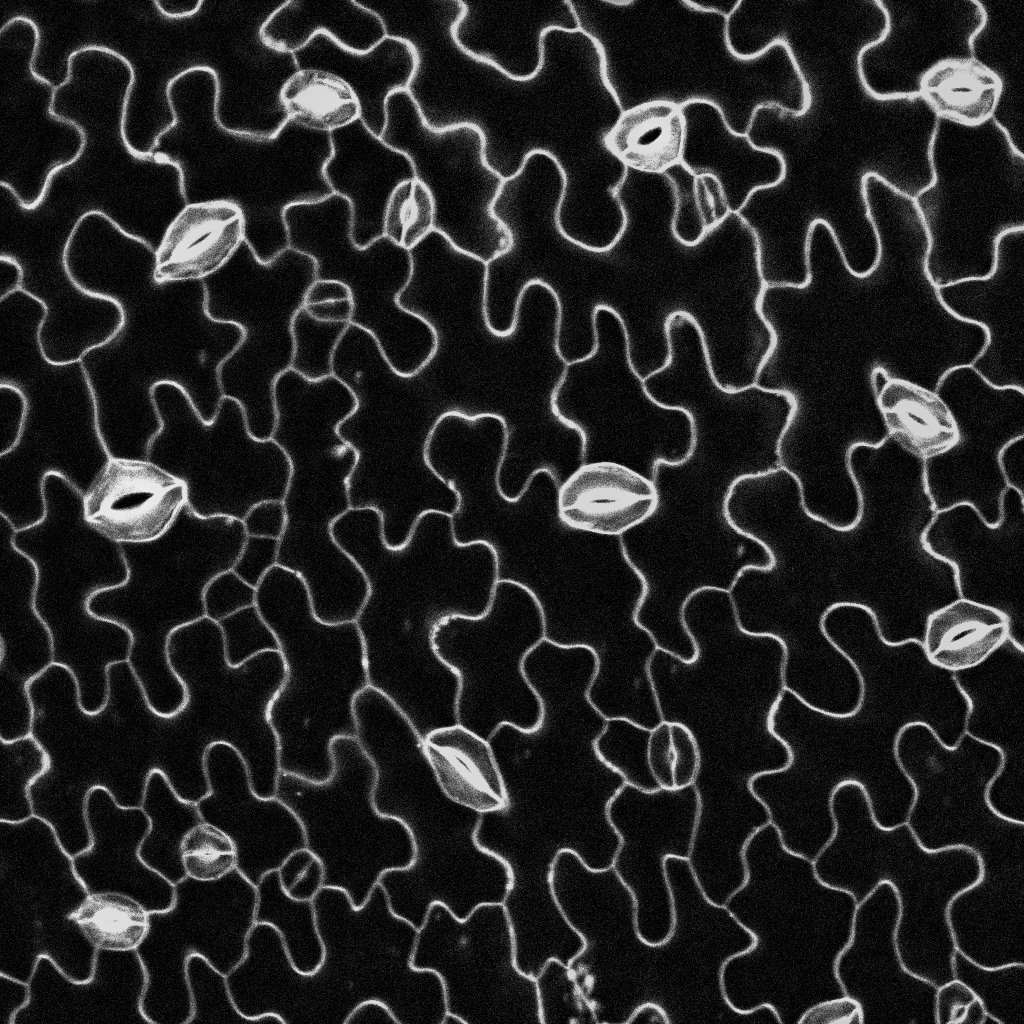

Supplement: Supplementary file 4 — Source Data [file 41467_2020_20730_MOESM4_ESM.zip › SourceData/Figure7_ShapeMutants/Images/ric1-1/ric1-1_S2.tif]

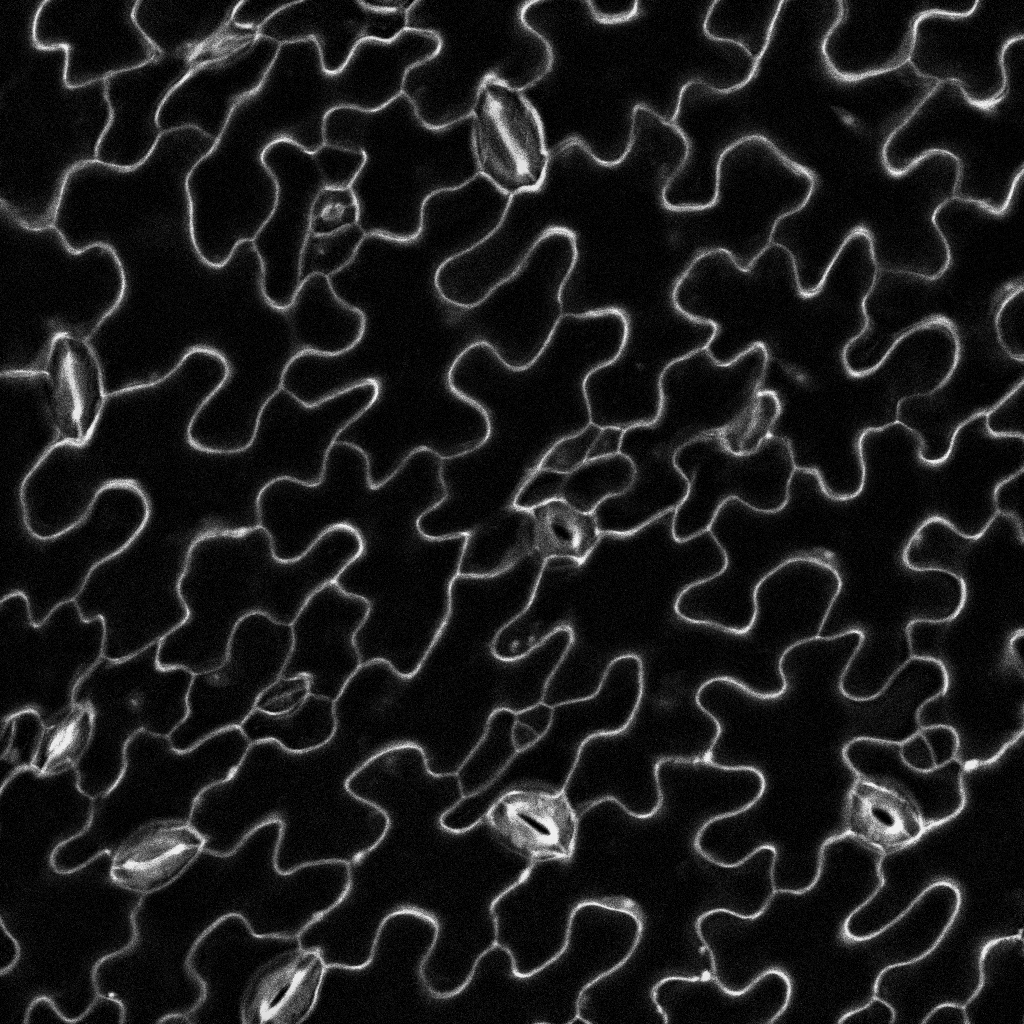

Supplement: Supplementary file 4 — Source Data [file 41467_2020_20730_MOESM4_ESM.zip › SourceData/Figure7_ShapeMutants/Images/ric1-1/ric1-1_S3.tif]

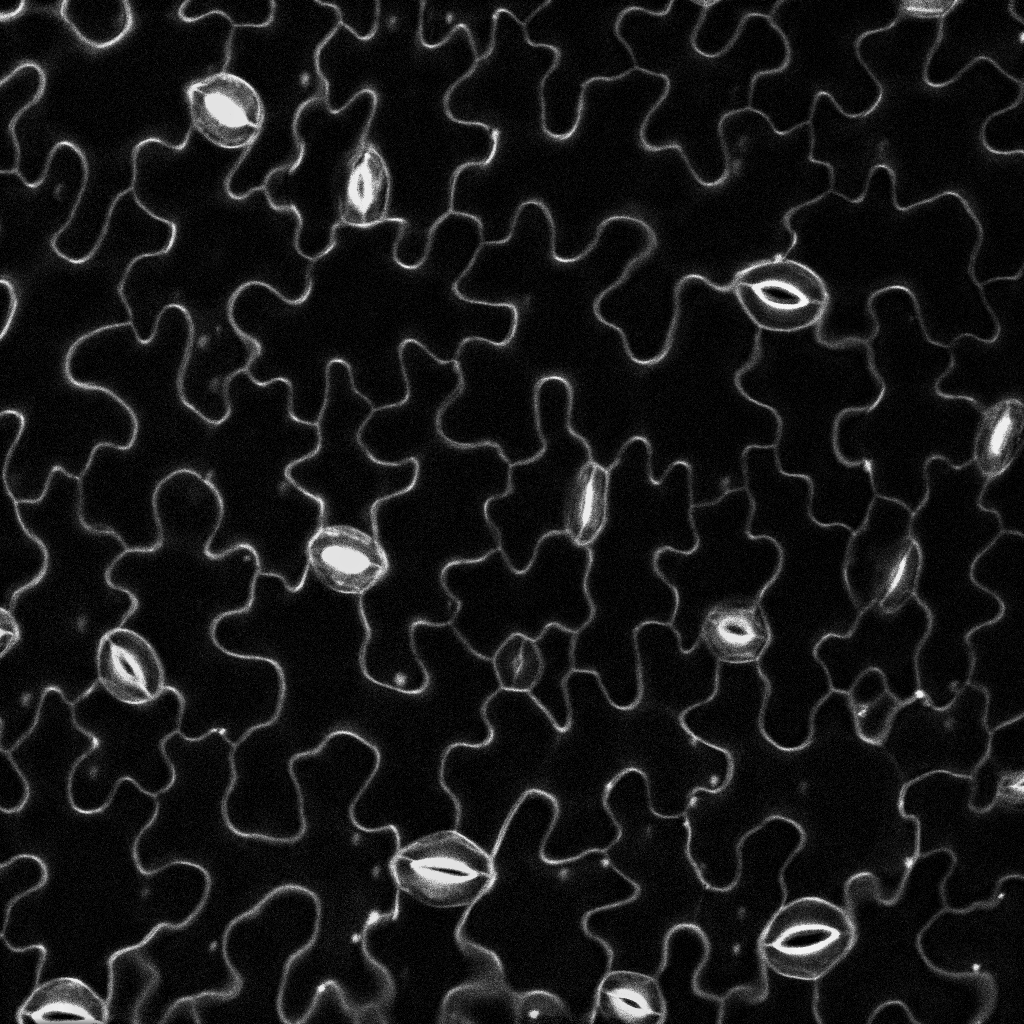

Supplement: Supplementary file 4 — Source Data [file 41467_2020_20730_MOESM4_ESM.zip › SourceData/Figure7_ShapeMutants/Images/ric1-1/ric1-1_S1.tif]

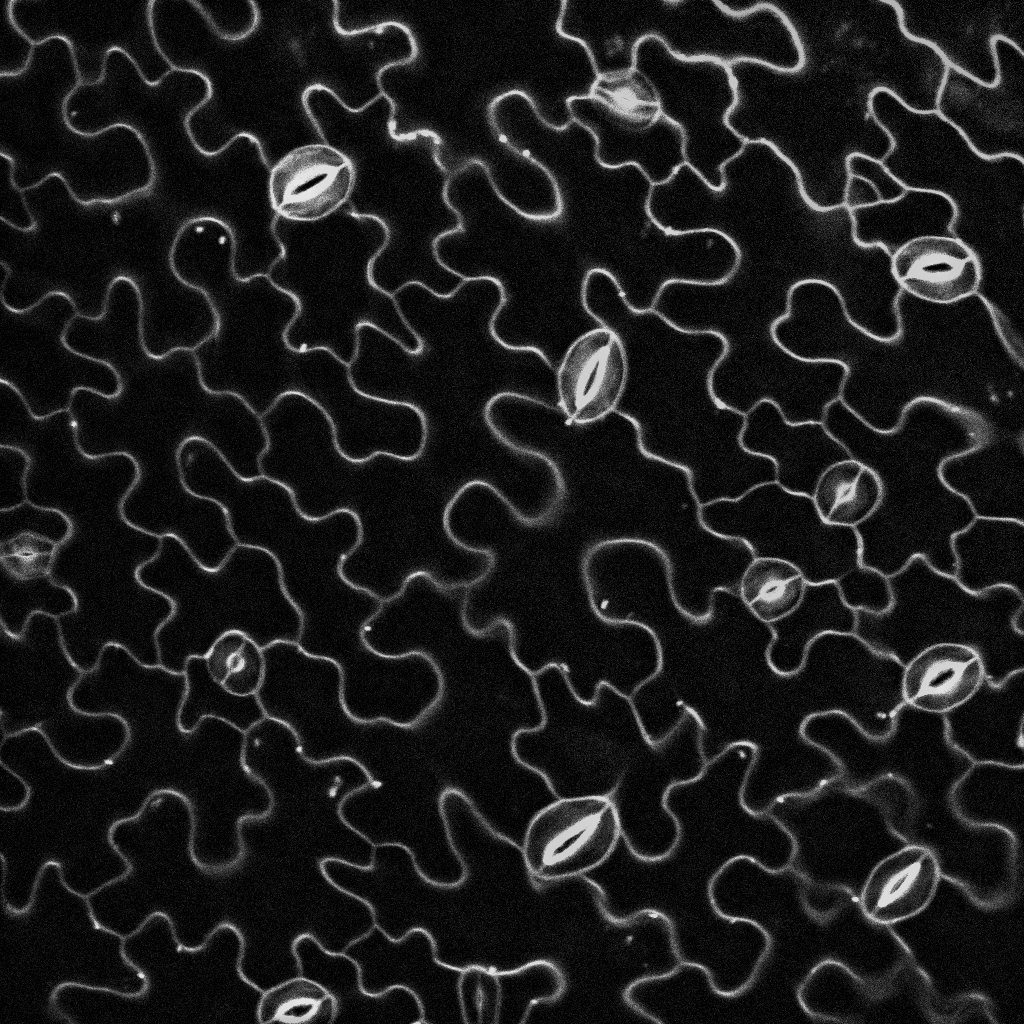

Supplement: Supplementary file 4 — Source Data [file 41467_2020_20730_MOESM4_ESM.zip › SourceData/Figure7_ShapeMutants/Images/ric1-1/ric1-1_S4.tif]

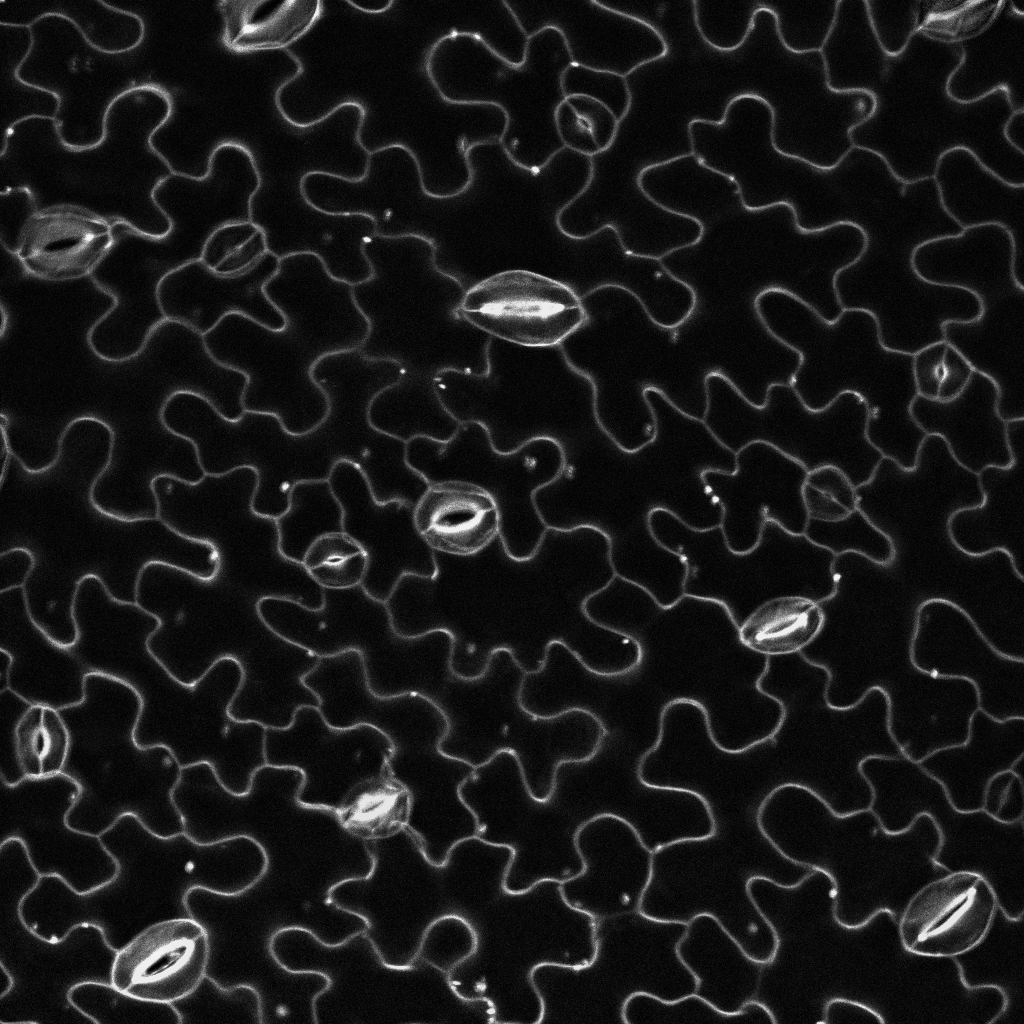

Supplement: Supplementary file 4 — Source Data [file 41467_2020_20730_MOESM4_ESM.zip › SourceData/Figure7_ShapeMutants/Images/ric1-1/ric1-1_S5.tif]

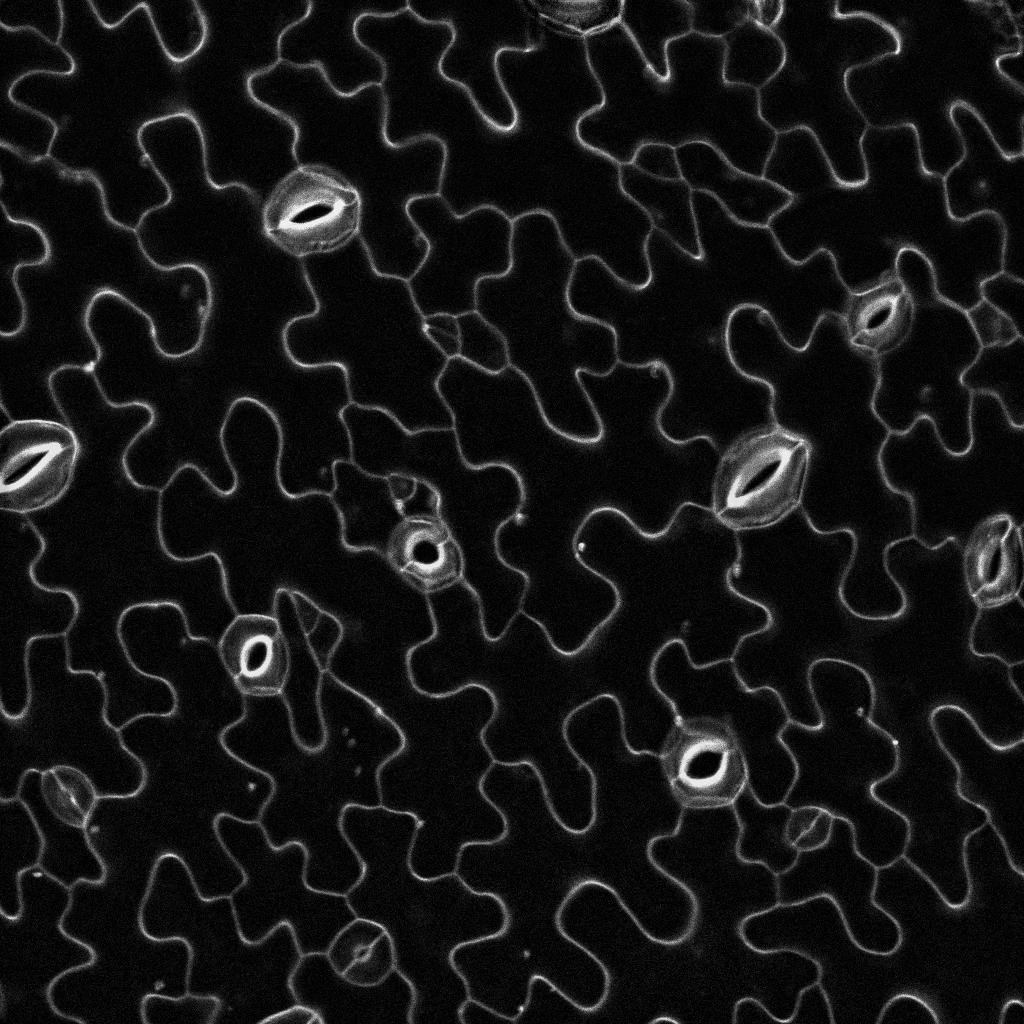

Supplement: Supplementary file 4 — Source Data [file 41467_2020_20730_MOESM4_ESM.zip › SourceData/Figure7_ShapeMutants/Images/ric1-1/ric1-1_S6.tif]

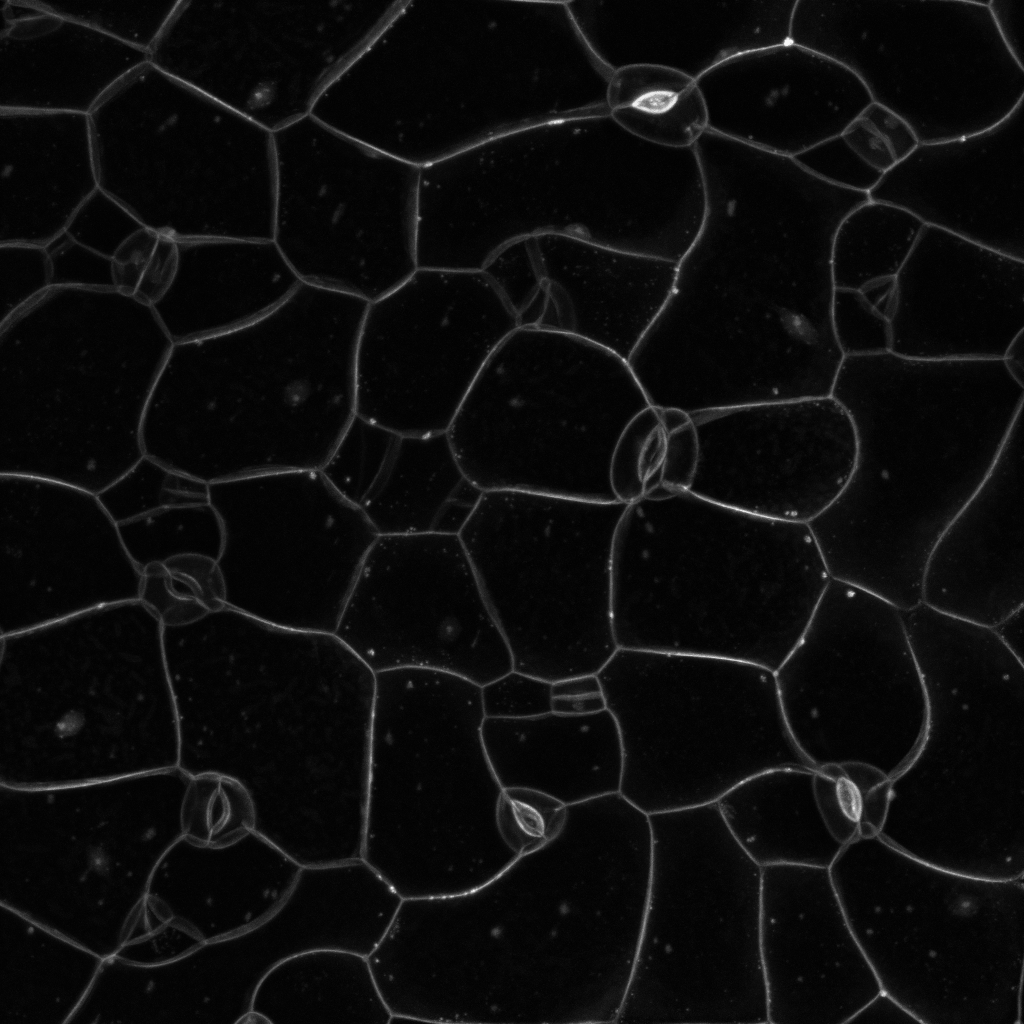

Supplement: Supplementary file 4 — Source Data [file 41467_2020_20730_MOESM4_ESM.zip › SourceData/Figure7_ShapeMutants/Images/CA-ROP2/CA-ROP2_S4.tif]

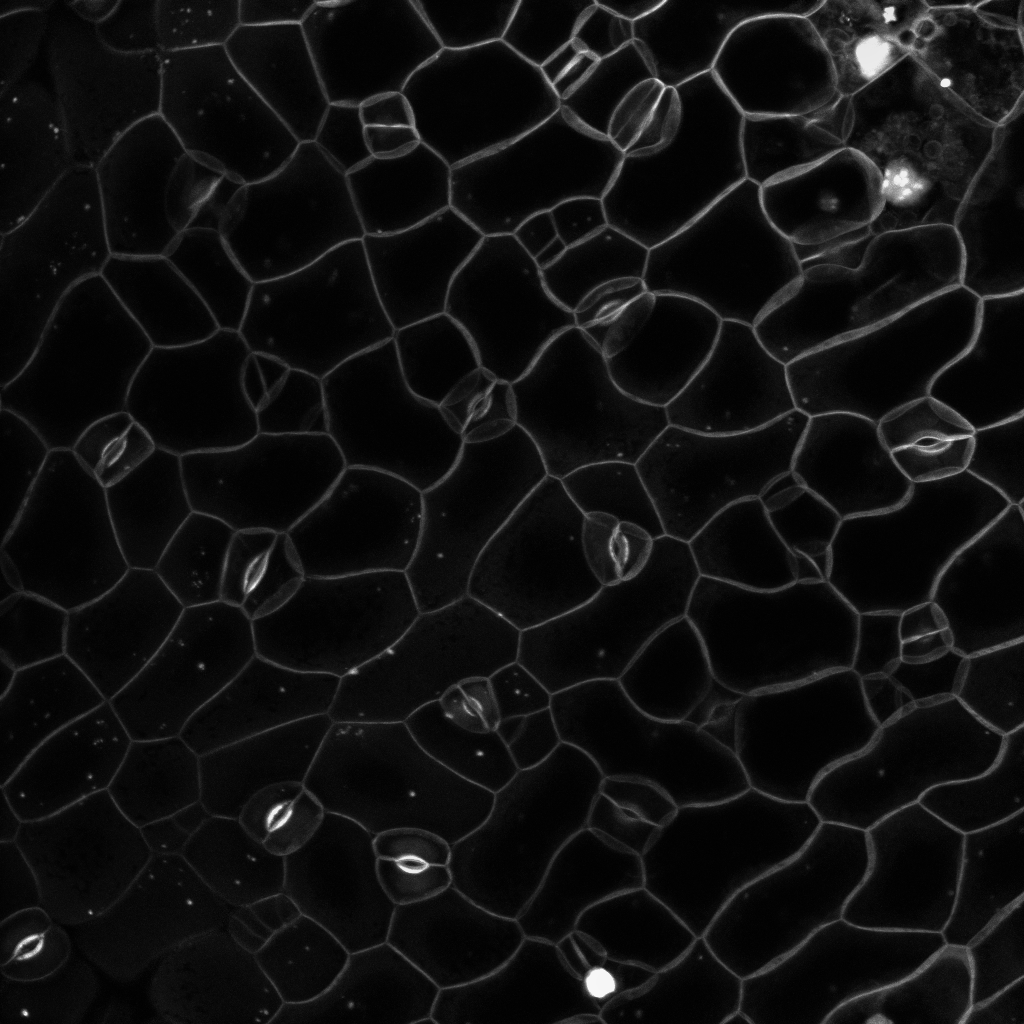

Supplement: Supplementary file 4 — Source Data [file 41467_2020_20730_MOESM4_ESM.zip › SourceData/Figure7_ShapeMutants/Images/CA-ROP2/CA-ROP2_S5.tif]

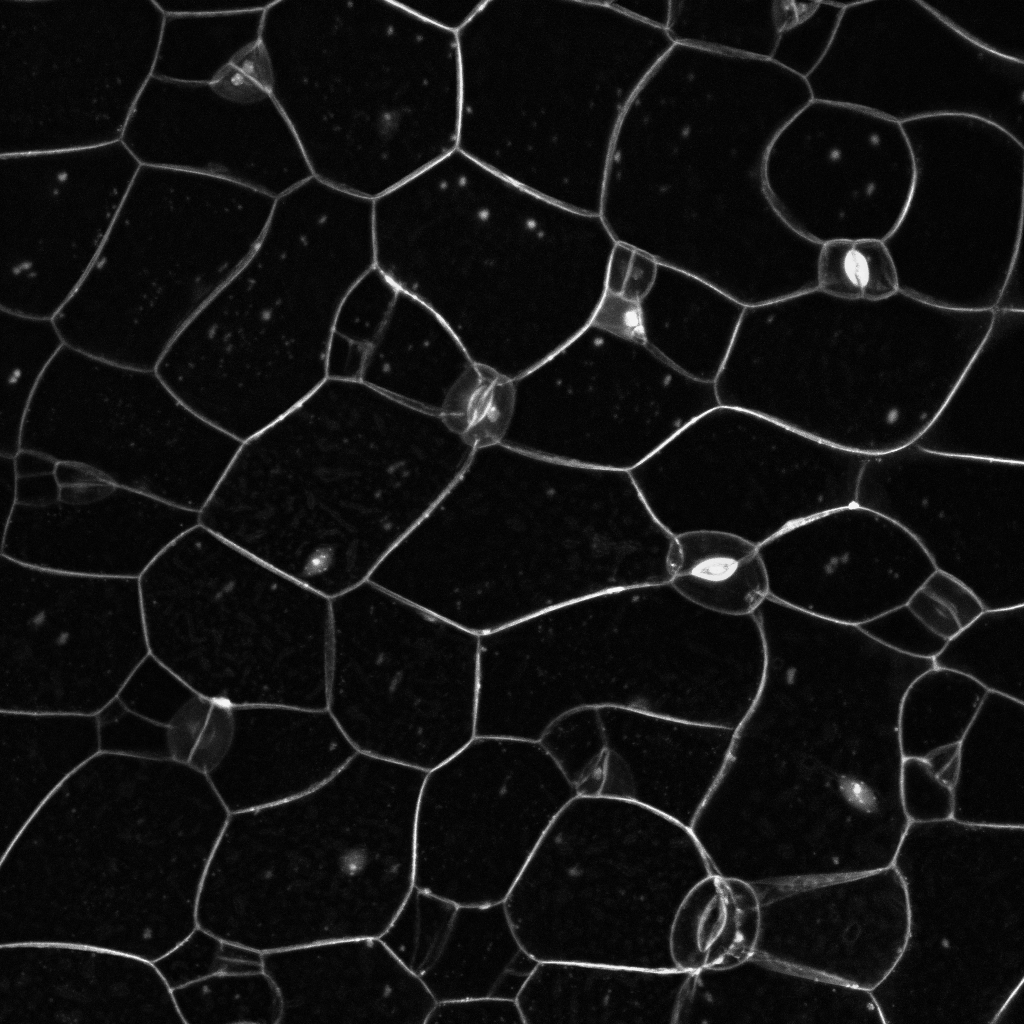

Supplement: Supplementary file 4 — Source Data [file 41467_2020_20730_MOESM4_ESM.zip › SourceData/Figure7_ShapeMutants/Images/CA-ROP2/CA-ROP2_S6.tif]

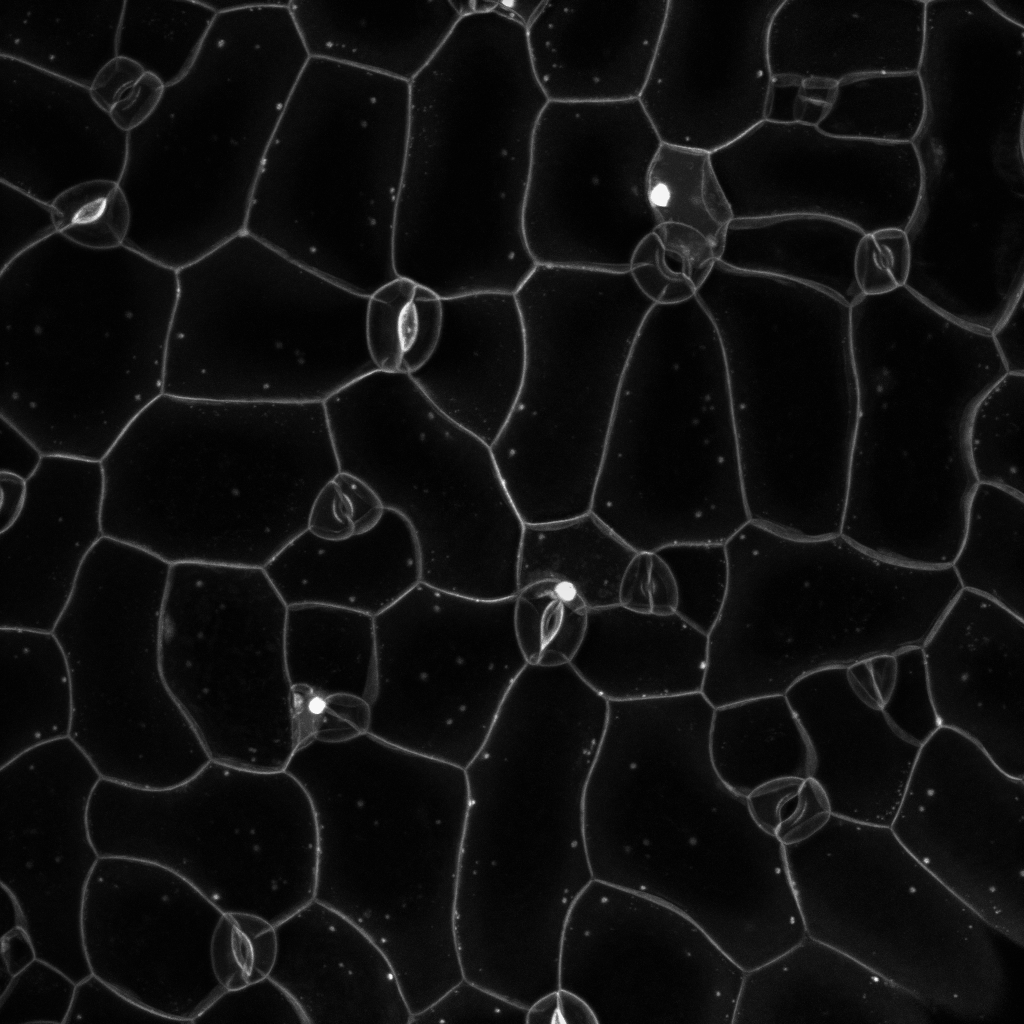

Supplement: Supplementary file 4 — Source Data [file 41467_2020_20730_MOESM4_ESM.zip › SourceData/Figure7_ShapeMutants/Images/CA-ROP2/CA-ROP2_S2.tif]

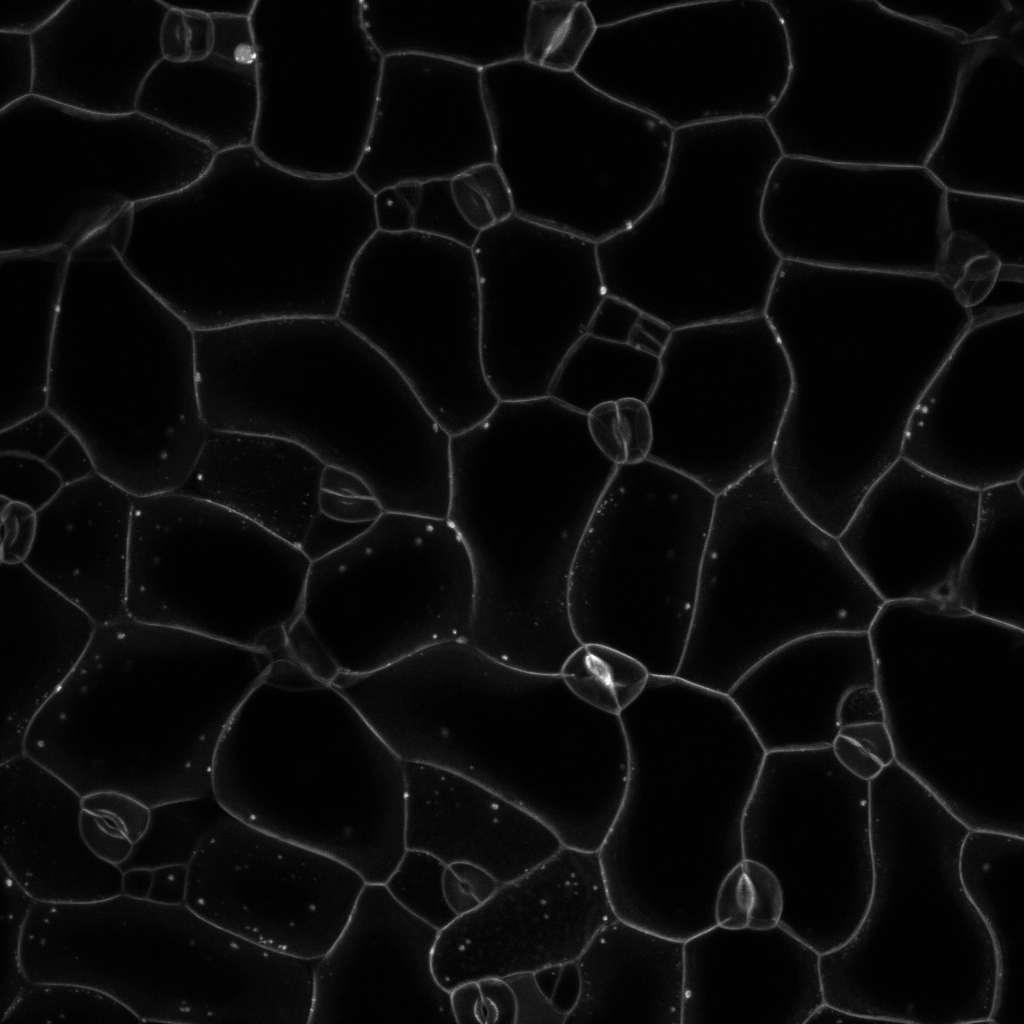

Supplement: Supplementary file 4 — Source Data [file 41467_2020_20730_MOESM4_ESM.zip › SourceData/Figure7_ShapeMutants/Images/CA-ROP2/CA-ROP2_S3.tif]

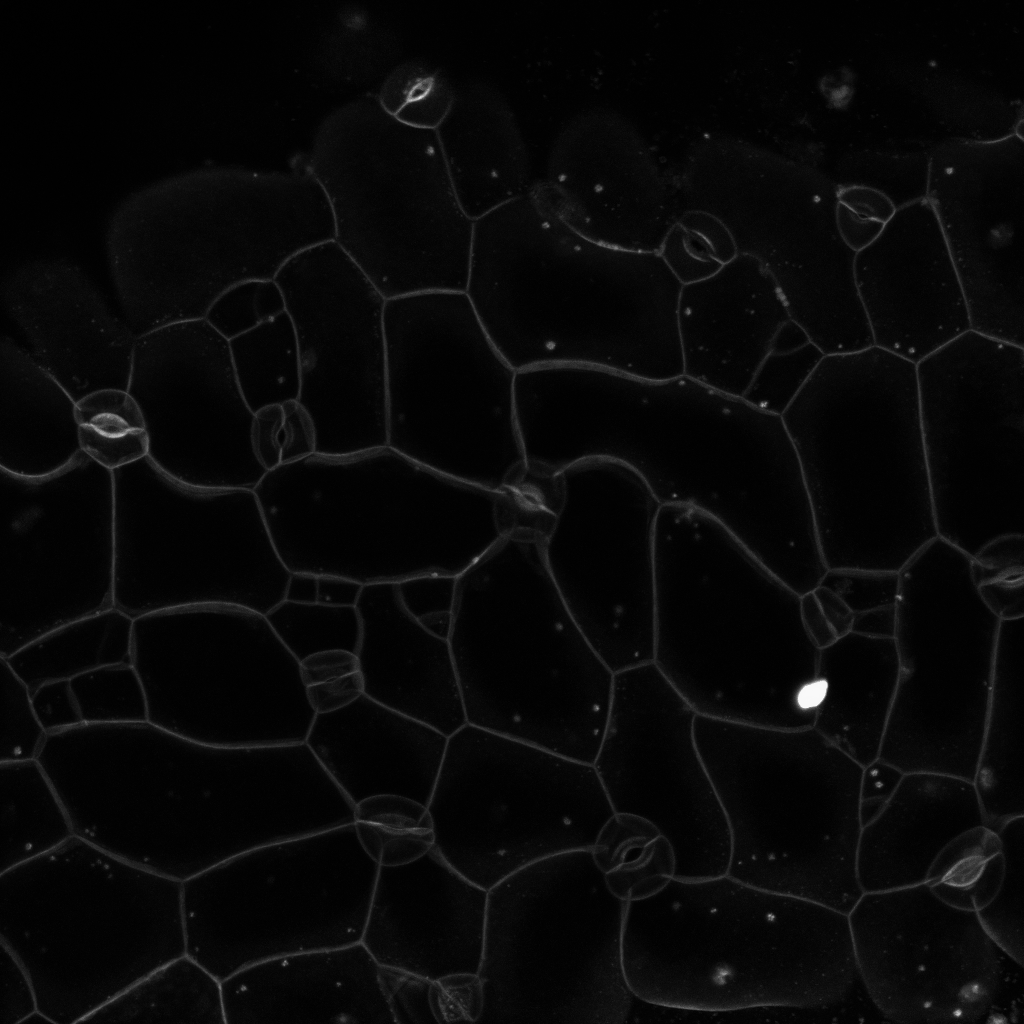

Supplement: Supplementary file 4 — Source Data [file 41467_2020_20730_MOESM4_ESM.zip › SourceData/Figure7_ShapeMutants/Images/CA-ROP2/CA-ROP2_S1.tif]

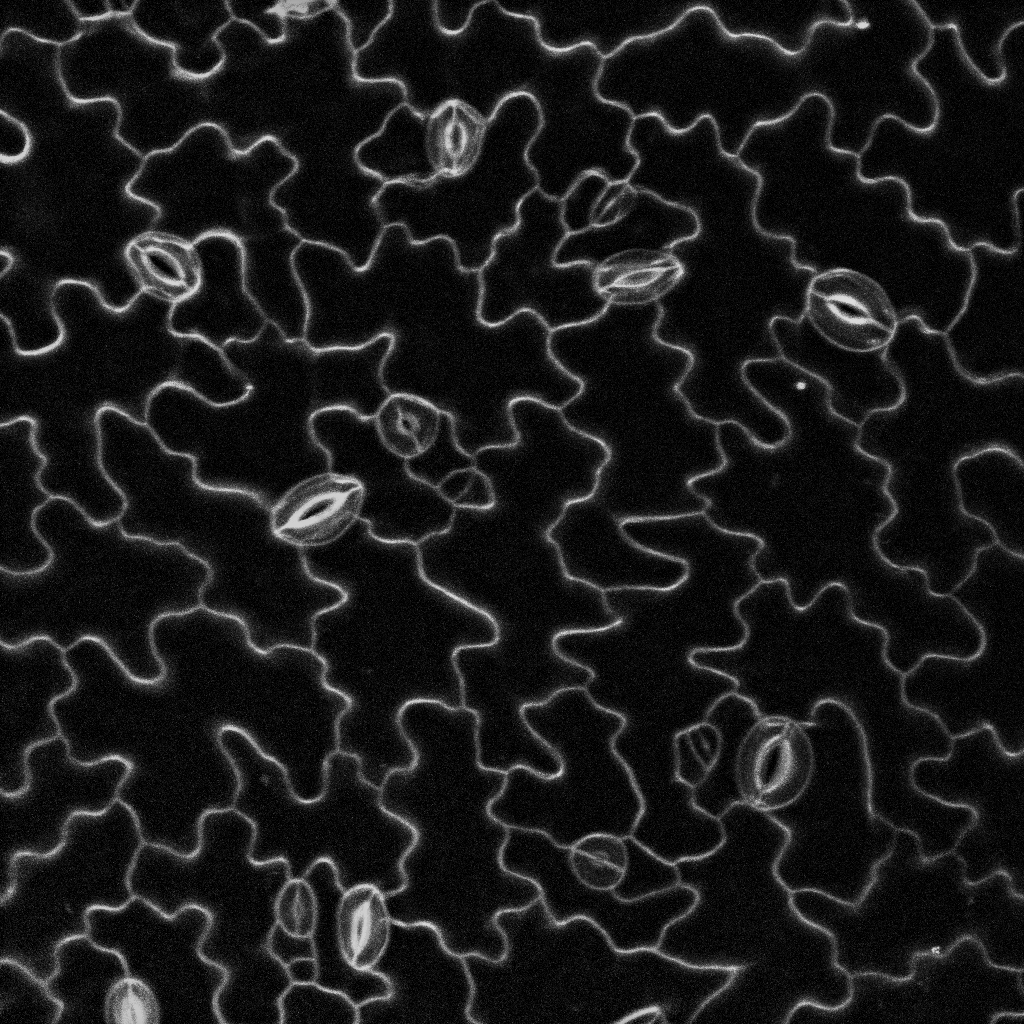

Supplement: Supplementary file 4 — Source Data [file 41467_2020_20730_MOESM4_ESM.zip › SourceData/Figure7_ShapeMutants/Images/spr2-2/spr2-2_S6.tif]

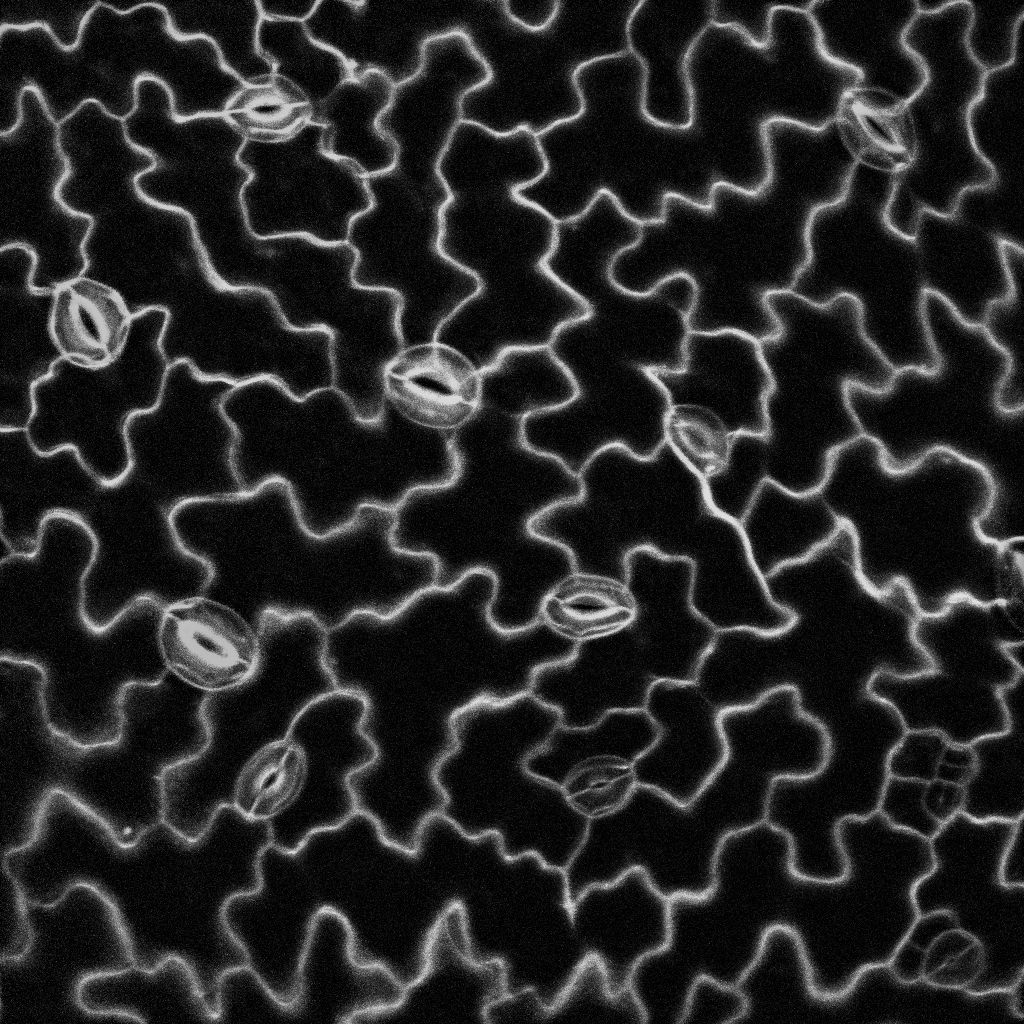

Supplement: Supplementary file 4 — Source Data [file 41467_2020_20730_MOESM4_ESM.zip › SourceData/Figure7_ShapeMutants/Images/spr2-2/spr2-2_S5.tif]

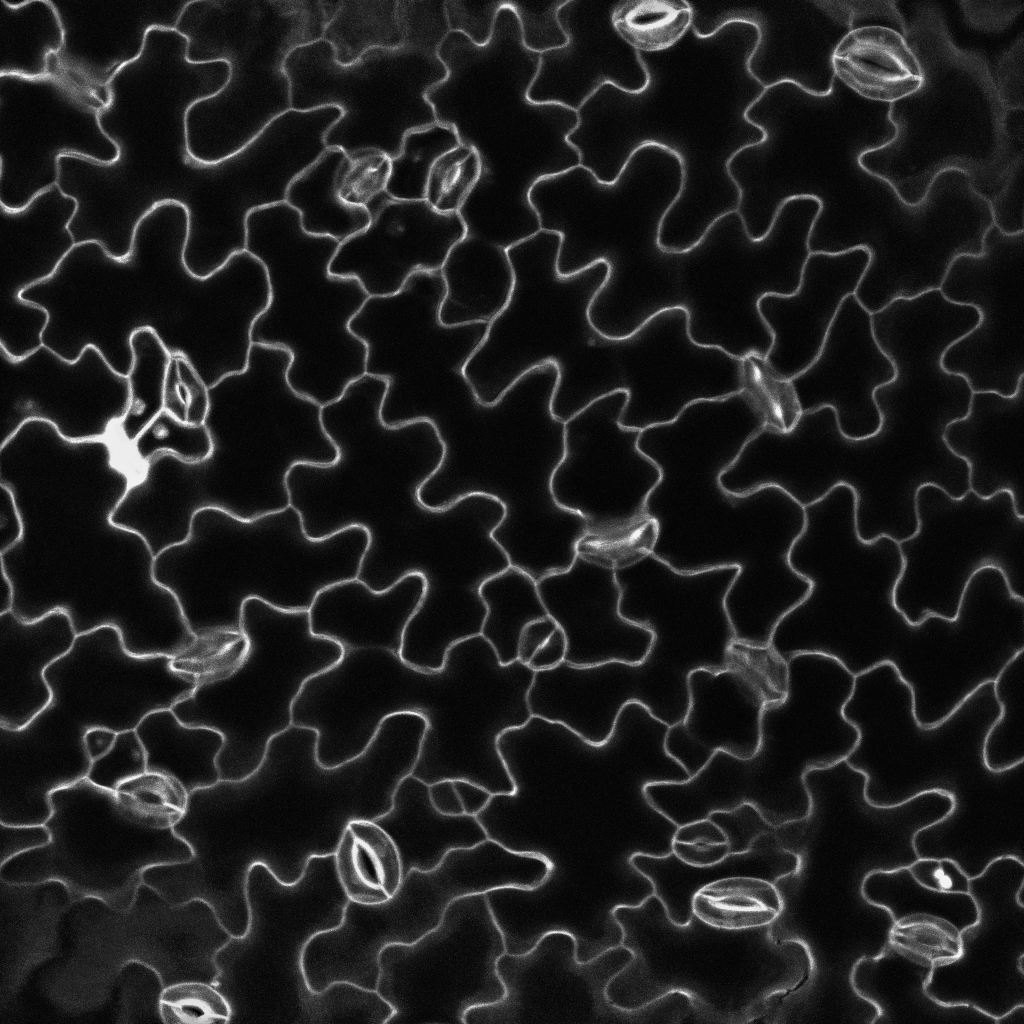

Supplement: Supplementary file 4 — Source Data [file 41467_2020_20730_MOESM4_ESM.zip › SourceData/Figure7_ShapeMutants/Images/spr2-2/spr2-2_S4.tif]

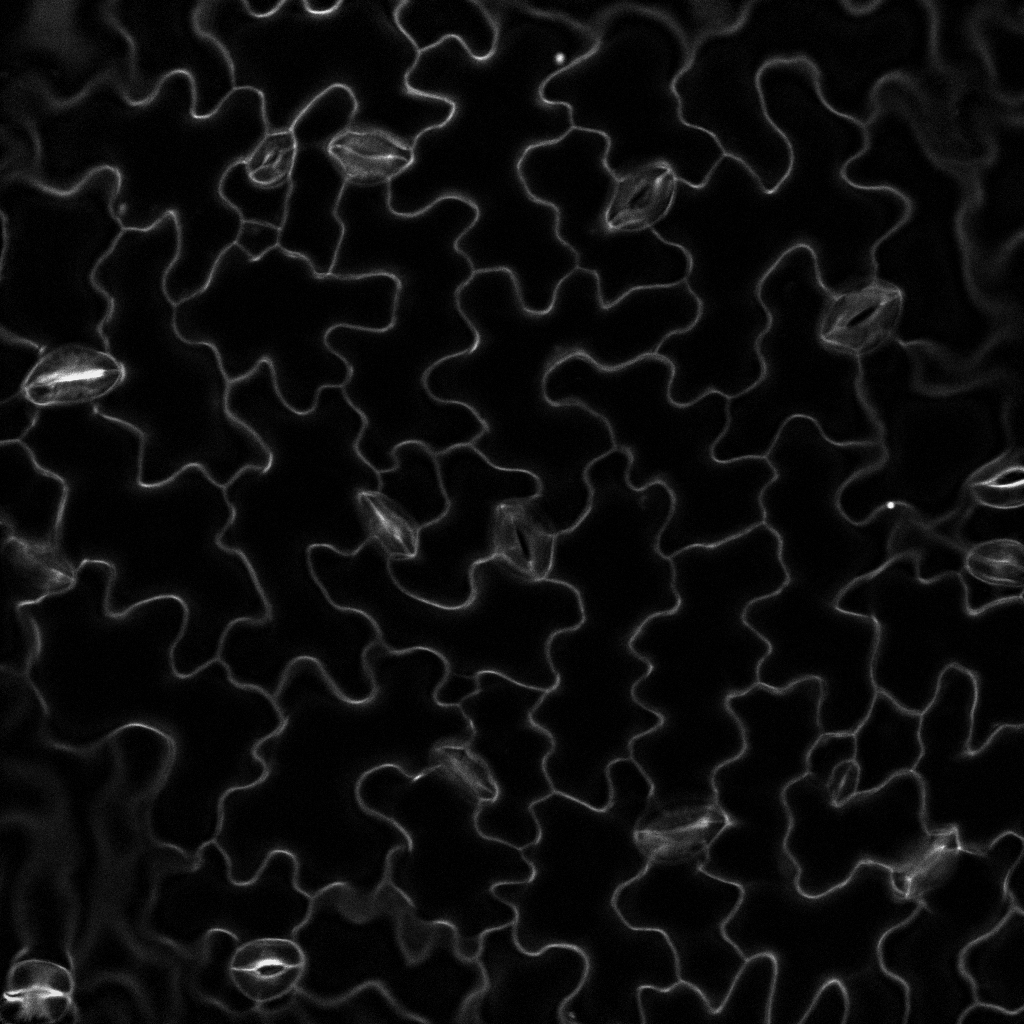

Supplement: Supplementary file 4 — Source Data [file 41467_2020_20730_MOESM4_ESM.zip › SourceData/Figure7_ShapeMutants/Images/spr2-2/spr2-2_S1.tif]

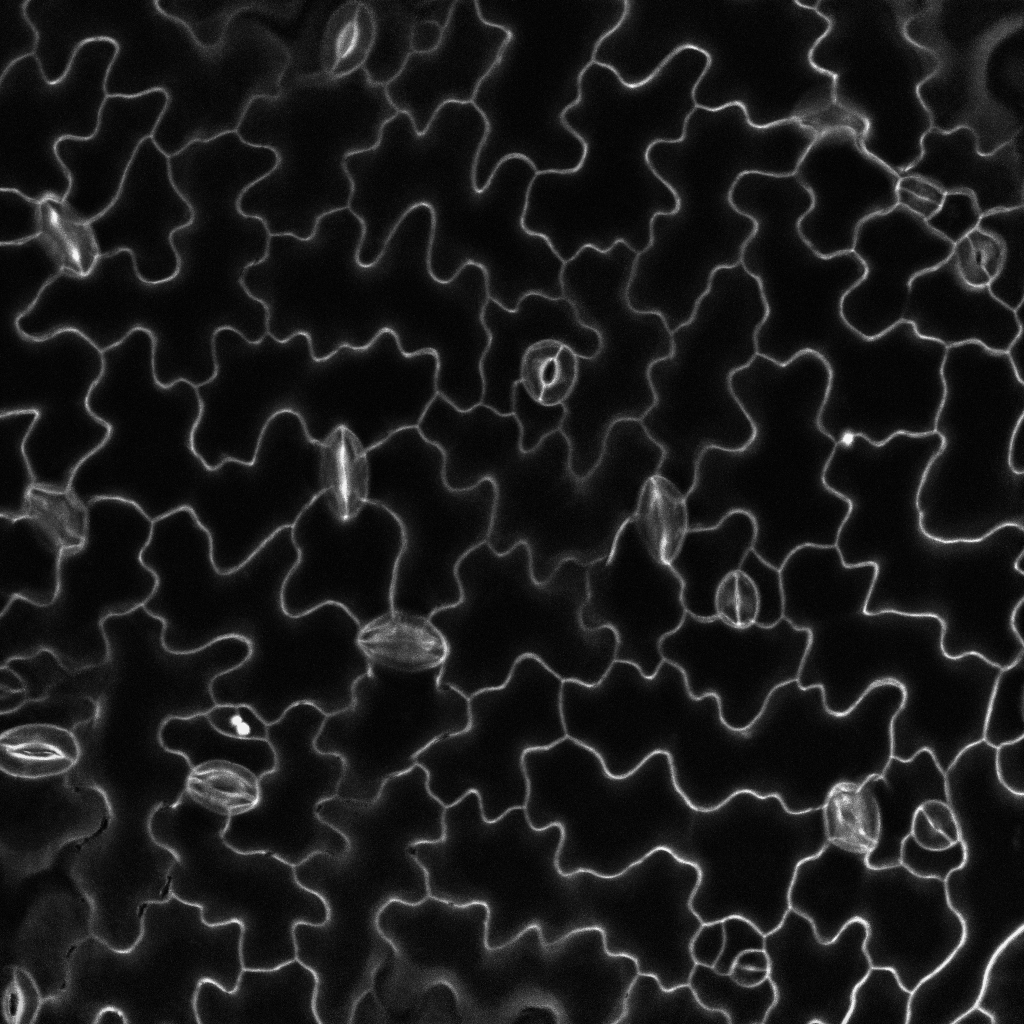

Supplement: Supplementary file 4 — Source Data [file 41467_2020_20730_MOESM4_ESM.zip › SourceData/Figure7_ShapeMutants/Images/spr2-2/spr2-2_S3.tif]

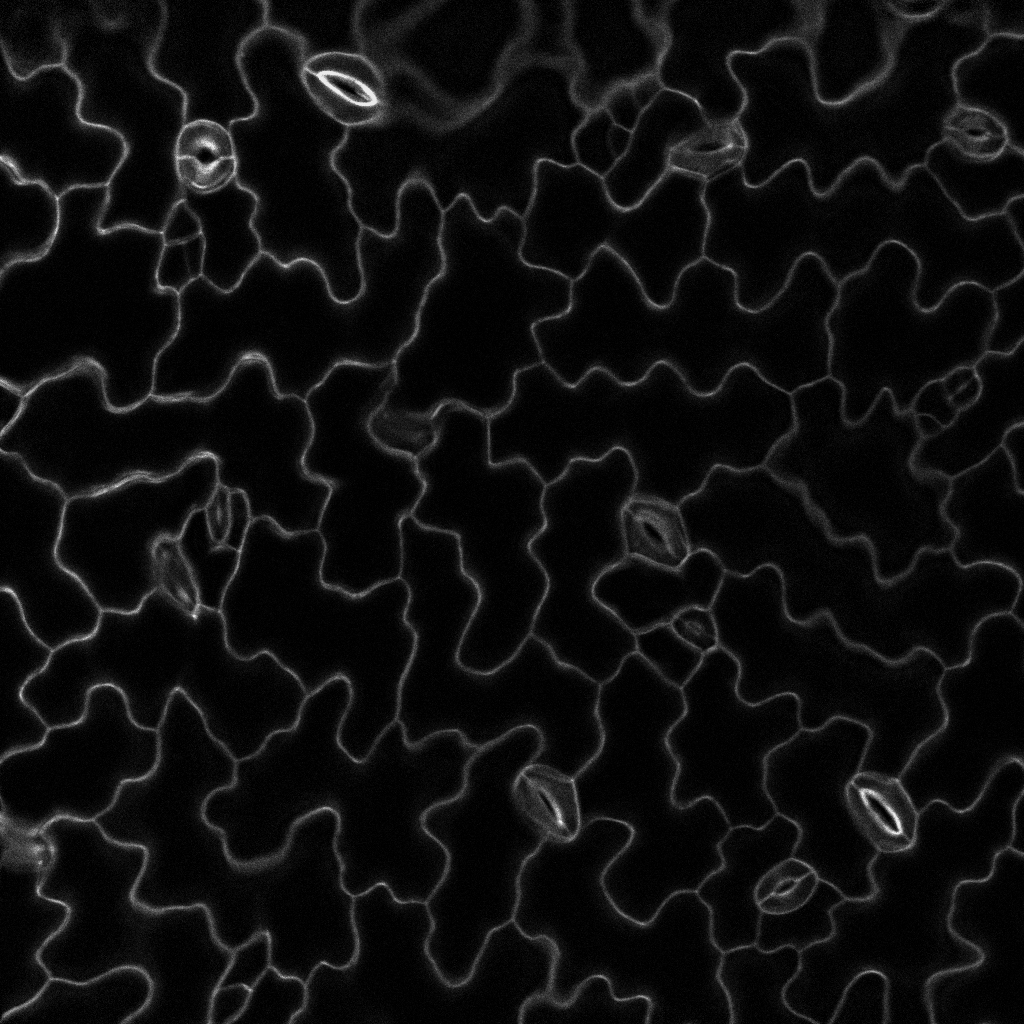

Supplement: Supplementary file 4 — Source Data [file 41467_2020_20730_MOESM4_ESM.zip › SourceData/Figure7_ShapeMutants/Images/spr2-2/spr2-2_S2.tif]

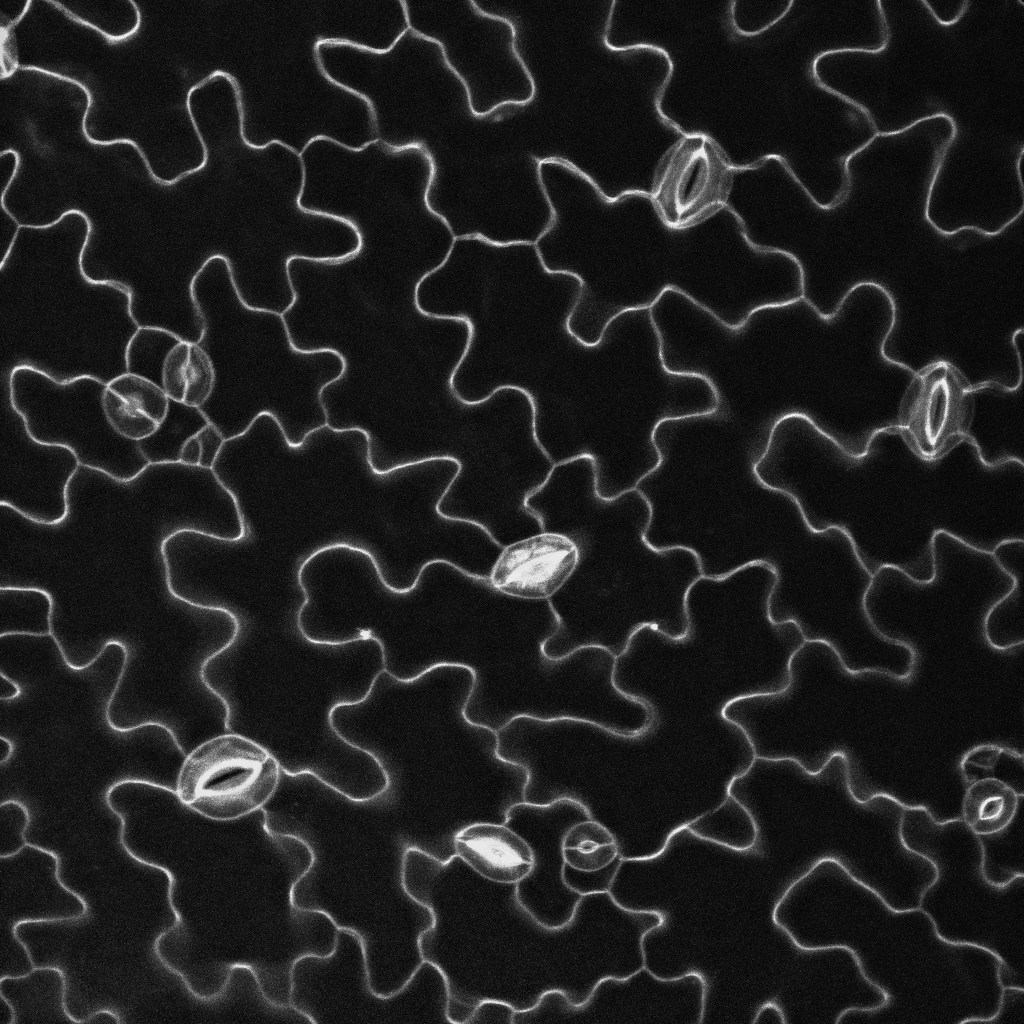

Supplement: Supplementary file 4 — Source Data [file 41467_2020_20730_MOESM4_ESM.zip › SourceData/Figure7_ShapeMutants/Images/Col-0/Col-0_S4.tif]

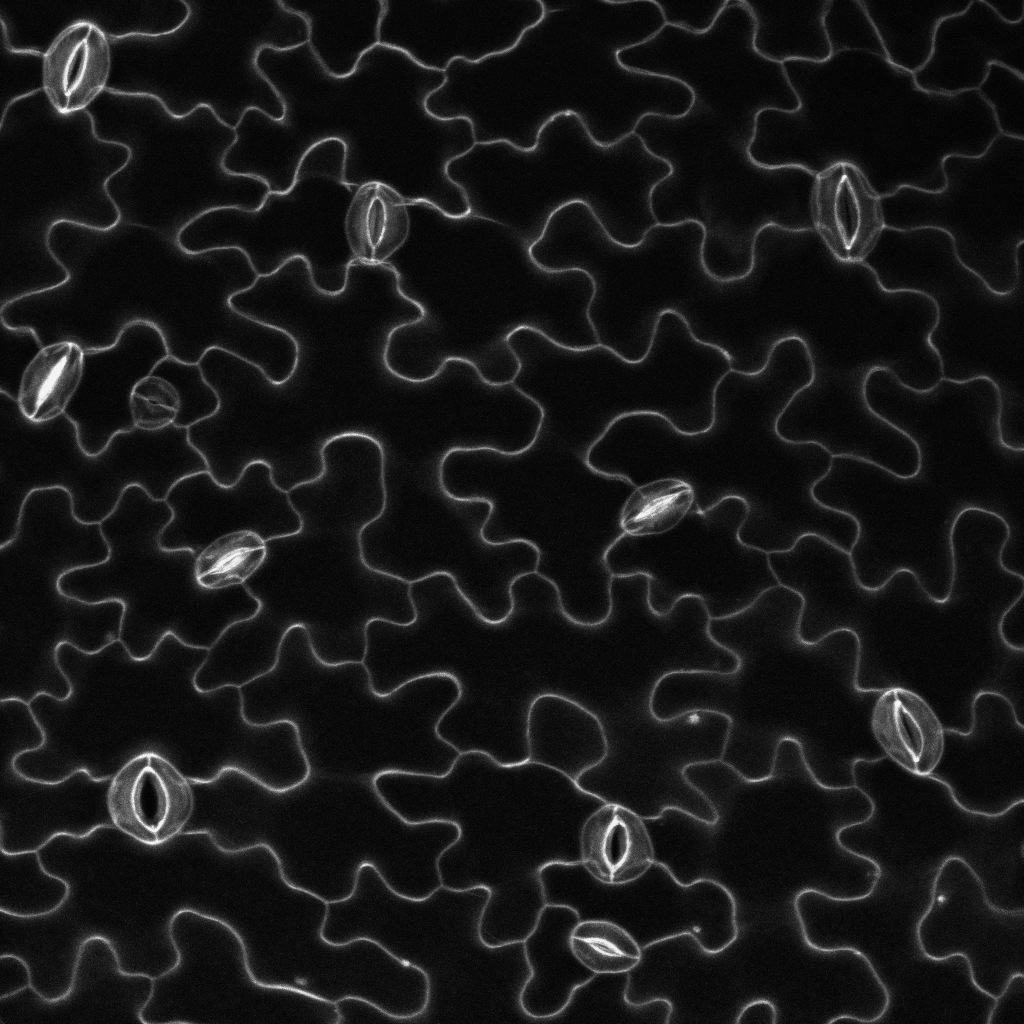

Supplement: Supplementary file 4 — Source Data [file 41467_2020_20730_MOESM4_ESM.zip › SourceData/Figure7_ShapeMutants/Images/Col-0/Col-0_S5.tif]

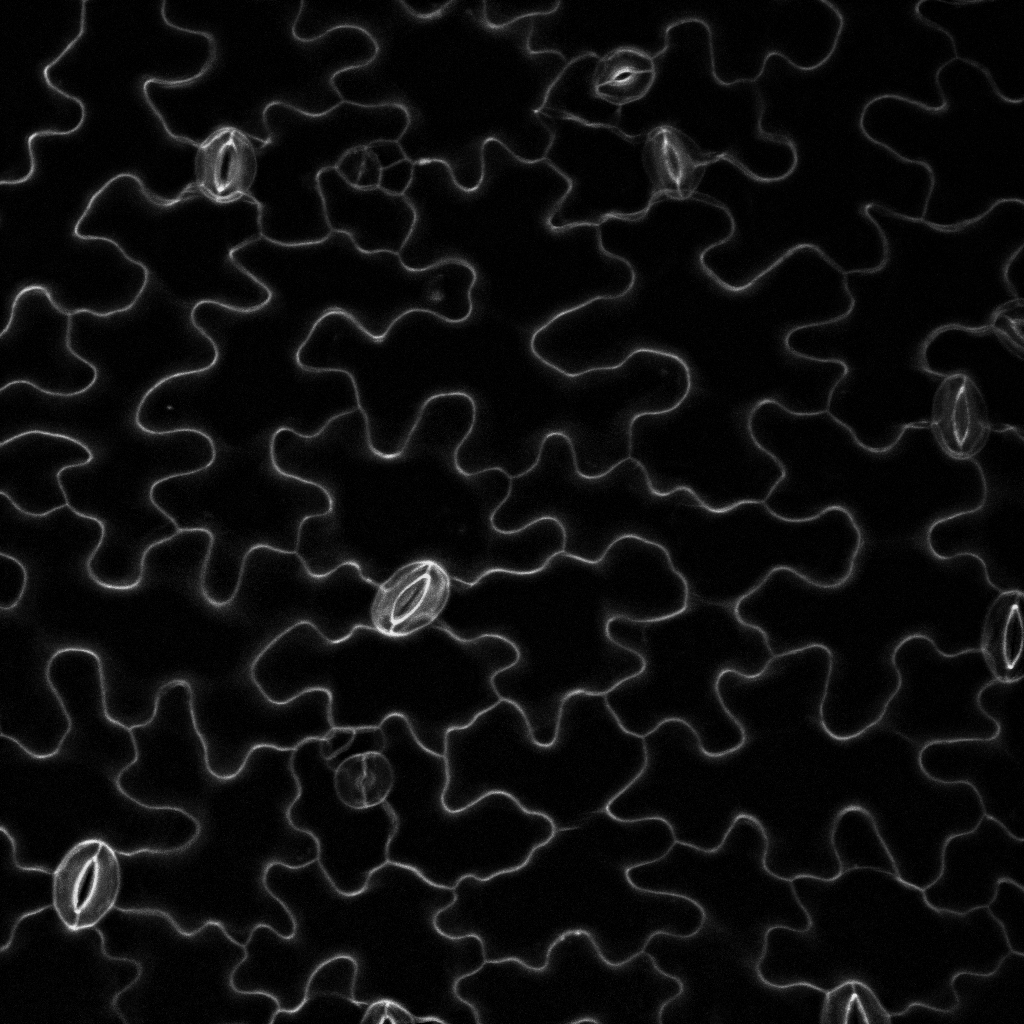

Supplement: Supplementary file 4 — Source Data [file 41467_2020_20730_MOESM4_ESM.zip › SourceData/Figure7_ShapeMutants/Images/Col-0/Col-0_S6.tif]

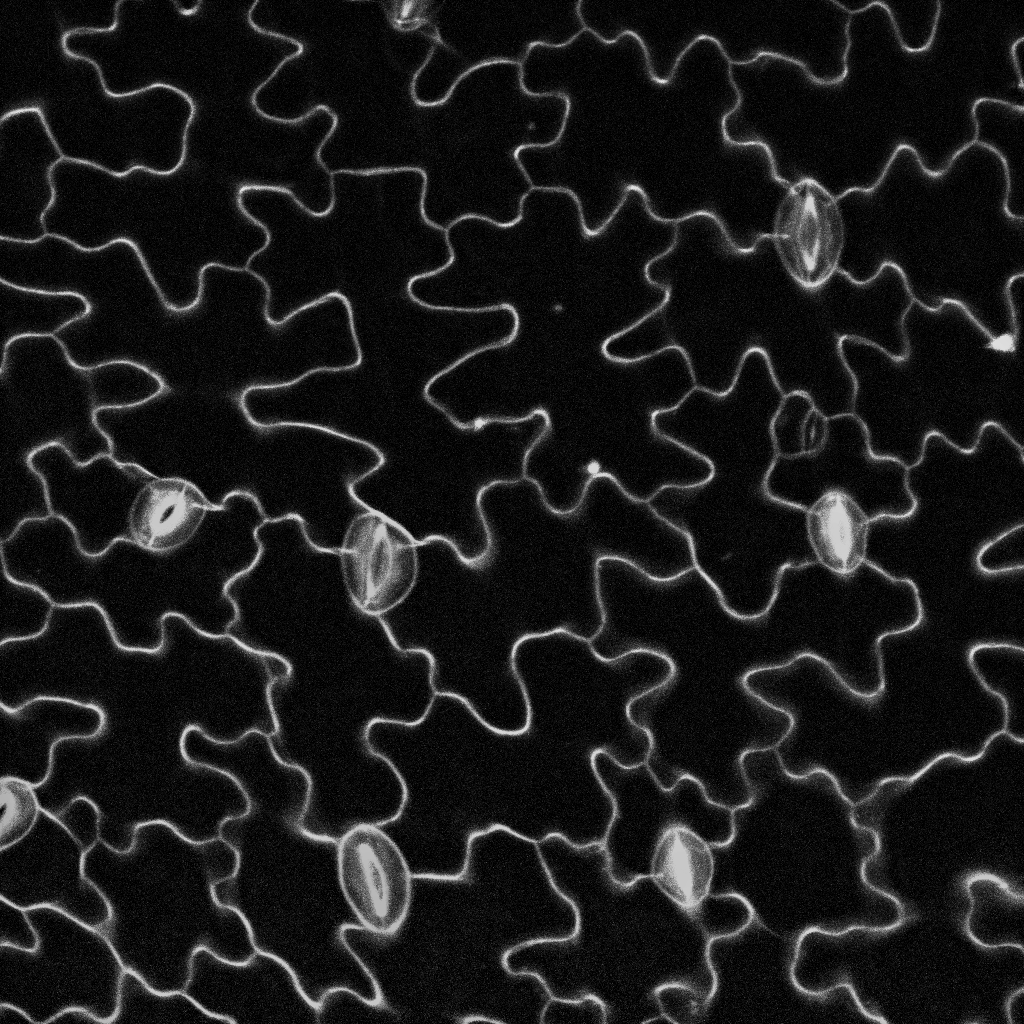

Supplement: Supplementary file 4 — Source Data [file 41467_2020_20730_MOESM4_ESM.zip › SourceData/Figure7_ShapeMutants/Images/Col-0/Col-0_S2.tif]

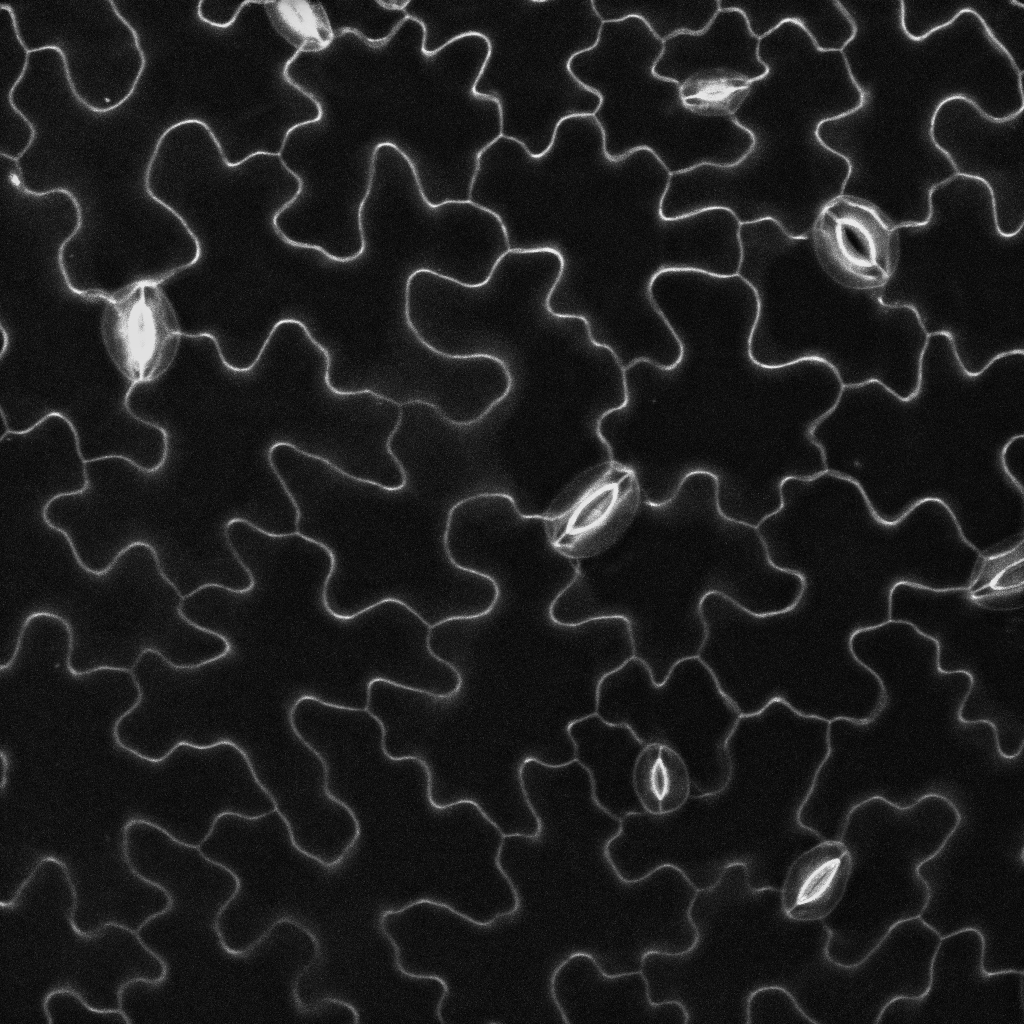

Supplement: Supplementary file 4 — Source Data [file 41467_2020_20730_MOESM4_ESM.zip › SourceData/Figure7_ShapeMutants/Images/Col-0/Col-0_S3.tif]

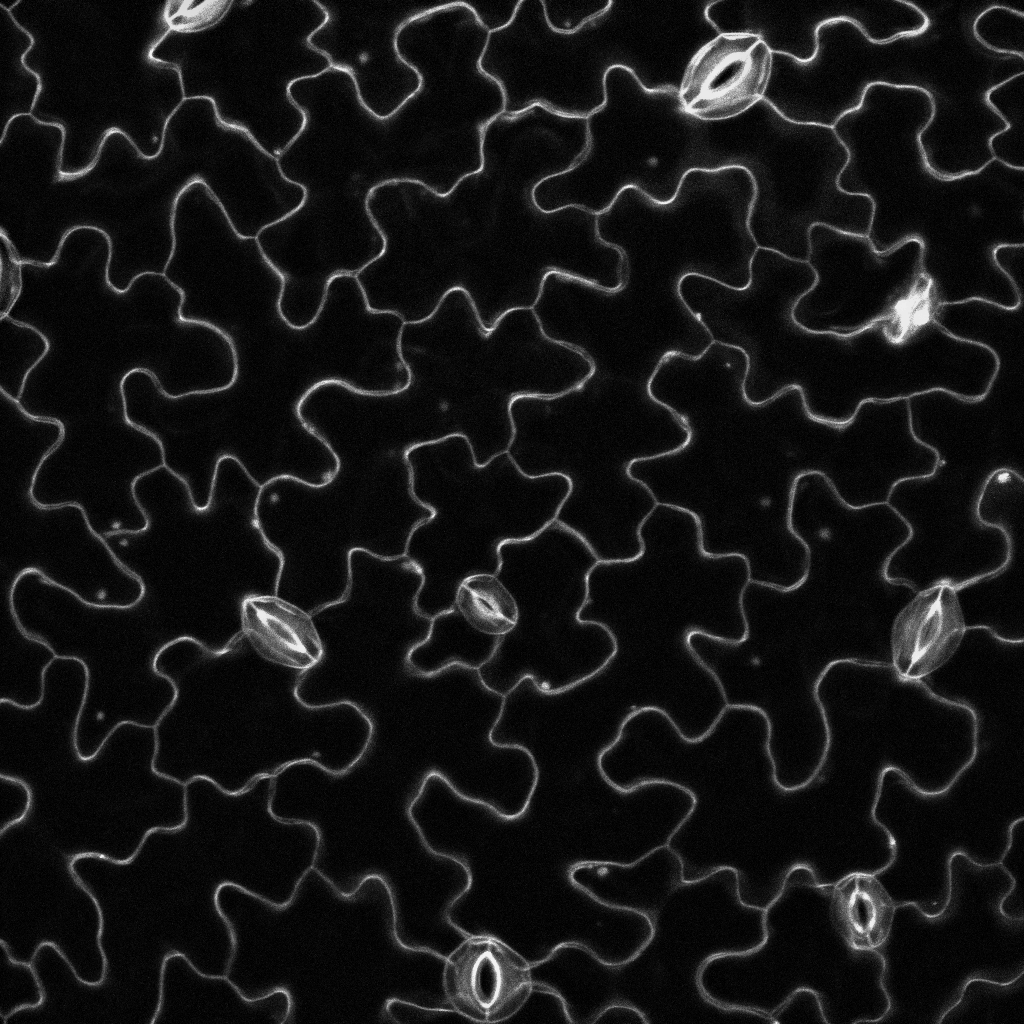

Supplement: Supplementary file 4 — Source Data [file 41467_2020_20730_MOESM4_ESM.zip › SourceData/Figure7_ShapeMutants/Images/Col-0/Col-0_S1.tif]

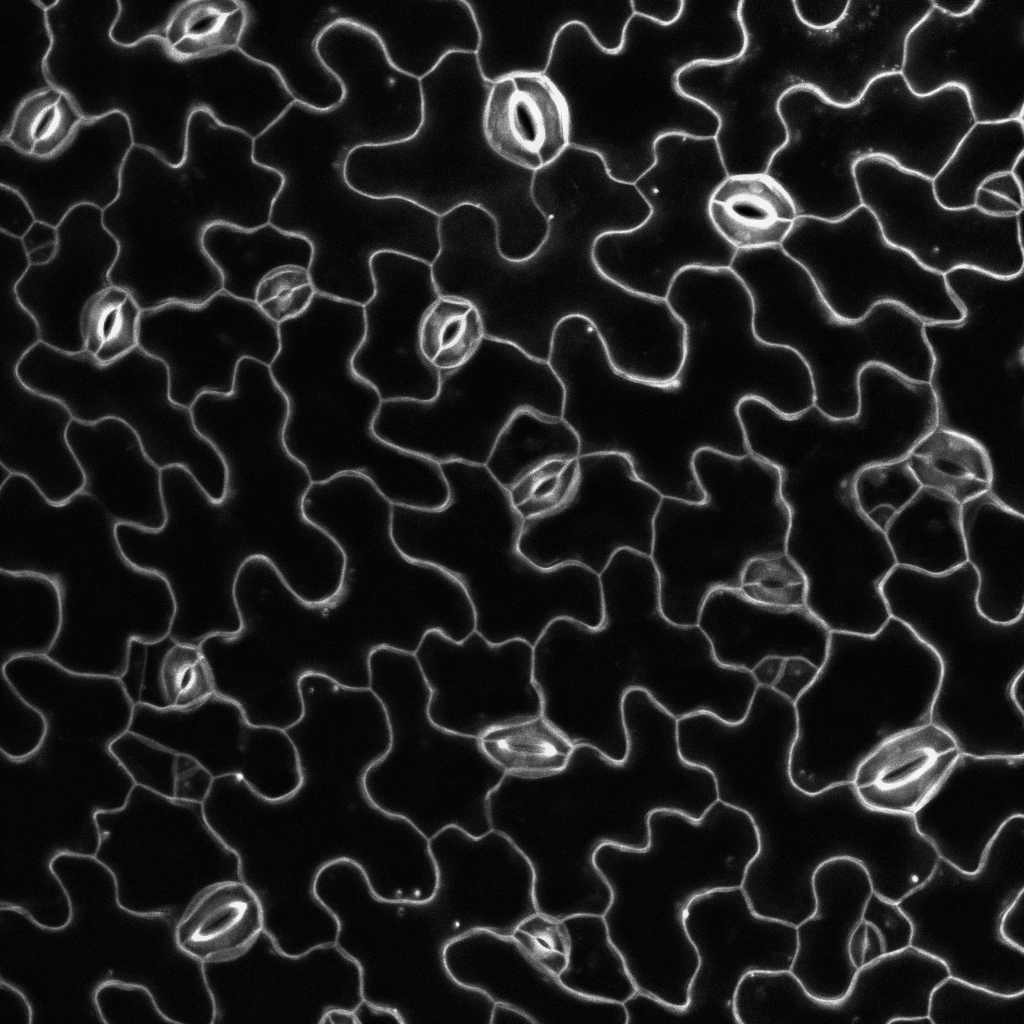

Supplement: Supplementary file 4 — Source Data [file 41467_2020_20730_MOESM4_ESM.zip › SourceData/Figure7_ShapeMutants/Images/rop4-1/rop4-1_S5.tif]

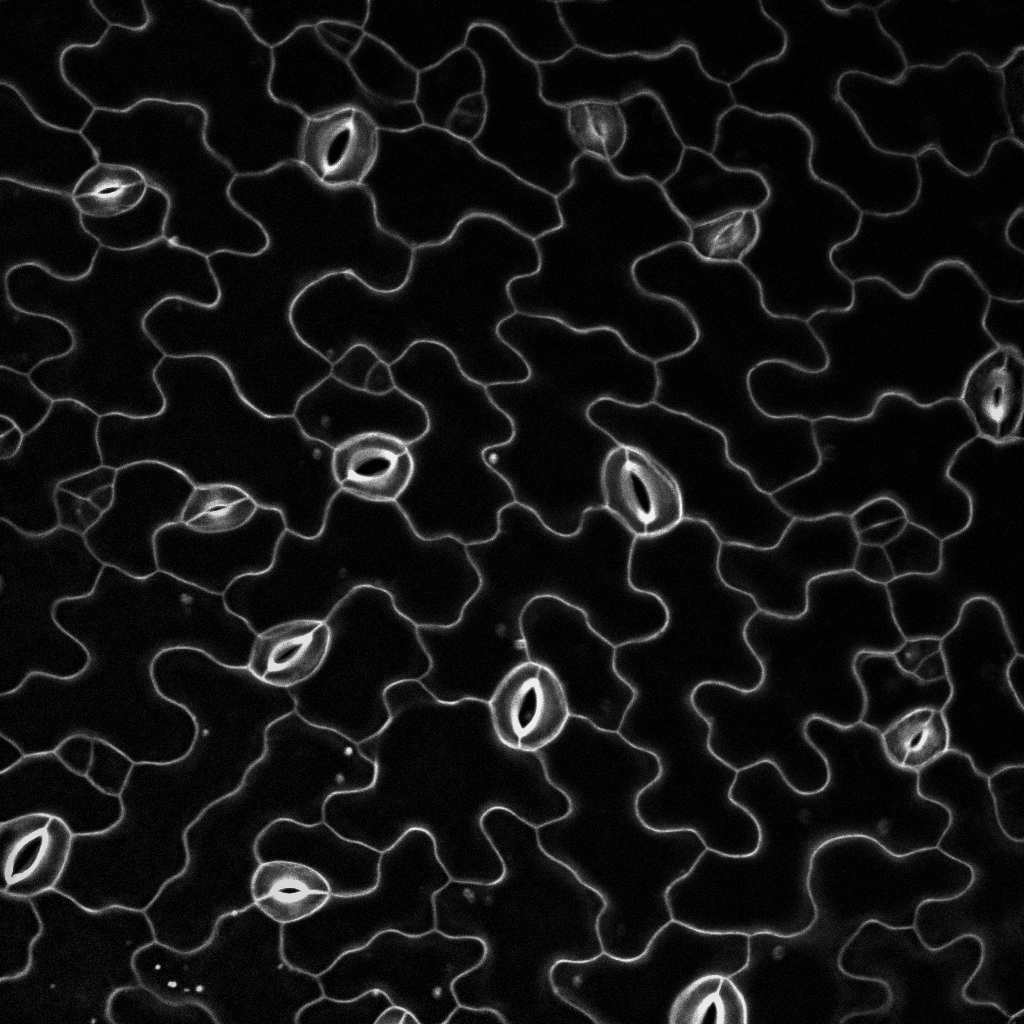

Supplement: Supplementary file 4 — Source Data [file 41467_2020_20730_MOESM4_ESM.zip › SourceData/Figure7_ShapeMutants/Images/rop4-1/rop4-1_S4.tif]

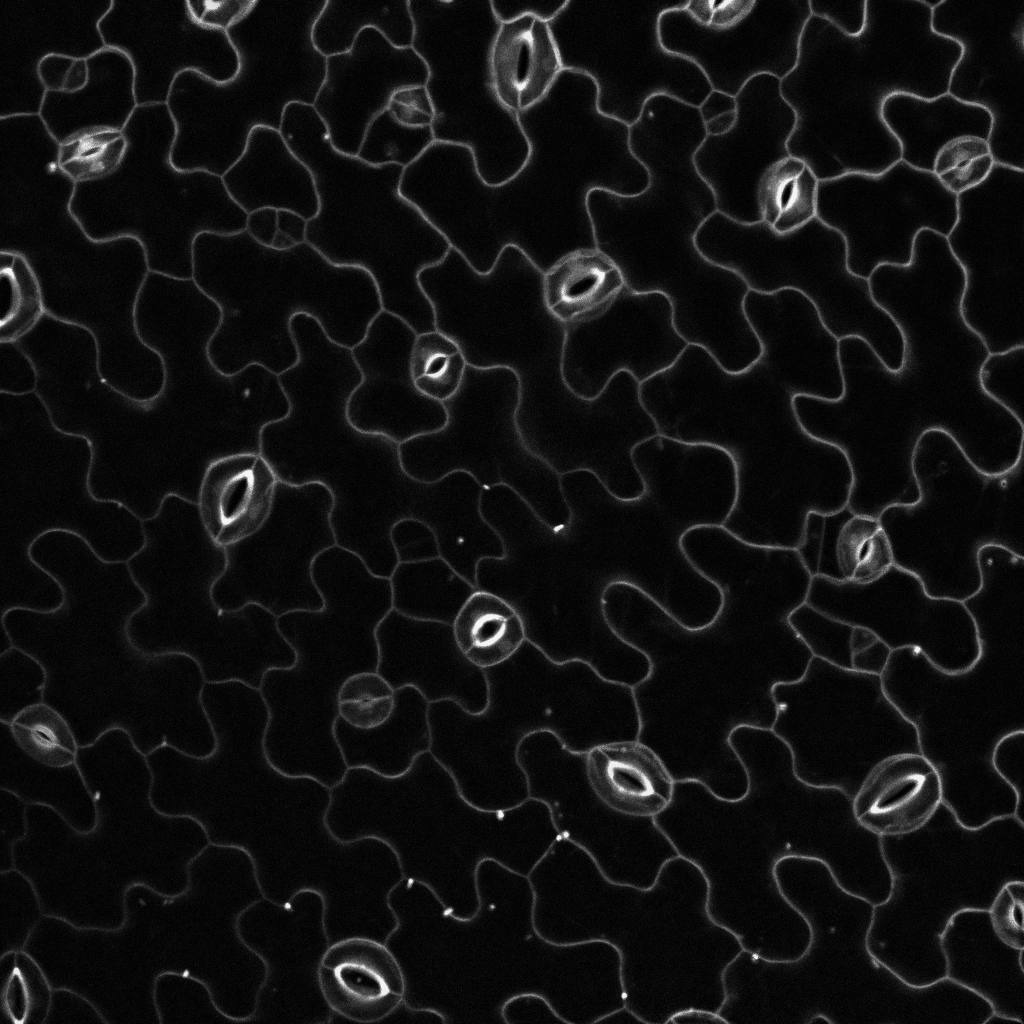

Supplement: Supplementary file 4 — Source Data [file 41467_2020_20730_MOESM4_ESM.zip › SourceData/Figure7_ShapeMutants/Images/rop4-1/rop4-1_S6.tif]

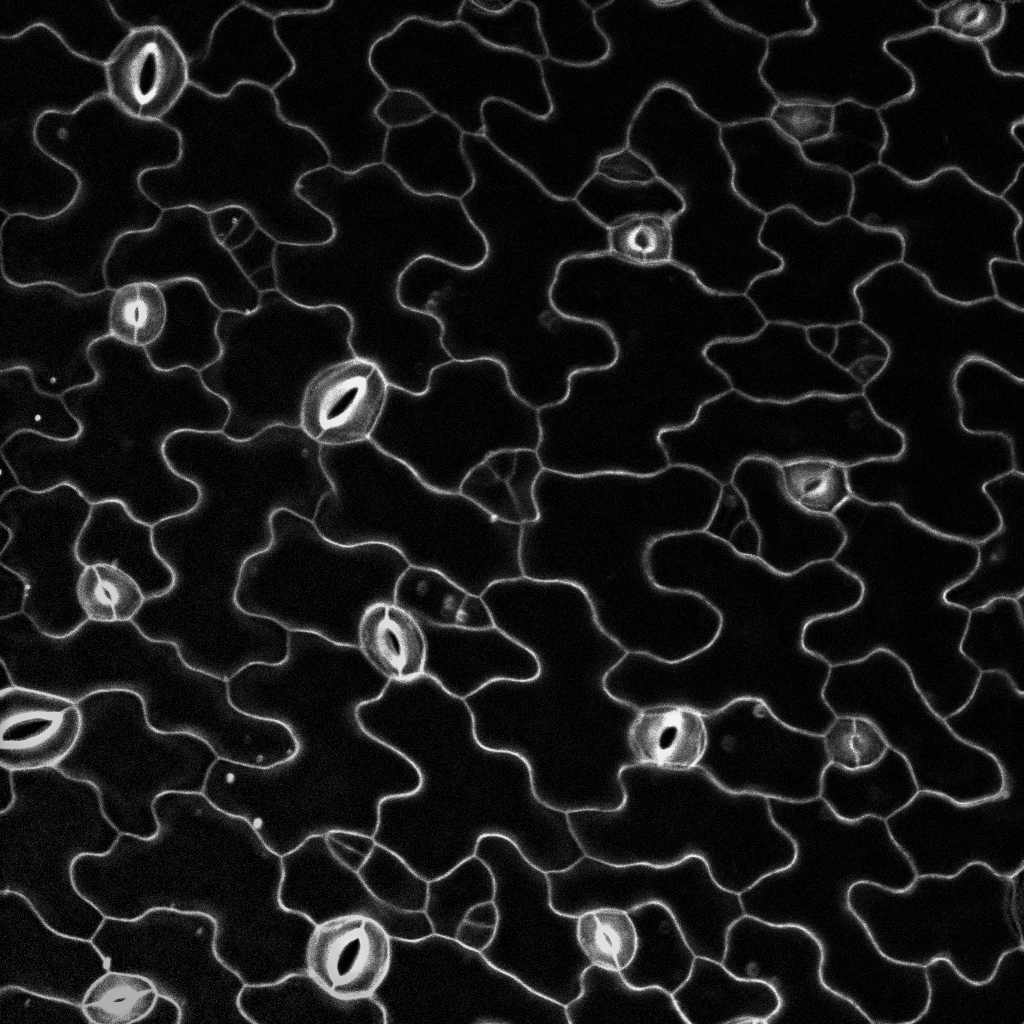

Supplement: Supplementary file 4 — Source Data [file 41467_2020_20730_MOESM4_ESM.zip › SourceData/Figure7_ShapeMutants/Images/rop4-1/rop4-1_S3.tif]

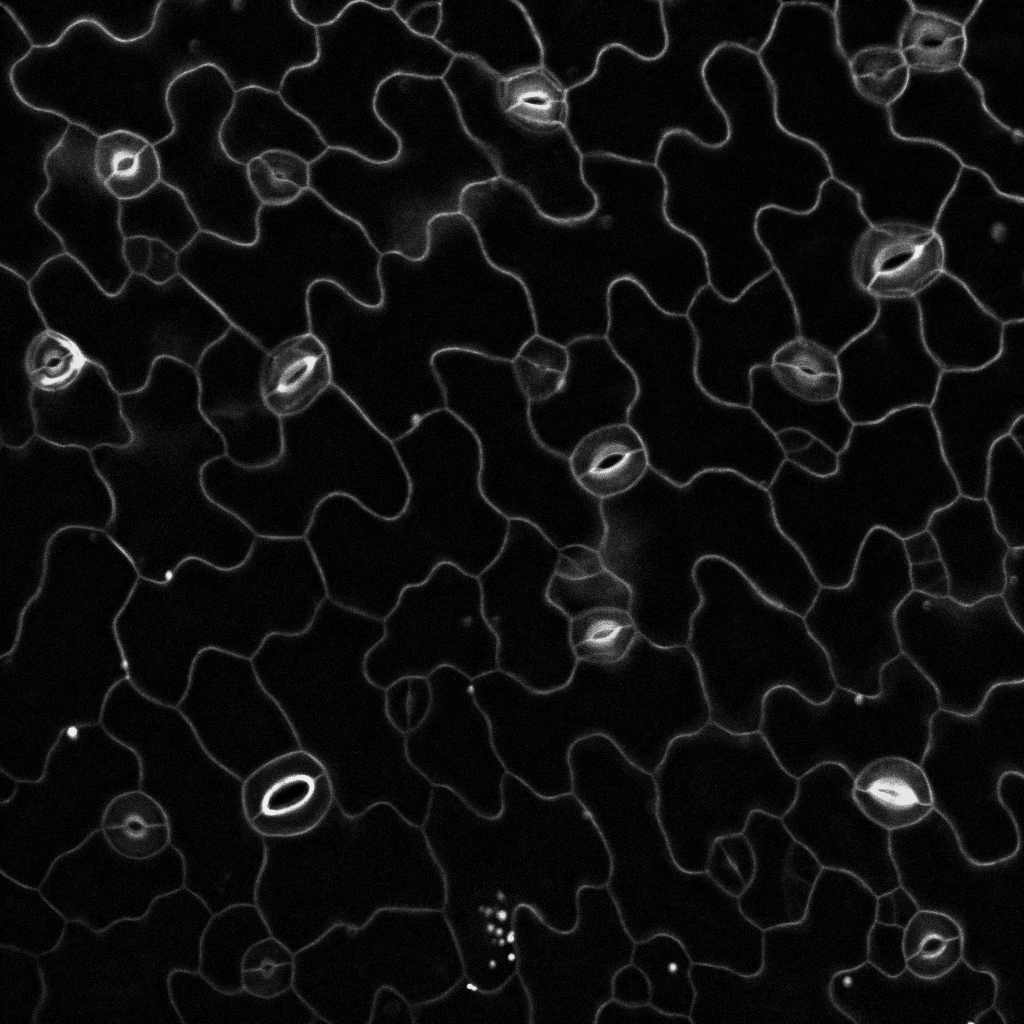

Supplement: Supplementary file 4 — Source Data [file 41467_2020_20730_MOESM4_ESM.zip › SourceData/Figure7_ShapeMutants/Images/rop4-1/rop4-1_S2.tif]

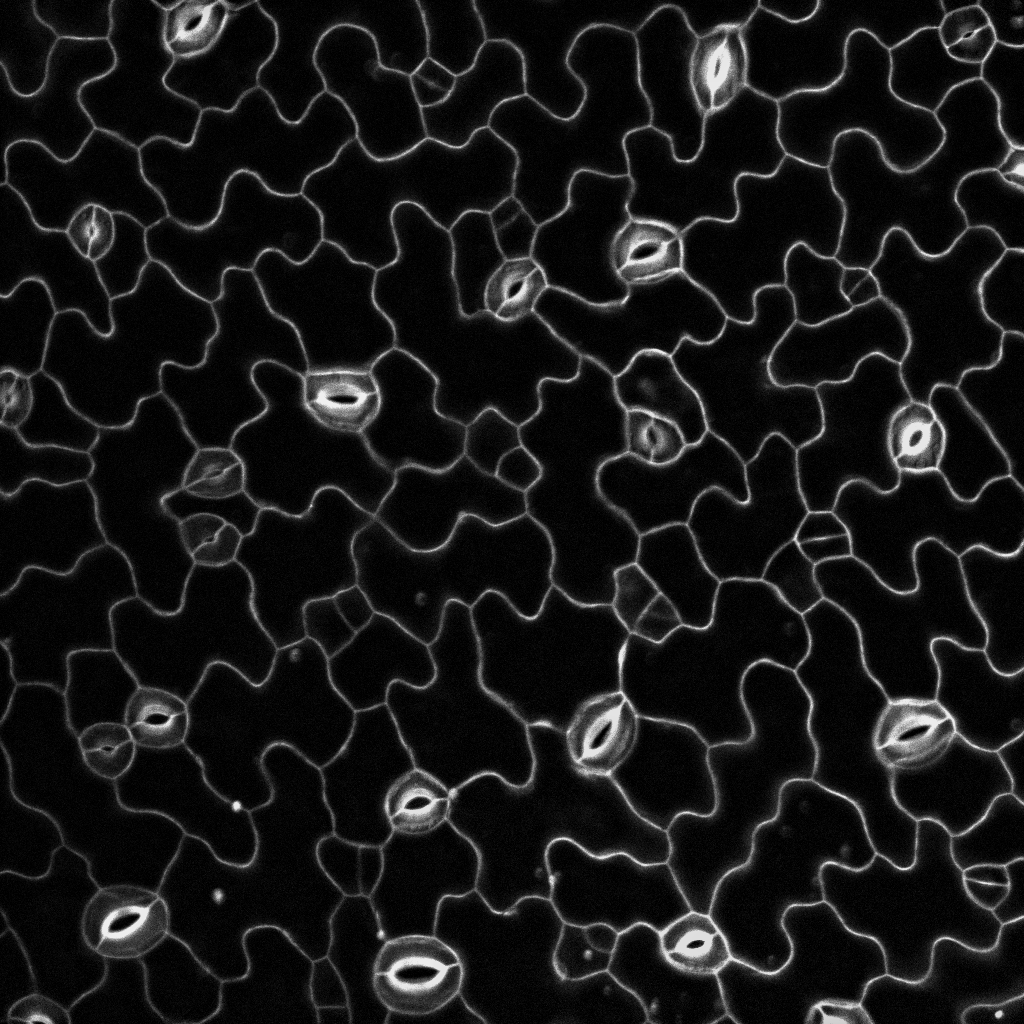

Supplement: Supplementary file 4 — Source Data [file 41467_2020_20730_MOESM4_ESM.zip › SourceData/Figure7_ShapeMutants/Images/rop4-1/rop4-1_S1.tif]

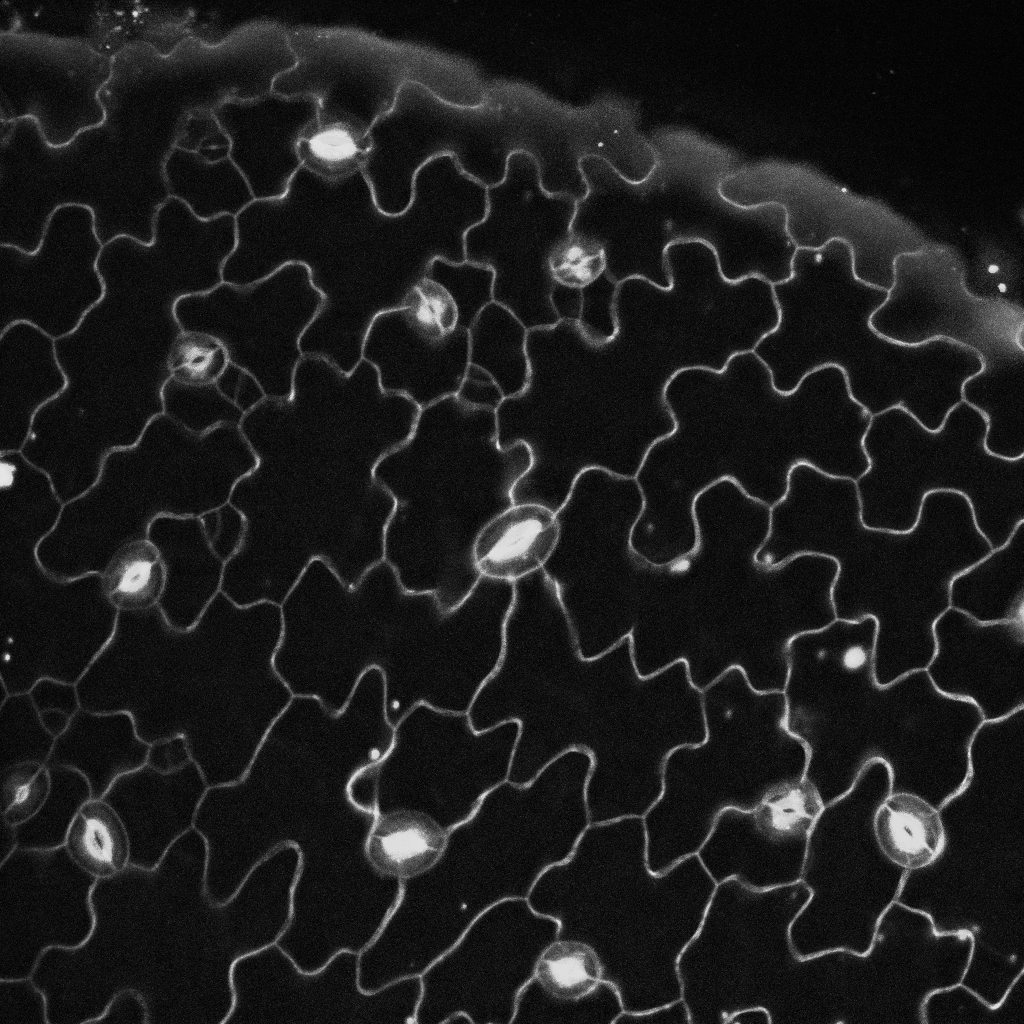

Supplement: Supplementary file 4 — Source Data [file 41467_2020_20730_MOESM4_ESM.zip › SourceData/Figure7_ShapeMutants/Images/DN-ROP2/DN-ROP2_S4.tif]

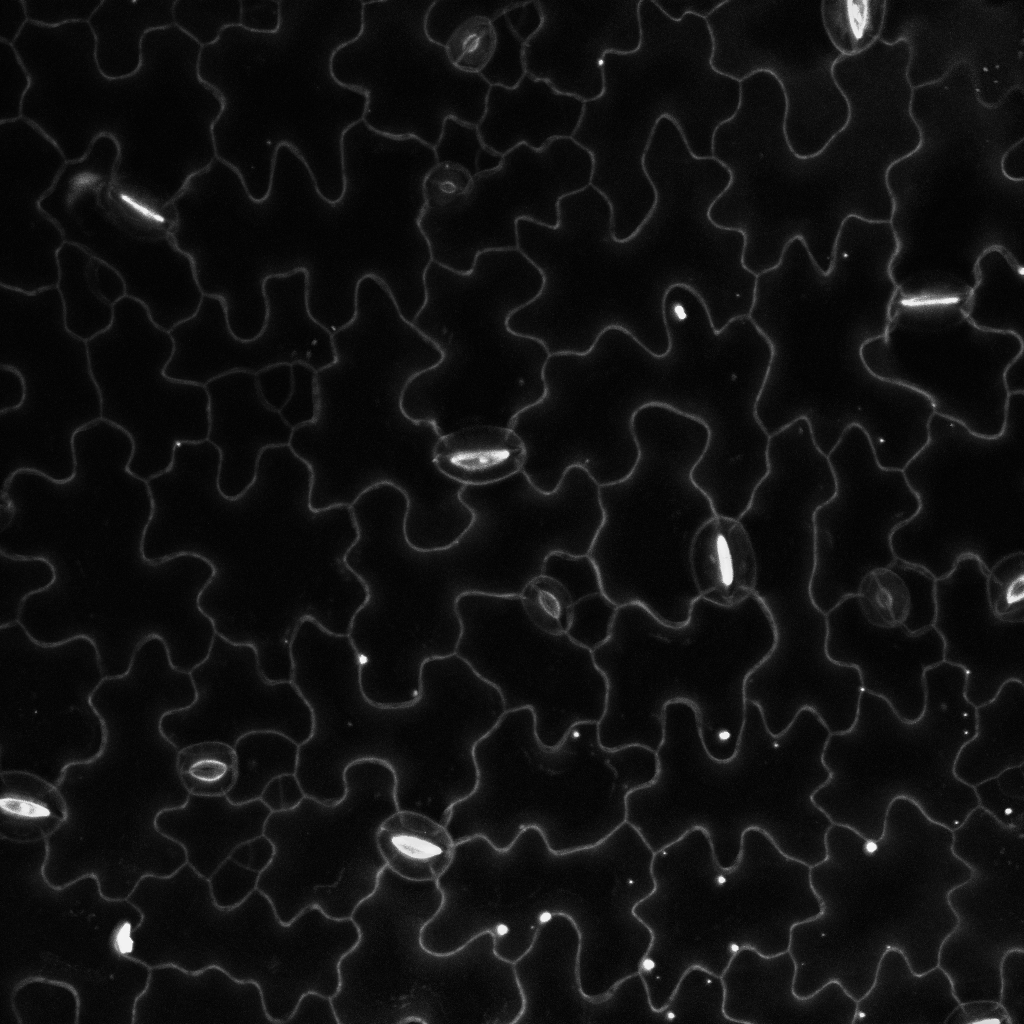

Supplement: Supplementary file 4 — Source Data [file 41467_2020_20730_MOESM4_ESM.zip › SourceData/Figure7_ShapeMutants/Images/DN-ROP2/DN-ROP2_S1.tif]

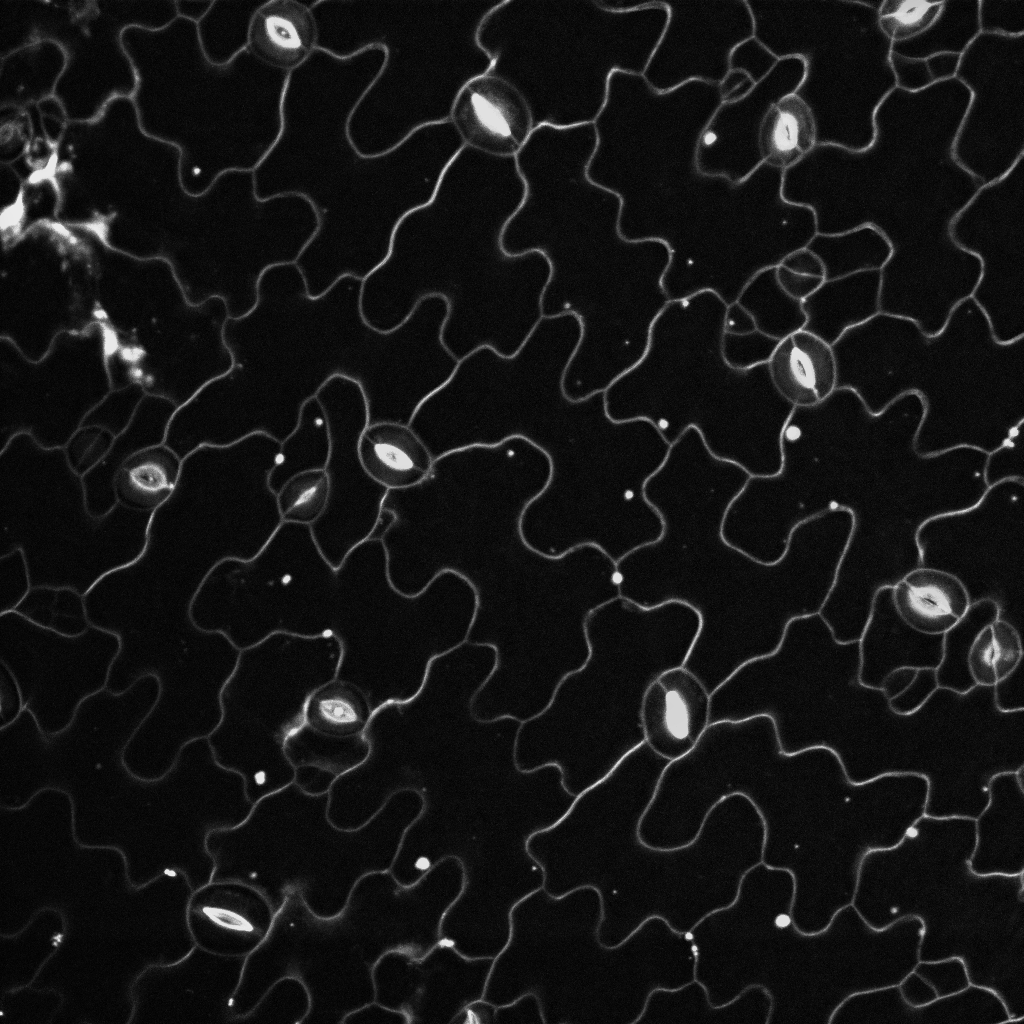

Supplement: Supplementary file 4 — Source Data [file 41467_2020_20730_MOESM4_ESM.zip › SourceData/Figure7_ShapeMutants/Images/DN-ROP2/DN-ROP2_S3.tif]

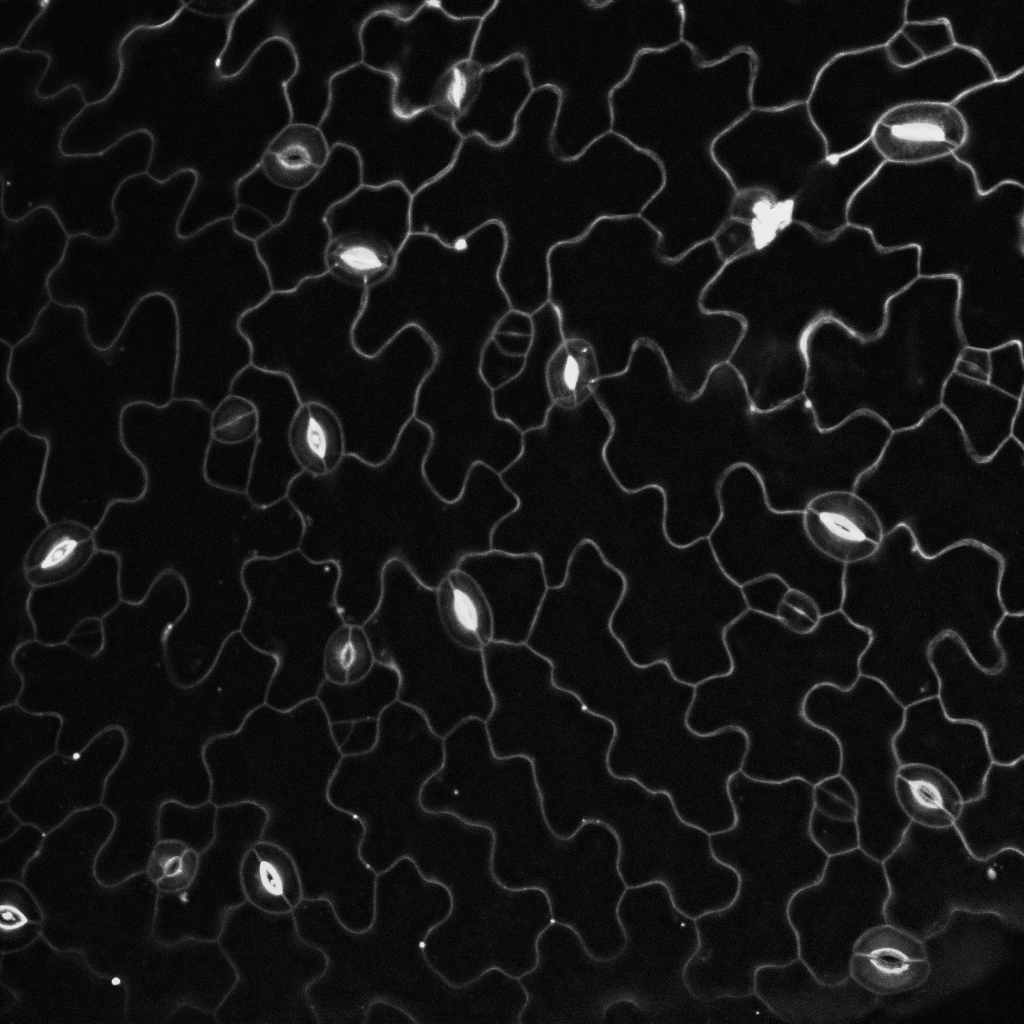

Supplement: Supplementary file 4 — Source Data [file 41467_2020_20730_MOESM4_ESM.zip › SourceData/Figure7_ShapeMutants/Images/DN-ROP2/DN-ROP2_S2.tif]

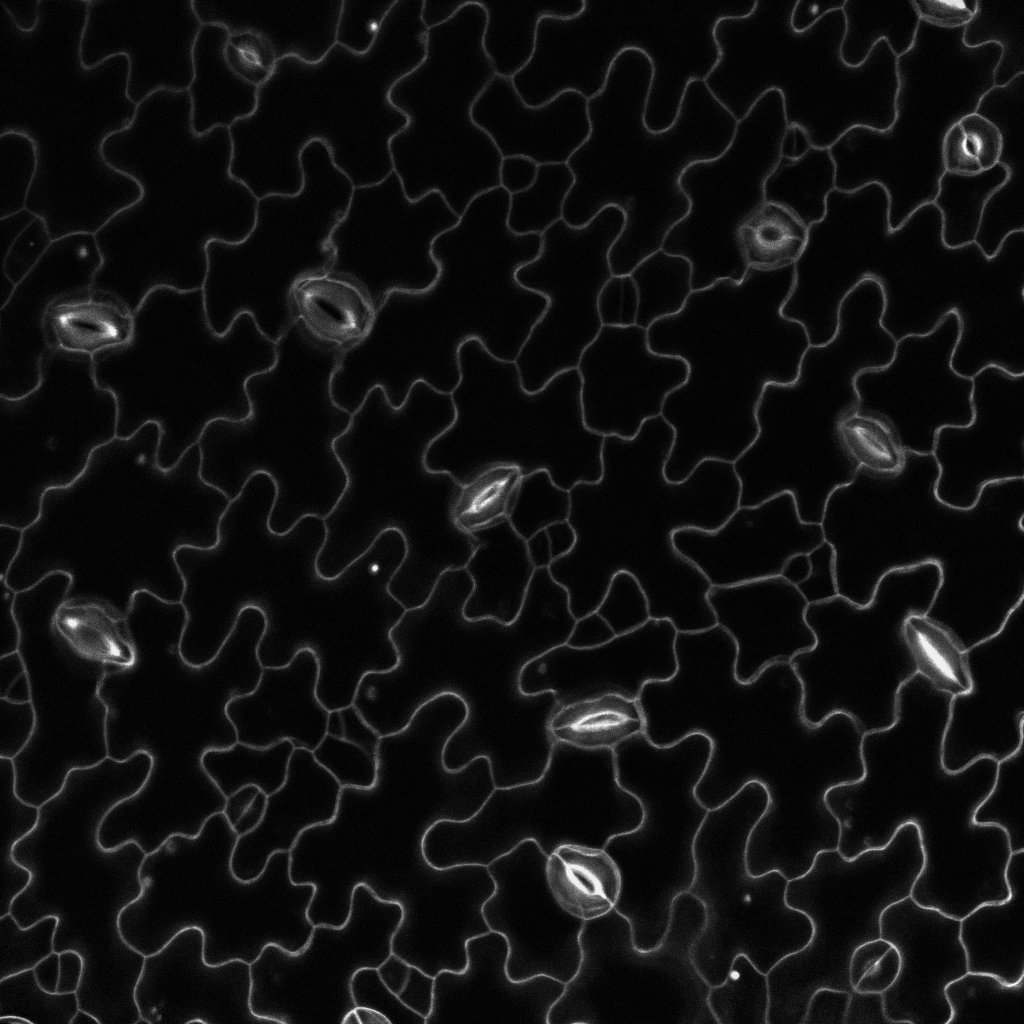

Supplement: Supplementary file 4 — Source Data [file 41467_2020_20730_MOESM4_ESM.zip › SourceData/Figure7_ShapeMutants/Images/lue1/lue1_S1.tif]

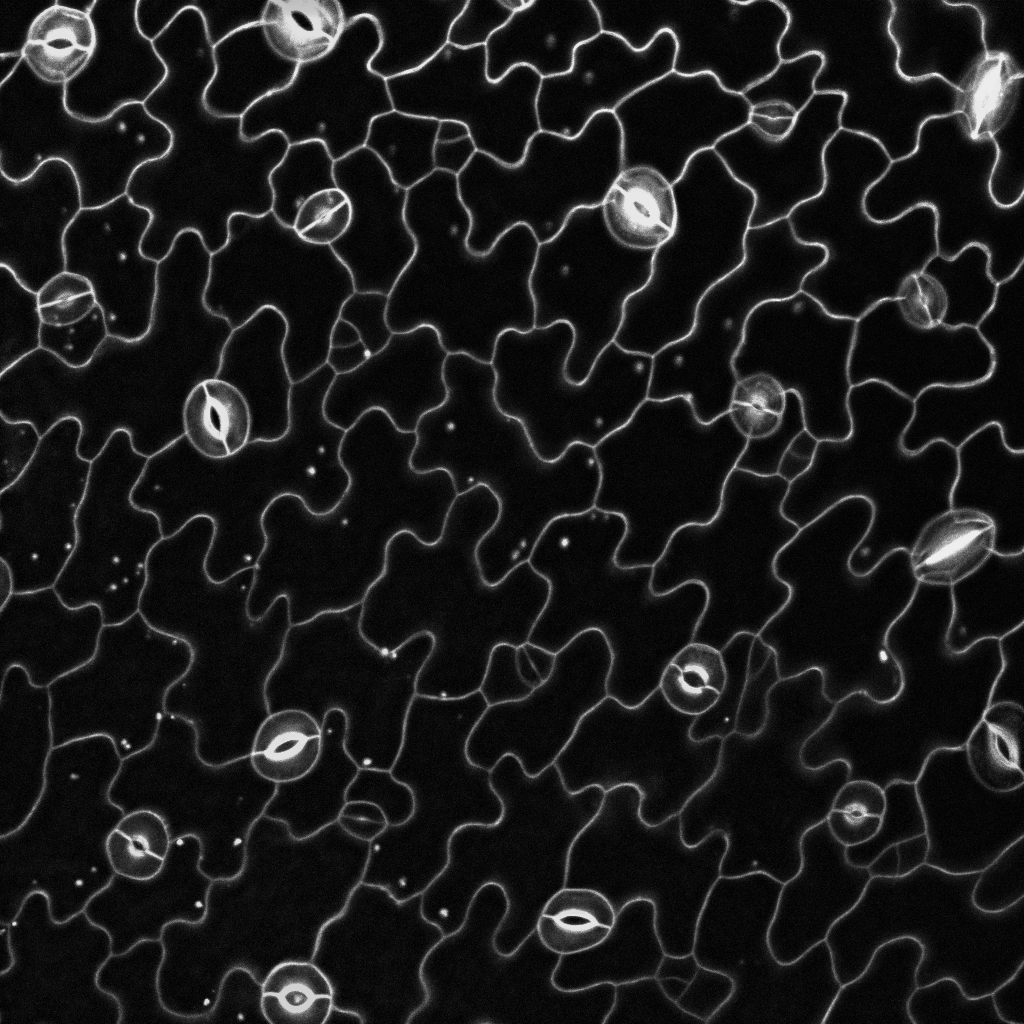

Supplement: Supplementary file 4 — Source Data [file 41467_2020_20730_MOESM4_ESM.zip › SourceData/Figure7_ShapeMutants/Images/lue1/lue1_S3.tif]

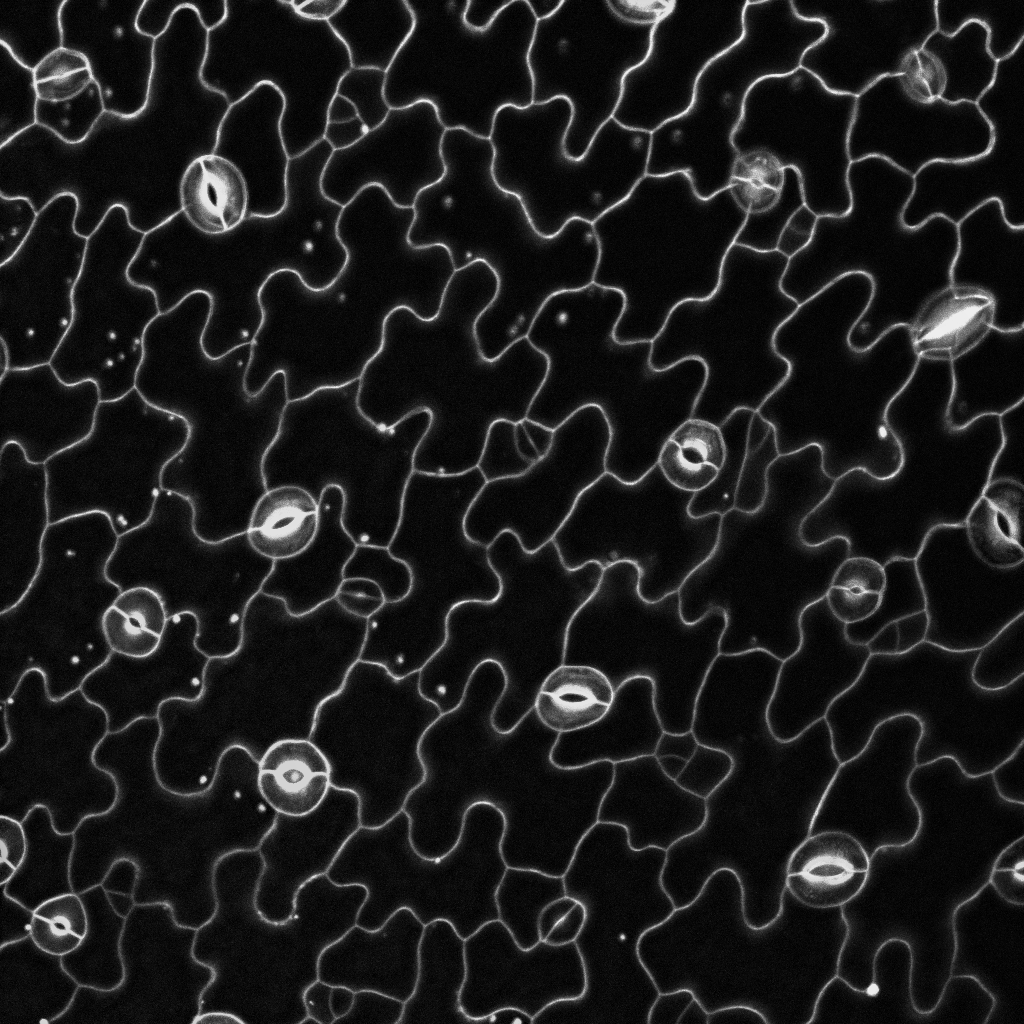

Supplement: Supplementary file 4 — Source Data [file 41467_2020_20730_MOESM4_ESM.zip › SourceData/Figure7_ShapeMutants/Images/lue1/lue1_S2.tif]

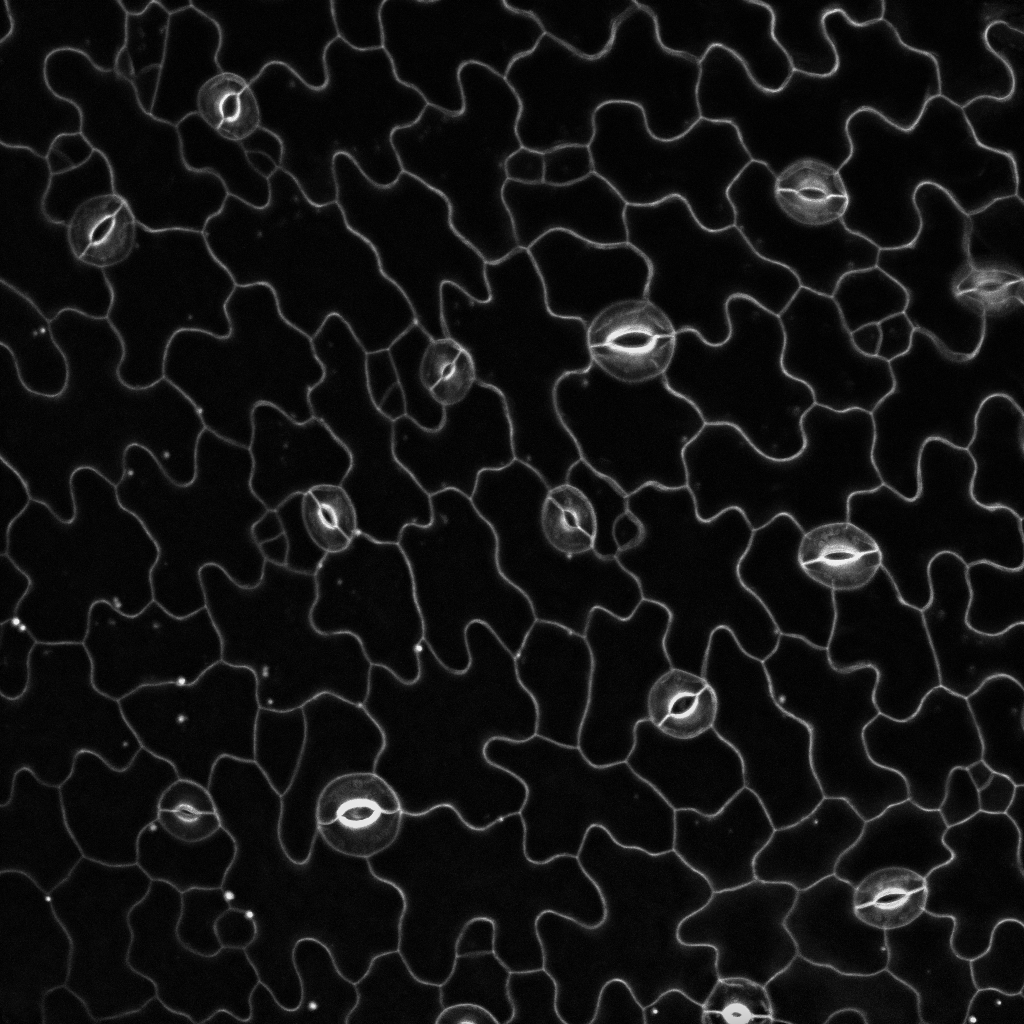

Supplement: Supplementary file 4 — Source Data [file 41467_2020_20730_MOESM4_ESM.zip › SourceData/Figure7_ShapeMutants/Images/lue1/lue1_S6.tif]

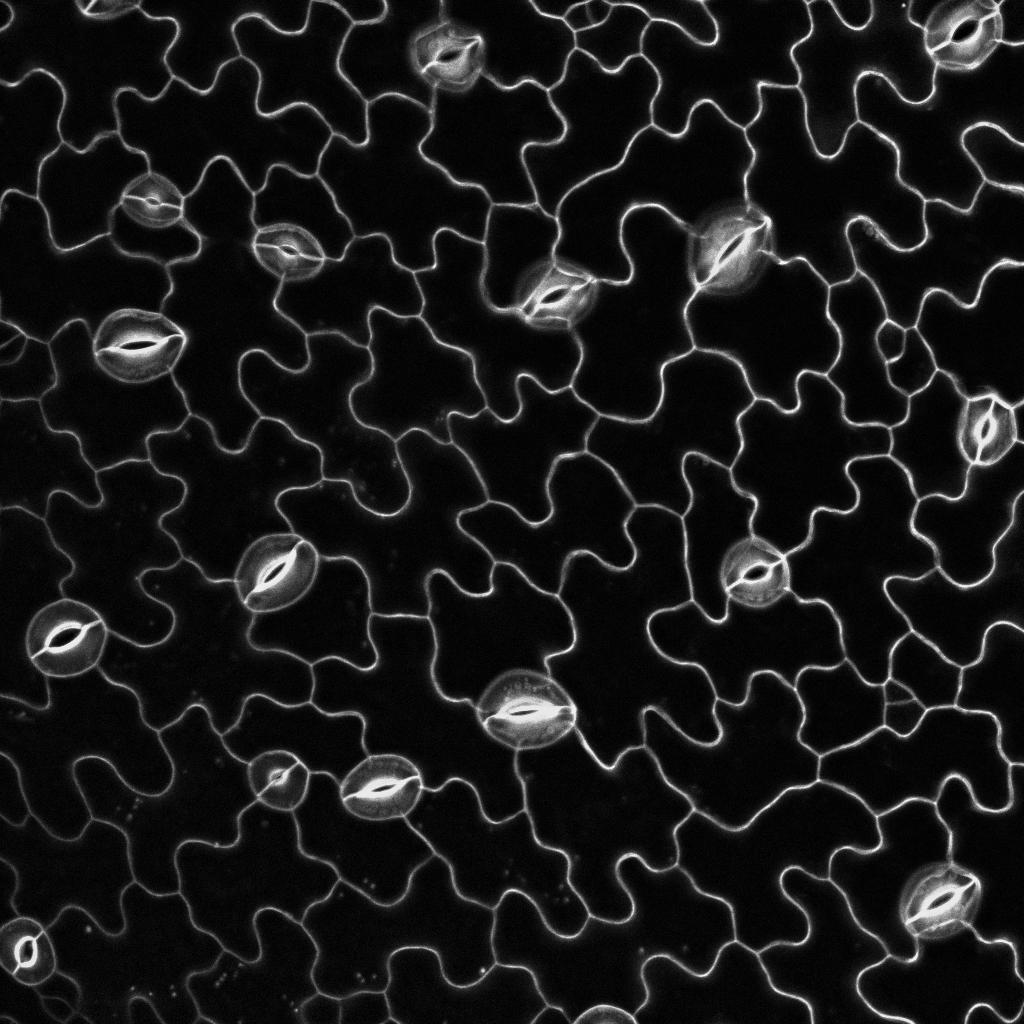

Supplement: Supplementary file 4 — Source Data [file 41467_2020_20730_MOESM4_ESM.zip › SourceData/Figure7_ShapeMutants/Images/lue1/lue1_S5.tif]

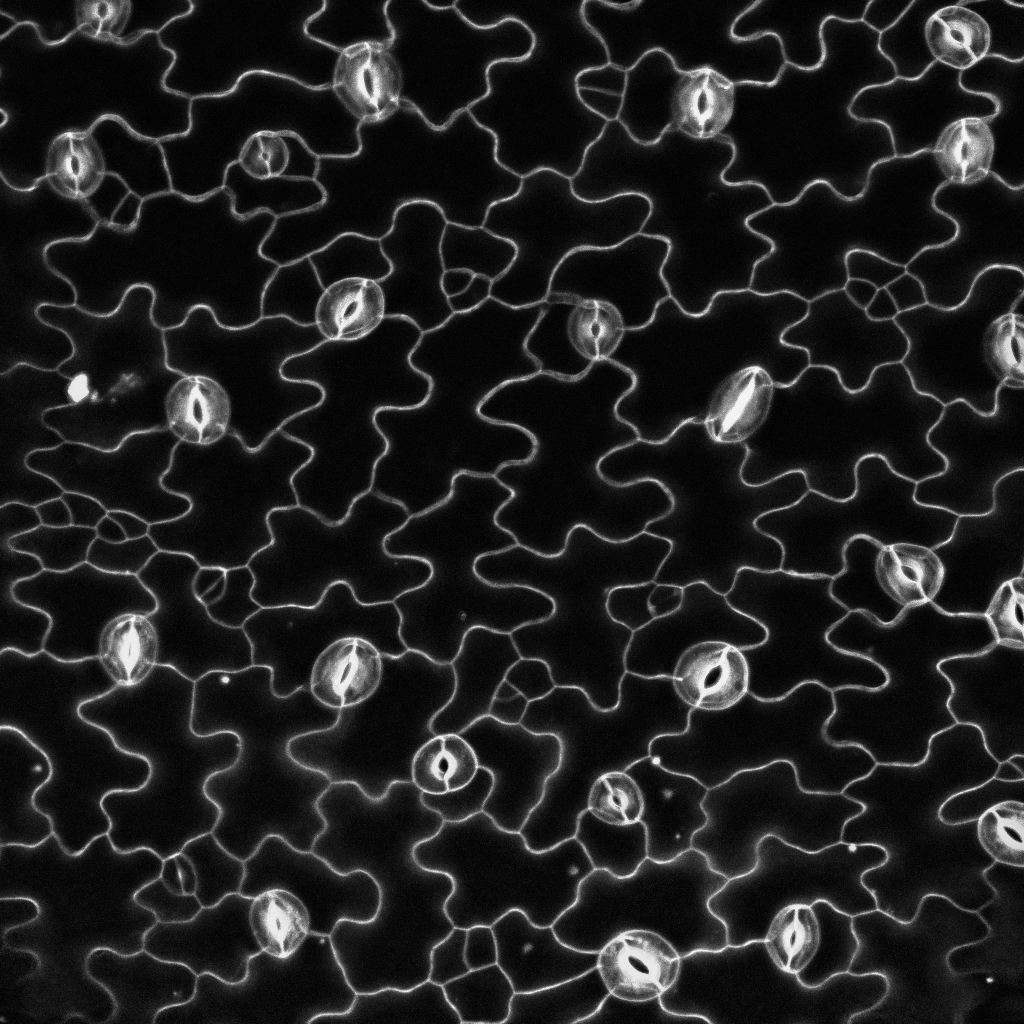

Supplement: Supplementary file 4 — Source Data [file 41467_2020_20730_MOESM4_ESM.zip › SourceData/Figure7_ShapeMutants/Images/lue1/lue1_S4.tif]

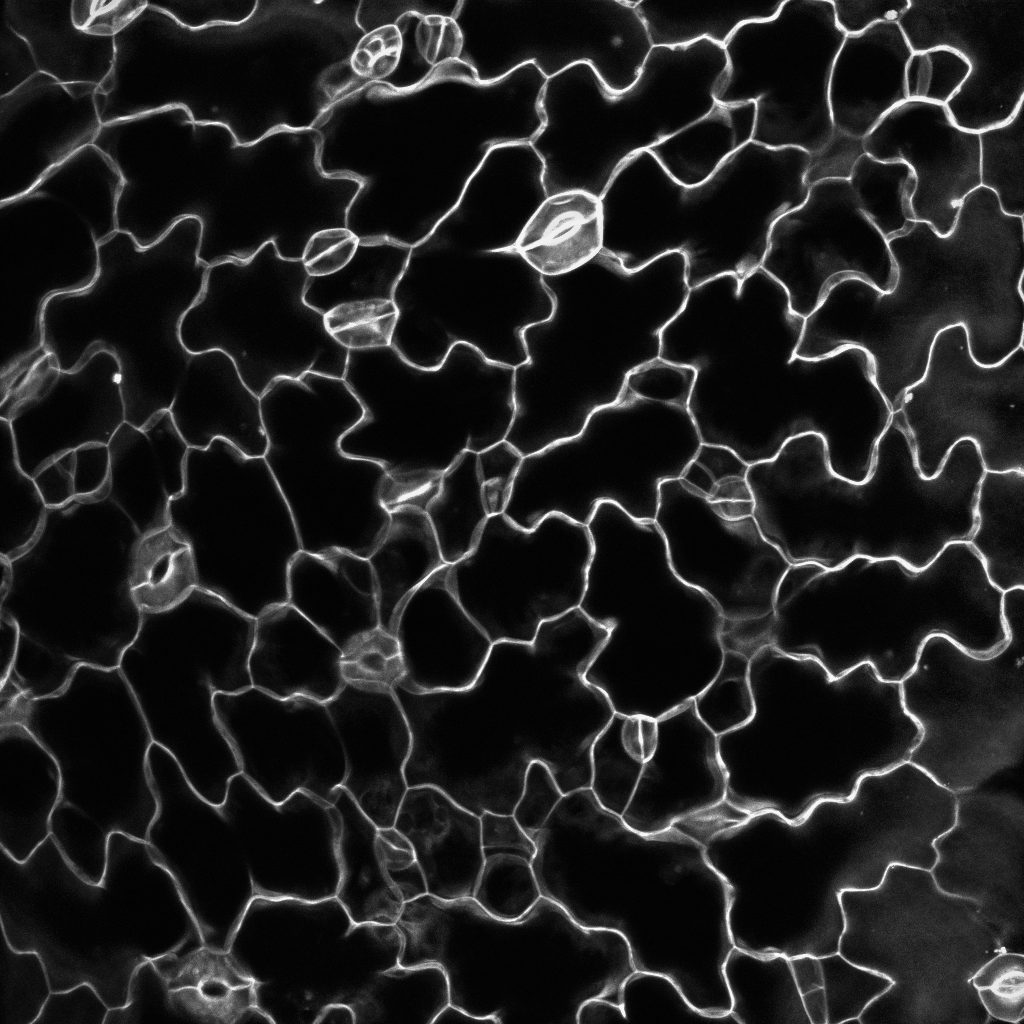

Supplement: Supplementary file 4 — Source Data [file 41467_2020_20730_MOESM4_ESM.zip › SourceData/Figure7_ShapeMutants/Images/clasp-1/clasp-1_S2.tif]

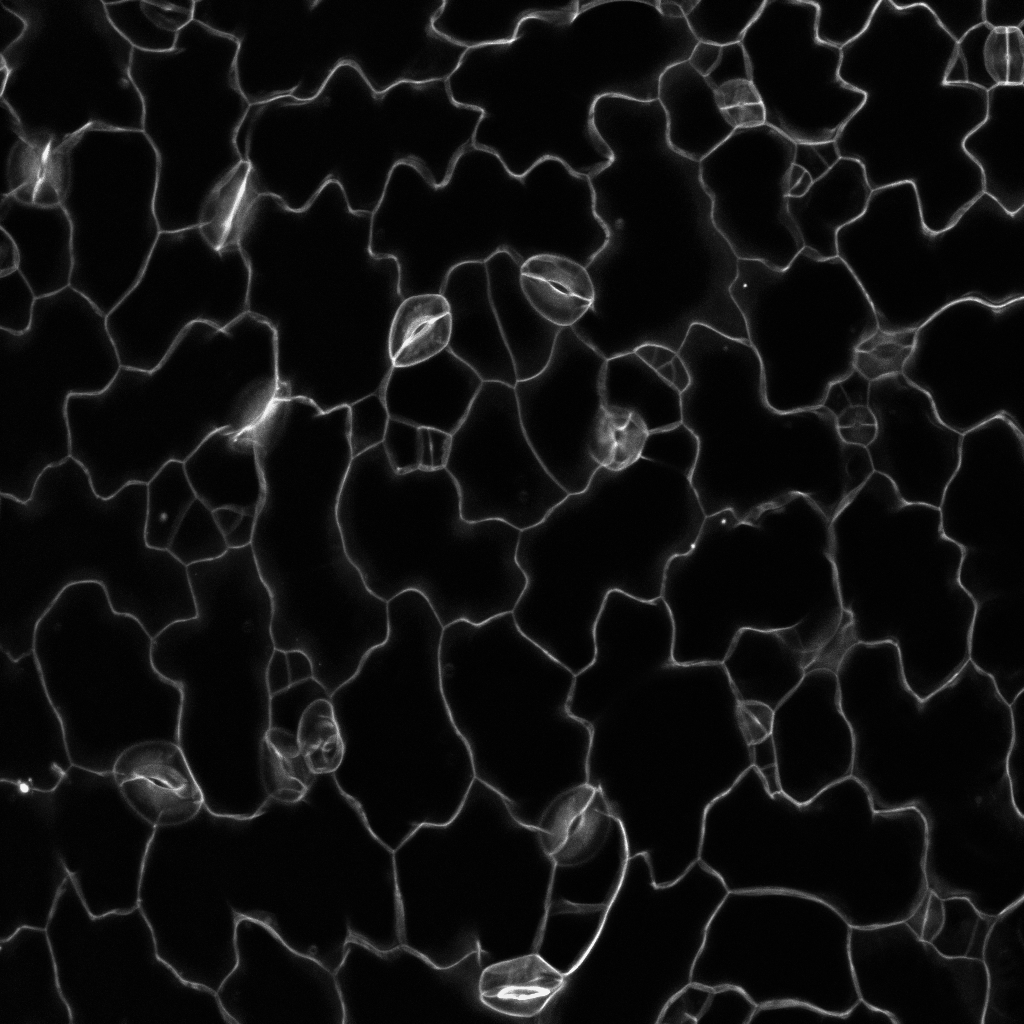

Supplement: Supplementary file 4 — Source Data [file 41467_2020_20730_MOESM4_ESM.zip › SourceData/Figure7_ShapeMutants/Images/clasp-1/clasp-1_S3.tif]

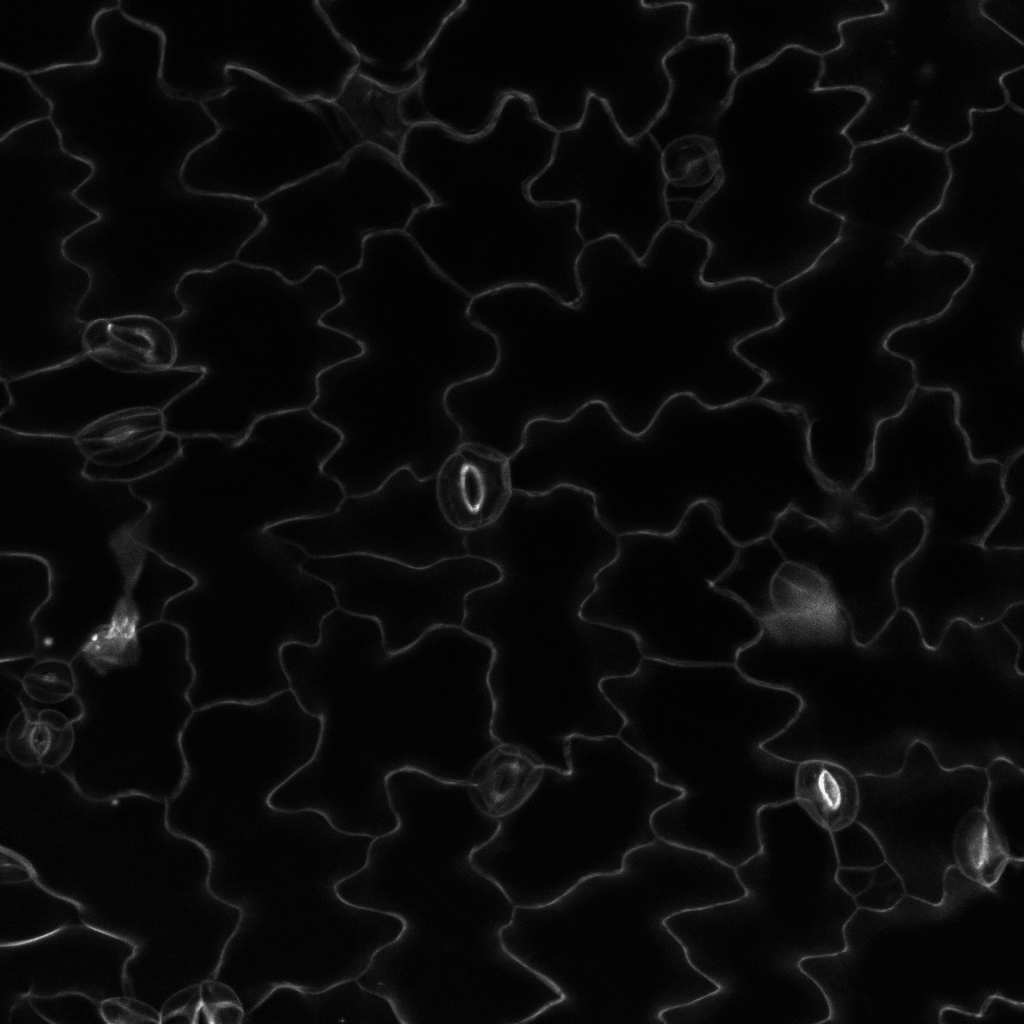

Supplement: Supplementary file 4 — Source Data [file 41467_2020_20730_MOESM4_ESM.zip › SourceData/Figure7_ShapeMutants/Images/clasp-1/clasp-1_S1.tif]

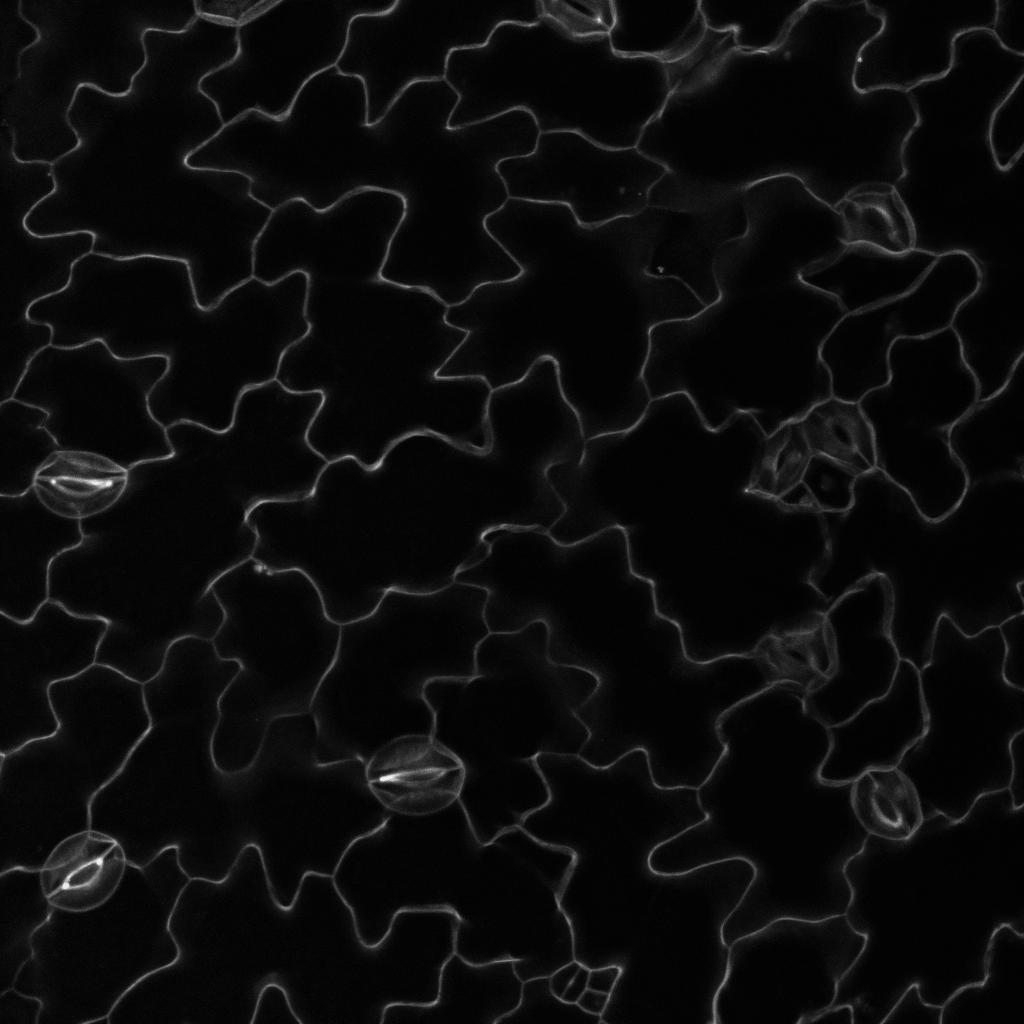

Supplement: Supplementary file 4 — Source Data [file 41467_2020_20730_MOESM4_ESM.zip › SourceData/Figure7_ShapeMutants/Images/clasp-1/clasp-1_S4.tif]

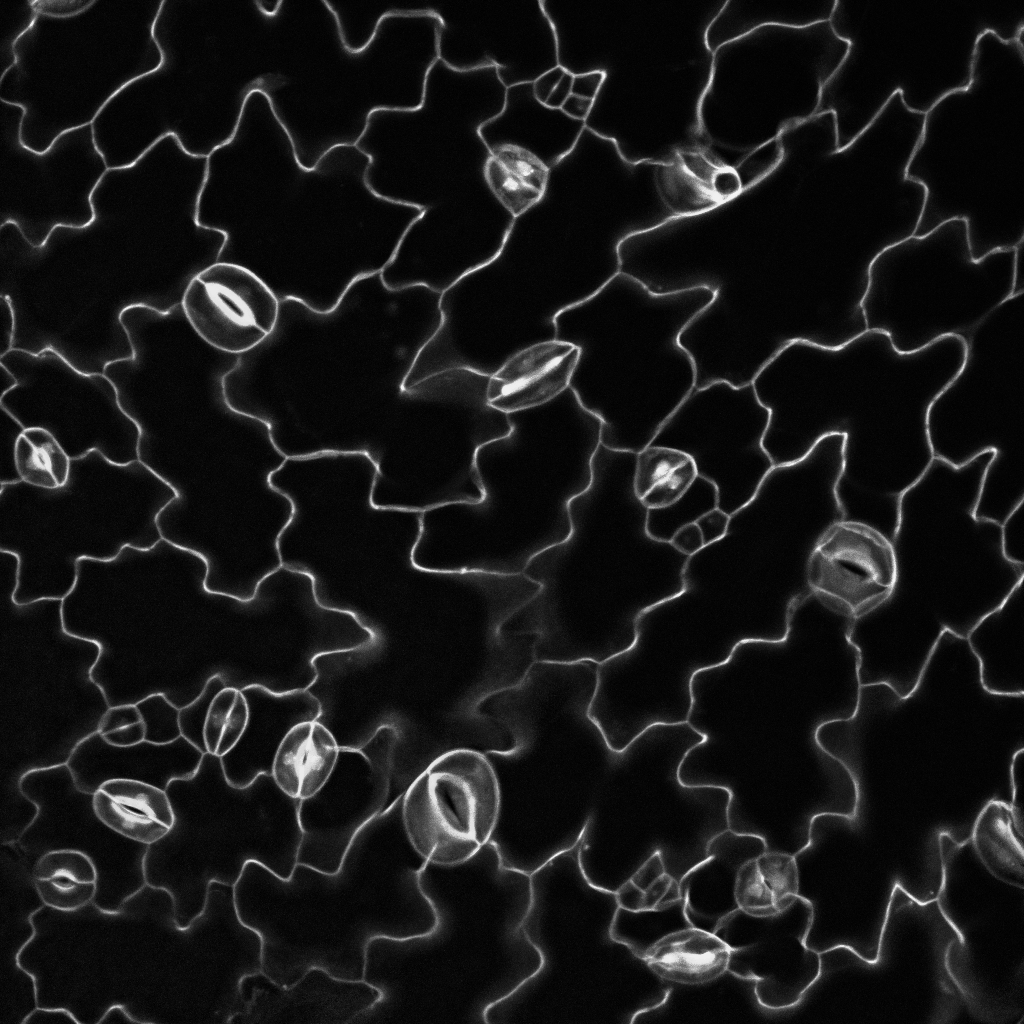

Supplement: Supplementary file 4 — Source Data [file 41467_2020_20730_MOESM4_ESM.zip › SourceData/Figure7_ShapeMutants/Images/clasp-1/clasp-1_S5.tif]

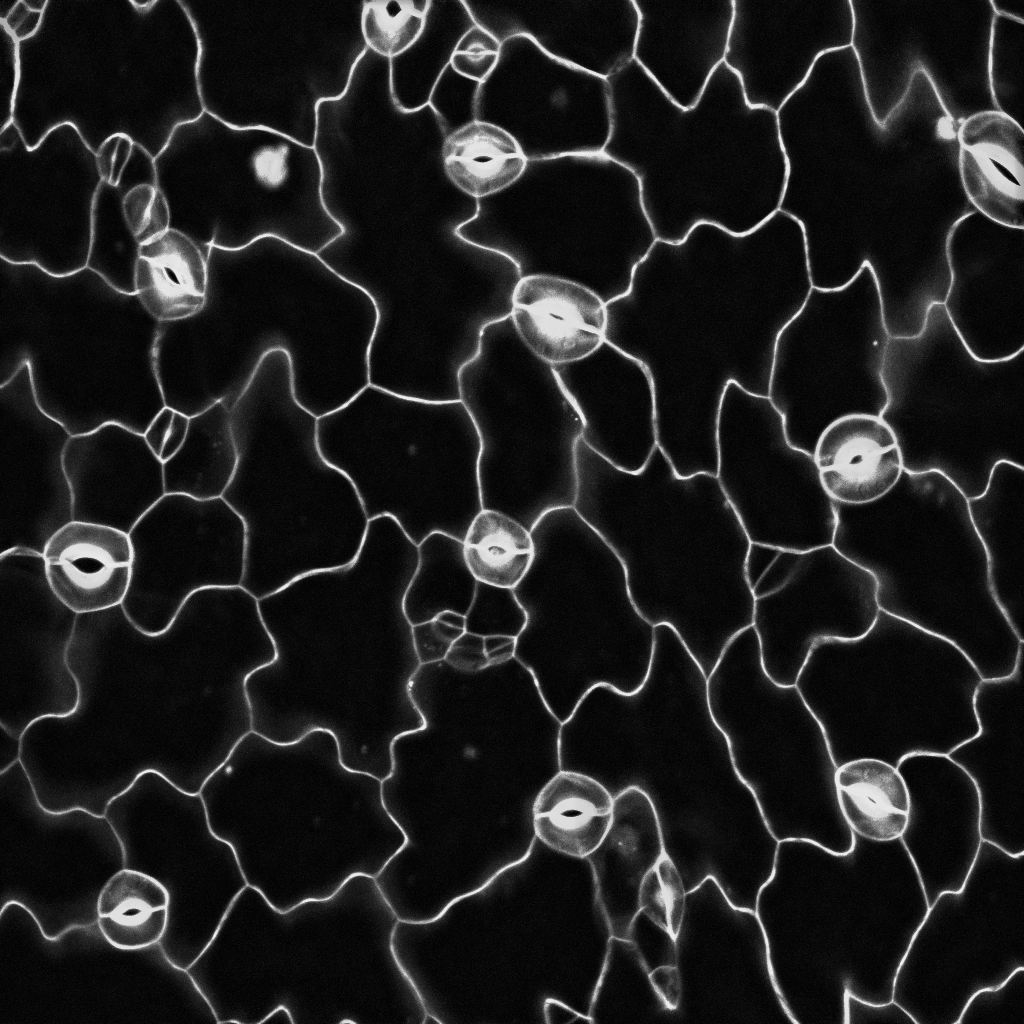

Supplement: Supplementary file 4 — Source Data [file 41467_2020_20730_MOESM4_ESM.zip › SourceData/Figure7_ShapeMutants/Images/clasp-1/clasp-1_S6.tif]

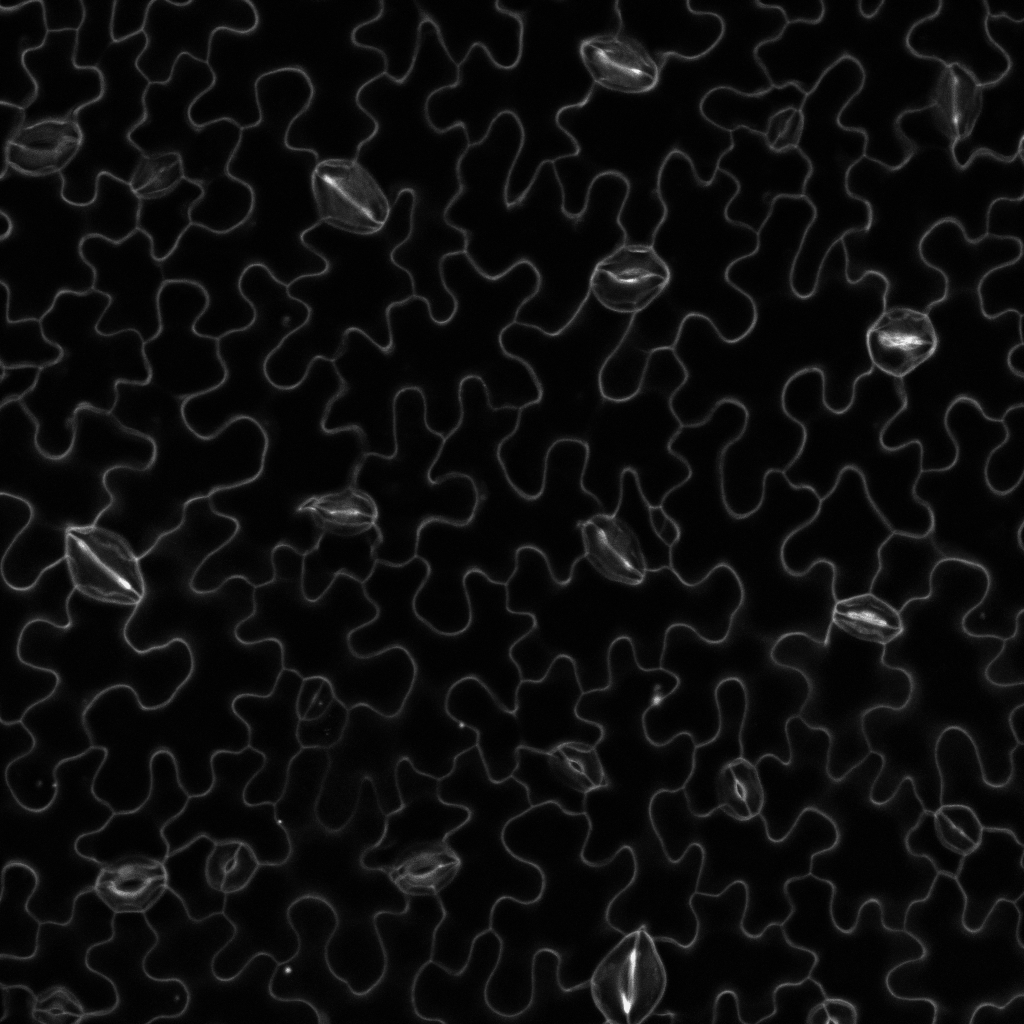

Supplement: Supplementary file 4 — Source Data [file 41467_2020_20730_MOESM4_ESM.zip › SourceData/Figure7_ShapeMutants/Images/Ws/Ws_S6.tif]

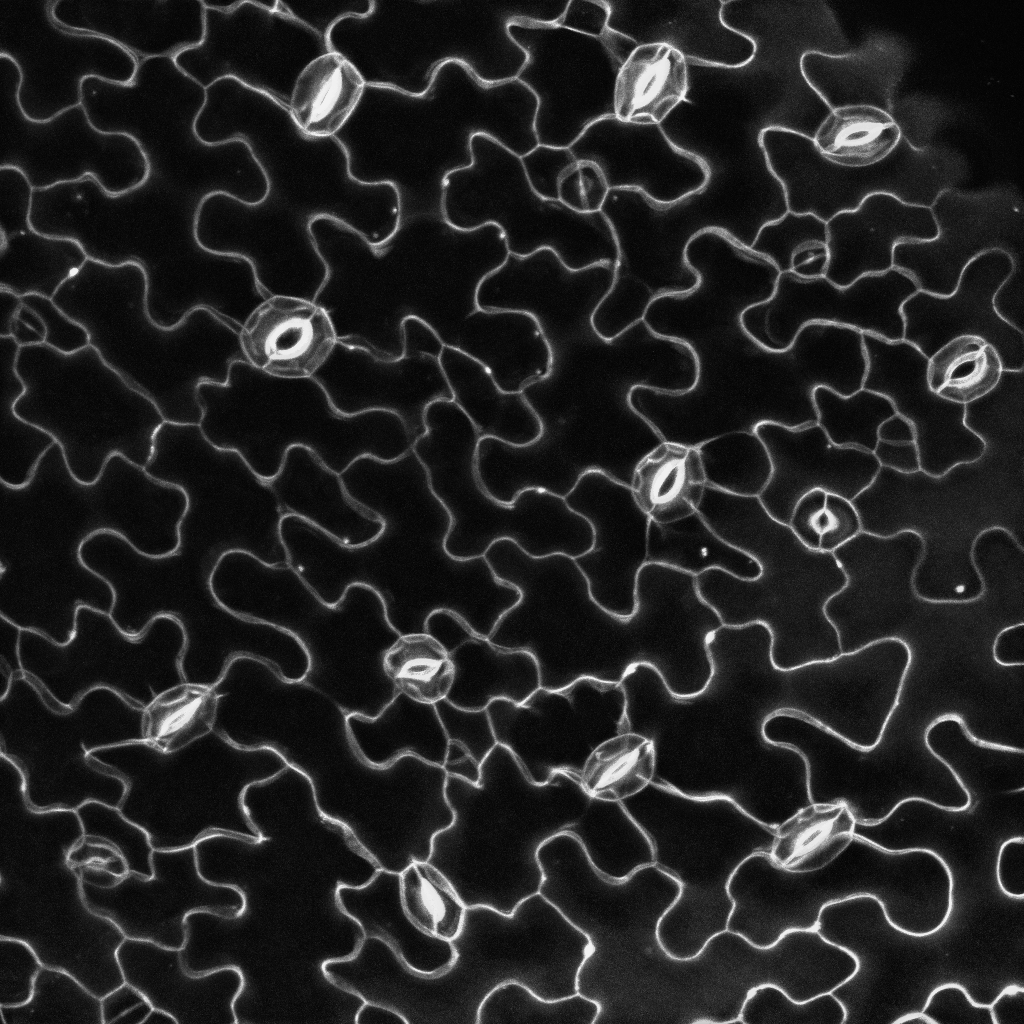

Supplement: Supplementary file 4 — Source Data [file 41467_2020_20730_MOESM4_ESM.zip › SourceData/Figure7_ShapeMutants/Images/Ws/Ws_S4.tif]

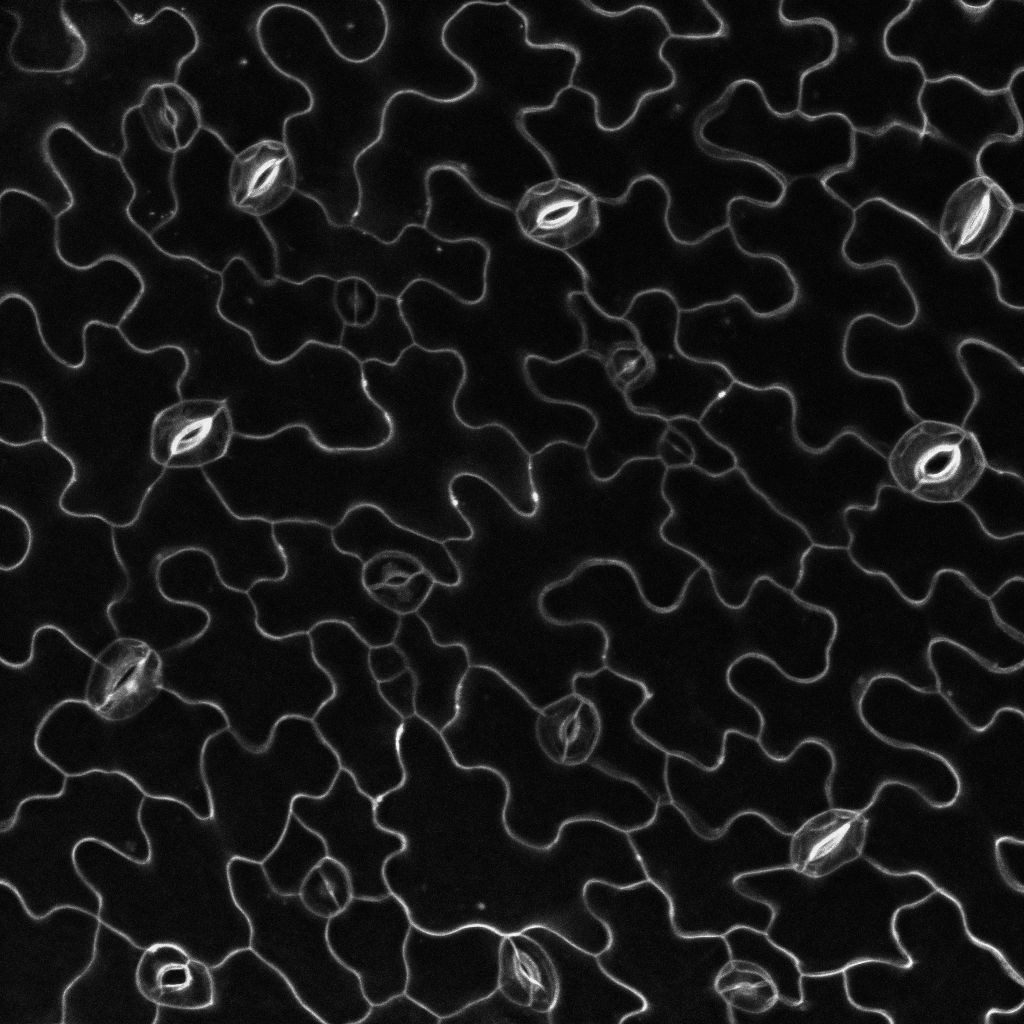

Supplement: Supplementary file 4 — Source Data [file 41467_2020_20730_MOESM4_ESM.zip › SourceData/Figure7_ShapeMutants/Images/Ws/Ws_S5.tif]

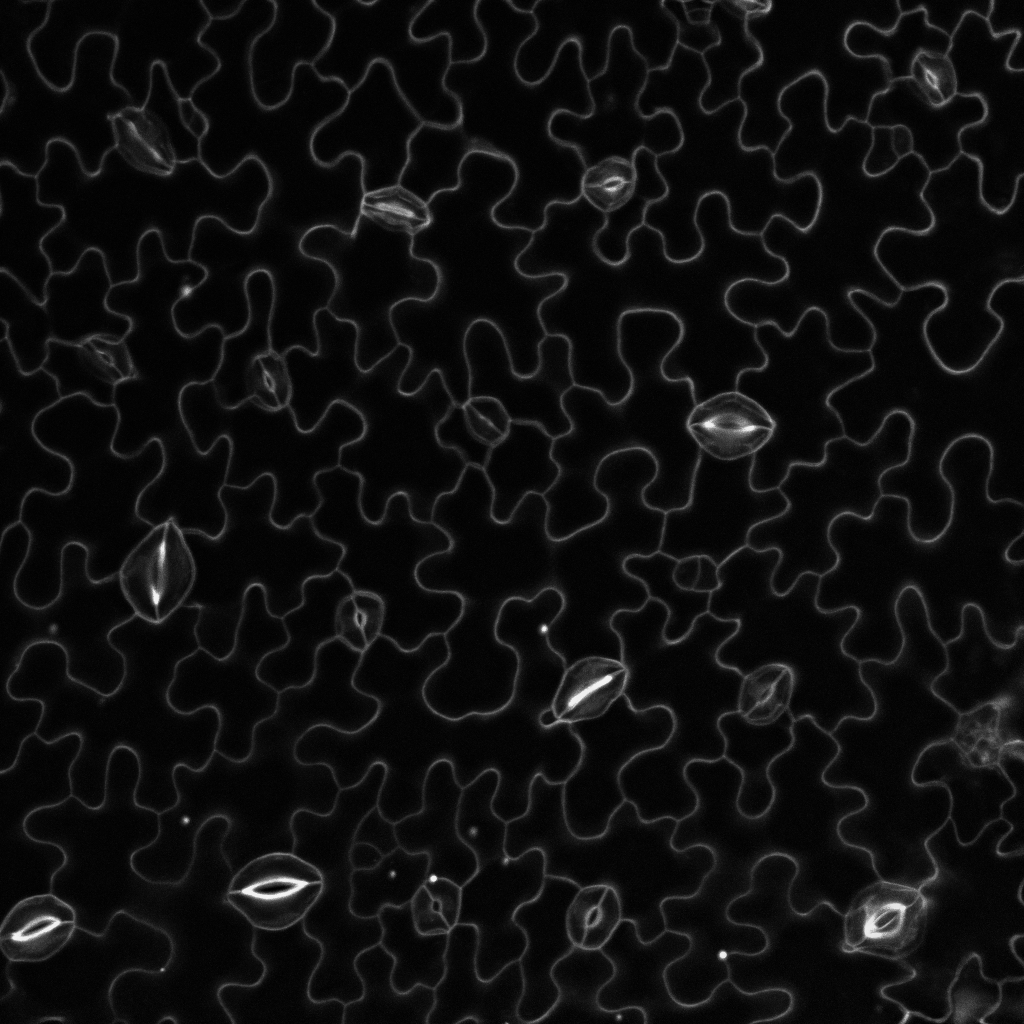

Supplement: Supplementary file 4 — Source Data [file 41467_2020_20730_MOESM4_ESM.zip › SourceData/Figure7_ShapeMutants/Images/Ws/Ws_S1.tif]

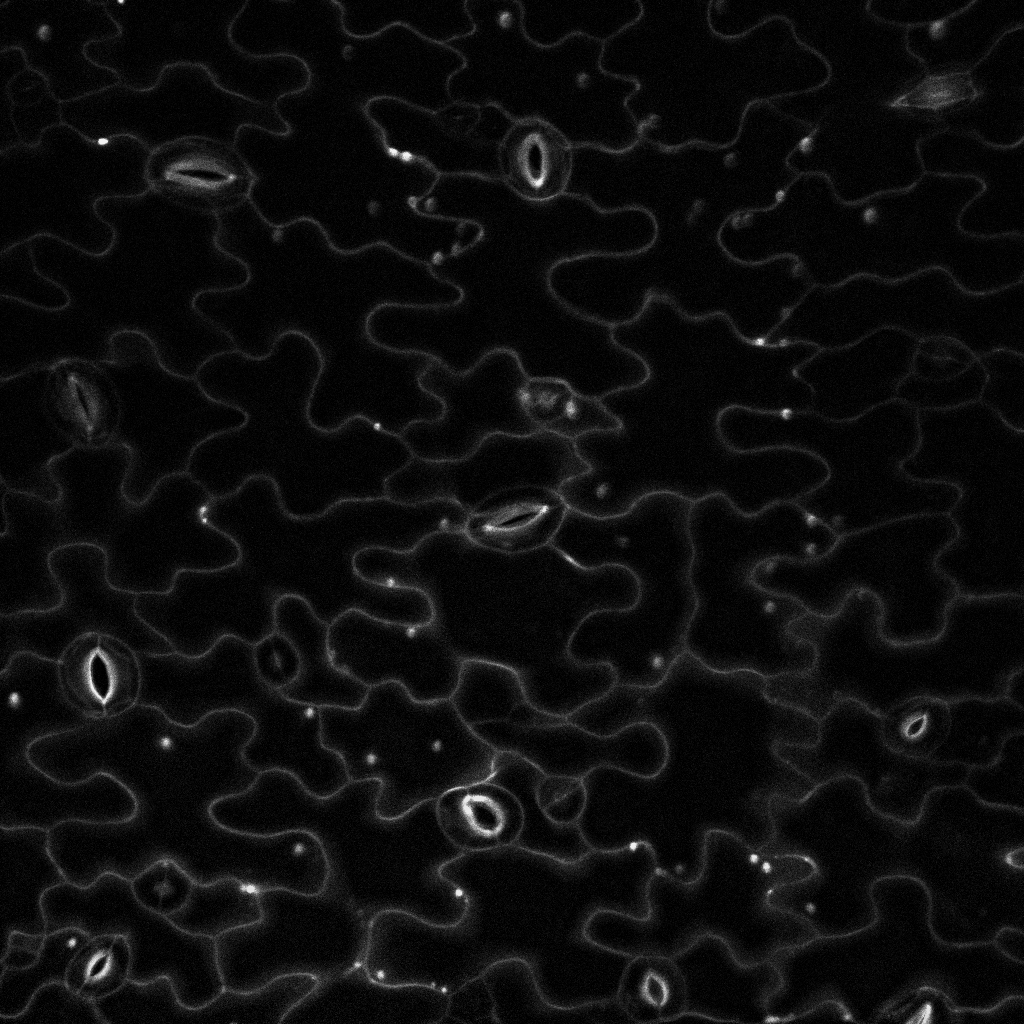

Supplement: Supplementary file 4 — Source Data [file 41467_2020_20730_MOESM4_ESM.zip › SourceData/Figure7_ShapeMutants/Images/Ws/Ws_S2.tif]

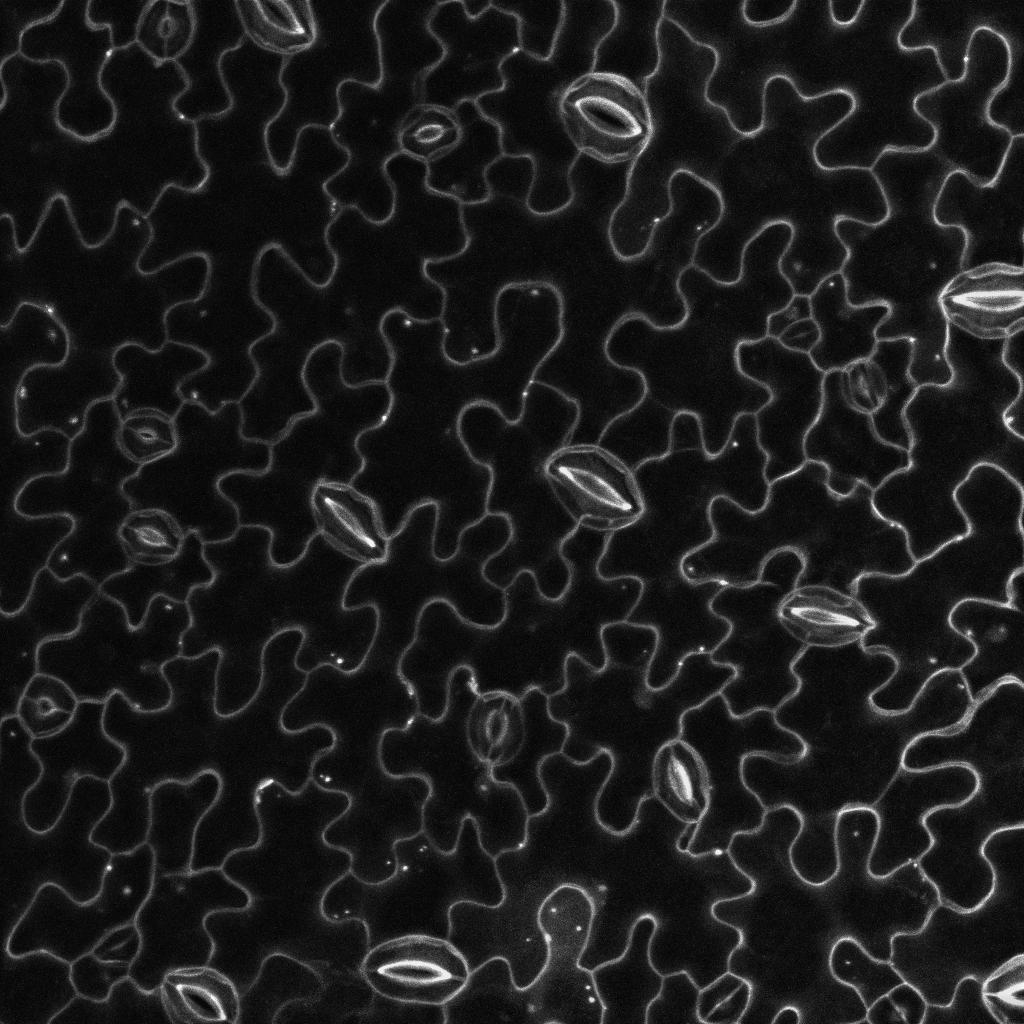

Supplement: Supplementary file 4 — Source Data [file 41467_2020_20730_MOESM4_ESM.zip › SourceData/Figure7_ShapeMutants/Images/Ws/Ws_S3.tif]

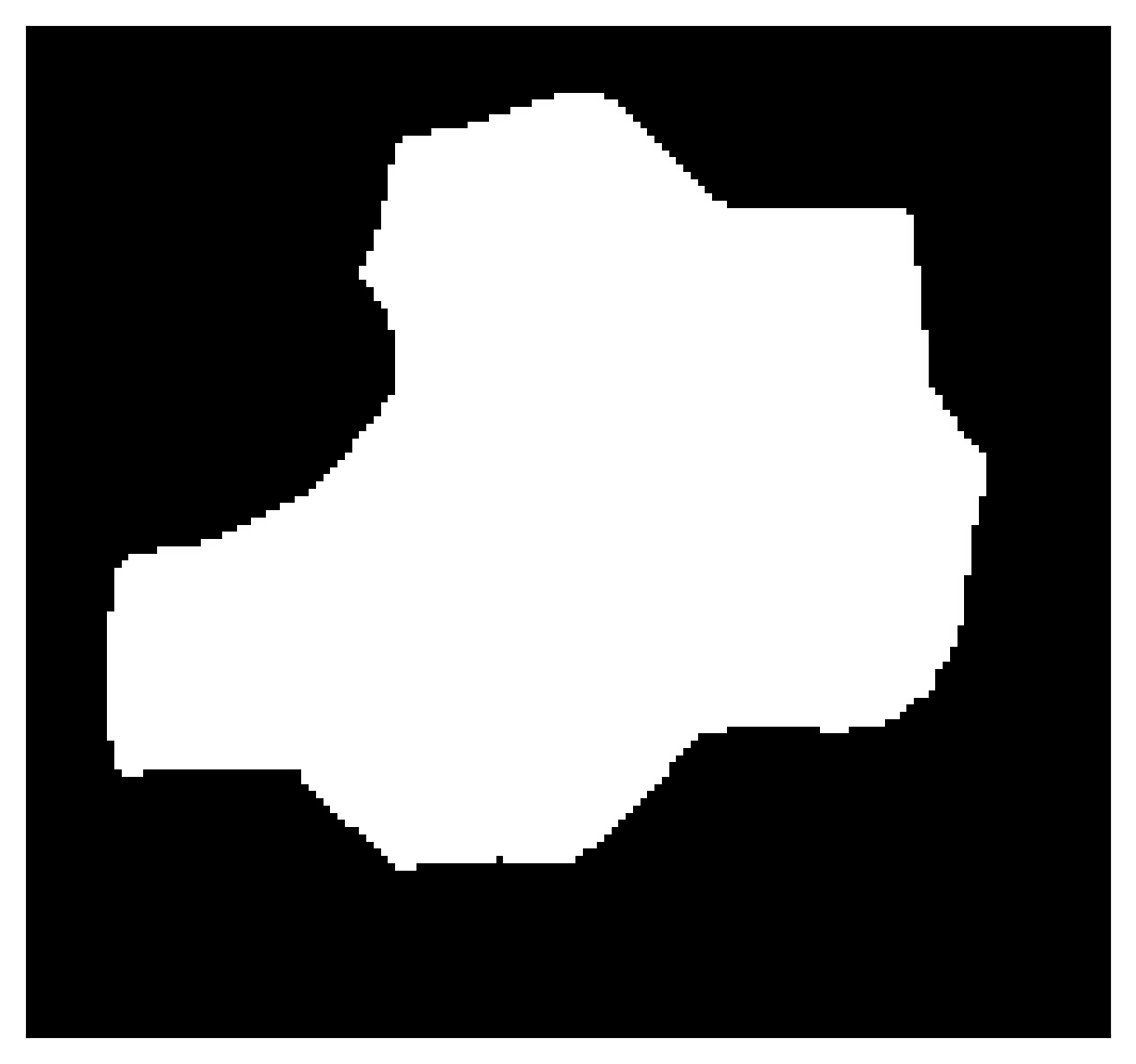

Supplement: Supplementary file 4 — Source Data [file 41467_2020_20730_MOESM4_ESM.zip › SourceData/Figure1_VisibilityGraphs/Fig1_cellBinary.png]

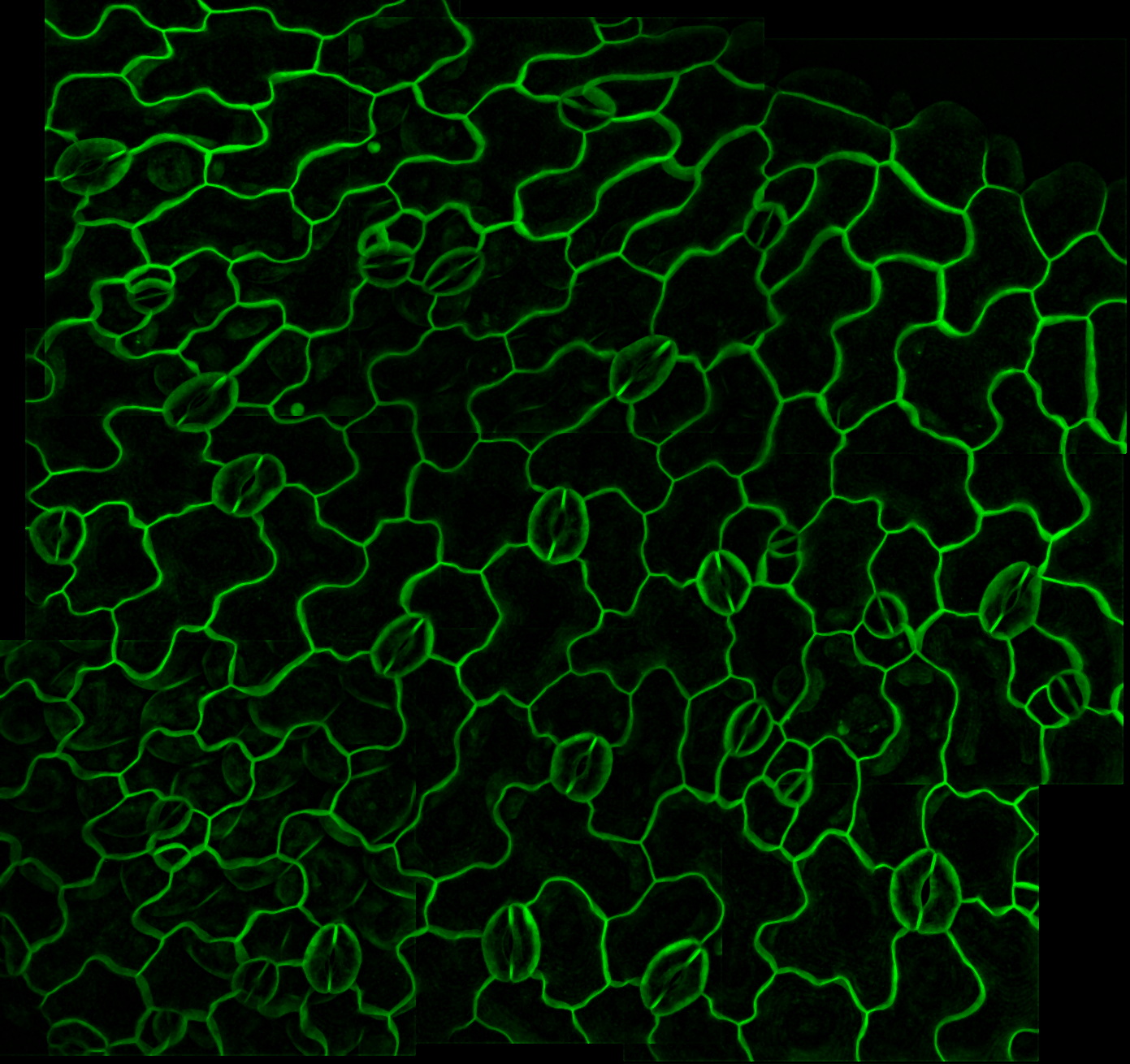

Supplement: Supplementary file 4 — Source Data [file 41467_2020_20730_MOESM4_ESM.zip › SourceData/FigureS12_GraVisOutput/WT_96h-GFP.tif]

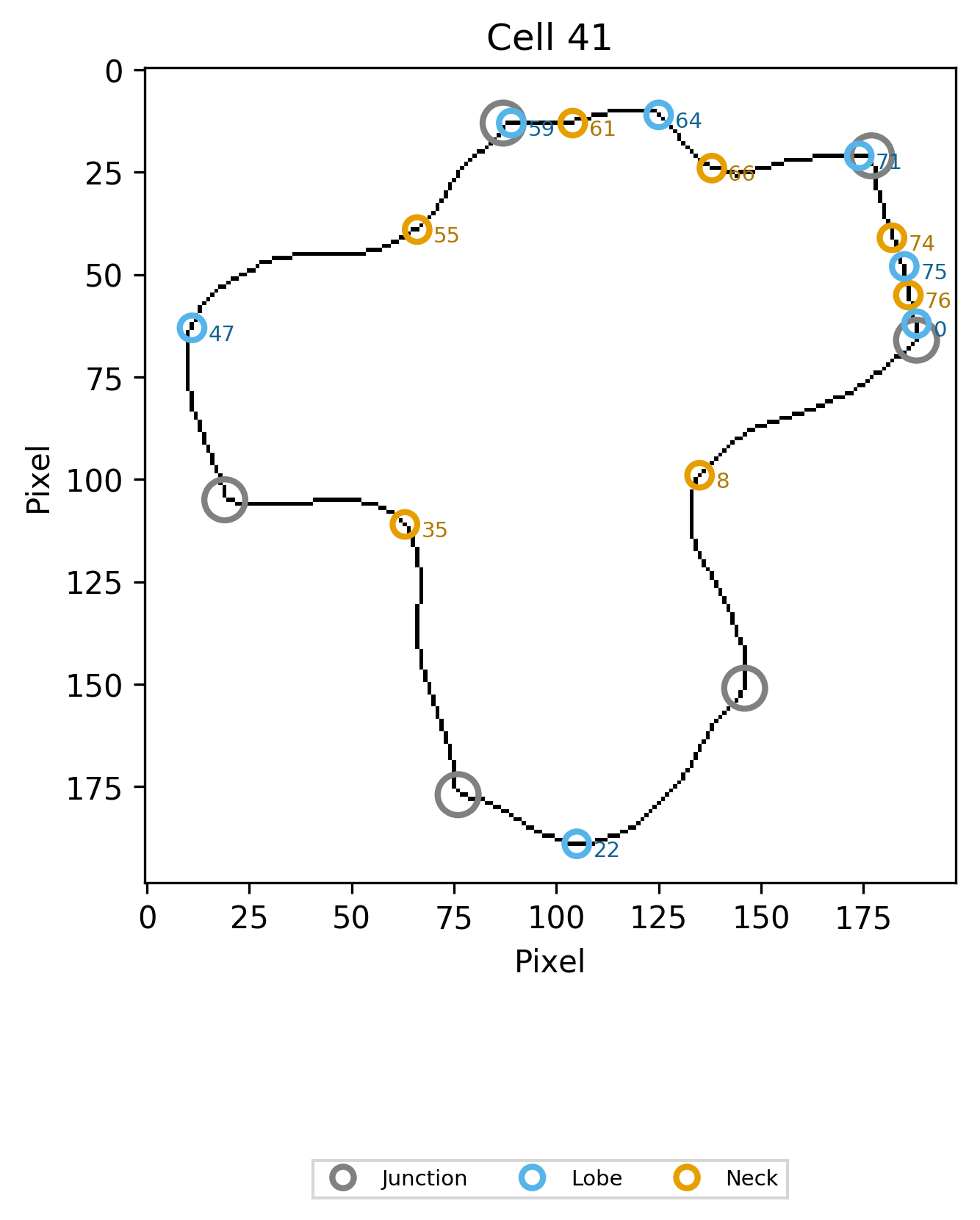

Supplement: Supplementary file 4 — Source Data [file 41467_2020_20730_MOESM4_ESM.zip › SourceData/FigureS12_GraVisOutput/Cell41_detectedFeatures.png]

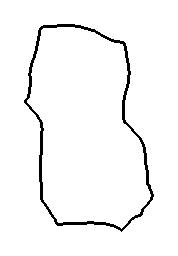

Supplement: Supplementary file 4 — Source Data [file 41467_2020_20730_MOESM4_ESM.zip › SourceData/Figure6_S13_S15_S19_LobeDetection/oryzalin_GoldStandard/Cell16.jpg]

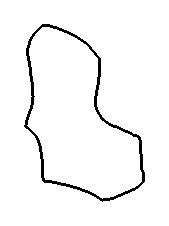

Supplement: Supplementary file 4 — Source Data [file 41467_2020_20730_MOESM4_ESM.zip › SourceData/Figure6_S13_S15_S19_LobeDetection/oryzalin_GoldStandard/Cell1.jpg]

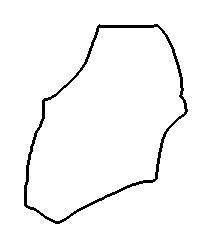

Supplement: Supplementary file 4 — Source Data [file 41467_2020_20730_MOESM4_ESM.zip › SourceData/Figure6_S13_S15_S19_LobeDetection/oryzalin_GoldStandard/Cell29.jpg]

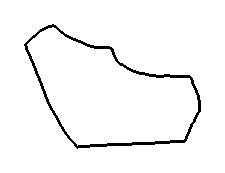

Supplement: Supplementary file 4 — Source Data [file 41467_2020_20730_MOESM4_ESM.zip › SourceData/Figure6_S13_S15_S19_LobeDetection/oryzalin_GoldStandard/Cell11.jpg]

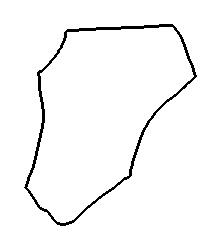

Supplement: Supplementary file 4 — Source Data [file 41467_2020_20730_MOESM4_ESM.zip › SourceData/Figure6_S13_S15_S19_LobeDetection/oryzalin_GoldStandard/Cell7.jpg]

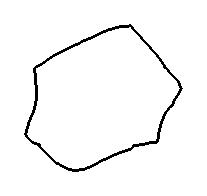

Supplement: Supplementary file 4 — Source Data [file 41467_2020_20730_MOESM4_ESM.zip › SourceData/Figure6_S13_S15_S19_LobeDetection/oryzalin_GoldStandard/Cell13.jpg]

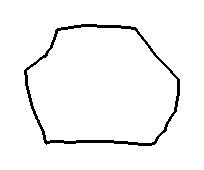

Supplement: Supplementary file 4 — Source Data [file 41467_2020_20730_MOESM4_ESM.zip › SourceData/Figure6_S13_S15_S19_LobeDetection/oryzalin_GoldStandard/Cell8.jpg]

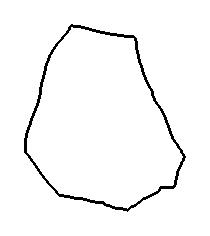

Supplement: Supplementary file 4 — Source Data [file 41467_2020_20730_MOESM4_ESM.zip › SourceData/Figure6_S13_S15_S19_LobeDetection/oryzalin_GoldStandard/Cell19.jpg]

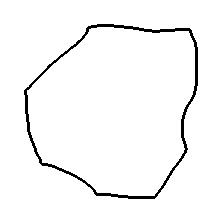

Supplement: Supplementary file 4 — Source Data [file 41467_2020_20730_MOESM4_ESM.zip › SourceData/Figure6_S13_S15_S19_LobeDetection/oryzalin_GoldStandard/Cell25.jpg]

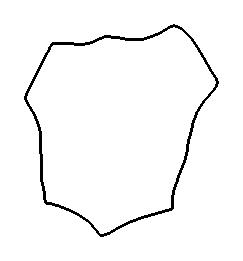

Supplement: Supplementary file 4 — Source Data [file 41467_2020_20730_MOESM4_ESM.zip › SourceData/Figure6_S13_S15_S19_LobeDetection/oryzalin_GoldStandard/Cell27.jpg]

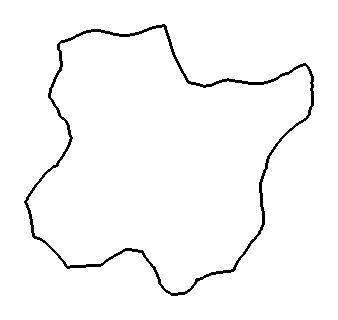

Supplement: Supplementary file 4 — Source Data [file 41467_2020_20730_MOESM4_ESM.zip › SourceData/Figure6_S13_S15_S19_LobeDetection/clasp-1_GoldStandard/Cell17.jpg]

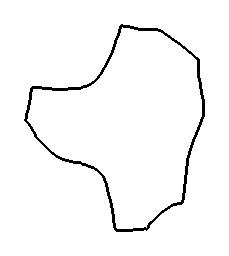

Supplement: Supplementary file 4 — Source Data [file 41467_2020_20730_MOESM4_ESM.zip › SourceData/Figure6_S13_S15_S19_LobeDetection/clasp-1_GoldStandard/Cell2.jpg]

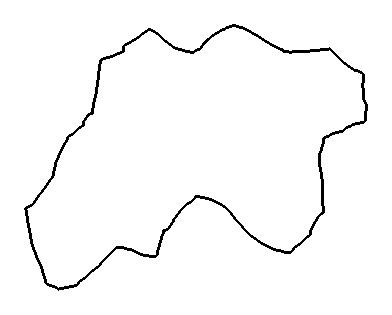

Supplement: Supplementary file 4 — Source Data [file 41467_2020_20730_MOESM4_ESM.zip › SourceData/Figure6_S13_S15_S19_LobeDetection/clasp-1_GoldStandard/Cell3.jpg]

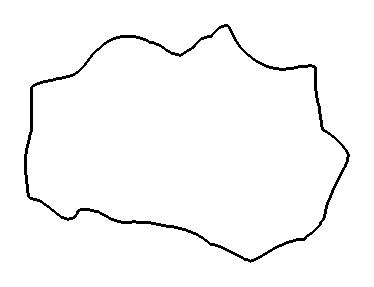

Supplement: Supplementary file 4 — Source Data [file 41467_2020_20730_MOESM4_ESM.zip › SourceData/Figure6_S13_S15_S19_LobeDetection/clasp-1_GoldStandard/Cell28.jpg]

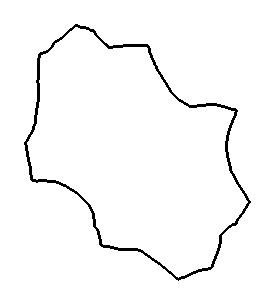

Supplement: Supplementary file 4 — Source Data [file 41467_2020_20730_MOESM4_ESM.zip › SourceData/Figure6_S13_S15_S19_LobeDetection/clasp-1_GoldStandard/Cell12.jpg]

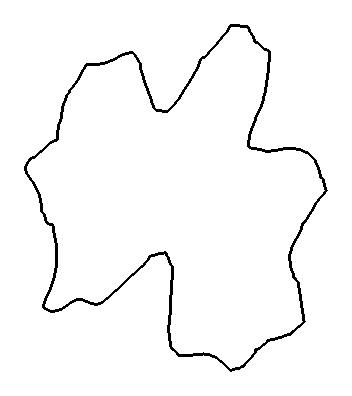

Supplement: Supplementary file 4 — Source Data [file 41467_2020_20730_MOESM4_ESM.zip › SourceData/Figure6_S13_S15_S19_LobeDetection/clasp-1_GoldStandard/Cell6.jpg]

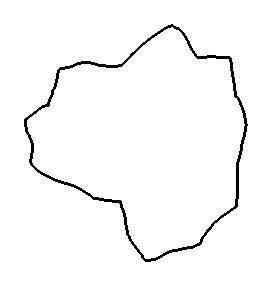

Supplement: Supplementary file 4 — Source Data [file 41467_2020_20730_MOESM4_ESM.zip › SourceData/Figure6_S13_S15_S19_LobeDetection/clasp-1_GoldStandard/Cell23.jpg]

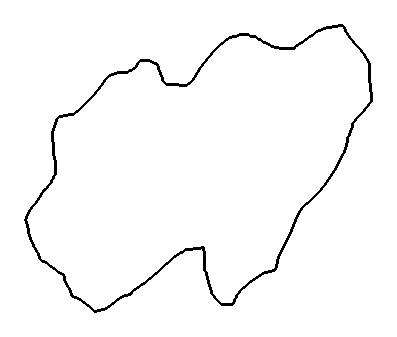

Supplement: Supplementary file 4 — Source Data [file 41467_2020_20730_MOESM4_ESM.zip › SourceData/Figure6_S13_S15_S19_LobeDetection/clasp-1_GoldStandard/Cell24.jpg]

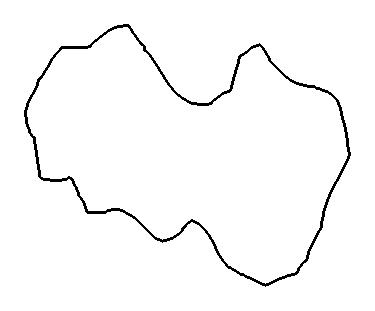

Supplement: Supplementary file 4 — Source Data [file 41467_2020_20730_MOESM4_ESM.zip › SourceData/Figure6_S13_S15_S19_LobeDetection/clasp-1_GoldStandard/Cell18.jpg]

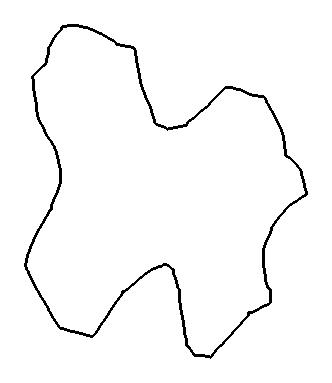

Supplement: Supplementary file 4 — Source Data [file 41467_2020_20730_MOESM4_ESM.zip › SourceData/Figure6_S13_S15_S19_LobeDetection/clasp-1_GoldStandard/Cell26.jpg]

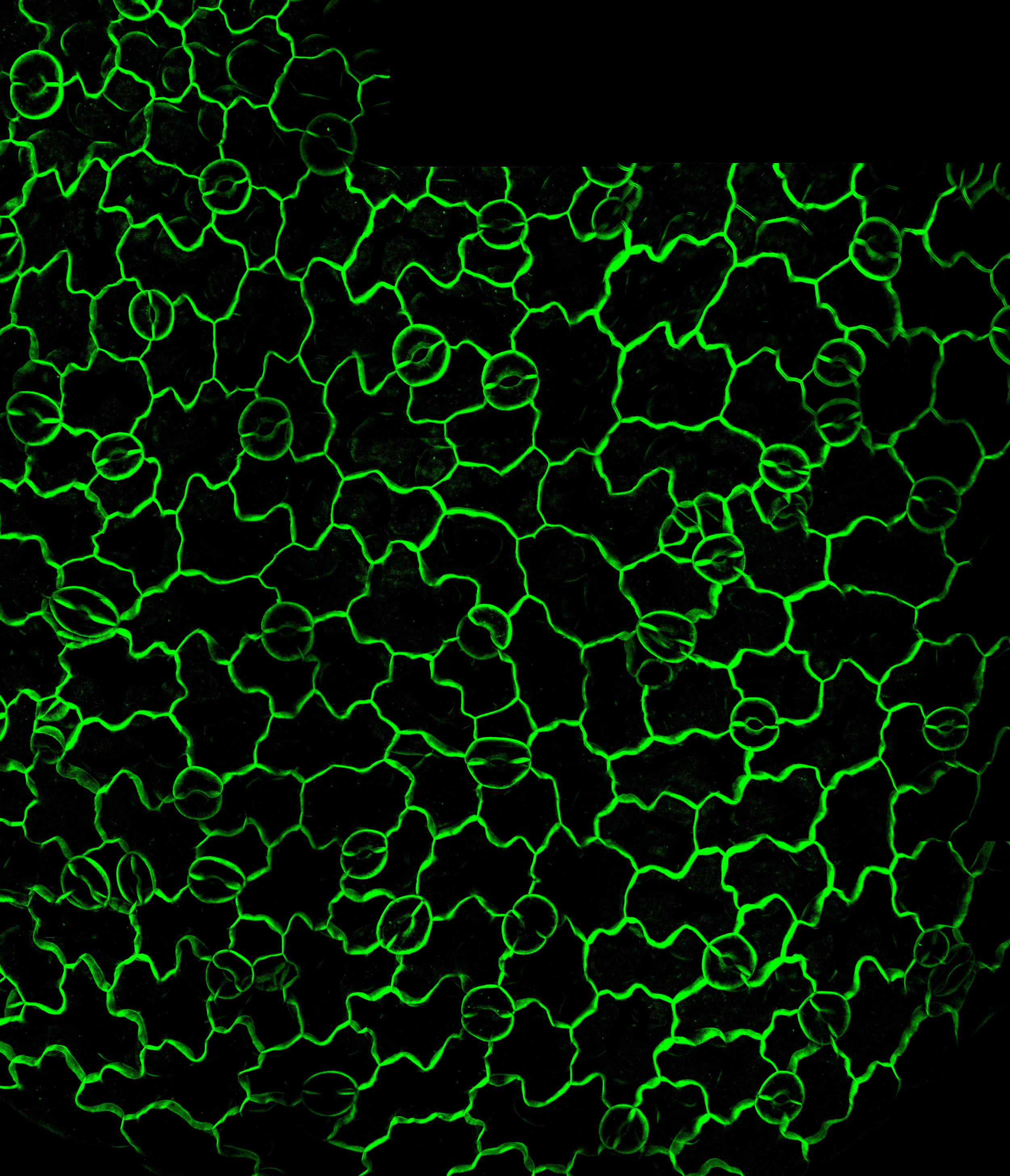

Supplement: Supplementary file 4 — Source Data [file 41467_2020_20730_MOESM4_ESM.zip › SourceData/Figure6_S13_S15_S19_LobeDetection/clasp-1_96h-GFP.tif]

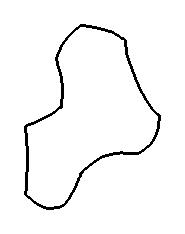

Supplement: Supplementary file 4 — Source Data [file 41467_2020_20730_MOESM4_ESM.zip › SourceData/Figure6_S13_S15_S19_LobeDetection/wt_GoldStandard/Cell14.jpg]

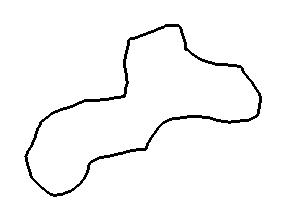

Supplement: Supplementary file 4 — Source Data [file 41467_2020_20730_MOESM4_ESM.zip › SourceData/Figure6_S13_S15_S19_LobeDetection/wt_GoldStandard/Cell15.jpg]

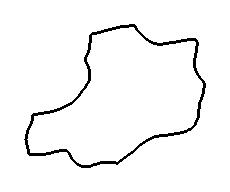

Supplement: Supplementary file 4 — Source Data [file 41467_2020_20730_MOESM4_ESM.zip › SourceData/Figure6_S13_S15_S19_LobeDetection/wt_GoldStandard/Cell4.jpg]

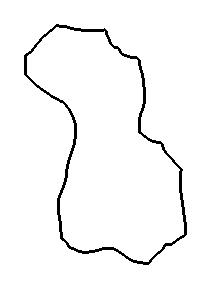

Supplement: Supplementary file 4 — Source Data [file 41467_2020_20730_MOESM4_ESM.zip › SourceData/Figure6_S13_S15_S19_LobeDetection/wt_GoldStandard/Cell5.jpg]

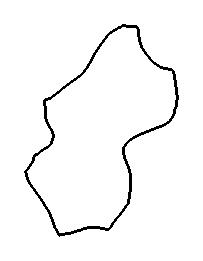

Supplement: Supplementary file 4 — Source Data [file 41467_2020_20730_MOESM4_ESM.zip › SourceData/Figure6_S13_S15_S19_LobeDetection/wt_GoldStandard/Cell10.jpg]

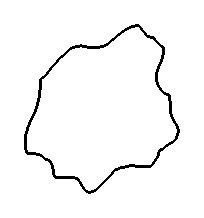

Supplement: Supplementary file 4 — Source Data [file 41467_2020_20730_MOESM4_ESM.zip › SourceData/Figure6_S13_S15_S19_LobeDetection/wt_GoldStandard/Cell22.jpg]

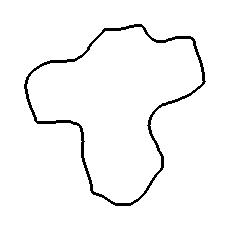

Supplement: Supplementary file 4 — Source Data [file 41467_2020_20730_MOESM4_ESM.zip › SourceData/Figure6_S13_S15_S19_LobeDetection/wt_GoldStandard/Cell21.jpg]

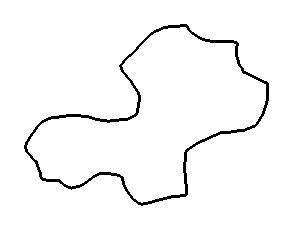

Supplement: Supplementary file 4 — Source Data [file 41467_2020_20730_MOESM4_ESM.zip › SourceData/Figure6_S13_S15_S19_LobeDetection/wt_GoldStandard/Cell9.jpg]

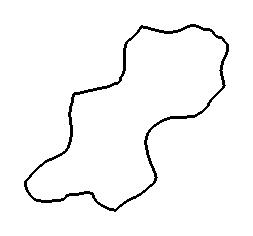

Supplement: Supplementary file 4 — Source Data [file 41467_2020_20730_MOESM4_ESM.zip › SourceData/Figure6_S13_S15_S19_LobeDetection/wt_GoldStandard/Cell20.jpg]

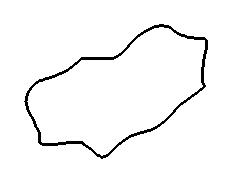

Supplement: Supplementary file 4 — Source Data [file 41467_2020_20730_MOESM4_ESM.zip › SourceData/Figure6_S13_S15_S19_LobeDetection/wt_GoldStandard/Cell30.jpg]

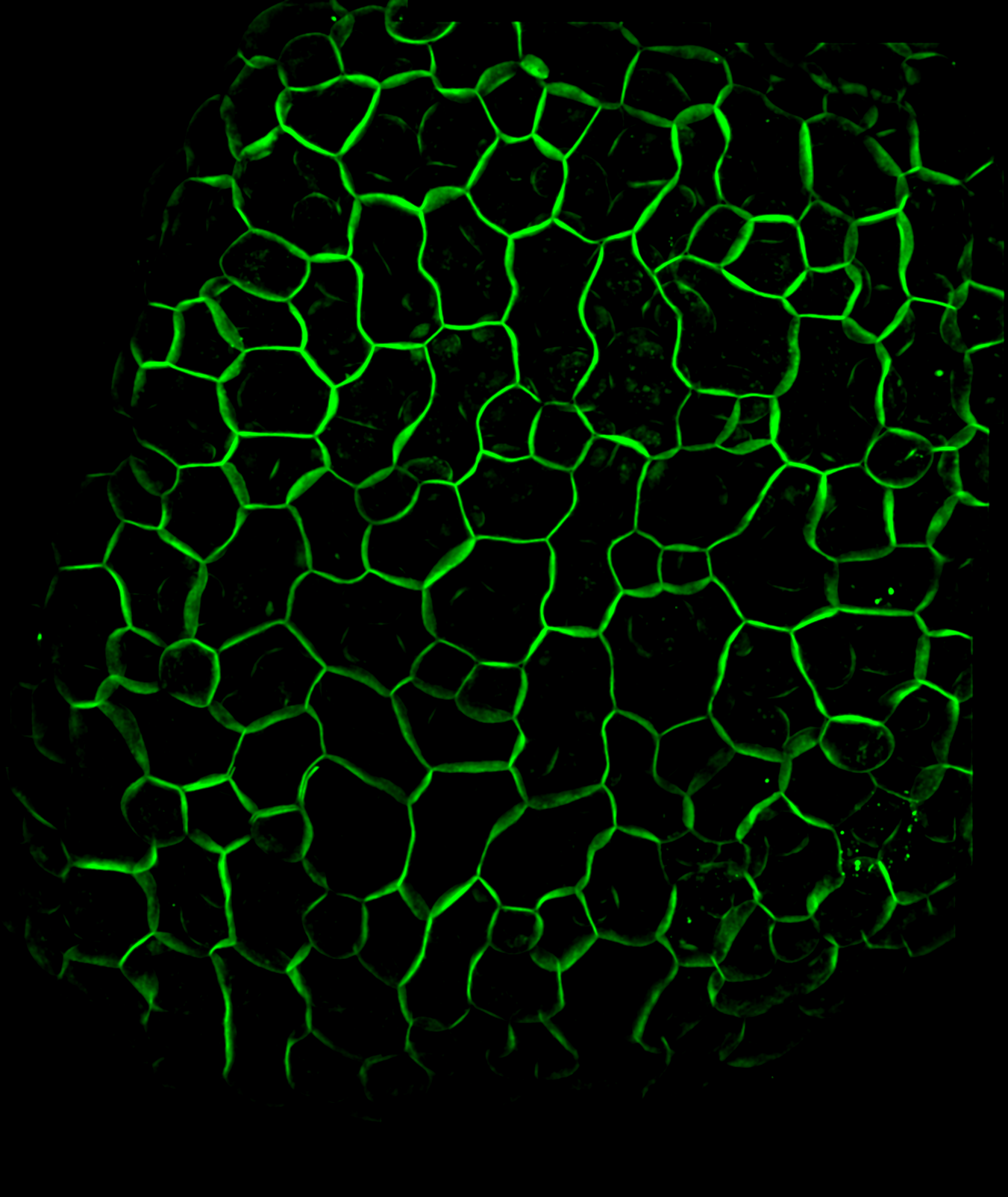

Supplement: Supplementary file 4 — Source Data [file 41467_2020_20730_MOESM4_ESM.zip › SourceData/Figure6_S13_S15_S19_LobeDetection/oryzalin_96h-GFP.tif]
