# Supplementary material for: A network-based framework for shape analysis enables accurate characterization of leaf epidermal cells
Source: Nat Commun. 2021 Jan 19;12:458. doi: 10.1038/s41467-020-20730-y (PMC7815848; doi:10.1038/s41467-020-20730-y)
Supplement: Supplementary file 2 — Peer Review File [file 41467_2020_20730_MOESM2_ESM.pdf]

Reviewer #1 (Remarks to the Author):

The present manuscript describes a new mathematical algorithm that serves to characterize 2D shapes. The algorithm is tested on simplified shapes, biologically relevant shapes (such as leaves or fish) and then focuses on the cell shapes in the plant leaf epidermis. The latter is a model system that has attracted quite a bit of attention as it is of interest in the context of plant evolution and plant development, as well as plant biomechanics.

There is a great need for a reliable algorithm since effective and efficient automated shape characterization is a requirement for high-throughput mutant analysis for example.

While the manuscript is certainly of interest and provides a solid comparison with other algorithms as well as examples for application, I have a number of concerns that the authors should consider:

MAJOR:
1. At many instances the writing style is cryptic for biologists. Given the type of journal and the targeted audience (many of whom are biologists), I recommend making an increased effort to write in more accessible manner.

2. If I understand correctly, Supplementary Figure 5 represents a sort of convergence test to illustrate the sensitivity of the allgorithm to the spatial resolution of node density (with respect to cell shape). This should be explained in much more detail since the choice of the number of nodes should high enough to not affect the outcome.

3. Do I understand correctly that the algorithm picks up differences in aspect ratios of cell shapes when the edges are weighted by length? As for figure 4, there was probably no weighting considered? Complexity simply referred to edge density? If so, what would be this figure look like if edges were weighted? Better explanations would be much appreciated.

4. It would truly be helpful if 'complexity' as defined in the present manuscript would be put into context with the definition of complexity as it was defined by previous publications on pavement cell shape (e.g. based on number of lobes per cell, or lobes per circumference (lobe frequency), lobe amplitude etc).

5. Figure 6: It is unclear to me whether the lobe numbers captured here include those created by three-cell junctions, and if so, whether that is the case for all algorithms used.

6. Figure 6: All data points should be represented as beeswarm, and the analysis should be paired since identical images were used to test all four algorithms. Importantly, the datapoints of the 'gold standard' obtained by 20 experts should be presented as beeswarm as well, not just a mean value. If for the gold standard, all 20 experts scored all 30 images, that beeswarm could show the average values of the 20 experts for each image.
Furthermore, it would also be interesting to see the raw data for the expert scores to establish how different individual 'experts' scored lobes. What is the threshold for a lobe to be a neck? Is there a minimum curvature? Minimum deviation from the overall convex shape or from a hull?

7. Figure 7: I suggest adding the data of clasp-1 here as well since it was used in Figure 6

MINOR
8. Line 22: Without having read the rest of the manuscript, the term 'domains' remains very cryptic here. Could it be replaced by 'objects' or 'shapes encountered in different domains'?

9. Line 45: 'principle' should be 'principal'

10. Line 77: The 'emergence of complex cell shapes' could pertain either to phylogeny or ontogeny. The cited paper provides an adequate example for the former, but it would be worthwhile to also refer to the latter. A fundamental mechanistic paper was Panteris and Galatis (2005 New Phytol) and over the past couple of years numerous important mechanistic papers have been published that illustrate the wider interest for this phenomenon.

11. Figure 1: I suggest indicating dE

12. Figure 1 and methods: The criteria and methodology of recognizing the contour itself are not described clearly anywhere. Please add this information somewhere, either under methods or in the legend of Fig 1.

13. Figure legend 1c. The purpose of this heat map remains cryptic until here. A bit more information already here would be helpful. Given that this manuscript is targeted to a journal with general audience it would really be helpful to provide the reader with a road map that hints at how such items are going to be used along the way. Secondly, it should be mentioned in the legend that black (or 0) can arise either through zero distance OR when the nodes cannot see each other.

Lines 114-15: Do you mean 'the same shape acquired under different resolution may have different number of nodes'? Or is the sentence meant as is, which seems trivial?

Fig. 2a: Labels on the axes are missing.

Figs. 2b,c: More explanation of these graphs and how they were produced would be welcome.

Line 186: How would groups be identified given that objects are on a spectrum and not in discrete categories?

Line 193: Replace 'understand' by 'investigate'?

Line 193-195: There are several recent papers that have investigated the biological/biochemical underpinnings of this process and/or modeled the mechanical mechanism. I strongly suggest citing them here.

Line 197: What is an 'undirected graph'? unweighted?

Line 226: Why do you state 'We hypothesize that lobes and necks correspond to local minima and maxima of the closeness centralities along the contour'? Isn't that the definition of closeness?

Lines 258-59: Shouldn't this sentence add '...compared to the other algorithms'?

Figure 7a: I suggest putting the color legend on the graph rather than in the legend text.

Reviewer #2 (Remarks to the Author):

NCOMMS-20-12304
A network-based framework for shape analysis enables accurate characterization and classification of leaf epidermal cells

The manuscript by Nowak et al presents a visibility graph-based method for comparative analysis of 2D shapes. It's usability is validated on different shapes, including simple geometrical shapes and fish

and plant leaf shapes. GraVis is applied to characterize shapes of leaf epidermis pavements cells and to quantify lobe numbers, which are a characteristic shape feature of pavement cells. Similar methods including measures of inner-distances between nodes at shape contours and visibility graphs have previously been used for shape characterization and in robotics. The novelty can be seen in applying these methods to quantification of pavement cell shapes and its adaptation to lobe quantification.

Comments:
Previous studies using similar approaches are not referenced. It should be clearly stated which algorithm and approaches have been applied from other published data and which of the algorithm have been developed within this study.

Comparison of visibility graphs of simple, synthetic shapes (Fig. 2) reveals that the quality of clustering depends on the number of nodes. Clustering works well when the same number of nodes is used for generation of visibility graphs. The quality of clustering significantly drops when different node numbers are used. Intriguingly, with different node numbers identical shapes that only differ in size (e.g., the two rectangles) are no longer classified as highly similar. Under conditions with different node numbers, size thus seems to be more relevant than shape. For pavement cell analysis, node number (or more precisely the distance between nodes) is calculated from the optimal number of nodes per µm and the image resolution (see below for additional comments). Image resolution and cell size thus may have a big impact on the robustness of the clustering approach. It would be helpful to provide similar comparative analyses not only for simple shapes but also for more complex shapes, e.g., for pavement cells, which are the focus of the study.

Visibility graphs were additionally tested for their ability to serve as global shape descriptors, using sand grains, fish outlines, and plant leaves as templates. While the separation worked well for fish shapes, the data are less clear for sand grains and leaves. To demonstrate the usability of the novel approach it would be helpful to include a comparison of the visibility graph approach to other existing approaches for shape description and clustering in PCA plots.

Heatmaps of pavement cell complexity are frequently used by showing circularity values. Is the heatmap shown in Fig.4 comparable to heat maps of circularity values (or other commonly used shape descriptors)? If so, what is the advantage of displaying visibility graph heatmaps? Which biologically relevant information can be extracted from these heatmaps that is not available from already existing approaches using heatmaps?

The authors state that comparative analyses for quantification of pavement cells, or more generally for shape descriptors, based on a gold standard are missing. In their study, they define a gold standard for lobe quantification but not for any other shape aspect. As gold standard, lobe numbers quantified in 30 cells by 20 experts are used and the mean is shown in the graph. Information on the variance of the individual measurements of the 20 experts, however, is not provided. It would be essential to provide information on the robustness of manual analysis, in particular as this serves as the gold standard and reference value. Manual quantification of lobe numbers by experts as gold standard has previously been used already, e.g. during validation of PaCeQuant in direct comparison to LobeFinder. Lastly, while the three other tools seem to overestimate lobe numbers, GraVis has a tendency to underestimate lobe numbers. I am wondering whether differences in lobe numbers may result from different numbers of nodes placed along the contour. How exactly was the optimal distance between nodes calculated? With identical distances between nodes larger cells would have more nodes. As shown in Fig. 2, however, differences in node number result in higher variance and less accuracy in clustering of simple shapes already.

For comparison of pavement cells in different Arabidopsis mutants, 20 cells are included for generation of the PCA plot. The PCA plot, however, only shows a single data point per genotype. Plotting the visibility graphs of all analyzed cells as individual data points would help to provide information on the comparability within the same genotype. Quantification of lobe numbers (and data shown in box plots)

on the other hand are results from 80 cells, and lobe numbers vary largely between individual cells of the analyzed genotypes. First, why did the authors exclude 60 cells from the PCA analysis? Second, wouldn’t a similar variation be expected in visibility graphs of cells within the same genotype? Also, no statistical analysis is provided for quantification of lobe numbers (box plots).

As stated in the manuscript, protrusions can be formed at 2-cell junctions or at tri-cellular junctions. According to the Materials and Methods section, crossing points of neighboring cell contours are used for detection of tri-cellular junctions. This, however, requires information on cell contours in all neighboring cells. To me it is not clear how this is guaranteed by the approach. Also, could the authors explain whether tri-cellular junctions were removed from the total lobe count and how they account for missing neighborhood information?

Lastly, the authors provide evidence that GraVis enables accurate classification of phylogenetic relationships from analysis of pavement cells. Again, only a single datapoint is shown for the individual phylogenetic clades (ferns, gymnosperms, angiosperms, eudicots and monocots) although several cells from 213 different species were included in the analysis. From the presented data it is not clear whether the GraVis-based approach for phylogenetic analyses outperforms the previously used approach based on select shape metrics (e.g., cell aspect ratio, solidity). A direct comparison would be helpful, in particular as the authors used a previously published dataset that was applied to a phylogenetic analysis.

The tool provides an additional image preprocessing and cell segmentation step. A description of the accuracy and precision of the segmentation pipeline, however, is not included in the manuscript.

Overall, the advantage of Gravis can be seen as offering a pipeline for comparative shape analysis based on a single shape descriptor. Except for lobe numbers, however, GraVis does not provide information on cell specific differences in pavement cell shape, such as lobe length, growth restriction at neck regions or mechanical stress acting on individual cells. I wonder how or if this tool can be applied to study changes in shape during development, as is suggested in the discussion.

Additional comments:
Fig.2, Fig. 5, Fig. S1, Fig. S2: poor image quality

GraVis can be downloaded from Github. Will the code be made available as well?

<u>Reviewer Comments:</u>

Reviewer #1 (Remarks to the Author):

The present manuscript describes a new mathematical algorithm that serves to characterize 2D shapes. The algorithm is tested on simplified shapes, biologically relevant shapes (such as leaves or fish) and then focuses on the cell shapes in the plant leaf epidermis. The latter is a model system that has attracted quite a bit of attention as it is of interest in the context of plant evolution and plant development, as well as plant biomechanics.

There is a great need for a reliable algorithm since effective and efficient automated shape characterization is a requirement for high-throughput mutant analysis for example.

While the manuscript is certainly of interest and provides a solid comparison with other algorithms as well as examples for application, I have a number of concerns that the authors should consider:

Response:
We thank the reviewer for recognizing the comparative analysis of our application. We carefully examined the stated concerns and made numerous updates to the algorithm and analyses, resulting in a fully revised version of the manuscript. We will make the data underlying our analysis available on our GitHub page.

MAJOR:
1. At many instances the writing style is cryptic for biologists. Given the type of journal and the targeted audience (many of whom are biologists), I recommend making an increased effort to write in more accessible manner.

Response:
We thank the reviewer for the recommendation to rewrite the manuscript in a more accessible manner. Therefore, we streamlined the manuscript and added more detailed examples and explanation for the methods. Each methodological step is now accompanied by a figure detailing and illustrating the respective part of the proposed algorithm.

2. If I understand correctly, Supplementary Figure 5 represents a sort of convergence test to illustrate the sensitivity of the algorithm to the spatial resolution of node density (with respect to cell shape). This should be explained in much more detail since the choice of the number of nodes should high enough to not affect the outcome.

Response:
We thank the reviewer for the suggestion and added a more detailed description of how the number of nodes influences the clustering. We implemented the Biological Homogeneity Index (BHI), as an objective measure to assess the quality of the clustering, given a priori information about groupings of shapes. The more shapes from a same group fall in a cluster, derived from the distances obtained from GraVis and the Fourier transform approach, the closer the value of BHI to 1 (Fig. 1b, Supplementary Fig. 5b-e).

Further, we proposed an additional approach for comparison of graphs which differ in their number of nodes. The approach reduced the larger of two compared graphs to have as many nodes as the smaller, by using the concept of network modularity

(Supplementary Fig. 4). The resulting reduced graph can be compared by using the rotational distance, already explained in the earlier version of the manuscript.

Interestingly, we find that the BHI value is the largest for the Laplacian approach, based on the comparison of graphs with the same number of nodes, followed by the rotational distance of the reduced graphs (which have different number of nodes, Supplementary Table 1). Therefore, the approaches based on the visibility graph, both on graphs with same and different number of nodes, outperform the approaches based on Fourier transform in this controlled synthetic case.

To show the sensitivity of the algorithm to the spatial resolution of the node density, we further used the set of synthetic shapes with the same number of nodes to illustrate the clustering quality based on different node densities (not included in the manuscript). Therefore, we selected the large shapes (3 triangles, 4 squares, 3 circles) with each the equal number of nodes (20). We used the node reduction method based on modularity clustering (Supplementary Fig. 4) to stepwise reduce the number of nodes of each graph, until all graphs were of order 12. For all these graph sets we calculated the distance matrices and used them for hierarchical complete-linkage clustering (Figure R1).

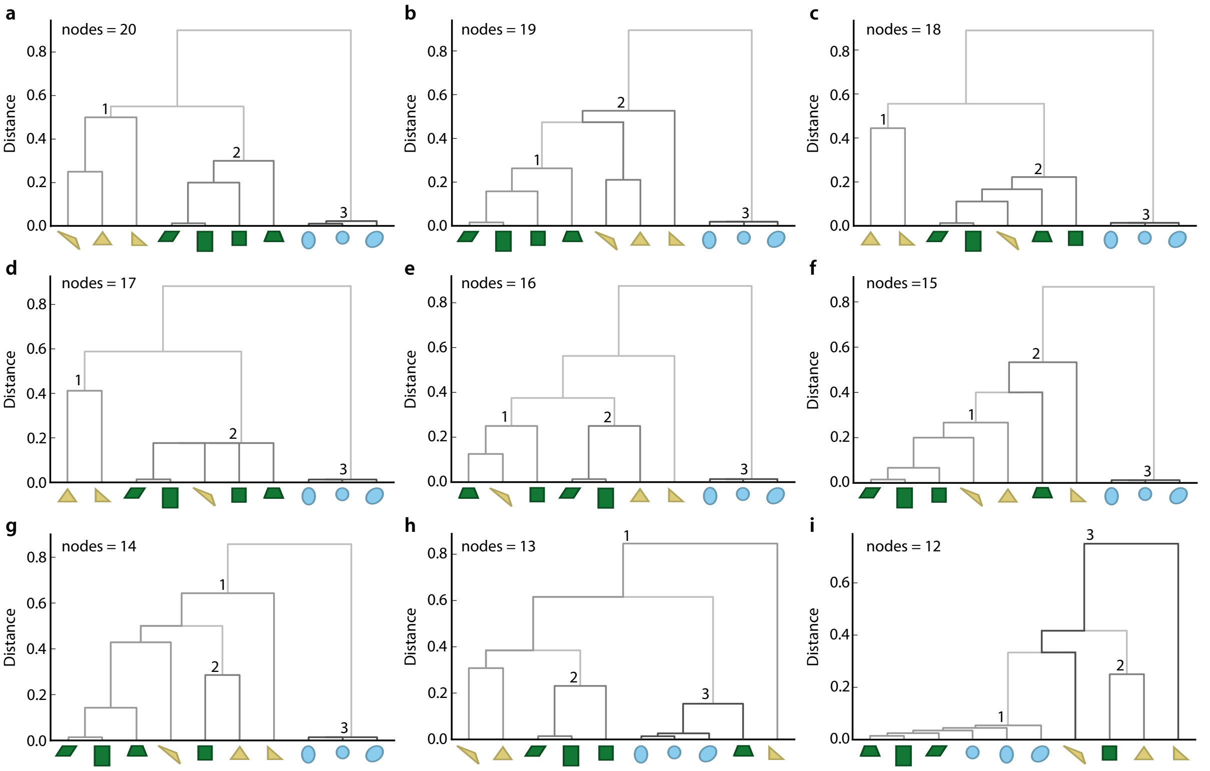

Figure R1: Comparison of visibility graphs with different node densities. (**a-i**) The visibility graphs of a set of synthetic shapes with the same number of nodes (20) were reduced stepwise by one node and used to calculate the distance matrix to use for hierarchical clustering. To measure the quality of the clustering, the BHI was calculated for the resulting clusters (see Figure R2).

We used the resulting clusters to compute the BHI and found that it decreases for visibility graphs with reduced node numbers (Figure R2). The visibility graphs with 20 and 19 nodes per graph had a perfect score of 1.0 for all clusters, thus showing that the corresponding node density of 10-14 pixel/nodes is optimal for the detection of distinguishing global shape features.

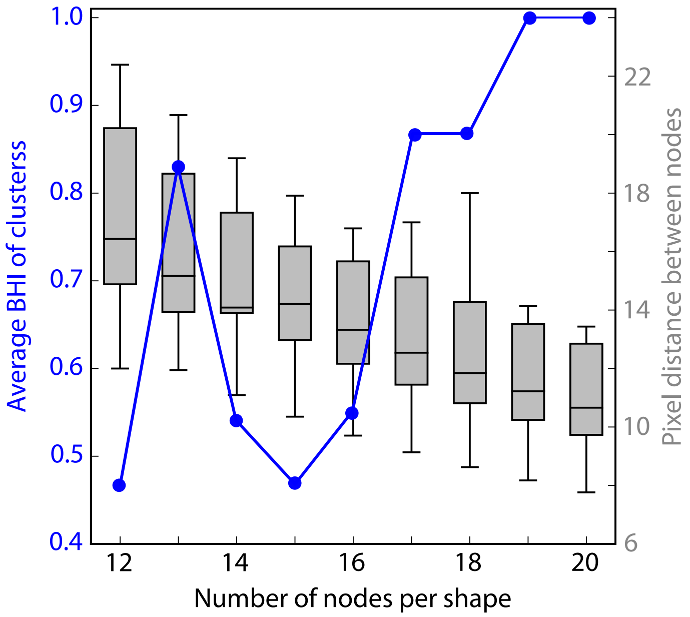

Figure R2: Sensitivity of the number of nodes on the performance of shape comparison. Visibility graphs with equal number of nodes were compared against each other for the synthetic shapes. The number of nodes were decreased in a stepwise fashion using the node-reducing method. The distance matrix was calculated for the resulting graphs and used for hierarchical clustering whose quality we quantified with the BHI (blue). The highest average BHI was calculated with graphs that have a distance between of 10-14 pixels between nodes (gray). Boxplots are shown with median (horizontal line), 25th and 75th percentiles (box edges) and 1.5-fold of the interquartile range (whiskers).

3. Do I understand correctly that the algorithm picks up differences in aspect ratios of cell shapes when the edges are weighted by length? As for figure 4, there was probably no weighting considered? Complexity simply referred to edge density? If so, what would be this figure look like if edges were weighted? Better explanations would be much appreciated.

Response:
For the clustering analysis and shape comparison based on GraVis we do not consider the weighted matrices. We have repeated the analysis to show that the weights of the edges actually worsen the results. This analysis points out that the structure of the network is determinant of the global shape properties (Supplementary Fig. 3). Nevertheless, we note that the weighting of edges begins to matter when we turn to local shape properties.

Finally, some of graph-theoretic properties are only defined for unweighted graphs, a typical example is the absolute density (number of edges relative to a clique). While relative density, corresponding to the average weighted degree, offers a possibility to consideration of edge weight, it cannot be used for comparison of two graphs due to the absence of a normalization factor. Clearly, the complexity of a shape is given by the number of convex / concave substructures, which can nicely be revealed by using simple measures defined on the unweighted graph, as we show in the updated version of the manuscript.

4. It would truly be helpful if 'complexity' as defined in the present manuscript would be put into context with the definition of complexity as it was defined by previous publications on pavement cell shape (e.g. based on number of lobes per cell, or lobes per circumference (lobe frequency), lobe amplitude etc).

Response:
We agree with the reviewer's comment and renamed complexity to relative completeness to avoid misunderstandings. We defined the relative completeness as the ratio between the graph edges and the maximum number of edges in the graph. In addition, we provide a comparison of the relative completeness to other pavement cell shape properties, like the circularity and the number of lobes per cell (see manuscript main text, Supplementary Fig. 8). Here, we show that, by using the relative completeness, we can identify subtle differences in cells with the same / similar circularity. For instance, we find that there is a class of cells which have small similarity but large relative completeness, which offers a refined description of cell shape.

5. Figure 6: It is unclear to me whether the lobe numbers captured here include those created by three-cell junctions, and if so, whether that is the case for all algorithms used.

Response:
We thank the reviewer for pointing out the confusion regarding the use of the number of lobes throughout the manuscript. We updated the manuscript text and the corresponding plots to make clear that we used the number of true lobes (excluding tri-cellular junctions) for the comparison of different pavement cell shape descriptors and the gold standard. Furthermore, we tested the statistical significance of differences between the number of detected true lobes by the shape descriptors and the gold standard (Figure 6). In the Supplementary Material, we added the comparison between the gold standard and the shape descriptors including both true lobes and tri-cellular junctions (Supplementary Fig. 13).

6. Figure 6: All data points should be represented as beeswarm, and the analysis should be paired since identical images were used to test all four algorithms. Importantly, the datapoints of the 'gold standard' obtained by 20 experts should be presented as beeswarm as well, not just a mean value. If for the gold standard, all 20 experts scored all 30 images, that beeswarm could show the average values of the 20 experts for each image. Furthermore, it would also be interesting to see the raw data for the expert scores to establish how different individual 'experts' scored lobes. What is the threshold for a lobe to be a neck? Is there a minimum curvature? Minimum deviation from the overall convex shape or from a hull?

Response:
We agree with the reviewer that the raw data of the gold standard should be included in the manuscript. Therefore, we added the bee swarms for each cell used for the gold standard and indicated the positions of tri-cellular junctions (Supplementary Fig. 11). The underlying raw data are made available in the provided Source Data (on the GitHub page). Furthermore, we added a supplementary figure which depicts the positions and consensus of detected lobes for nine representative cells of the gold standard (three cells each selected from the different conditions: Col-0, oryzalin-treated and *clasp-1*, see Supplementary Fig. 12). We did not use a score to evaluate the detected lobes, but rather indicated the percentage of expert consensus at a given position. In fact, Supplementary Figure 12 shows that small cell protrusions were less often detected while larger protrusions were recognized by the majority of experts.

7. Figure 7: I suggest adding the data of clasp-1 here as well since it was used in Figure 6

Response:
We added the data of *clasp-1* to Figure 7 and the subsequent analysis. We would like to highlight that the *clasp-1* data used for the comparison of the lines was different from the *clasp-1* data used for the comparison against the gold standard, as the corresponding underlying images were captured at different time points. More specifically, for the gold standard, images of *clasp-1* were captured at 96 hours after germination. The *clasp-1* images for the comparison of different lines was captured at 120 hours after post-dissection (see the updated Materials and Methods). Following this, the results for the number of detected lobes differ between the two comparisons.

MINOR
8. Line 22: Without having read the rest of the manuscript, the term 'domains' remains very cryptic here. Could it be replaced by 'objects' or 'shapes encountered in different domains'?

Response:
We changed the term 'domains' to 'shapes encountered in different domains'.

9. Line 45: 'principle' should be 'principal'.

Response:
We changed 'principle' to 'principal'.

10. Line 77: The 'emergence of complex cell shapes' could pertain either to phylogeny or ontogeny. The cited paper provides an adequate example for the former, but it would be worthwhile to also refer to the latter. A fundamental mechanistic paper was Panteris and Galatis (2005 New Phytol) and over the past couple of years numerous important mechanistic papers have been published that illustrate the wider interest for this phenomenon.

Response:
We thank the reviewer for the suggestion to cite additional papers and added adequate papers to the Introduction.

11. Figure 1: I suggest indicating dE

Response:
We indicated the Euclidean distance in the Figure heading.

12. Figure 1 and methods: The criteria and methodology of recognizing the contour itself are not described clearly anywhere. Please add this information somewhere, either under methods or in the legend of Fig 1.

Response:
We agree with the reviewer and added a more detailed description of how the contour is recognized in the Methods and Materials section.

13. Figure legend 1c. The purpose of this heat map remains cryptic until here. A bit more information already here would be helpful. Given that this manuscript is targeted to a journal

with general audience it would really be helpful to provide the reader with a road map that hints at how such items are going to be used along the way. Secondly, it should be mentioned in the legend that black (or 0) can arise either through zero distance OR when the nodes cannot see each other.

Response:
We added a more detailed description to the legend of Figure 1. Furthermore, we would like to clarify that a weight of zero indicated the absence of an edge, which ultimately indicates that two nodes are not visible to each other. Since we use simple graphs, no loops are allowed (with a loop denoting an edge connecting the node to itself).

Lines 114-15: Do you mean 'the same shape acquired under different resolution may have different number of nodes'? Or is the sentence meant as is, which seems trivial?

Response:
We thank the reviewer for picking this up. The sentence was changed to indicate that same shapes were meant.

Fig. 2a: Labels on the axes are missing.

Response:
We added the missing labels to the Figure axes.

Figs. 2b,c: More explanation of these graphs and how they were produced would be welcome.

Response:
We added a more detailed description of the approaches used in the Materials and Methods sections, as well as in the Supplementary Material.

Line 186: How would groups be identified given that objects are on a spectrum and not in discrete categories?

Response:
To represent objects on a spectrum, we added a set of 12 pavement cells selected from three genotypes and tested the different shape comparison methods (Supplementary Fig. 6, Supplementary Table 2).

Line 193: Replace 'understand' by 'investigate'?

Response:
We replaced ‘understand’ by ‘investigate’.

Line 193-195: There are several recent papers that have investigated the biological/biochemical underpinnings of this process and/or modeled the mechanical mechanism. I strongly suggest citing them here.

Response:
We thank the reviewer for the suggestion and added recent papers to the references.

Line 197: What is an 'undirected graph'? unweighted?

Response:
The visibility graphs we create are undirected graphs, i.e. their edges are bidirectional. Furthermore, the edges of these undirected graphs can be either weighted, in our case by the Euclidean distance between two nodes, or unweighted. We added a short description to define undirected graphs in the manuscript text.

Line 226: Why do you state 'We hypothesize that lobes and necks correspond to local minima and maxima of the closeness centralities along the contour'? Isn't that the definition of closeness?

Response:
The closeness centrality of a node in a graph provides a measure of how close the node is to the rest of the nodes in the graph. We use this idea to identify the nodes which are located in neck or lobe region by identifying the local maxima or minima of closeness centralities along the cell contour, respectively. The concept we use here is the fact that nodes in neck regions are very central and are more visible to other nodes, while the visibility of nodes in lobe regions is more restricted to nodes in the immediate proximity. Following this, the nodes in a graph with the highest closeness centralities are the nodes, which have the shortest distance to all other nodes, i.e. they are likely to be in neck regions. In contrast, nodes with a low closeness centrality are more likely to be in a lobe region.

Lines 258-59: Shouldn't this sentence add '...compared to the other algorithms'?

Response:
We corrected the sentence.

Figure 7a: I suggest putting the color legend on the graph rather than in the legend text.

Response:
We added a color legend to the plot for easier discrimination of the depicted data.

Reviewer #2 (Remarks to the Author):

A network-based framework for shape analysis enables accurate characterization and classification of leaf epidermal cells

The manuscript by Nowak et al presents a visibility graph-based method for comparative analysis of 2D shapes. Its usability is validated on different shapes, including simple geometrical shapes and fish and plant leaf shapes. GraVis is applied to characterize shapes of leaf epidermis pavements cells and to quantify lobe numbers, which are a characteristic shape feature of pavement cells. Similar methods including measures of inner-distances between nodes at shape contours and visibility graphs have previously been used for shape characterization and in robotics. The novelty can be seen in applying these methods to quantification of pavement cell shapes and its adaptation to lobe quantification.

Response:
We thank the reviewer for recognizing the novelty of our approach. We attentively examined the comments and added changes to the manuscript accordingly.

Comments:
Previous studies using similar approaches are not referenced. It should be clearly stated which algorithm and approaches have been applied from other published data and which of the algorithm have been developed within this study.

Response:
We thank the reviewer for pointing out the missing references. To account for this, we added a paragraph in the Results section, which explains the various applications of visibility graphs in numerous areas and how these differ from our proposed approach. We hope the reviewer will recognize that we were exhaustive in reviewing the literature about all concepts of visibility graphs proposed to date along with their applications, as documented by the chronology and time span of the cited references.

Comparison of visibility graphs of simple, synthetic shapes (Fig. 2) reveals that the quality of clustering depends on the number of nodes. Clustering works well when the same number of nodes is used for generation of visibility graphs. The quality of clustering significantly drops when different node numbers are used. Intriguingly, with different node numbers identical shapes that only differ in size (e.g., the two rectangles) are no longer classified as highly similar. Under conditions with different node numbers, size thus seems to be more relevant than shape. For pavement cell analysis, node number (or more precisely the distance between nodes) is calculated from the optimal number of nodes per µm and the image resolution (see below for additional comments). Image resolution and cell size thus may have a big impact on the robustness of the clustering approach. It would be helpful to provide similar comparative analyses not only for simple shapes but also for more complex shapes, e.g., for pavement cells, which are the focus of the study.

Response:
We agree with the reviewer and selected a set of 12 pavement cells from three different genotypes to provide a similar comparative analysis as with the set of synthetic shapes (Supplementary Fig. 6). Due to the different number of nodes of extracted visibility graphs, the rotational distance and Fourier transform analysis could not be applied to compare the shapes. Therefore, we implemented a node reducing method, which uses modularity clustering to get graphs with equal number of nodes (Supplementary Fig. 4). The resulting reduced graphs can be then compared by using the rotational distance or Fourier transform, as already explained in the earlier version of the manuscript.

We implemented the Biological Homogeneity Index (BHI), as an objective measure to assess the quality of the clustering, given a priori information about groupings of shapes. The more shapes from a same group fall in a cluster, derived from the distances obtained from GraVis and the Fourier transform approach, the closer the value of BHI to 1 (Supplementary Table 1 for synthetic shapes, Supplementary Table 2 for selected pavement cells).

We found that the Laplacian approach used by GraVis resulted in the most homogenous clusters for the pavement cells, closely followed by the Fourier transform approach using the correlation distance (Supplementary Fig. 6, Supplementary Table 2).

Visibility graphs were additionally tested for their ability to serve as global shape descriptors, using sand grains, fish outlines, and plant leaves as templates. While the separation worked well for fish shapes, the data are less clear for sand grains and leaves. To demonstrate the usability of the novel approach it would be helpful to include a comparison of the visibility graph approach to other existing approaches for shape description and clustering in PCA plots.

Response:
We thank the reviewer for the suggestion. The separation of plant leaves is driven by the serration pattern, which cannot be seen in the small panel. As suggested, we used the Fourier transform to cluster the visibility graphs of sand grains, fish and leaves (Supplementary Fig. 7). Our analysis demonstrates that Fourier transform separates the shapes less well than the visibility graphs.

Heatmaps of pavement cell complexity are frequently used by showing circularity values. Is the heatmap shown in Fig.4 comparable to heat maps of circularity values (or other commonly used shape descriptors)? If so, what is the advantage of displaying visibility graph heatmaps? Which biologically relevant information can be extracted from these heatmaps that is not available from already existing approaches using heatmaps?

Response:
As also pointed out by reviewer #1, we analysed the correlation between the relative completeness (the new name for complexity, prompted by a comment of reviewer #1) and the circularity and number of lobes (Supplementary Fig. 8). We found that the relative circularity offers a refinement in the description of cells, since we identify cells which are of same circularity but they clearly differ with respect to their relative completeness. Another main advantage of using the relative completeness is the potential for an automated pipeline to accurately identify and screen for stomata, which is part of our future work based on the framework proposed here.

The authors state that comparative analyses for quantification of pavement cells, or more generally for shape descriptors, based on a gold standard are missing. In their study, they define a gold standard for lobe quantification but not for any other shape aspect. As gold standard, lobe numbers quantified in 30 cells by 20 experts are used and the mean is shown in the graph. Information on the variance of the individual measurements of the 20 experts, however, is not provided.

Response:
We added a more detailed depiction of the gold standard measurements in the Supplementary Material (see reviewer #1).

It would be essential to provide information on the robustness of manual analysis, in particular as this serves as the gold standard and reference value. Manual quantification of lobe numbers by experts as gold standard has previously been used already, e.g. during validation of PaCeQuant in direct comparison to LobeFinder.

Response:
We corrected our statement in the manuscript.

Lastly, while the three other tools seem to overestimate lobe numbers, GraVis has a tendency to underestimate lobe numbers. I am wondering whether differences in lobe numbers may result from different numbers of nodes placed along the contour. How exactly was the optimal distance between nodes calculated? With identical distances between nodes larger cells would have more nodes. As shown in Fig. 2, however, differences in node number result in higher variance and less accuracy in clustering of simple shapes already.

Response:
We thank the reviewer for noticing the tendencies in under- and overestimation of lobes by the different tools. We repeated the analysis to make sure that we only analyse true lobes without tri-cellular junctions and did a statistical testing to see if there are differences in the means of the tools compared to the gold standard mean. Although GraVis seems to underestimate the number of lobes, we could detect no significant difference to the mean of the gold standard. In contrast, the other tools were overestimating the number of lobes and often showed a significant difference. We furthermore added the analysis including tri-cellular junctions and found a similar result (Supplementary Fig. 13).

To calculate the optimal distance, we selected specific pixel distances to create the visibility graphs of the gold standard pavement cells. We started with a pixel distance of one, which places a node at each second pixel of the contour and increased the pixel distance stepwise by one to a maximum of 39 pixels between nodes. As described in the manuscript, we calculated the RMSE between detected lobes of the visibility graphs with a certain pixel distance and the manually detected lobes of the gold standard (Supplementary Fig. 14). We found that very small pixel distances (2-3 px) are too sensitive to capture meaningful local shape features (due to subtle oscillations in their values due to small perturbations of the contour), as shown by a very high RMSE (2-4 times higher than for a pixel distance of 4 px, Supplementary Fig. 13a). The lowest RMSE was displayed between pixel distances of six and 12 pixels. Based on the pixel distances that resulted in the lowest RMSE and the image resolutions, we calculated the optimal distance for the placement of nodes.

In addition, with the newly proposed approach for comparison of graphs with different number of nodes, we can actually reduce the larger to have the same number of nodes as the smaller graph (using the modularity approach) and then apply the rotational distance for their comparison. Our analysis of synthetic shapes actually shows that this approach outperforms the Fourier transformation approach and fairs as good as the Laplacian approach proposed in the first version of the manuscript.

For comparison of pavement cells in different Arabidopsis mutants, 20 cells are included for generation of the PCA plot. The PCA plot, however, only shows a single data point per genotype. Plotting the visibility graphs of all analyzed cells as individual data points would help to provide information on the comparability within the same genotype. Quantification of lobe numbers (and data shown in box plots) on the other hand are results from 80 cells, and lobe numbers vary largely between individual cells of the analyzed genotypes. First, why did the authors exclude 60 cells from the PCA analysis? Second, wouldn't a similar variation be expected in visibility graphs of cells within the same genotype? Also, no statistical analysis is provided for quantification of lobe numbers (box plots).

Response:
We apologize for the non-visible data points in the PCA plots. We made sure the missing data points are shown now and included the data points for all 80 cells per genotype. In addition, we added a statistical analysis using ANOVA and a pairwise t-test for the number of detected lobes. Using the information from the pairwise t-tests we created a graph, which depicts clusters of genotypes with similar pavement cell shapes (p-value > 0.01), and confirms the results of the PCA (Supplementary Fig. 15). For example, we found that the two genotypes expressing nearly no lobes (*CA-ROP2*, *RIC1-OX)* were clustered together. Furthermore, the genotypes with highly lobed pavement cells are clustered together (*spr2-2,* Ws, *ric1-1*), although Col-0, which expresses the most complex pavement cells, is different to all other genotypes.

As stated in the manuscript, protrusions can be formed at 2-cell junctions or at tri-cellular junctions. According to the Materials and Methods section, crossing points of neighboring cell contours are used for detection of tri-cellular junctions. This, however, requires information on cell contours in all neighboring cells. To me it is not clear how this is guaranteed by the approach. Also, could the authors explain whether tri-cellular junctions were removed from the total lobe count and how they account for missing neighborhood information?

Response:
The extraction of tri-cellular junctions is based on information we extract directly from the underlying image data. The input for the analysis of pavement cells are images of epidermal tissues, thus depicting a multiple of neighbouring pavement cells. During the pre-processing of the input image, truncated cells are removed and only whole cells kept. We use the skeletonized representation of the cell contours to identify tri-cellular junctions by detecting three-way crossways. In addition, we use cell contour information of previously removed truncated cells to detect tri-cellular junctions of pavement cells at the image border. In conclusion, the detection of tri-cellular junctions is quite accurate for cells which are in the interior of the image, while we might miss tri-cellular junctions of cells at the border of the image. In the output table that is created by GraVis, we indicate the number of all detected lobes, as well as the number of detected tri-cellular junctions and how many of the detected lobes correspond with a tri-cellular junction. The results for cells at the image border should therefore be treated with care.

In the updated version of the manuscript we indicated that the comparisons are done using the number of lobes without tri-cellular junctions, but we additionally did comparisons including tri-cellular junctions in the Supplementary Material (Supplementary Fig. 13).

Lastly, the authors provide evidence that GraVis enables accurate classification of phylogenetic relationships from analysis of pavement cells. Again, only a single datapoint is shown for the individual phylogenetic clades (ferns, gymnosperms, angiosperms, eudicots and monocots) although several cells from 213 different species were included in the analysis. From the presented data it is not clear whether the GraVis-based approach for phylogenetic analyses outperforms the previously used approach based on select shape metrics (e.g., cell aspect ratio, solidity). A direct comparison would be helpful, in particular as the authors used a previously published dataset that was applied to a phylogenetic analysis.

Response:
We apologize for the erroneous depiction of the data points in the presented data. We revised the plot representation and ensured that all data points are visible. Furthermore, we used ImageJ to extract the aspect ratio and the solidity of the 6359 selected cells from the provided dataset. We were able to reproduce the results for the solidity and aspect ratio distribution across different plant clades as shown in Vöfely *et al.*, 2018. In addition, we plotted the distribution of parameters that were extracted using GraVis, such as the number of lobes and the relative completeness (previously complexity, see reviewer #1). Together, the plots serve as a direct comparison between different cell shape metrics (Supplementary Fig. 17). Using a one-way ANOVA coupled with post-hoc tests, we found that all of the clade pairs could be distinguished using the solidity, while 10% of the clade pairs could not be distinguished by the aspect ratio, number of lobes and relative completeness (Supplementary Table 5).

The tool provides an additional image preprocessing and cell segmentation step. A description of the accuracy and precision of the segmentation pipeline, however, is not included in the manuscript.

Response:
We thank the reviewer for suggesting the addition of the accuracy and precision description of our segmentation pipeline (Supplemenatary Fig. 18, Supplementary Table 6). We added the description to the Materials and Methods section.

Overall, the advantage of Gravis can be seen as offering a pipeline for comparative shape analysis based on a single shape descriptor. Except for lobe numbers, however, GraVis does not provide information on cell specific differences in pavement cell shape, such as lobe length, growth restriction at neck regions or mechanical stress acting on individual cells. I wonder how or if this tool can be applied to study changes in shape during development, as is suggested in the discussion.

Response:
We are thankful for the encouragement to provide additional parameters to the output of our framework. Therefore, we added parameters like the cell area, the cell perimeter, the circularity, the lobe length and the neck width to the output. Furthermore, we added a visual output for GraVis that depicts the positions of detected necks, lobes and tri-cellular junctions for each cell (Supplementary Fig. 10).

We would like to point out that no other tool provides mechanical stresses, since they come from the Finite Element Method. To study shape changes during development, our framework can nicely be used to show how properties change over time. This will require the tracking of cells over time, which is not part of the study presented here.

Additional comments:
Fig.2, Fig. 5, Fig. S1, Fig. S2: poor image quality

Response:
We improved the quality of all our images.

GraVis can be downloaded from Github. Will the code be made available as well?

Response:
We thank the reviewer for the interest in the source code of our framework. We are happy to share the code of the application on GitHub once the approach is published. Until then, we will make the code available upon request.

Reviewer #1 (Remarks to the Author):

The authors have responded to my previous concerns in satisfactory manner.

The new version of the manuscript raises a new (but minor) question though with regards to Supplementary Figure 12: I am puzzled that there would be any less than 100% detection of tri-cellular junctions by the experts. Tri-cellular junctions are clearly and unambiguously discernable on a tissue micrograph because of the third cell wall segment radiating from that point. Why would there be a difference in judgement between experts?

Reviewer #2 (Remarks to the Author):

I would like to thank the authors for submitting their revised manuscript, in which many of my previous comments have been addressed. The addition of more detailed descriptions of the methods and approaches, however, raised a number of additional questions that are critical for evaluation of the overall quality of GraVis.
The accuracy of shape comparison with GraVis highly depends on the number of nodes placed along the contour (Fig. R1, R2, Fig 2 b-d). This raises a number of questions related to the usability of GraVis for comparison of biological input data sets:
Given that nodes are placed equidistantly along a given cell contour as stated in the Methods section it will directly affect the shape comparison in multiple ways. The number of nodes will differ between cells of different sizes within a genotype (stomata guard cells, SLGCs, meristemoids, PCs of different size) or between different species and between cells of identical area but different degree of lobing (e.g. in different genotypes). Wouldn't it then be more accurate to only compare cells with a similar contour length? In this case, GraVis would not be best suited for high throughput comparative analyses as only a small set of cells could be included in the analysis. Could the difference in cell size explain why GraVis doesn't work well for separation of cells from different species (Fig. S16 and S17)?
The detection of lobes largely depends on the number of nodes placed along the contour. The optimal distance between nodes thus is very critical for accuracy of GraVis-based lobe detection as also mentioned in the Methods section. In their proof-of-concept study the authors explicitly tuned this parameter by selecting the optimal pixel distance identified by creating "visibility graphs using node distances ranging from 2 to 39 pixels for the test set of 30 pavement cells". The authors then selected the node distance for their comparative analysis which gave the highest overlap with their gold standard. Consequently, the comparison of the RMSE is misleading since only for GraVis the detection/analysis parameters have been optimized to result in the lowest RMSE. Therefore, obviously, GraVis will have the best RMSE (Fig. 6d, S13d). For all other tools included in the comparative analysis no tuning/detection optimization was performed, which introduces bias in the presented data. Such tuning would also be possible at least for LOCO-EFA and PaCeQuant. How would the other tools perform with manually adjusted start settings/tuning of their performance to match the defined gold standard? Would GraVis still outperform existing approaches? With input images acquired with different imaging settings: Would GraVis still outperform the other tools without additional tuning/node distance optimization?
The data presentation of the gold standard appears a bit misleading to me. Why is information on the variance in lobe detection by experts shown for lobes at two cell junctions only (Fig S11)? Apparently, even among experts a lot of variation in lobe recognition occurred (Fig. S11 and S12). Lobe numbers, e.g. vary from 1 to 8 or 2 to 10 within single cells of clasp mutants. How reliable is it to compare GraVis and the other tools only to the mean of the gold standard? It would be helpful to include confidence intervals for the gold standard in Fig 6 or even better include the values of the gold standard as additional data point in the box plots, which could serve as the reference for statistical analysis.
It is difficult to see differences between light and dark gray in Fig S12.
The mean of the gold standard in Fig 6b and c and Fig S13 b and c is not consistent with the data

shown in Fig S11. Are claps and oryzalin mixed up? Fig. 6b/S13b seems to show the results from clasp mutants and Fig. 6c/S13c the oryzalin-treated cells. Why are the medians (shown in the boxes) compared to the mean of the gold standard (and not the median?) Given the large variance in lobe numbers detected by experts in clasp mutants: would there still be a significant difference between the median of the manual lobe quantification and the quantification with LobeFinder or PaCeQuant? Fig S13e: What is the variance in recovery of tri-cellular junctions by the 20 experts? It is surprising that manual recovery was lower than the automatic approaches.
Usability of GraVis for comparative analysis of wild type and mutants (Fig. 7): Fig.7b: By including 80 instead of 30 cells the outcome of the comparative analysis changed dramatically when compared to the data presented in the first draft of the manuscript. Except for the light blue cells distinct clusters are barely visible. It would help to use the different shapes (triangles, squared, pentagons) in the legend, too. Statistical analysis of (differences in) lobe number is still missing in Fig. 7c. Lobe numbers seems to be overestimated in CA-ROP2 and RIC1-OX, at least in comparison to the representative images shown in Fig. 7a. Without statistical information it is difficult to interpret the presented data. I, however, doubt that the number of lobes quantified per cell with GraVis will differ between rop4-1, lue1 and RIC1-OX, which I would expect from the shown images (Fig. 7a).
For statistical analysis, the parametric ANOVA was used, which requires normal distribution of data. The data presented, however, fail normal distribution assumption as indicated by a tendency of medians to lie outside the center of the boxes, and lobe numbers in different genotypes show different levels of variance. Therefore, the statistical analysis should be revisited.

REVIEWER COMMENTS

Reviewer #1 (Remarks to the Author):

The authors have responded to my previous concerns in satisfactory manner.

The new version of the manuscript raises a new (but minor) question though with regards to Supplementary Figure 12: I am puzzled that there would be any less than 100% detection of tri-cellular junctions by the experts. Tri-cellular junctions are clearly and unambiguously discernable on a tissue micrograph because of the third cell wall segment radiating from that point. Why would there be a difference in judgement between experts?

Response:
We thank the reviewer for acknowledging the changes in the revision. Regarding the minor point concerning Supplementary Fig. 12 (now Supplementary Fig. 14), we would like to point out that we agree with the reviewer: Tri-cellular junctions are easily distinguishable when taking the whole tissue of pavement cells into account. Here, however, we provided the experts with the contours of individual pavement cells, outside of the tissue context, and thus provided no information about cell wall segments with neighbouring cells. We further agree that this information would be helpful to recover 100% of the tri-cellular junctions and will take this into account when preparing future gold standards.

Reviewer #2 (Remarks to the Author):

I would like to thank the authors for submitting their revised manuscript, in which many of my previous comments have been addressed. The addition of more detailed descriptions of the methods and approaches, however, raised a number of additional questions that are critical for evaluation of the overall quality of GraVis.

Response:
We thank the reviewer for carefully examining the provided Supplementary Material and the additional questions regarding the quality of GraVis, which we address in the following point-by-point responses.

The accuracy of shape comparison with GraVis highly depends on the number of nodes placed along the contour (Fig. R1, R2, Fig 2 b-d).

Response:

We would like to refer to our response in the earlier reviewer response, where we compared visibility graphs with different node densities (Supplementary Fig. 6, 7). Here, we showed that visibility graphs with 20 and 19 nodes per graph had a perfect BHI score of 1.0 for all clusters. In addition, we observe that the BHI score is slightly below 0.9 for 17 and 18 nodes, demonstrating the robustness of our approach for small differences in node numbers. Changes in BHI for number of nodes that differ by one is not larger than 36%.

This raises a number of questions related to the usability of GraVis for comparison of biological input data sets:

Given that nodes are placed equidistantly along a given cell contour as stated in the Methods section it will directly affect the shape comparison in multiple ways.

The number of nodes will differ between cells of different sizes within a genotype (stomata guard cells, SLGCs, meristemoids, PCs of different size) or between different species and between cells of identical area but different degree of lobing (e.g. in different genotypes).

Response:

We do not see the point in the reviewer's argument that equidistant placing of nodes is problematic for the shape comparison. Using a set of synthetic shapes and four different approaches for measuring the distance between shapes, we showed that the shape comparison of synthetic shapes of different sizes (small/large) with either equal or different number of nodes works very well, precisely as shown in Figure 2b-d. Here, the shapes were clustered independent of the shape size. Furthermore, using the gold standard we calculated the optimal distance between nodes to be place along a shape contour to enable the optimal detection of lobes based on the underlying visibility graph. This optimal distance depends on the length of the cell contour and the image resolution, thus allowing the analysis of cells of different sizes/experiments/species.

Wouldn't it then be more accurate to only compare cells with a similar contour length? In this case, GraVis would not be best suited for high throughput comparative analyses as only a small set of cells could be included in the analysis.

Response:

We do not agree with the reviewer here, as we show that GraVis is indeed capable of comparing shapes with different contour lengths. In Supplementary Table 1 we show the quality of synthetic shape clusters derived from different comparison methods. Here, it can be seen that the Laplacian method perfectly clusters the synthetic set with equal number of nodes into three distinct clusters (mean BHI=1.0). We used sets of smaller and larger shapes; thus, the shapes have different contour lengths. Nevertheless, the clustering based on the Laplacian method (used in GraVis) on shapes with equal number of nodes outperforms all other methods. Furthermore, the Laplacian method also performs very well for the synthetic set with different number of nodes, where the shapes also have different contour lengths.

Could the difference in cell size explain why GraVis doesn't work well for separation of cells from different species (Fig. S16 and S17)?

Response:

We do not agree with the explanation that the difference in cell size is problematic for the quality of separation of cells. In fact, the separation of cells from different species is a difficult classification problem, since cells in the same clade are not, *per se*, of same shape (Vőfély *et al.,* 2018). Therefore, we cannot see a distinction of different clades on the 2D PCA plot. Furthermore, no distinction can be seen in the 3D PCA (Supplementary Figure 22b), suggesting nonlinear dependencies which do not allow for a simple shape separation. This is why we rely on machine learning to make the distinction of cell shapes based on multiple features.

In addition, we would like to add that nodes in the visibility graphs are placed according to the size of a cell. We gauged the optimal number of nodes per contour length based on the gold standard and used the resulting expression (dependent on the image resolution) to calculate the node distance for all further analysis, including the comparison of different genotypic pavement cell lines and the comparison of cells from different plant clades.

The detection of lobes largely depends on the number of nodes placed along the contour. The optimal distance between nodes thus is very critical for accuracy of GraVis-based lobe detection as also mentioned in the Methods section. In their proof-of-concept study the authors explicitly tuned this parameter by selecting the optimal pixel distance identified by creating “visibility graphs using node distances ranging from 2 to 39 pixels for the test set of 30 pavement cells”. The authors then selected the node distance for their comparative analysis which gave the highest overlap with their gold standard.

Response:

We would like to stress that we removed LOCO-EFA from the comparison of lobe detection tools for the number of lobes without tri-cellular junctions in the main text. We did so since LOCO-EFA provides no information about the number of true lobes and junctions, and we did not want to bias the comparison. As a result, we moved the comparison of LOCO-EFA’s performance to the Supplementary Material (Supplementary Fig. 15), where the lobe detection tools are compared using both the number of true lobes and tri-cellular junctions. We updated the text in the manuscript accordingly. The rest of the comment is fully addressed in the paragraph below.

Consequently, the comparison of the RMSE is misleading since only for GraVis the detection/analysis parameters have been optimized to result in the lowest RMSE. Therefore, obviously, GraVis will have the best RMSE (Fig. 6d, S13d). For all other tools included in the comparative analysis no tuning/detection optimization was performed, which introduces bias in the presented data.

Such tuning would also be possible at least for LOCO-EFA and PaCeQuant. How would the other tools perform with manually adjusted start settings/tuning of their performance to match the defined gold standard? Would GraVis still outperform existing approaches?

Response:

We thank the reviewer for the suggestion to tune the tool parameters to compare the tools performances. We would like to note that the parameter for GraVis (node distance) was only optimized once on the set of the gold standard. The resulting formula for the calculation of the optimal node distance is then used in all further pavement cell analysis. This step is fully automated and only needs the image resolution as user input. In contrast, we found that the user can change parameters in PaCeQuant to change the quantity of

detected lobes. While we could find no parameters to tune in LobeFinder, we found that in LOCO-EFA different thresholds between consecutive modes could be used as parameter for detecting lobes. To tune the parameters, we implemented a method which splits the gold standard into a training and test set to find the optimal parameter setting for each tool (see Supplementary Figure 18). The results show that, indeed, the performance of PaCeQuant and LOCO-EFA improved (Supplementary Figure 19). Interestingly, the difference between GraVis and PaCeQuant was not significant for both scenarios when excluding and including tri-cellular junctions (Supplementary Figure 19d, h). LobeFinder performed slightly worse than GraVis for lobes excluding tri-cellular junctions, while there was no difference for lobes including junctions.
In addition, we would like to point out that there is a difference in variance between contending tools (Bartlett's test: p-values $< 10^{-8}$ (default, without junctions), p-value $< 10^{-9}$ (default, with junctions), p-value $< 10^{-5}$ (tuned, without junctions), p-value < 0.005 (tuned, with junctions)) that is higher for the contenders in comparison to GraVis. The higher variance of the contending tools, PaCeQuant, LobeFinder, and LOCO-EFA, particularly for the comparison of true lobes without tri-cellular junctions, shows that these tools are not suitable for applications with different genotypes, cell types or cell sizes (Supplementary Table 5). This has a significant impact on any conclusion drawn from application of these tools with and without fine tuning of parameters --- which cannot be done repeatedly on a case-by-case basis, risking inflation of the bias of the findings.
Although the results show that tuning of the tools parameters change the performances of PaCeQuant and LOCO-EFA, they also demonstrate that the user has to figure out which parameters will give the best results. In the case of PaCeQuant, it is not obvious which parameters and combinations thereof will achieve this goal, thus leading the user to try random parameter settings. In contrast, these efforts are not necessary in GraVis, as all steps are fully automated.

With input images acquired with different imaging settings: Would GraVis still outperform the other tools without additional tuning/node distance optimization?

Response:
We appreciate the suggestion of the reviewer and would happily analyse the performance of the different lobe detection tools using another set of images. This would require a new gold standard, i.e. manually detected lobes by experts, which we currently do not have.

The data presentation of the gold standard appears a bit misleading to me. Why is information on the variance in lobe detection by experts shown for lobes at two cell junctions only (Fig S11)?

Response:
We do not fully understand the question of the reviewer. In Supplementary Figure 13, we depict the number of manually detected lobes without taking into account the tri-cellular junctions. We highlighted the position of tri-cellular junctions by green circles, which does not indicate the positions of the detected true lobes. The experts had no prior knowledge of tri-cellular junctions since we only provided the cell contour without cell wall segments of neighbouring cells. All lobes that were detected by the experts are in positions not indicated in the Figure. For exact positions of detected lobes and tri-cellular junctions we would like to refer to Supplementary Figure 14.

Apparently, even among experts a lot of variation in lobe recognition occurred (Fig. S11 and S12). Lobe numbers, e.g. vary from 1 to 8 or 2 to 10 within single cells of clasp mutants.

Response:
The variation of lobes detected by the experts is not uncommon and occurs across different applications (Wu *et al.*, 2016; Moeller *et al.*, 2017). During the analysis of the manually detected lobes, we found that some experts tended to be more sensitive in their lobe detection, i.e. very small bumps in the contour were detected as lobes. In contrast, other experts were more lenient and selected very few positions to be lobes. As a result, the variation among the experts can, indeed, be as described by the reviewer.

How reliable is it to compare GraVis and the other tools only to the mean of the gold standard?

Response:
We took the comment of the reviewer into consideration and also compared the performance of the lobe detection tools using the median of the gold standard (Supplementary Figure 16). We found that the results did not change, and GraVis still performed best, followed by LobeFinder, PaceQuant and LOCO-EFA (Supplementary Table 5).

It would be helpful to include confidence intervals for the gold standard in Fig 6 or even better include the values of the gold standard as additional data point in the box plots, which could serve as the reference for statistical analysis.

Response:
We incorporated the suggestion of the reviewer and added the mean values of the gold standard as additional data points in the corresponding boxplots (Figure 6, Supplementary Figure 15).

It is difficult to see differences between light and dark gray in Fig S12.

Response:
We thank the reviewer for raising this point, although we would like to point out that in general, the consensus of manually detected tri-cellular junctions is very high, thus most of the circles are dark gray. Nevertheless, we increased the line width of the circles for better visibility.

The mean of the gold standard in Fig 6b and c and Fig S13 b and c is not consistent with the data shown in Fig S11.
Are claps and oryzalin mixed up?

Response:
We would like to apologize for the mix up and thank the reviewer for pointing this out. We relabelled the corresponding plots with the correct treatment names (Figure 6, Supplementary Figure 15).

Fig. 6b/S13b seems to show the results from clasp mutants and Fig. 6c/S13c the oryzalin-treated cells. Why are the medians (shown in the boxes) compared to the mean of the gold standard (and not the median?)

Response:
Boxplots are typically displayed using the median, although we used the means of the gold standard to compute the RMSE of the different lobe detection tools. To avoid confusion, we also added the mean to the boxplots (Figure 6, Supplementary Figure 15, squares).

Given the large variance in lobe numbers detected by experts in clasp mutants: would there still be a significant difference between the median of the manual lobe quantification and the quantification with LobeFinder or PaCeQuant?

Response:
As mentioned above, we also used the median of the manual lobe detection and compared it to the lobe detection tools. We found no change of results, i.e. GraVis still performed best (Supplementary Figure 16, Supplementary Table 5).

Fig S13e: What is the variance in recovery of tri-cellular junctions by the 20 experts? It is surprising that manual recovery was lower than the automatic approaches.

Response:
The pavement cell contours we provided for the manual detection did not include cell wall segments with neighbouring cells, thus making it difficult to estimate the positions of tri-cellular junctions. For this reason, the recovery of junctions by the experts is not at 100%. We plotted the recovery of tri-cellular junctions by the experts for each cell of the gold standard and added the variances (Supplementary Figure 20). We can only do this for the set of manually detected lobes, since we only have single data points for the different tools. It can be seen that the discrepancy between experts is not high, implying that missing tri-cellular junctions were not detected by any of the experts (see Supplementary Figure 20, cell 17/19).

Usability of GraVis for comparative analysis of wild type and mutants (Fig. 7): Fig.7b: By including 80 instead of 30 cells the outcome of the comparative analysis changed dramatically when compared to the data presented in the first draft of the manuscript. Except for the light blue cells distinct clusters are barely visible. It would help to use the different shapes (triangles, squared, pentagons) in the legend, too.

Response:

We added a legend to the PCA in Figure 7b depicting the level of shape complexity for the used centres of mass.

Statistical analysis of (differences in) lobe number is still missing in Fig. 7c.
Lobe numbers seems to be overestimated in CA-ROP2 and RIC1-OX, at least in comparison to the representative images shown in Fig. 7a. Without statistical information it is difficult to interpret the presented data. I, however, doubt that the number of lobes quantified per cell with GraVis will differ between rop4-1, lue1 and RIC1-OX, which I would expect from the shown images (Fig. 7a).

Response:

We would like to highlight that we did a statistical analysis of the data in the manuscript and the Supplementary Material. We used ANOVA to show that there is a significant difference between means of detected lobes between the genotypes. We exchanged the ANOVA with the Kruskal-Wallis test as indicated below by the reviewer. Furthermore, we updated the pairwise testing of differences between the genotypes using Dunn's post-hoc test and added a table with the corresponding adjusted p-values to the Supplementary Material (Supplementary Table 6). Genotypes with no significant difference in means (adjusted p-value > 0.05) were used to create a clustering graph depicting genotypes with similar phenotypes (Supplementary Figure 21).

For statistical analysis, the parametric ANOVA was used, which requires normal distribution of data. The data presented, however, fail normal distribution assumption as indicated by a tendency of medians to lie outside the center of the boxes, and lobe numbers in different genotypes show different levels of variance. Therefore, the statistical analysis should be revisited.

Response:

We thank the reviewer for the comment regarding the statistical analysis. Based on the comment we revisited the statistical analysis and used the nonparametric Kruskal-Wallis test. The comparison of the different genotypes showed that the overall results did not change.

Reviewer #1 (Remarks to the Author):

My earlier concerns have been addressed satisfactorily.

Reviewer #2 (Remarks to the Author):

I acknowledge the effort the authors have made to address my comments.
Overall, the use of visibility graphs presents an interesting novel approach for description of complex shapes. Quantification of the relative completeness of cells and its correlation to cell circularity proves to be a promising method to detect stomata guard cells. Gravis thus will be useful for shape comparison of pavement cells and beyond.

Comments:
The newly added Supplementary Fig 19 presents novel information on the performance of Gravis compared to contending approaches. With all tools being optimized on the input data set their performance is in general very good, and definitely much better than the original data indicated (Fig. 6). I thus recommend to replace Fig. 6 with Supplementary Fig 19 in the main part of the manuscript and to tone down the statement that 'Gravis outperforms contending approaches'. Gravis definitely works comparably well and additionally offers novel functionality, which on its own is suitable for publication. It remains to be tested whether the default settings of Gravis will work equally well on input data sets generated in different laboratories and with different imaging settings or whether tuning also should be done on a case-by-case basis.

Changes in BHI values are not larger than 36% for numbers of nodes that differ by one. When node numbers differ by three or four changes in BHI values increase up to >50%. What is the average difference in node numbers among pavement cells within biological samples, e.g., in the set of 80 analyzed pavement cells per sample in wild type and the different genetic lines? Could the authors please add this information?

Quantification of lobe numbers in the data set used for generation of the gold standard indicates that lobe numbers are higher in clasp-1 mutants than in wild type (Col-0) (Fig. 6, Supplementary Fig. 13, 14, and 15). Application of Gravis to compare pavement cell shapes and lobe numbers in lines with different genetic background (Fig. 7), however, shows an opposite tendency, i.e. reduced lobe numbers in clasp-1 when compared to wild type. What is the explanation for these differences?

REVIEWER COMMENTS

Reviewer #1 (Remarks to the Author):

My earlier concerns have been addressed satisfactorily.

Reviewer #2 (Remarks to the Author):

I acknowledge the effort the authors have made to address my comments. Overall, the use of visibility graphs presents an interesting novel approach for description of complex shapes. Quantification of the relative completeness of cells and its correlation to cell circularity proves to be a promising method to detect stomata guard cells. Gravis thus will be useful for shape comparison of pavement cells and beyond.

Comments:
The newly added Supplementary Fig 19 presents novel information on the performance of Gravis compared to contending approaches. With all tools being optimized on the input data set their performance is in general very good, and definitely much better than the original data indicated (Fig. 6). I thus recommend to replace Fig. 6 with Supplementary Fig 19 in the main part of the manuscript and to tone down the statement that 'Gravis outperforms contending approaches'. Gravis definitely works comparably well and additionally offers novel functionality, which on its own is suitable for publication. It remains to be tested whether the default settings of Gravis will work equally well on input data sets generated in different laboratories and with different imaging settings or whether tuning also should be done on a case-by-case basis.

Response:
We took the suggestion of the reviewer into consideration, and decided to include both the boxplots for the tuned and default parameters for true lobes in the manuscript. The remaining boxplots for detected lobes including tri-cellular junctions for both tuned and default parameters are shown in the Supplementary Material (Supplementary Figure 15). In addition, we adjusted the statement regarding the performance of GraVis throughout the text when we talk about tuned parameters as well as in the abstract and discussion.

Changes in BHI values are not larger than 36% for numbers of nodes that differ by one. When node numbers differ by three or four changes in BHI values increase up to >50%. What is the average difference in node numbers among pavement cells within biological samples, e.g., in the set of 80 analyzed pavement cells per sample in wild type and the different genetic lines? Could the authors please add this information?

Response:

We thank the reviewer for the suggestion and added the number of nodes per µm cell contour into the Supplementary Material (Supplementary Figure 20).

Quantification of lobe numbers in the data set used for generation of the gold standard indicates that lobe numbers are higher in clasp-1 mutants than in wild type (Col-0) (Fig. 6, Supplementary Fig. 13, 14, and 15). Application of Gravis to compare pavement cell shapes and lobe numbers in lines with different genetic background (Fig. 7), however, shows an opposite tendency, i.e. reduced lobe numbers in clasp-1 when compared to wild type. What is the explanation for these differences?

Response:

We would like to point out that we answered the question of the reviewer in the first round of responses. The *clasp-1* data used for comparison of the different genotypes was different from the *clasp-1* data used for the gold standard. More precisely, the *clasp-1* images were captured at different time points. For the gold standard, the images were captured at 96 hours after germination, for the comparison of the different genotypes the images were captured at 120 hours post dissection.

Reviewer #2 (Remarks to the Author):

Thanks to the authors for fully addressing my concerns.

Minor comment:
To eliminate confusion regarding shape characteristics in clasp mutants could you please add information on the time point of analysis in the respective figure legends and/or in the manuscript?

REVIEWER COMMENTS

Reviewer #2 (Remarks to the Author):

Thanks to the authors for fully addressing my concerns.

Minor comment:
To eliminate confusion regarding shape characteristics in clasp mutants could you please add information on the time point of analysis in the respective figure legends and/or in the manuscript?

Response:
We added the information regarding the different *clasp-1* data sets to the relevant results section in the manuscript and the legend of the corresponding figure.
